# Identification of evolutionarily conserved genetic regulators of cellular aging

**DOI:** 10.1111/j.1474-9726.2010.00637.x

**Published:** 2010-12

**Authors:** Gerhard T Laschober, Doris Ruli, Edith Hofer, Christoph Muck, Didac Carmona-Gutierrez, Julia Ring, Eveline Hutter, Christoph Ruckenstuhl, Lucia Micutkova, Regina Brunauer, Angelika Jamnig, Daniela Trimmel, Dietmar Herndler-Brandstetter, Stefan Brunner, Christoph Zenzmaier, Natalie Sampson, Michael Breitenbach, Kai-Uwe Fröhlich, Beatrix Grubeck-Loebenstein, Peter Berger, Matthias Wieser, Regina Grillari-Voglauer, Gerhard G Thallinger, Johannes Grillari, Zlatko Trajanoski, Frank Madeo, Günter Lepperdinger, Pidder Jansen-Dürr

**Affiliations:** 1 Institute for Biomedical Aging Research, Austrian Academy of SciencesRennweg 10, A-6020 Innsbruck, Austria; 2 Institute for Molecular Biosciences, University of GrazHumboldtstrasse 50, 8010 Graz, Austria; 3 Institute for Genomics and Bioinformatics, Graz University of TechnologyPetersgasse 14, 8010 Graz, Austria; 4 Institute for Cell Biology, Salzburg UniversitySalzburg, Austria; 5 Aging and Immortalization Research, Department of Biotechnology, University of Natural Resources and Applied Life SciencesVienna, Austria; 6 Biocenter, Section for Bioinformatics, Innsbruck Medical UniversityInnsbruck, Austria

**Keywords:** aging, evolution, replicative lifespan, replicative senescence, senescence, yeast

## Abstract

To identify new genetic regulators of cellular aging and senescence, we performed genome-wide comparative RNA profiling with selected human cellular model systems, reflecting replicative senescence, stress-induced premature senescence, and distinct other forms of cellular aging. Gene expression profiles were measured, analyzed, and entered into a newly generated database referred to as the GiSAO database. Bioinformatic analysis revealed a set of new candidate genes, conserved across the majority of the cellular aging models, which were so far not associated with cellular aging, and highlighted several new pathways that potentially play a role in cellular aging. Several candidate genes obtained through this analysis have been confirmed by functional experiments, thereby validating the experimental approach. The effect of genetic deletion on chronological lifespan in yeast was assessed for 93 genes where (i) functional homologues were found in the yeast genome and (ii) the deletion strain was viable. We identified several genes whose deletion led to significant changes of chronological lifespan in yeast, featuring both lifespan shortening and lifespan extension. In conclusion, an unbiased screen across species uncovered several so far unrecognized molecular pathways for cellular aging that are conserved in evolution.

## Introduction

A major goal in current aging research is to understand genetic regulation of aging in humans. However, many recent insights into genetic determinants of aging have been gained from studies with short-lived eukaryotic model systems of aging [for recent review, see (Partridge, 2009)], which, unlike human beings, are amenable to molecular genetic analysis in the laboratory. Extrapolation of results obtained in lower model organisms identified cellular pathways such as the insulin/IGF pathway (Holzenberger, 2004; Katic & Kahn, 2005), which are conserved in evolution and appear to modulate aging in a variety of organisms including mammals (Bartke, 2005). Although it is very likely that these very same mechanisms also modulate aging in humans, this has not yet been formally proven. Moreover, extrapolating findings in lower model organisms to more complex organisms, such as humans, bears the inherent risk that some pathways, which are less important or absent there, may be overlooked.

To circumvent this potential problem, we applied an alternative strategy, namely to employ human model systems of cellular aging and senescence as the primary screening system. There is now increasing evidence that the appearance of cellular senescence contributes to the aging of tissues, such as the skin (Dimri *et al.*, 1995; Ressler *et al.*, 2006), the vascular system (Vasile *et al.*, 2001; Minamino *et al.*, 2002; reviewed by Erusalimsky, 2009), and the kidney (Melk & Halloran, 2001). We performed genome-wide RNA profiling on several experimental models for cellular senescence, including stress-induced premature senescence (SIPS), replicative senescence, and other distinct forms of cellular aging, as proposed recently (Lepperdinger *et al.*, 2008; Hackl *et al.*, 2010) and collected the data in a novel database referred to as the GiSAO (genes involved in senescence, apoptosis, and oxidative stress) database. Comparison of the differential gene expression patterns of the models should in turn unveil distinct novel genetic regulators of cellular aging. Potentially, the information obtained from these experiments, when filtered accordingly, should provide molecular information about highly conserved gene expression patterns related to cellular aging. Because of the genome-wide approach, the data collected from cell types as diverse as those employed herein should also uncover recurrent cellular alterations indicative for hitherto unknown biological aging mechanisms. As a further step in evaluation of the functional role of the individually selected genes, those that were also found to be highly conserved during phylogeny were taken to functional tests in yeast deletion strains thereby monitoring the respective lifespan of the mutant organisms.

## Results

### Comparative RNA profiling of aging and senescence in human cellular models

Accumulation of damage is considered a key factor driving cellular aging, and oxidative stress is a common denominator of stress that induces cellular senescence *in vitro* in a variety of cell types (Dumont *et al.*, 2000). To identify key transcriptional events preceding senescence induced by oxidative stress, a comprehensive set of human cellular models were subjected to oxidative stress at doses known to elicit a senescence response. The corresponding model systems are referred to as experimental group 1 (EG1) (Table 1). For comparison, other model systems of cellular aging were also analyzed collectively referred to as experimental group 2 (EG2) (Table 1). On the one hand, we used models of *in vitro* cellular senescence not triggered by oxidative stress, namely replicative senescence of human umbilical vein endothelial cells (HUVEC) and renal proximal tubular epithelial cells (RPTEC). Similarly, we applied primary foreskin fibroblasts (PFF) in which premature senescence was induced by mitochondrial dysfunction (Passos *et al.*, 2007), which is largely independent of oxidative stress (Stockl *et al.*, 2007). On the other hand, three alternative models of cellular aging (included in experimental group 2, see Table 1) were also analyzed: first, we compared CD28^−^ and CD28^+^ CD8^+^ T lymphocytes, based on the assumption that the increased percentage of CD28^+^ T cells result from high level cell proliferation and hence represent a form of *in vivo* senescence (Rufer *et al.*, 2003; Lazuardi *et al.*, 2009). Furthermore, we also analyzed differences in the gene expression profiles of mesenchymal stem cells (MSC) obtained from young and old human donors, another system that permits the impact of human donor age on cellular phenotype to be assessed (Fehrer *et al.*, 2007). Finally, we also analyzed gene expression profiles of prostate stromal cells (PrSC), which were driven to transdifferentiate into premature senescent myofibroblasts by TGFβ1, a process considered a hallmark of the aging human prostate, leading to benign prostatic hyperplasia (Untergasser *et al.*, 2005). RNA was prepared from all samples and analyzed on Affymetrix arrays for whole-genome expression profiling. For validation purposes, the expression levels of 22 genes, which were found to be differentially expressed in a high number of individual experiments, were determined by quantitative reverse transcription–PCR (qPCR). Good concordance between data of the microarray and qPCR analyses was obtained. Linear regression yielded an *r*-value of 0.68 and 362 of 380 individual experimental data pairs sitting within the 95% prediction interval (Fig. S1).

**Table 1:** Overview microarray experiments: experimental model systems: HUVEC, human umbilical vein epithelial cells; PFF, primary foreskin fibroblasts; PrSC, primary prostatic stromal fibroblasts; RPTEC, renal proximal tubular epithelial cells; CD8, CD8^+^ T lymphocytes; MSC, mesenchymal stem cells; reagents: t-BHP (*tert*-butylhydroperoxide), FCCP (carbonyl cyanide p-(trifluoromethoxy)phenylhydrazone), AMP (adenosine monophosphate); number of array hybridizations/samples; number of array experiments for senescence and oxidative stress; EMBL-EBI Array Express accession number

	Controls	Experimental group 1 (oxidative stress)		Experimental group 2 (cellular aging)		
Celltype	No. of arrays	Treatment	No. of arrays	Treatment	No. of arrays	Array Express accession number
HUVEC	6	t-BHP	2	RS	2	E-MEXP-2283
PFF	8	ND	ND	FCCP	2	E-MEXP-2285
				AMP	2	
				Oligomycin	2	
RPTEC	4	ND	ND	RS	1	E-MEXP-2683
				High/low ROS	1	
PrSC	10	t-BHP	2	TGF-β (trans-differentiation)	2	E-MEXP-2167
				20% vs. 3% O_2_	4	
CD8	15	t-BHP	7	CD28^−^/CD28^+^	4	E-MEXP-2345
MSC	4	20% vs. 3% O_2_	2	Young vs. old donor	2	E-MEXP-1506
Total	47		17		18	

RS, replicative senescence; ND, not determined.

### Identification of transcriptional signatures of oxidative stress and cellular aging

**Fig. 1:**
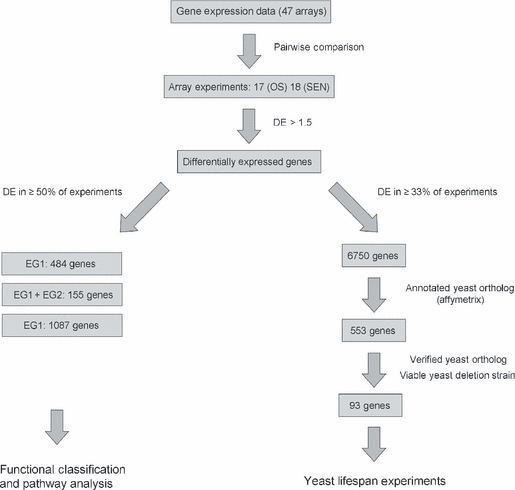
Workflow for candidate gene identification and functional classification. First, gene expression data from 47 microarrays were processed to identify differentially expressed (DE) genes in experimental group 1 (EG1) and experimental group 2 (EG2). The selection of genes for functional classification and pathway analysis, starting from a total of 1566 genes, is shown (left arm). In the right arm of the diagram, the strategy to identify yeast genes for testing in lifespan analysis is depicted.

For computational comparison, normalized whole-genome expression profiles from 47 independent experimental samples were established (Table 1), and hierarchical clustering of the gene expression profiles derived from all experimental human cell models was performed (Fig. S2). Differential expression values were calculated and compiled for further analysis by assembling 35 pairs of individual experiments with regard to cellular aging or oxidative stress (Fig. 1; see also Table S1). Considerable resemblance with respect to sets of differentially expressed genes was observed between HUVEC and RPTEC that have undergone replicative senescence, *in vivo* aged CD8 T cells and MSC, as well as in a compilation of these experimental model systems (Fig. S3).

Several methods have been established to identify genes that are differentially expressed in a homologous set of biological replicates. However, complex datasets such as those described herein, which comprise transcriptional profiles from different cell types derived from differently aged, nonrelated human individuals, often show highly variable transcript levels with regard to the expression of individual genes (Lu *et al.*, 2004; Rodwell *et al.*, 2004; Tan *et al.*, 2005; Lener *et al.*, 2006). This can be either because of variant genetic background or the redundant use of closely related homologous genes. Therefore, we employed a value counting method.

The number a particular gene was found under- or overexpressed was counted, and the list of all genes was sorted according to occurrence. Using this methodology, we identified 586 probe sets representing 484 genes across EG1 with differential expression (DE) higher than ±1.5-fold in more than 50% of individual pair-wise comparisons, and 1423 probe sets representing 1087 genes across EG2, again with the same criteria of DE > ±1.5 in > 50% of experiments, whereas 180 probe sets representing 155 genes were present in both data sets. With respect to a cumulative binomial distribution (CBD), we additionally calculated *P*-values together with the corresponding *q*-values (Table S2) yielding a probability of 0.12 for any probe set to be differentially expressed with senescence and a probability of 0.11 for a probe set to be differentially expressed with oxidative stress. The *q*-values were ≤ 0.0030 in both EG1 and EG2, and *P*-values were ≤ 0.000028 in EG1 and ≤ 0.000085 in EG2 (Table S2). When evaluating both experimental groups along these lines, we revealed a set of 335 candidate genes with DE > ±1.5 in at least 18 pair-wise comparisons (Table S3).

The highest overall score (with most occurrences of DE) was observed for *PAF* (PCNA associated factor) (Table 2A) (Yu *et al.* 2001), a gene recently reported to be involved in DNA repair in HeLa cells (Turchi *et al.*, 2009). Another top scoring gene was SKP2 (Table 2A), encoding a regulatory subunit of an ubiquitin ligase complex, which was recently identified as playing a key role in setting the threshold for stress-induced cellular senescence (Lin *et al.*, 2010). Several additional genes were identified with high scores, such as SLC1A4, a member of the solute carrier family 1 (Table 2A), which so far have not been linked to the senescent phenotype. Of note, four genes involved in the regulation of transcription (BHLHB2, TCF8, RUNX1, and TCEA3) were included in the top 15 genes common to both experimental groups (Table 2A).

**Table 2:** (A) Genes that were found differentially expressed with highest occurrence in both experimental groups, (B) group 1 (oxidative stress), (C) group 2 (cellular aging)

Symbol	Description	Unigene	GenBank	EG1 up	EG1 down	EG2 up	EG2 down	EG1 + EG2DE
(A)								
KIAA0101	KIAA0101/PAF	Hs.81892	NM_014736	7	7	6	7	27
APOBEC3G	Apolipoprotein B mRNA editing enzyme, catalytic polypeptide-like 3G	Hs.660143	NM_021822	0	10	8	8	26
MEST	Mesoderm-specific transcript homolog (mouse)	Hs.270978	NM_002402	4	5	8	8	25
RRM2	Ribonucleotide reductase M2 polypeptide	Hs.226390	BE966236	7	5	4	9	25
SYDE2	Synapse defective 1, Rho GTPase, homolog 2 (*C. elegans*)	Hs.718601	N90719	7	5	5	8	25
TXNIP	Thioredoxin interacting protein	Hs.715525	AA812232	2	9	7	7	25
BHLHB2	Basic helix-loop-helix domain containing, class B, 2	Hs.719093	NM_003670	5	6	10	3	24
BICD1	Bicaudal D homolog 1 (*Drosophila*)	Hs.505202	BC010091	10	3	7	4	24
CYP51A1	Cytochrome P450, family 51, subfamily A, polypeptide 1	Hs.417077	U40053	10	1	10	3	24
HMGCS1	3-hydroxy-3-methylglutaryl-Coenzyme A synthase 1 (soluble)	Hs.397729	NM_002130	9	3	9	3	24
SKP2	S-phase kinase-associated protein 2 (p45)	Hs.23348	BC001441	5	9	3	7	24
SLC1A4	Solute carrier family 1 (neutral amino acid transporter), member 4	Hs.654352	W72527	6	1	11	6	24
TCF8	Transcription factor 8 (represses interleukin 2 expression)	Hs.282113	NM_030751	6	6	6	6	24
C20orf129	Chromosome 20 open reading frame 129	Hs.472716	BC001068	11	1	7	4	23
CD302	CD302 antigen	Hs.130014	NM_014880	7	4	6	6	23
IFI44L	Interferon-induced protein 44-like	Hs.389724	NM_006820	3	7	8	5	23
PDGFD	Platelet derived growth factor D	Hs.352298	NM_025208	5	4	6	8	23
RUNX1	Runt-related transcription factor 1 (acute myeloid leukemia 1)	Hs.675708	BU789637	2	9	9	3	23
TCEA3	Transcription elongation factor A (SII), 3	Hs.446354	AI675780	2	8	4	9	23
TSPAN2	Tetraspanin 2	Hs.310458	AI743596	4	6	7	6	23

Symbol	Description	Unigene	GenBank	EG1 up	EG1 down	EG1 DE
(B)						
KIAA0101	KIAA0101/PAF	Hs.81892	NM_014736	7	7	14
RRM2	Ribonucleotide reductase M2 polypeptide	Hs.226390	BC001886	8	6	14
SKP2	S-phase kinase-associated protein 2 (p45)	Hs.23348	BC001441	5	9	14
TRPV2	Transient receptor potential cation channel, subfamily V, member 2	Hs.279746	NM_015930	4	10	14
BICD1	Bicaudal D homolog 1 (Drosophila)	Hs.505202	BC010091	10	3	13
EIF4A2	Eukaryotic translation initiation factor 4A, isoform 2	Hs.518475	AI332397	10	3	13
NQO1	NAD(P)H dehydrogenase, quinone 1	Hs.406515	NM_000903	9	4	13
C20orf129	Chromosome 20 open reading frame 129	Hs.472716	BC001068	11	1	12
CASP1	Caspase 1, apoptosis-related cysteine peptidase (interleukin 1, beta, convertase)	Hs.2490	U13699	2	10	12
CCNB2	Cyclin B2	Hs.194698	NM_004701	5	7	12
CIRBP	Cold-inducible RNA-binding protein	Hs.634522	AL565767	8	4	12
CKAP2	Cytoskeleton-associated protein 2	Hs.444028	NM_018204	11	1	12
CYBA	Cytochrome *b*-245, alpha polypeptide	Hs.513803	NM_000101	3	9	12
DAAM1	Disheveled-associated activator of morphogenesis 1	Hs.19156	AA890373	8	4	12
EGR1	Early growth response 1	Hs.326035	NM_001964	6	6	12
EIF5A	Eukaryotic translation initiation factor 5A	Hs.534314	NM_001970	11	1	12
ERRFI1	ERBB receptor feedback inhibitor 1	Hs.658370	AW612461	7	5	12
FBXO9	F-box protein 9	Hs.216653	AK095307	8	4	12
HMGCS1	3-hydroxy-3-methylglutaryl-Coenzyme A synthase 1 (soluble)	Hs.397729	NM_002130	9	3	12
IGFBP3	Insulin-like growth factor binding protein 3	Hs.450230	BF340228	4	8	12

Symbol	Description	Unigene	GenBank	EG2 up	EG2 down	EG2 DE
(C)						
SLC1A4	Solute carrier family 1 (neutral amino acid transporter), member 4	Hs.654352	BF340083	11	7	18
ARRB1	Arrestin, beta 1	Hs.503284	BE207758	8	9	17
APOBEC3G	Apolipoprotein B mRNA editing enzyme, catalytic polypeptide-like 3G	Hs.660143	NM_021822	8	8	16
MEST	Mesoderm-specific transcript homolog (mouse)	Hs.270978	NM_002402	8	8	16
CTSC	Cathepsin C	Hs.128065	AI246687	10	5	15
METTL7A	Methyltransferase like 7A	Hs.716437	NM_014033	10	5	15
GLIPR1	GLI pathogenesis-related 1 (glioma)	Hs.205558	AV682252	7	8	15
RAB27B	RAB27B, member RAS oncogene family	Hs.25318	BF438386	11	4	15
TRIB3	Tribbles homolog 3 (Drosophila)	Hs.516826	NM_021158	10	5	15
ABL2	v-abl Abelson murine leukemia viral oncogene homolog 2	Hs.159472	AK025877	9	5	14
EIF4EBP1	Eukaryotic translation initiation factor 4E binding protein 1	Hs.411641	AB044548	8	6	14
ETV1	ets variant gene 1	Hs.22634	BE881590	9	5	14
IFIT3	Interferon-induced protein with tetratricopeptide repeats 3	Hs.714337	AI075407	7	7	14
KLF9	Kruppel-like factor 9	Hs.150557	AI690205	3	11	14
MCC	Mutated in colorectal cancers	Hs.593171	BE967311	3	11	14
PDGFD	Platelet derived growth factor D	Hs.352298	NM_025208	6	8	14
PHGDH	Phosphoglycerate dehydrogenase	Hs.487296	NM_006623	4	10	14
PHLDA1	Pleckstrin homology-like domain, family A, member 1	Hs.602085	AK026181	10	4	14
PTGS2	Prostaglandin-endoperoxide synthase 2	Hs.196384	NM_000963	8	6	14
RAB1A	RAB1A, member RAS oncogene family	Hs.709257	BG530481	4	10	14

**Fig. 2:**
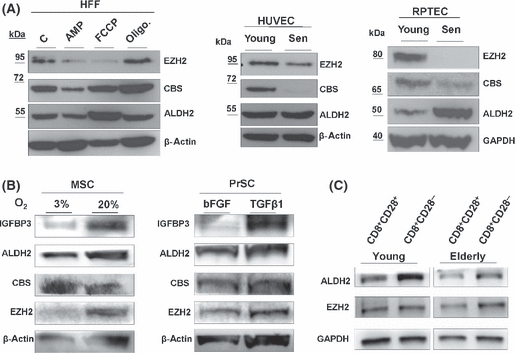
Western blot analysis. Cell lysates were prepared from various model systems employed in this study: young and senescent human umbilical vein endothelial cells and renal proximal tubular epithelial cells, respectively; PFF treated with FCCP, AMP, and oligomycin for three days (A); mesenchymal stem cells cultured at atmospheric conditions of 3% or 20% oxygen; prostate stromal cells treated with either basic fibroblast growth factor (bFGF) or transforming growth factor beta 1 (TGFβ1) (B); CD8 T cells either isolated from a young or an elderly donor and sorted with respect to CD28 (C); Proteins were analyzed by immunoblotting using antibodies to EZH2, ALDH2, cystathionine beta-synthase, and IGFBP3, as indicated. For loading control, antibodies to β-actin and GAPDH were used as indicated.

When both experimental groups were treated separately, the highest scoring genes (with most occurrences of DE) for experimental group 1 (oxidative stress) were genes involved in regulating cell proliferation, such as RRM2, cyclin B2, EGR1, and IGFBP-3 (Table 2B), consistent with the induction of rapid growth arrest by oxidative stress (Chen & Ames, 1994). In addition, several genes were identified for which a role in stress-induced senescence was not known before, such as the translation initiation factors EIF4A and EIF5A (Table 2B). The highest scoring genes (with most occurrences of DE) for experimental group 2 (cellular aging) are shown in Table 2C. Surprisingly, the top 15 genes in this group were distinct from the top scorers obtained in experimental group 1. Moreover, most of the genes identified in this approach were not previously associated with cellular senescence. Hence, this list of genes may contain novel information about potential unidentified regulators of cellular aging, probably including genes that are relevant for cellular aging *in vivo*. The regulation of selected genes was also addressed at the protein level. On the one hand, selected genes were analyzed across all cellular model systems. For example, protein levels were determined for the conserved candidate gene EZH2 (DE = 19; Table S2), coding for a histone methyltransferase that plays a major role in epigenetic regulation of senescence (Bracken *et al.*, 2007.). Consistent with data obtained by RNA profiling, EZH2 protein level was considerably reduced in senescent RPTEC and HUVEC, as well as in PFF after treatment with adenosine monophosphate (AMP) and carbonyl cyanide 4-(trifluoromethoxy)phenylhydrazone (FCCP) but not oligomycin (Fig. 2A). EZH2 levels were not significantly regulated in MSC, PrSC, and CD8 T lymphocytes (Fig. 2B, C) consistent with the results of RNA profiling. Additional protein analyses were performed for relevant proteins selectively in particular model systems. Thus, we confirmed by Western blot upregulation of IGFBP-3 in trans-differentiated PrSC and MSC grown in a hyperoxic atmosphere (Fig. 2B). In additional experiments, we confirmed the upregulation of IGFBP-3 and IGFBP-6 in cellular supernatants by ELISA and the downregulation of IGF1R in CD8^+^CD28^−^ T lymphocytes by antibody assisted flow cytometry (data not shown).

### Pathway analysis

To analyze the large amount of gene expression data in a user-friendly way and for optimal data mining, a database was constructed referred to as the GiSAO database (‘Genes Involved in Senescence, Apoptosis and Oxidative stress’ (Lepperdinger *et al.*, 2008); for details, see ‘Experimental procedures’). Currently, more than a hundred whole-genome array data sets fulfilling MIAME criteria from cellular aging model systems were deposited by this consortium in the GiSAO database (https://gisao.genome.tugraz.at/gisao_web/), also including the datasets of miRNA expression published recently (Hackl *et al.*, 2010). To identify principal biological processes associated with age-related transcriptional changes, we first evaluated top-ranked genes via Pathway Explorer (Mlecnik *et al.*, 2005), a web-accessible tool that allows life scientists to infer the biological meaning behind large gene lists. Pathway analysis for genes that are DE in both experimental groups highlighted the p53 signaling pathway, apoptosis, and cell cycle regulation as the most relevant pathways. The general importance of pathways controlling cell proliferation and cell death for cellular senescence is well established (Mallette & Ferbeyre, 2007). Thus, these observations validate our experimental approach. The analysis identified additional pathways that were significantly overrepresented (*P* < 0.001) in the group of differentially regulated genes, which have not previously been linked to cellular senescence, such as cholesterol biosynthesis, IL-18 signaling, and nitrogen metabolism (Table 3).

**Table 3:** Pathway analysis: functional classification of genes that are differentially expressed in experimental groups 1, 2 or both, using Pathway Explorer (Mlecnik *et al.*, 2005; available online: https://pathwayexplorer.genome.tugraz.at/), gene number attributed to pathway, *P*-values were calculated from complete expression value dataset (54 675 probe sets) with Fisher’s exact test, where gene numbers are given, all *P*-values are < 0.05

Pathway	Gene number EG1	Gene number EG2	Gene number EG1 + EG2
Apoptosis	17	39	7
Cell cycle	15	27	7
p53 signaling pathway	7	32	7
Cell proliferation	*	32	7
Chromosome	14	*	5
Lipid biosynthesis	13	*	4
Cytokinesis	10	*	5
Pyrimidine metabolism	9	*	3
G1 to S cell cycle control	8	*	3
mRNA metabolism	10	*	*
ATP-dependenthelicase activity	9	*	*
Fatty acid metabolism	9	*	*
mRNA processing	9	*	*
D4-GDI signaling pathway	7	*	*
DNA replication	7	*	*
MAPK signaling pathway	*	36	*
Extracellular matrix	*	33	*
Focal adhesion	*	29	*
Enzyme inhibitor activity	*	28	*
Cytokine–cytokinereceptor interaction	*	27	*
Cell growth	*	24	*
Cell surface receptor linked signal transduction	*	24	*
Wnt signaling pathway	*	24	*
Cholesterol biosynthesis	*	*	3
Fructose and mannose metabolism	*	*	3
IL 18 signaling pathway	*	*	2
Nitrogen metabolism	*	*	3
Synthesis and degradation of ketone bodies	*	*	2

* No genes were found for particular pathway or *P* > 0.05.

Pathway data source: KEGG pathway database (http://www.genome.jp/kegg/pathway.html), GenMapp (http://www.genmapp.org/), Biocarta (http://www.biocarta.com/genes).

Pathway analysis was also separately applied to experimental groups 1 and 2. For genes differentially expressed in experimental group 1 (oxidative stress), the highest ranked clusters were related to the regulation of apoptosis and cell cycle, in particular the G1/S transition. Additionally, chromosome maintenance, lipid biosynthesis, D4-GDI signaling, and DNA replication scored highly significant (*P* < 0.001). Among the genes differentially expressed in experimental group 2, top clusters related to apoptosis, MAPK signaling, and extracellular matrix. In addition, highly significant scores (*P* < 0.001) were observed for p53 signaling, enzyme inhibitor activity, cell cycle regulation, cell surface receptor linked signal transduction, and cell growth. In this group, we also noted a high representation of the Wnt signaling pathway and cytokine–cytokine receptor interactions, two pathways previously linked with cellular senescence (Ye *et al.*, 2007; Acosta et al. 2008; Coppe *et al.*, 2010b); however, in both cases, statistical significance was rather weak.

### Functional validation of candidate genes by lifespan analysis in yeast mutants

**Fig. 3:**
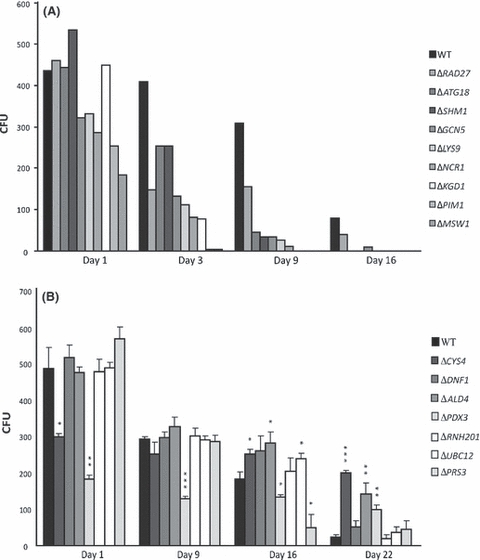
Functional analysis of selected candidate genes in yeast. Lifespan data for the nine yeast mutants with shortest lifespan (A) and for the seven mutants with lifespan extension (B) are shown. (*P < 0.05; **P < 0.01; ***P < 0.001).

Next, potential homologues of the human candidate genes were identified in the genome of budding yeast, *Saccharomyces cerevisiae*. To this end, an extended list of human genes was compiled with differential expression (DE > 1.5) in at least 6 of 17 experiments in experimental group 1 or at least 6 of 18 experiments in experimental group 2 (Fig. 1). Both upregulated and downregulated genes were considered. Starting with a total of 6750 human genes, 553 yeast orthologs were identified, of which only the nonessential genes were considered for further analysis. For the top ranking human genes, 93 nonessential yeast orthologs were identified (Table S4). Subsequently, functional tests regarding long-term proliferation were performed, and the viability of the respective mutant yeast strain was validated. The deletion mutants of the corresponding *S. cerevisiae* homologues were obtained from the EUROSCARF knockout strain collection. A panel of yeast mutant cells in stationary phase was analyzed by chronological lifespan experiments (MacLean *et al.*, 2001; Piper, 2006) involving 22-day culturing in stationary phase as described (Fabrizio *et al.*, 2004; Herker *et al.*, 2004). As control, wild-type strains were also analyzed, which have a mean lifespan of about 11 days under these conditions (Fabrizio *et al.*, 2004; Herker *et al.*, 2004). Twenty-two days after the start of the experiment, the wild-type strain completely ceased growth and was all found dead. The 93 selected yeast mutant strains displayed significantly different survival rates (Table 4), ranging from drastic lifespan shortening to lifespan extension relative to wild-type cells. For a more detailed analysis, the lifespan data were repeated for the nine most short-lived strains containing mutations in ATG18, GCM5, KGD1, LYS69, MSW1, NCR1, TIM1, RAD27, and SHM1. Lifespan shortening relative to the wild-type was highly reproducible (Fig. 3A), with the disruption of KGD1, MSW1, and TIM1 having the greatest effect on lifespan, suggesting that these genes play important roles for survival in stationary culture. Similarly, yeast mutants with extended lifespan were also identified (Table 4), and a significant extension of lifespan was obtained for the mutants ΔCYS4, ΔALD4, and ΔPDX3 (Fig. 3B). Whereas yeast strains mutated in UBC12 and PAS3 showed a trend for extended lifespan, this did not reach statistical significance. The *CYS4* gene encodes a cystathionine beta-synthase involved in the first step of cysteine biosynthesis while Ald4p is the major aldehyde dehydrogenase isoform. *PDX3*, encoding pyridoxine (pyridoxamine) 5′-phosphate oxidase, is involved in the salvage pathway of pyridoxal 5′-phosphate. As a first step to address the relevance of the human orthologs in our cellular senescence models, expression and regulation of ALDH2 and cystathionine beta-synthase (CBS) was analyzed by Western blot. ALDH2 protein level was upregulated in senescent RPTEC and in FCCP-treated PFF (Fig. 2), but remained unchanged in the other experimental models (Fig. 2 and data not shown). Cystathionine beta-synthase protein level was downregulated with senescence in both HUVEC and RPTEC, as well as in AMP-treated PFF (Fig. 2), consistent with the data obtained by RNA profiling. In contrast, CBS was not significantly regulated in the other cellular model systems (data not shown). Together, the data suggest that the levels of ALDH2 and CBS gene products are indeed regulated by the various treatments, in most cases reflecting the differential expression values as elucidated by our initial genomic screening method. The pattern of regulation is complex in both cases, and more work is required to understand the exact contribution of these genes to cellular senescence.

**Table 4:** Effects on chronological aging observed in 93 single deletion strains: deletion strains were assigned to five categories, depending on the effects on survival during aging when compared to the wild-type

Survival during chronological aging (compared to WT)	Single deletion of
Strongly reduced (< 50% of wt)	*KGD1*, *SOD2*, *RPL31A*, *NCR1*, *LYS9*, *SHM1*, *GCN5*, *HAP3*, *PIM1*, *PRY1*, *ATG18*, *CYC1*, *RPL13B*, *MSW1*, *RAD27*, *YDC1*, *TIF3*
Slightly reduced(> 50% and < 85%)	*CLB1*, *CLB2*, *APM1*, *SYM1*, *IDP2*, *GSY2*, *ALT2*, *SIZ1*, *TIF1*, *TIF2*, *NPL3*, *PTC5*, *RNR3*, *ERG24*, *MRT4*, *MSH6*, *NUP170*, *HAT1*, *INP53*, *SSO1*, *TWF1*, *SGT2*, *LHS1*, *RPL37A*, *ARF1*, *DHP5*, *CHL1*, *TRX1*, *UBC8*, *CHD1*, *SPE2*, *HRT3*, *MRE11*, *MSH2*, *DBP1*, *SSN3*, *SSF1*
Not affected(same as wt ±15%)	*HTA2*, *AIP1*, *HXK1*, *SER1*, *YPK2*, *UBC11*, *ENO1*, *RPL22B*, *RPL43B*, *PEX13*, *REV3*, *SYG1*, *MSH5*, *ERP6*, *TPK1*, *PER1*, *URA2*, *SLH1*, *CHK1*, *ALD5*, *CAF40*
Slightly increased (> 115% and < 150%)	*MAD2*, *PCH2*, *YTA7*, *PHO89*, *UGA1*,*DIE2*, *CAF17*, *HMT1*, *ALG5*, *SSA3*, *LSB6*
Strongly increased (> 150%)	*CYS4*, *DNF1*, *ALD4*, *PDX3*, *RNH201*, *UBC12*, *PRS3*

## Discussion

The here presented analysis has identified gene expression signatures of cellular aging, which are conserved between different human tissues. The experimental design was based on the assumptions that (i) genes that are differentially regulated in human cellular aging and senescence are trustworthy candidates for modulators of aging and that (ii) the ability of a certain gene to restrict lifespan in yeast suggests that this gene has a role in modulating the rate of aging that potentially extends beyond yeast. This analysis revealed several genes and molecular pathways already known to be involved in aging, thereby validating the screen. In addition, a reasonable number of novel genes and a few novel pathways were identified that have not been linked to aging before. Based on these findings, new experimental approaches to study human aging will become possible.

### Model systems for cellular aging

According to current hypotheses, aging at the cellular level contributes significantly to organismic aging, and for a better understanding of human aging, cellular model systems are indispensible (Campisi, 2005; Toussaint *et al.*, 2002a, 2002b). Several model systems for cellular aging have been developed, and a representative selection of these models was applied for the present study: replicative senescence, a process of cellular aging *in vitro* that is primarily driven by telomere erosion, is a classic experimental system considered relevant for the aging of tissues with high regenerative potential (von Figura *et al.*, 2009). In addition to replicative senescence, most if not all primary human cells can be driven into premature senescence by various stressors, in a process which does not necessarily involve telomere shortening. It is assumed that SIPS is relevant for aging of both mitotic and postmitotic tissues (Toussaint *et al.*, 2002a, 2002b). The importance of SIPS for *in vivo* aging is supported by the fact that SIPS can be induced *in vitro* by factors that are well-known risk factors for age-associated degeneration of the corresponding tissue *in vivo*. For example, vascular endothelial cells undergo SIPS in response to oxidative stress, high glucose, shear stress, and incubation with oxidized LDL, all of which are known risk factors for age-associated vascular dysfunction and disease (Erusalimsky & Kurz, 2006; Goligorsky *et al.*, 2009). Three additional model systems of cellular aging, with a particular focus on aging *in vivo*, were included in the analysis: first, we compared CD28^−^ and CD28^+^ CD8^+^ T lymphocytes, described as one of the rare models for *in vivo* senescence (Rufer *et al.*, 2003; Lazuardi *et al.*, 2009); second, we compared MSC freshly obtained from young and old human donors (Fehrer *et al.*, 2007); finally, PrSC were included, which can be transdifferentiated into premature senescent myofibroblasts, a process considered a hallmark of the aging human prostate (Untergasser *et al.*, 2005). These cellular model systems were developed by the participating laboratories and are not easily available elsewhere. Information on genetic regulators of cellular aging obtained from these systems is considered particularly valuable, given the scarcity of knowledge on molecular genetic processes involved in human aging.

### The GiSAO database

Other resources have been created to systematically compare the influence of the genome on aging processes. Of note, the Human Ageing Genomic Resources (HAGR) (de Magalhaes *et al.*, 2005) represent a systematic collection of genomic data relevant for aging processes. It consists of the database GenAge featuring genes associated with aging and longevity in short-lived model organisms and humans, and AnAge, a database of aging in animals, featuring information on aging processes in over 4000 animal species (de Magalhaes *et al.*, 2009a, 2009b). The GiSAO database established in the current communication focuses on genetic regulation of aging at the cellular level, based on human gene expression data, and allows the identification of evolutionarily conserved regulators of cellular aging. In our view, information contained in the GiSAO database conveniently complements the data on organismic aging available in the HAGR. We have compared data sets in both studies. Indeed, several genes with a high score in the meta-analysis described earlier (de Magalhaes *et al.*, 2009a, 2009b), including TXNIP, S100A4, and MT1F, featured prominently in our analysis described here (Table S3). In turn, 15 genes taken from Table S3, including IGFBP-3, PTGS2, EGR1, FOS, ATM, DDIT3 (DE ≥ 20) are also highlighted as potential regulators of aging in the HAGR database. Hence, future attempts to link both resources are warranted.

### Confirmation of known association of genes/pathways with aging

Several of the pathways highlighted in the current analysis have been associated with aging in cellular senescence and/or animal models before. For example, apoptosis regulation (Campisi, 2003), MAP kinase signaling (Maruyama *et al.*, 2009), p53 signaling (Zuckerman *et al.*, 2009), control of cell proliferation and cell cycle (Chen, 2000), cytokine–cytokine receptor interaction (Coppe *et al.*, 2010a), and Wnt signaling (Ye *et al.*, 2007) are thought to contribute to cellular senescence and aging. In addition, several of the genes identified in the unbiased screen described here were found to play important roles as regulators of senescence. Thus, Skp2 (Lin *et al.*, 2010) and EZH2 (Bracken *et al.*, 2007) are known to contribute to the senescence response in various cell types. Work in the consortium established the genes coding for IGFBP-3 (Kim et al., 2007; Muck *et al.*, 2008), glutaminase (Unterluggauer *et al.*, 2008), and IGFBP-6 (Micutkova *et al.*, submitted) as functional regulators of cellular senescence. The prominent appearance of these pathways and genes in our screening results serves as positive control and indicates that the screening procedure was adequate.

### Identification of novel genes/pathways related to aging

Some interesting human genes, e.g. genes related to cholesterol biosynthesis, came up in our screen, which have not been related to aging before. Similarly, genes related to the regulation of focal adhesion, extracellular matrix, mRNA metabolism, fatty acid metabolism, and lipid biosynthesis were not known to be associated with aging processes. Although many of these genes could not be analyzed in yeast because there are no obvious orthologs in the yeast genome, these observations will stimulate future research on the role of the respective genes/pathways both in short-lived model organisms of aging and in human cellular aging.

Using extension of the chronological lifespan in yeast, which depends far less on cell-to-cell communication compared to higher organisms, as an additional screening tool, we expected to identify primarily basic cell autonomous mechanisms of aging. In this category, three genes particularly stood out, as follows: (i) ALD4, the major (mitochondrial) aldehyde dehydrogenase isoform, which is involved in the degradation of various amino acids and fatty acids in yeast, whereas the human homolog ALDH2 acts in the major oxidative pathway of alcohol metabolism; (ii) CYS4, cystathionine beta-synthase, that carries out the first committed step in cysteine biosynthesis, whereas the human homolog CBS catalyzes the conversion of homocysteine to cystathionine, the first step in the trans-sulfuration pathway; (iii) Pyridoxine (pyridoxamine) phosphate oxidase (PDX3), known to play a role in the synthesis of pyridoxal 5′-phosphate in yeast, similar to the human homolog pyridoxamine 5′-phosphate oxidase (PNPO), which catalyzes the terminal, rate-limiting step in the synthesis of pyridoxal 5′-phosphate, also known as vitamin B6. Interestingly, CBS requires vitamin B6 as a cofactor for the conversion of homocysteine to cystathionine in human cells, suggesting that CBS and PNPO influence aging via the same pathway. Importantly, none of these pathways have been linked to aging before, and future studies are required to address the role of these proteins in multi-cellular aging model systems including humans. As a first step to validate their relevance in human cellular senescence, expression levels were analyzed in cellular extracts where antibodies were available. We found differential regulation of ALDH2 and CBS protein levels in several of our model systems (Fig. 2). Accordingly, further studies are warranted to determine their role in cellular senescence.

## Experimental procedures

### Cell isolation, cultivation, and characterization

Human diploid fibroblasts from foreskin were seeded in 10-cm dishes at a density of 200,000 cells per dish and treated in parallel with the following compounds: untreated, vehicle treated (ethanol), oligomycin (8 μmol L^−1^ final concentration), FCCP (3 μmol L^−1^ final concentration), and AMP (3 μmol L^−1^ final concentration). Every day, medium was replaced and inhibitory compounds freshly added. This treatment leads to premature senescence within 14 days (data not shown, see also (Stockl *et al.*, 2006, 2007; Zwerschke *et al.*, 2003). RNA was prepared after 72 h of incubation and analyzed by RNA profiling as described earlier.

Human umbilical vein endothelial cells were isolated from human umbilical veins and cultured in Endothelial Cell Basal Medium (Lonza, Basel, Switzerland) supplemented with endothelial cell growth medium (EGM) Single Quots (Lonza), containing hEGF 0.5 mL, hydrocortisone 0.5 mL, GA-1000 0.5 mL, BBE 2.0 mL, and FBS 10.0 mL. The cells were subcultured by trypsination with trypsin-EDTA (Gibco Life Technologies, Vienna, Austria), seeded on cell culture dishes coated with 0.2% gelatine and grown at 37°C at ambient atmosphere containing 5% CO_2_. Cells were passaged at a ratio of 1:5 in regular intervals. At later passages, the splitting ratio was reduced to 1:3 and 1:2, respectively. Cells were passaged before reaching 70–80% confluence. Population doublings (PDL) were estimated using the following equation: *n*= (log10 *F* − log10 *I*)/0.301 (where *n* is the population doublings, *F*, number of cells at the end of one passage, and *I*, number of cells that were seeded at the beginning of one passage). After 50 PDL, the cells reached growth arrest, and the senescent phenotype was verified by staining for senescence-associated beta galactosidase, which was positive for ≥ 95% of cells.

Human primary PrSC were established and trans-differentiation induced by TGF-beta1 treatment, as described (Untergasser *et al.*, 2005). Briefly, PrSC were derived from prostate cancer patients who have not received hormonal therapy (*n* = 3, age 65–72) after obtaining written informed consent. After radical prostatectomy and inspection by the pathologist, two tissue wedges showing no histological signs of malignancy were removed from the transition zone. These explants were minced into organoids of ∼ 1 mm^3^ and seeded on uncoated plastic material in stromal cell growth medium containing insulin, human basic fibroblast growth factor, 5% fetal calf serum, and gentamycin and amphotericin-B as antibiotics (SCGM; Lonza). Explants were maintained at 37°C in a humidified atmosphere of 5% CO_2_. These conditions produce a homogeneous fibroblast cell population after 7 days of culture. When cells reached 70% confluence, they were split at a 1:3 ratio using trypsin-EDTA to expand the population. In all experiments, cells of passage 2–4 were used directly from culture (not previously frozen).

Renal proximal tubular epithelial cells were cultivated as recently reported (Wieser *et al.*, 2008). In brief, within 24 h after surgery, tissue from the renal cortex was fragmented and incubated at 37°C for 15–20 min in DMEM/Ham’s F12 (1:1) (Biochrom KG, Berlin, Germany) containing 1 mg mL^−1^ collagenase type IV (PAN-BioTech GmbH, Aidenbach, Germany) and 1 mg mL^−1^ trypsin-inhibitor (Sigma, Vienna, Austria). After being passed through a 105-μm nylon mesh, the filtrate was centrifuged, washed twice with phosphate-buffered saline (PBS), resuspended in medium, and dispensed into roux-flasks (Nunc, Wiesbaden, Germany). 24 hours thereafter medium was changed. The initial passage of confluent cells after 3–5 days was considered as PDL zero. Cells were passaged (1:2 to 1:4) at confluence, using 0.25% trypsin/0.02% EDTA, which was inactivated with 1 mg mL^−1^ trypsin-inhibitor. Cumulative PDL was calculated as a function of passage number and split ratio (4). Medium consisted of DMEM/Ham’s F12 (1:1) supplemented with 4 mm l-glutamine, 10 mm HEPES buffer, 5 pm tri-iodothyronine, 10 ng mL^−1^ recombinant human EGF, 3.5 μg mL^−1^ ascorbic acid, 5 μg mL^−1^ transferrin, 5 μg mL^−1^ insulin, 25 ng mL^−1^ prostaglandin E1, 25 ng mL^−1^ hydrocortisone, and 8.65 ng mL^−1^ sodium selenite (all from Sigma). After 24 PDL, the cells reached growth arrest, and the senescent phenotype was verified by staining for senescence-associated beta galactosidase, which was positive for ≥ 95% of cells. Cells at intermediate passage could be divided into two groups concerning their redox status, as monitored by dichlorofluorescein diacetate (DCFDA) staining. To address the significance of this distinction, RPTECs were sorted into DCFDA^bright^ and DCFDA^dim^ subpopulations, which were analyzed separately.

Mesenchymal stem cells were isolated from the iliac crest of systemically healthy individuals (young donor, age 5; elderly donor, age 56), which had been harvested for reconstructive bone surgery of defects within other areas of the body as described previously (Fehrer *et al.*, 2007). Briefly, a small biopsy of substantia spongiosa osseum, which otherwise would have been discarded based on necessary bone for molding and re-contouring prior to insertion into the recipient site, was taken to further investigation under an Institutional Review Board-approved protocol after having obtained the parents’ and the respective patient’s written consent. After surgery, the bone was transferred into minimal essential medium (MEM) supplemented with 20% heat-inactivated fetal calf serum, 100 units mL^−1^ penicillin, and 100 μg mL^−1^ streptomycin (growth medium) for transportation from the operation theater to the clean room at room temperature. The biopsies were fragmented, and marrow cells were isolated from pieces (20–100 mm^3^) by centrifugation (400 *g*, 1 min). After centrifugation, the remaining pieces were treated with collagenase (2.5 mg mL^−1^ in MEM) for 2–3 h at 37°C, 20% O_2_, and 5% CO_2_. Thereafter, the specimen was again centrifuged (400 *g*, 1 min). Cells were resuspended and loaded on a Ficoll-Paque Plus® gradient and centrifuged at 2500 *g* for 30 min. Cells were harvested from the interphase (density < 1.075 g mL^−1^), washed, and collected by centrifugation (1500 *g*, 15 min). Cells were cultured at a density of 0.2–0.5 × 10^6^ cells cm^−2^ at either 20% or 3% O_2_ in combination with 5% CO_2_ and 37°C (HeraCell240 – Heraeus, Thermo Scientific, Vienna, Austria; Thermo Electron Forma Series II, 3110). After 24 h, the nonadherent cell fraction was removed by washing twice with PBS. After the primary culture had reached approximately 30–50% confluence, cells were washed twice with PBS and subsequently treated with 0.05% trypsin/1 mm EDTA for 3–5 min at 37°C. Cells were harvested, washed in MEM, and further expanded at a density of 50 cells cm^−2^.

Isolation of CD8^+^CD28^+^ and CD8^+^CD28^−^ T cells from peripheral blood of apparently healthy young (< 35 year, *n* = 6, mean age 29, range 26–35) and elderly (> 65 year, *n*= 10, mean age 72, range 66–87) donors was performed by preparing peripheral blood mononuclear cells (PBMCs) by Ficoll-Paque PLUS (Amersham Biosciences) density gradient centrifugation as approved by the Ethics committee of Innsbruck Medical University. CD8^+^ T cells were negatively selected from the obtained PBMC fraction by applying the magnetic separation protocol CD8^+^ T cell isolation kit II (depleting CD4, CD14, CD16, CD19, CD36, CD56, CD123, TCRγ/δ, and CD235a; Miltenyi Biotec, Bergisch Gladbach, Germany) according to the manufacturer’s instructions. Subsequently, purified CD8^+^ T cells were stained with an allophycocyanin (APC)-conjugated αCD28 monoclonal antibody (mAb) and split into CD8^+^CD28^+^ and CD8^+^CD28^−^ T-cell populations using αAPC MicroBeads (Miltenyi Biotec) by passing the cell suspension through a positive selection column (LS; Miltenyi Biotec) mounted in a magnetic field. The CD8^+^CD28^−^ T-cell fraction was then re-incubated with αAPC MicroBeads and run over a fresh LS-column to increase purity. For phenotypic analysis, purified T-cell fractions were labelled with a combination of mAbs (αTCRαβ-FITC, αCD16-PE, αCD4-PerCP, αCD8-PE-Cy7, αCD28-APC, and αCD3-APC-Cy7; all BD Biosciences, Heidelberg, Germany) and analyzed on a FACSCanto II (BD Biosciences) revealing that the described isolation protocol yields population homogeneities of > 95%.

### Applying oxidative stress by tBHP treatment

Human umbilical vein endothelial cells, PrSC, RPTEC, and CD8^+^ T lymphocytes were treated by exposure to sublethal doses of tBHP (Unterluggauer *et al.*, 2003). Induction of senescence by repeated exposure to sublethal levels of tBHP takes 10–14 days (Unterluggauer *et al.*, 2003, and data not shown). To identify early transcriptional events involved in the senescence response, cells were analyzed 3 days after starting the treatment.

### RNA Isolation, whole-genome array analysis of mRNA expression, and quantitative PCR

RNA was isolated using either Tri Reagent (Sigma-Aldrich) or performing homogenization in 4.2 m guanidinium thiocyanate before phenol extraction and ethanol precipitation (Chomczynski & Sacchi, 1987), followed by LiCl precipitation at a final concentration of 4.5 m. Whole-genome expression analysis was carried out on GeneChip HG-U133 Plus 2.0 Arrays (Affymetrix) by MFT Services, Tuebingen, Germany.

### Bioinformatics, clustering, and visualization of array data

Array raw data were normalized via CARMAweb (Rainer *et al.*, 2006) using the gcrma algorithm (Wu *et al.*, 2004). Hierarchical clustering of samples and genes (Euclidian distance, average linkage) was performed after filtering out genes with low variance on a subset of 20 000 genes with the MeV program package (Saeed *et al.*, 2006) available online: http://www.tm4.org/mev/).

To evaluate the probability of observing an elevated number of under- or overexpressed gene occurrences, the distinct number of occurrences in which the gene is under- or overexpressed in a respective group of experiments was determined. The resulting gene list was ranked according to occurrences with respect to either age/senescence-related model systems or oxidative stress–derived data sets as well as a gene list of highly under- or overrepresented candidates present in both cases.

Statistical analysis to identify significant probe sets was performed as described by de Magalhaes *et al.* (2009a,b);: For each probe set, a *P*-value was calculated with the cumulative binomial distribution (CBD): 
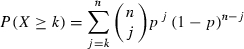
 providing the probability P for a probe set to be as often or more often differentially expressed than the times k, it was actually differentially expressed in n experiments. The threshold for differential expression of a probe set was defined as a fold change between samples ≥ ±1.5. Applying statistical analysis based on CBD, the probability *P* that any probe set is differentially expressed with senescence or oxidative stress was defined as the average of differentially expressed probe sets in the experimental group senescence or oxidative stress divided by the number of all probe sets per whole-genome analysis.

The *q*-value was calculated for each probe set using Storey’s false discovery rate (FDR) approach with the bootstrapping method (Storey *et al.*, 2004). The robust parameter was used to make the *q*-values more accurate for small *P*-values (Storey, 2002). Statistical computation was carried out using the statistical framework R (R-Team, 2007) version 2.5.1. Bioconductor *q*-value package version 1.10.0 (Storey & Tibshirani, 2003) was used to calculate the FDR.

Differentially expressed genes were grouped into protein families associated with characterized pathways applying Pathway Explorer ((Mlecnik *et al.*, 2005), available online: https://pathwayexplorer.genome.tugraz.at/), *P*-values were calculated from the complete expression value dataset (54 675 probe sets) with Fisher’s exact test, pathway data sources: KEGG pathway database (http://www.genome.jp/kegg/pathway.html), GenMapp (http://www.genmapp.org/), Biocarta (http://www.biocarta.com/genes/).

### Database

GiSAO.db (https://gisao.genome.tugraz.at) is a web-based database system for storing and retrieving data of genes involved in senescence, apoptosis, and oxidative stress. The application is based on a three-tier architecture consisting of a web interface, business logic, and a database. The web interface enables data input and presentation. It was implemented by using Struts framework (http://struts.apache.org/) with Java Server Pages (http://java.sun.com/products/jsp/). The business logic, which is responsible for data processing is an Enterprise JavaBeans 3 (http://java.sun.com/products/ejb/) application deployed on JBoss (http://www.jboss.org/jbossas/) application server. The data are stored in an Oracle database, a relational database management system.

The GiSAO database contains normalized gene expression values obtained from experiments evaluated with the aid of Affymetrix arrays. Gene expression values of each experiment can be displayed and compared with the gene expression values of two or more microarray experiments. Additionally, experimental data of follow-up experiments regarding candidate genes, such as qPCR or Western Blot analysis, are entered into the GiSAO.db. Furthermore, GiSAO.db contains two types of orthologue data: orthologue data provided and updated by Affymetrix from cross reference tables linking *Saccharomyces cerevisiae*, *Caenorhabditis elegans*, *Drosophila melanogaster*, *Mus musculus* and *Homo sapiens* facilitate comparative genomic analyses, as well as orthology data computed and entered manually. Besides mRNA expression profiles, also data from proteome analysis and further validation with respect to functional analyses together with information from public resources are available for distinct candidate genes. Moreover, external links lead to orthologs of HomoloGene (http://www.ncbi.nlm.nih.gov/homologene) and InParanoid (http://inparanoid.sbc.su.se/cgi-bin/index.cgi). The gene IDs are linked to their respective database, such as Entrez Gene or RefSeq. GiSAO.db also provides gene annotation (Gene Symbol, Gene Name, etc.) and GO terms (http://www.geneontology.org/). Finally, KEGG pathways (http://www.genome.jp/kegg/) can be displayed and data can be exported in various formats.

### Identification of human orthologues in *Sacharomyces cerevisiae*

Either previously assigned cross references distinctly referring to homologous gene identifiers from other species provided in the array annotation tables accompanying the Affymetrix gene chip or data from reciprocal-best-fit-protein-homology searches (Moreno-Hagelsieb & Latimer, 2008) were employed to define the closest homologous gene pair between man and yeast. Only orthologous genes, which were found nonessential after genomic deletion in yeast laboratory strains (http://mips.helmholtz-muenchen.de/proj/eurofan/eurofan_1/b0/search/simpleSearch.html) were selected for further functional analyses.

### Lifespan analysis of gene disruption mutants of *Sacharomyces cerevisiae*

Experiments were carried out in BY4741 (MATa *his3Δ1 leu2Δ0 met15Δ0 ura3Δ0*) as the wild-type strain and respective null mutants, obtained from Euroscarf. Strains were grown at 28°C on SC medium containing 0.17% yeast nitrogen base (Difco), 0.5% (NH_4_)_2_SO_4_ and 30 mg L^−1^ of all amino acids (except 80 mg L^−1^ histidine and 200 mg L^−1^ leucine), 30 mg L^−1^ adenine, and 320 mg L^−1^ uracil with 2% glucose. For all experiments, yeast cells were grown at 28°C and 145 rpm. For chronological lifespan experiments, cultures were inoculated at an OD_600_ of 0.1, and aliquots were taken to perform survival plating at indicated time points.

### Statistics

All values were expressed as means ± standard deviation of the mean. Statistical differences of experimental scores were evaluated using Student’s *t* tests. Differences were considered significant when *P* was < 0.05.

## Supporting Information

**Fig. S1** Validation of microarray analysis: expression values of 22 genes exhibiting differential expression of > 1.5-fold in more than 18 of 37 experimental pairs were assessed by quantitative reverse transcription–PCR.

**Fig. S2** Hierarchical clustering of genome-wide gene expression profiles derived from all experimental human cell models (47 individual datasets).

**Fig. S3** Comparison of numbers of differentially expressed probe sets (DE > 1.5) between individual members of EG2.

**Table S1** Experimental systems: HUVEC (human umbilical vein epithelial cells), PFF (primary foreskin fibroblasts), PrSC (primary prostate stromal cells), RPTEC (renal proximal tubular epithelial cells), CD8 (CD8^+^ T lymphocytes), MSC (mesenchymal stem cells), Reagents: bFGF: basic fibroblast growth factor, TGFβ: transforming growth factor-β 1, t-BHP (tert-butylhydroperoxide), FCCP (carbonyl cyanide 4-(trifluoromethoxy)phenylhydrazone), AMP (adenosine monophosphate), DCFDA (2′,7′-dichlorofluorescein diacetate), number of up- and downregulated probe sets for each array experiment (DE > 1.5, total probe set number: 54 675 for Affymetrix U133 Plus 2.0 array)

**Table S2** Genes that are differentially expressed (DE > 1.5) in ≥ 50% of the experiments for experimental groups 1 and 2, numbers for up- and downregulation in EG1 and EG2, *P*-values (cumulative binomial distribution), *q*-values (Storeys FDR method)

**Table S3** Three hundred and thirty-four genes differentially expressed (DE > 1.5) with the highest combined score for both experimental groups (≥ 18), provided as Excel file

**Table S4** Highest scoring genes, which were found differentially expressed (DE > 1.5) in both experimental groups, for which an orthologous nonessential gene in yeast could be identified (according to orthology information provided by Affymetrix Inc

As a service to our authors and readers, this journal provides supporting information supplied by the authors. Such materials are peer-reviewed and may be re-organized for online delivery, but are not copy-edited or typeset. Technical support issues arising from supporting information (other than missing files) should be addressed to the authors.
