# Supplementary figures and images for: Identification of evolutionarily conserved genetic regulators of cellular aging
Source: Aging Cell. 2010 Dec;9(6):1084–97. doi: 10.1111/j.1474-9726.2010.00637.x (PMC2997327; doi:10.1111/j.1474-9726.2010.00637.x)

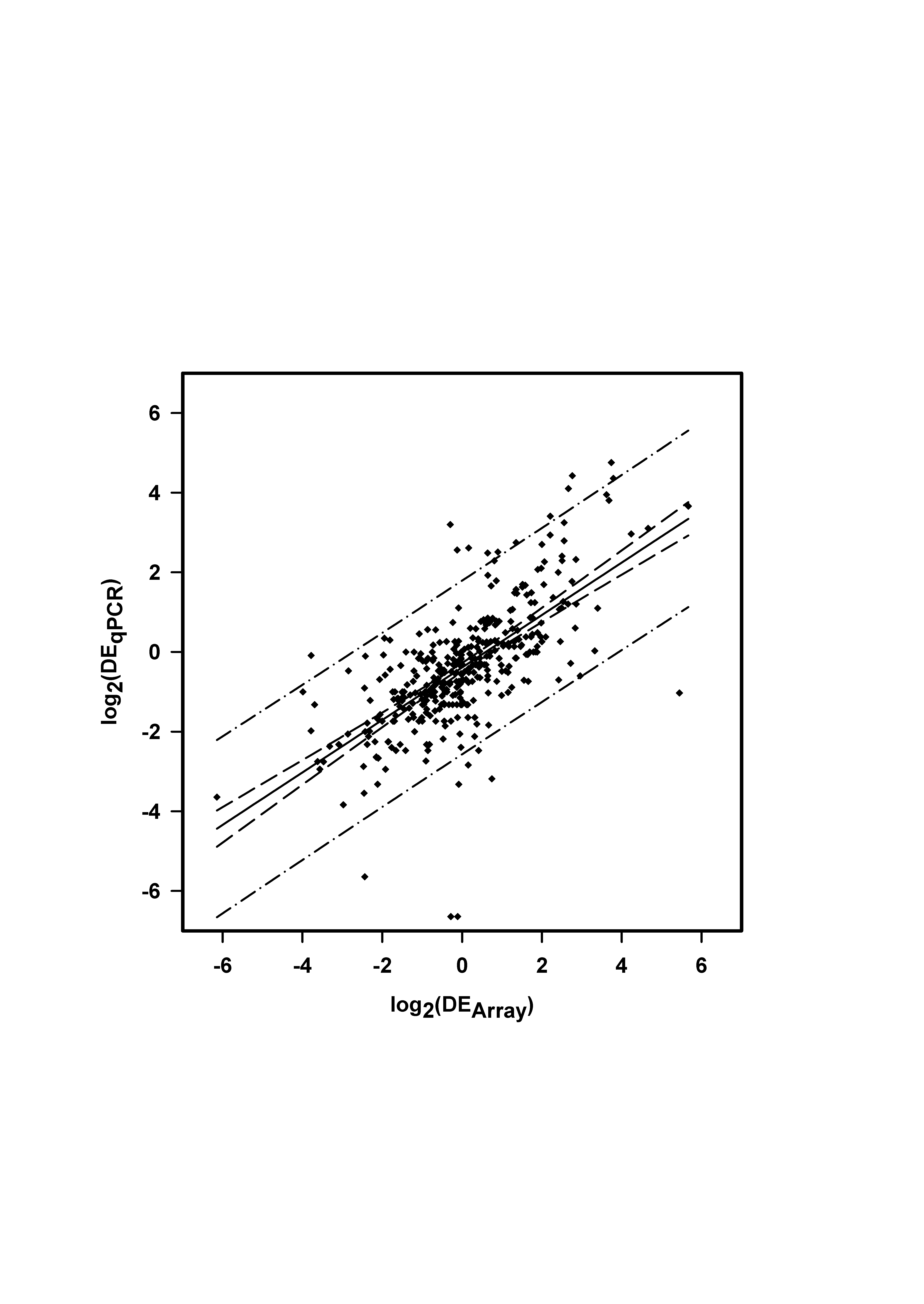

Supplement: Supplementary file 1 [file acel0009-1084-SD1.jpg]

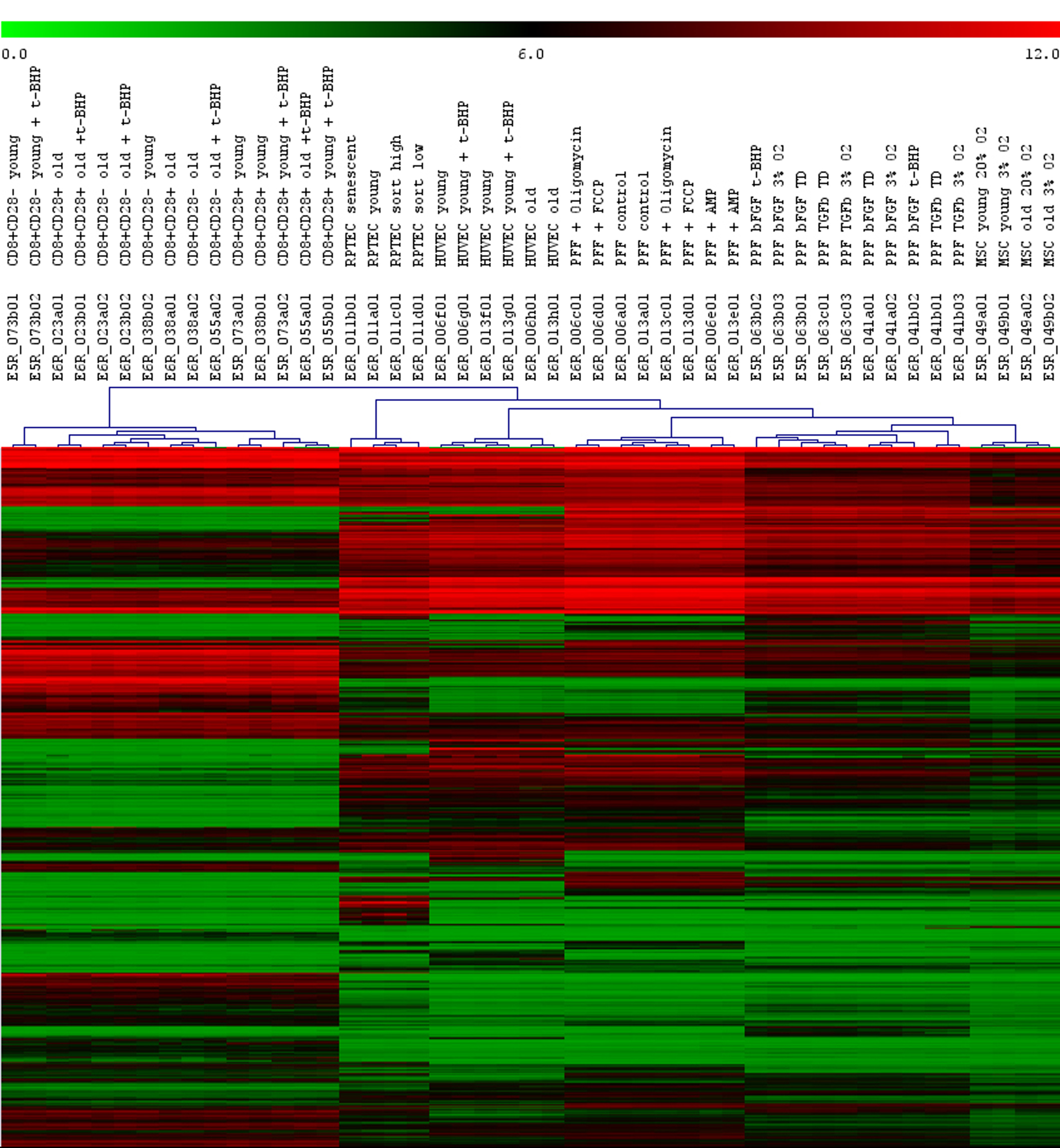

Supplement: Supplementary file 2 [file acel0009-1084-SD2.jpg]

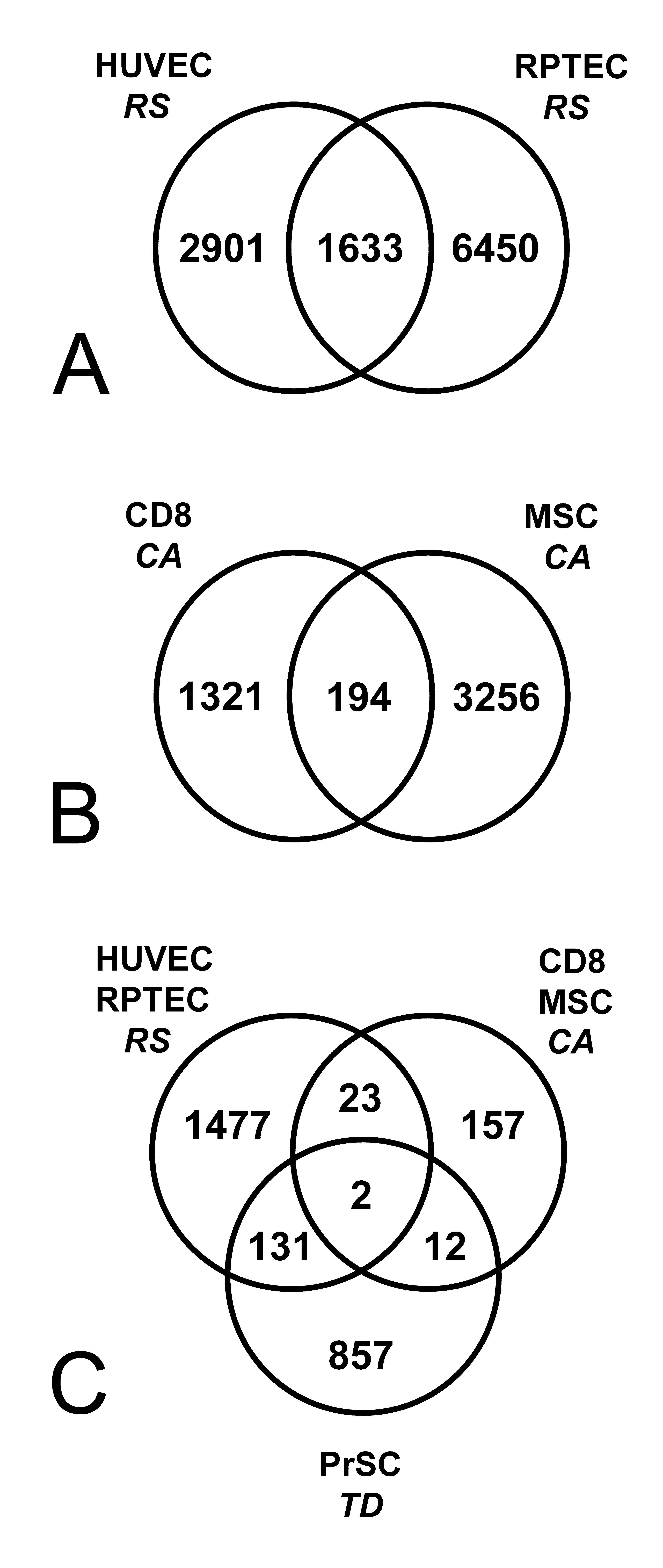

Supplement: Supplementary file 3 [file acel0009-1084-SD3.jpg]
