# Supplementary material for: Identification of evolutionarily conserved genetic regulators of cellular aging
Source: Aging Cell. 2010 Dec;9(6):1084–97. doi: 10.1111/j.1474-9726.2010.00637.x (PMC2997327; doi:10.1111/j.1474-9726.2010.00637.x)
Supplement: Supplementary file 4 [file acel0009-1084-SD4.doc]

| **Cell Type** | **Experimental group 1**  **(oxidative stress)** | | | **Experimental group 2**  **(senescence)** | | |
| --- | --- | --- | --- | --- | --- | --- |
|  | **experiment** | **probe sets**  **upreg. (DE>1.5)** | **probe sets**  **downreg. (DE>1.5)** | **experiment** | **probe sets**  **upreg.**  **(DE>1.5)** | **probe sets**  **downreg. (DE>1.5)** |
| **HUVEC** | t-BHP / control | 1321 | 1351 | senescent / young | 3298 | 3275 |
|  | t-BHP / control | 1194 | 1060 | senescent / young | 3522 | 3237 |
| **RPTEC** |  | | | senescent / young | 4301 | 3782 |
|  |  |  |  | DCFDA^bright^ /  DCFDA^dim^ | 4168 | 2869 |
| **PrSC** | t-BHP / control | 1940 | 2287 | TGFβ / control | 1349 | 995 |
|  | t-BHP / control | 1474 | 2207 | TGFβ / control | 3683 | 4453 |
|  | 20% O_2_ / 3% O_2_ | 1790 | 2103 |  | | |
|  | 20% O_2_ / 3% O_2_ | 1599 | 1963 |  |  |  |
|  | 20% O_2_ / 3% O_2_ (TGF-ß) | 1187 | 1152 |  |  |  |
|  | 20% O_2_ / 3% O_2_ (TGF-ß) | 2032 | 2059 |  |  |  |
| **PFF** |  | | | Oligomycin / control | 1324 | 1755 |
|  |  |  |  | Oligomycin / control | 1231 | 1233 |
|  |  |  |  | FCCP / control | 1329 | 1025 |
|  |  |  |  | FCCP / control | 1496 | 1104 |
|  |  |  |  | AMP / control | 3874 | 4365 |
|  |  |  |  | AMP / control | 3596 | 3586 |
| **CD8** | t-BHP / control  (CD28^+^ young donor) | 3893 | 6425 | CD28^-^ / CD28^+^  (young donor) | 6214 | 4689 |
|  | t-BHP / control  (CD28^+^ young donor) | 6845 | 6061 | CD28^-^ / CD28^+^  (young donor) | 5390 | 5506 |
|  | t-BHP / control  (CD28^+^ old donor) | 2651 | 3506 | CD28^-^ / CD28^+^  (old donor) | 3797 | 4986 |
|  | t-BHP / control  (CD28^+^ old donor) | 6055 | 4976 | CD28^-^ / CD28^+^  (old donor) | 3072 | 2808 |
|  | t-BHP / control  (CD28^-^ young donor) | 3171 | 4076 |  |  |  |
|  | t-BHP / control  (CD28^-^ old donor) | 4618 | 3672 |  |  |  |
|  | t-BHP / control  (CD28^-^ old donor) | 4950 | 3226 |  |  |  |
| **MSC** | 20% O_2_ / 3% O_2_  (young donor) | 4919 | 3744 | old donor /  young donor  (3% O_2_) | 5614 | 3864 |
|  | 20% O_2_ / 3% O_2_  (old donor) | 2230 | 1597 | old donor /  young donor  (20% O_2_) | 3887 | 2403 |

**Table S1**
