# Supplementary material for: Identification of evolutionarily conserved genetic regulators of cellular aging
Source: Aging Cell. 2010 Dec;9(6):1084–97. doi: 10.1111/j.1474-9726.2010.00637.x (PMC2997327; doi:10.1111/j.1474-9726.2010.00637.x)
Supplement: Supplementary file 5 [file acel0009-1084-SD5.pdf]

| Id | GenBank | UniGene | Description | Symbol | EG | upregulated in EG1 | downregulated in EG1 | upregulated in EG2 | downregulated in EG2 | EG1 p-value | EG1 q-value (Storey) | EG2 p-value | EG2 q-value (Storey) |
|---|---|---|---|---|---|---|---|---|---|---|---|---|---|
| 219282_s_at | NM_015930 | Hs.279746 | transient receptor potential cation channel, subfamily | TRPV2 | EG1 | 4 | 10 | 5 | 1 | 2,15E-11 | 0,0029338410 | 0,0147492140 | 0,111110101 |
| 1555996_s_at | AI332397 | Hs.478553 | eukaryotic translation initiation factor 4A, isoform 2 | EIF4A2 | EG1 | 10 | 3 | 6 | 2 | 6,10E-10 | 0,0029338410 | 0,0005786180 | 0,011359908 |
| 201468_s_at | NM_000903 | Hs.406515 | NAD(P)H dehydrogenase, quinone 1 | NQO1 | EG1 | 9 | 4 | 3 | 2 | 6,10E-10 | 0,0029338410 | 0,0540847830 | 0,274766231 |
| 1555989_at | AA890373 | Hs.19156 | dishevelled associated activator of morphogenesis 1 | DAAM1 | EG1 | 8 | 4 | 5 | 3 | 1,28E-08 | 0,0029338410 | 0,0005786180 | 0,011359908 |
| 218252_at | NM_018204 | Hs.444028 | cytoskeleton associated protein 2 | CKAP2 | EG1 | 11 | 1 | 4 | 4 | 1,28E-08 | 0,0029338410 | 0,0005786180 | 0,011359908 |
| 201123_s_at | NM_001970 | Hs.534314 | eukaryotic translation initiation factor 5A | EIF5A | EG1 | 11 | 1 | 4 | 2 | 1,28E-08 | 0,0029338410 | 0,0147492140 | 0,111110101 |
| 203028_s_at | NM_000101 | Hs.513803 | cytochrome b-245, alpha polypeptide | CYBA | EG1 | 3 | 9 | 5 | 1 | 1,28E-08 | 0,0029338410 | 0,0147492140 | 0,111110101 |
| 212366_at | BF223237 | Hs.485892 | zinc finger protein 292 | ZNF292 | EG1 | 8 | 4 | 3 | 3 | 1,28E-08 | 0,0029338410 | 0,0147492140 | 0,111110101 |
| 225191_at | AL565767 | Hs.501309 | cold inducible RNA binding protein | CIRBP | EG1 | 8 | 4 | 3 | 3 | 1,28E-08 | 0,0029338410 | 0,0147492140 | 0,111110101 |
| 225767_at | AI825833 | Hs.370699 | | LOC284801 | EG1 | 7 | 5 | 1 | 5 | 1,28E-08 | 0,0029338410 | 0,0147492140 | 0,111110101 |
| 228273_at | BG165011 | Hs.274448 | | FLJ11029 | EG1 | 5 | 7 | 2 | 4 | 1,28E-08 | 0,0029338410 | 0,0147492140 | 0,111110101 |
| 229390_at | AV734646 | Hs.381220 | | LOC441168 | EG1 | 5 | 7 | 3 | 3 | 1,28E-08 | 0,0029338410 | 0,0147492140 | 0,111110101 |
| 239577_at | AV699781 | | | | EG1 | 10 | 2 | 5 | 1 | 1,28E-08 | 0,0029338410 | 0,0147492140 | 0,111110101 |
| 235419_at | AW612461 | Hs.11169 | ERBB receptor feedback inhibitor 1 | ERRFI1 | EG1 | 7 | 5 | 3 | 1 | 1,28E-08 | 0,0029338410 | 0,1581335050 | 0,559565828 |
| 201310_s_at | NM_004772 | Hs.483067 | chromosome 5 open reading frame 13 | C5orf13 | EG1 | 4 | 7 | 5 | 3 | 2,08E-07 | 0,0029338410 | 0,0005786180 | 0,011359908 |
| 202589_at | NM_001071 | Hs.369762 | thymidylate synthetase | TYMS | EG1 | 4 | 7 | 2 | 6 | 2,08E-07 | 0,0029338410 | 0,0005786180 | 0,011359908 |
| 204127_at | BC000149 | Hs.115474 | replication factor C (activator 1) 3, 38kDa | RFC3 | EG1 | 10 | 1 | 3 | 5 | 2,08E-07 | 0,0029338410 | 0,0005786180 | 0,011359908 |
| 205193_at | NM_012323 | Hs.517617 | v-maf musculoaponeurotic fibrosarcoma oncogene ho | MAFF | EG1 | 8 | 3 | 5 | 3 | 2,08E-07 | 0,0029338410 | 0,0005786180 | 0,011359908 |
| 209211_at | AF132818 | Hs.508234 | Kruppel-like factor 5 (intestinal) | KLF5 | EG1 | 7 | 4 | 2 | 6 | 2,08E-07 | 0,0029338410 | 0,0005786180 | 0,011359908 |
| 219049_at | NM_018371 | Hs.387794 | | ChGn | EG1 | 2 | 9 | 2 | 6 | 2,08E-07 | 0,0029338410 | 0,0005786180 | 0,011359908 |
| 223255_at | AA642341 | Hs.509008 | KIAA1333 | KIAA1333 | EG1 | 11 | 0 | 4 | 4 | 2,08E-07 | 0,0029338410 | 0,0005786180 | 0,011359908 |
| 228974_at | AI816281 | Hs.20506 | zinc finger protein 677 | ZNF677 | EG1 | 8 | 3 | 5 | 3 | 2,08E-07 | 0,0029338410 | 0,0005786180 | 0,011359908 |
| AFFX-PheX-M | AFFX-PHEX-M | | | | EG1 | 5 | 6 | 5 | 3 | 2,08E-07 | 0,0029338410 | 0,0005786180 | 0,011359908 |
| 216237_s_at | AA807529 | Hs.517582 | MCM5 minichromosome maintenance deficient 5, cell | MCM5 | EG1 | 5 | 6 | 2 | 5 | 2,08E-07 | 0,0029338410 | 0,0032420520 | 0,038332332 |
| 224407_s_at | AF344882 | Hs.444247 | | MASK | EG1 | 10 | 1 | 4 | 3 | 2,08E-07 | 0,0029338410 | 0,0032420520 | 0,038332332 |
| 201792_at | NM_001129 | Hs.439463 | AE binding protein 1 | AEBP1 | EG1 | 3 | 8 | 2 | 4 | 2,08E-07 | 0,0029338410 | 0,0147492140 | 0,111110101 |
| 202581_at | NM_005346 | Hs.274402 | heat shock 70kDa protein 1B | HSPA1B | EG1 | 10 | 1 | 3 | 3 | 2,08E-07 | 0,0029338410 | 0,0147492140 | 0,111110101 |
| 202688_at | NM_003810 | Hs.478275 | tumor necrosis factor (ligand) superfamily, member 1 | TNFSF10 | EG1 | 2 | 9 | 1 | 5 | 2,08E-07 | 0,0029338410 | 0,0147492140 | 0,111110101 |
| 214132_at | BG232034 | Hs.271135 | ATP synthase, H+ transporting, mitochondrial F1 comp | ATP5C1 | EG1 | 8 | 3 | 5 | 1 | 2,08E-07 | 0,0029338410 | 0,0147492140 | 0,111110101 |
| 214820_at | AJ002572 | Hs.314338 | bromodomain and WD repeat domain containing 1 | BRWD1 | EG1 | 9 | 2 | 2 | 4 | 2,08E-07 | 0,0029338410 | 0,0147492140 | 0,111110101 |
| 226998_at | AL556909 | Hs.555985 | NMDA receptor regulated 1 | NARG1 | EG1 | 11 | 0 | 4 | 2 | 2,08E-07 | 0,0029338410 | 0,0147492140 | 0,111110101 |
| 228334_x_at | AI633734 | Hs.555989 | KIAA1712 | KIAA1712 | EG1 | 9 | 2 | 3 | 3 | 2,08E-07 | 0,0029338410 | 0,0147492140 | 0,111110101 |
| 229391_s_at | AV734646 | Hs.381220 | | LOC441168 | EG1 | 4 | 7 | 3 | 3 | 2,08E-07 | 0,0029338410 | 0,0147492140 | 0,111110101 |
| 235412_at | AI040887 | Hs.508738 | Rho guanine nucleotide exchange factor (GEF) 7 | ARHGEF7 | EG1 | 6 | 5 | 4 | 2 | 2,08E-07 | 0,0029338410 | 0,0147492140 | 0,111110101 |
| 236114_at | AI798118 | Hs.149261, H | runt-related transcription factor 1 (acute myeloid leuk | RUNX1 | EG1 | 0 | 11 | 6 | 0 | 2,08E-07 | 0,0029338410 | 0,0147492140 | 0,111110101 |
| AFFX-PheX-3_ | AFFX-PHEX-3 | | | | EG1 | 5 | 6 | 3 | 3 | 2,08E-07 | 0,0029338410 | 0,0147492140 | 0,111110101 |
| 201211_s_at | AF061337 | Hs.380774 | DEAD (Asp-Glu-Ala-Asp) box polypeptide 3, X-linked | DDX3X | EG1 | 9 | 2 | 3 | 2 | 2,08E-07 | 0,0029338410 | 0,0540847830 | 0,274766231 |
| 205092_x_at | NM_014950 | Hs.400802 | zinc finger and BTB domain containing 1 | ZBTB1 | EG1 | 8 | 3 | 2 | 3 | 2,08E-07 | 0,0029338410 | 0,0540847830 | 0,274766231 |
| 207838_x_at | NM_020524 | Hs.505806 | pre-B-cell leukemia transcription factor interacting pr | PBXIP1 | EG1 | 5 | 6 | 1 | 4 | 2,08E-07 | 0,0029338410 | 0,0540847830 | 0,274766231 |
| 209679_s_at | BC003379 | Hs.206501 | | LOC57228 | EG1 | 5 | 6 | 3 | 2 | 2,08E-07 | 0,0029338410 | 0,0540847830 | 0,274766231 |
| 221848_at | AL121845 | Hs.11900 | zinc finger, CCCH-type with G patch domain | ZGPAT | EG1 | 2 | 9 | 4 | 1 | 2,08E-07 | 0,0029338410 | 0,0540847830 | 0,274766231 |
| 226613_at | AI742029 | Hs.444950 | TBC1 domain family, member 10A | TBC1D10A | EG1 | 2 | 9 | 1 | 4 | 2,08E-07 | 0,0029338410 | 0,0540847830 | 0,274766231 |
| 228328_at | AI982758 | | | | EG1 | 9 | 2 | 2 | 3 | 2,08E-07 | 0,0029338410 | 0,0540847830 | 0,274766231 |

| | | | | | | | | | | | | | |
|---|---|---|---|---|---|---|---|---|---|---|---|---|---|
| 233014_at | AK022980 | Hs.283652 | isopentenyl-diphosphate delta isomerase 1 | IDI1 | EG1 | 8 | 3 | 3 | 2 | 2,08E-07 | 0,002933841 | 0,054084783 | 0,274766231 |
| 231975_s_at | AK002183 | Hs.419195 | | FLJ35954 | EG1 | 11 | 0 | 3 | 1 | 2,08E-07 | 0,002933841 | 0,158133505 | 0,559565828 |
| 241242_at | BE503118 | Hs.372003 | chromosome 9 open reading frame 10 | C9orf10 | EG1 | 3 | 8 | 4 | 0 | 2,08E-07 | 0,002933841 | 0,158133505 | 0,559565828 |
| 207247_s_at | NM_003411 | Hs.522845 | zinc finger protein, Y-linked | ZFY | EG1 | 10 | 1 | 3 | 0 | 2,08E-07 | 0,002933841 | 0,363634923 | 0,911710613 |
| 211538_s_at | U56725 | Hs.432648 | heat shock 70kDa protein 2 | HSPA2 | EG1 | 7 | 4 | 2 | 1 | 2,08E-07 | 0,002933841 | 0,363634923 | 0,911710613 |
| 221135_s_at | NM_014065 | Hs.100878 | | HT001 | EG1 | 9 | 2 | 2 | 0 | 2,08E-07 | 0,002933841 | 0,649016171 | 0,911710613 |
| 1552701_a_at | NM_052889 | Hs.348365 | | COP1 | EG1 | 1 | 9 | 7 | 1 | 2,66E-06 | 0,002933841 | 0,000578618 | 0,011359908 |
| 1554609_at | BC019340 | Hs.292791 | | MGC12965 | EG1 | 3 | 7 | 1 | 7 | 2,66E-06 | 0,002933841 | 0,000578618 | 0,011359908 |
| 204092_s_at | NM_003600 | Hs.250822 | serine/threonine kinase 6 | STK6 | EG1 | 8 | 2 | 3 | 5 | 2,66E-06 | 0,002933841 | 0,000578618 | 0,011359908 |
| 204244_s_at | NM_006716 | Hs.489037 | | ASK | EG1 | 10 | 0 | 3 | 5 | 2,66E-06 | 0,002933841 | 0,000578618 | 0,011359908 |
| 204709_s_at | NM_004856 | Hs.270845 | kinesin family member 23 | KIF23 | EG1 | 7 | 3 | 3 | 5 | 2,66E-06 | 0,002933841 | 0,000578618 | 0,011359908 |
| 207165_at | NM_012485 | Hs.72550 | hyaluronan-mediated motility receptor (RHAMM) | HMMR | EG1 | 7 | 3 | 3 | 5 | 2,66E-06 | 0,002933841 | 0,000578618 | 0,011359908 |
| 211368_s_at | U13700 | Hs.2490 | caspase 1, apoptosis-related cysteine peptidase (inter | CASP1 | EG1 | 2 | 8 | 6 | 2 | 2,66E-06 | 0,002933841 | 0,000578618 | 0,011359908 |
| 212977_at | AI817041 | Hs.471751 | chemokine orphan receptor 1 | CMKOR1 | EG1 | 0 | 10 | 3 | 5 | 2,66E-06 | 0,002933841 | 0,000578618 | 0,011359908 |
| 217924_at | AL523965 | Hs.485162 | chromosome 6 open reading frame 106 | C6orf106 | EG1 | 7 | 3 | 8 | 0 | 2,66E-06 | 0,002933841 | 0,000578618 | 0,011359908 |
| 222040_at | AI144007 | Hs.210850, H | heterogeneous nuclear ribonucleoprotein A1 | HNRPA1 | EG1 | 9 | 1 | 4 | 4 | 2,66E-06 | 0,002933841 | 0,000578618 | 0,011359908 |
| 222088_s_at | AA778684 | Hs.210227 | solute carrier family 2 (facilitated glucose transporter) | SLC2A14 | EG1 | 5 | 5 | 4 | 4 | 2,66E-06 | 0,002933841 | 0,000578618 | 0,011359908 |
| 225902_at | AW194716 | Hs.470544 | peptidyl-prolyl isomerase G (cyclophilin G) | PPIG | EG1 | 9 | 1 | 1 | 7 | 2,66E-06 | 0,002933841 | 0,000578618 | 0,011359908 |
| 228751_at | AW975057 | Hs.406557 | CDC-like kinase 4 | CLK4 | EG1 | 7 | 3 | 5 | 3 | 2,66E-06 | 0,002933841 | 0,000578618 | 0,011359908 |
| 230461_s_at | AI860326 | Hs.515016 | melanoma associated antigen (mutated) 1 | MUM1 | EG1 | 7 | 3 | 4 | 4 | 2,66E-06 | 0,002933841 | 0,000578618 | 0,011359908 |
| 232797_at | AU144005 | Hs.436873 | integrin, alpha V (vitronectin receptor, alpha polypept | ITGAV | EG1 | 4 | 6 | 8 | 0 | 2,66E-06 | 0,002933841 | 0,000578618 | 0,011359908 |
| 233899_x_at | AK024296 | Hs.205742 | zinc finger and BTB domain containing 10 | ZBTB10 | EG1 | 8 | 2 | 3 | 5 | 2,66E-06 | 0,002933841 | 0,000578618 | 0,011359908 |
| 235152_at | AW293849 | | | | EG1 | 1 | 9 | 7 | 1 | 2,66E-06 | 0,002933841 | 0,000578618 | 0,011359908 |
| 235167_at | BE972419 | Hs.528462, Hs.531664, Hs.547315, Hs.556385 | | DKFZp547E087 | EG1 | 8 | 2 | 5 | 3 | 2,66E-06 | 0,002933841 | 0,000578618 | 0,011359908 |
| 236402_at | AW184034 | Hs.490366 | v-raf murine sarcoma viral oncogene homolog B1 | BRAF | EG1 | 6 | 4 | 4 | 4 | 2,66E-06 | 0,002933841 | 0,000578618 | 0,011359908 |
| 236752_at | AA913146 | Hs.407580 | plakophilin 4 | PKP4 | EG1 | 6 | 4 | 8 | 0 | 2,66E-06 | 0,002933841 | 0,000578618 | 0,011359908 |
| 238712_at | BF801735 | | | | EG1 | 1 | 9 | 6 | 2 | 2,66E-06 | 0,002933841 | 0,000578618 | 0,011359908 |
| 243648_at | AA280627 | Hs.532399 | zinc finger CCCH-type containing 11A | ZC3H11A | EG1 | 3 | 7 | 5 | 3 | 2,66E-06 | 0,002933841 | 0,000578618 | 0,011359908 |
| AFFX-r2-Bs-th | AFFX-R2-BS-THR-3 | | | | EG1 | 5 | 5 | 6 | 2 | 2,66E-06 | 0,002933841 | 0,000578618 | 0,011359908 |
| 201774_s_at | AK022511 | Hs.5719 | | CNAP1 | EG1 | 5 | 5 | 2 | 5 | 2,66E-06 | 0,002933841 | 0,003242052 | 0,038332332 |
| 202338_at | NM_003258 | Hs.515122 | thymidine kinase 1, soluble | TK1 | EG1 | 5 | 5 | 2 | 5 | 2,66E-06 | 0,002933841 | 0,003242052 | 0,038332332 |
| 203109_at | NM_003969 | Hs.406068 | ubiquitin-conjugating enzyme E2M (UBC12 homolog, y | UBE2M | EG1 | 7 | 3 | 4 | 3 | 2,66E-06 | 0,002933841 | 0,003242052 | 0,038332332 |
| 203422_at | NM_002691 | Hs.279413 | polymerase (DNA directed), delta 1, catalytic subunit | POLD1 | EG1 | 3 | 7 | 2 | 5 | 2,66E-06 | 0,002933841 | 0,003242052 | 0,038332332 |
| 206917_at | NM_006572 | Hs.515018 | guanine nucleotide binding protein (G protein), alpha | GNA13 | EG1 | 9 | 1 | 7 | 0 | 2,66E-06 | 0,002933841 | 0,003242052 | 0,038332332 |
| 209093_s_at | K02920 | Hs.282997, H | glucosidase, beta, acid (includes glucosylceramidase) | GBA | EG1 | 2 | 8 | 4 | 3 | 2,66E-06 | 0,002933841 | 0,003242052 | 0,038332332 |
| 210006_at | BC002571 | Hs.534400 | abhydrolase domain containing 14A | ABHD14A | EG1 | 2 | 8 | 4 | 3 | 2,66E-06 | 0,002933841 | 0,003242052 | 0,038332332 |
| 211366_x_at | U13698 | Hs.2490 | caspase 1, apoptosis-related cysteine peptidase (inter | CASP1 | EG1 | 2 | 8 | 6 | 1 | 2,66E-06 | 0,002933841 | 0,003242052 | 0,038332332 |
| 212187_x_at | NM_000954 | Hs.446429 | prostaglandin D2 synthase 21kDa (brain) | PTGDS | EG1 | 6 | 4 | 5 | 2 | 2,66E-06 | 0,002933841 | 0,003242052 | 0,038332332 |
| 213023_at | NM_007124 | Hs.133135 | utrophin (homologous to dystrophin) | UTRN | EG1 | 8 | 2 | 4 | 3 | 2,66E-06 | 0,002933841 | 0,003242052 | 0,038332332 |
| 214877_at | BE794663 | Hs.484844 | CDK5 regulatory subunit associated protein 1-like 1 | CDKAL1 | EG1 | 5 | 5 | 4 | 3 | 2,66E-06 | 0,002933841 | 0,003242052 | 0,038332332 |
| 219004_s_at | NM_018944 | Hs.190518 | chromosome 21 open reading frame 45 | C21orf45 | EG1 | 5 | 5 | 2 | 5 | 2,66E-06 | 0,002933841 | 0,003242052 | 0,038332332 |
| 219334_s_at | NM_022837 | Hs.118183 | | FLJ22833 | EG1 | 5 | 5 | 3 | 4 | 2,66E-06 | 0,002933841 | 0,003242052 | 0,038332332 |
| 221843_s_at | AA195017 | Hs.288274 | | KIAA1609 | EG1 | 2 | 8 | 2 | 5 | 2,66E-06 | 0,002933841 | 0,003242052 | 0,038332332 |
| 225762_x_at | AL531683 | Hs.370699 | | LOC284801 | EG1 | 7 | 3 | 2 | 5 | 2,66E-06 | 0,002933841 | 0,003242052 | 0,038332332 |
| 225786_at | AI440495 | Hs.411490, H | family with sequence similarity 36, member A | FAM36A | EG1 | 8 | 2 | 2 | 5 | 2,66E-06 | 0,002933841 | 0,003242052 | 0,038332332 |

| | | | | | | | | | | | | | |
|---|---|---|---|---|---|---|---|---|---|---|---|---|---|
| 227198_at | AW085505 | Hs.444414 | AF4/FMR2 family, member 3 | AFF3 | EG1 | 5 | 5 | 1 | 6 | 2,66E-06 | 0,002933841 | 0,003242052 | 0,038332332 |
| 228561_at | BF510563 | Hs.493361 | CDC37 cell division cycle 37 homolog (S. cerevisiae)-li | CDC37L1 | EG1 | 9 | 1 | 4 | 3 | 2,66E-06 | 0,002933841 | 0,003242052 | 0,038332332 |
| 231069_at | AI768895 | | | | EG1 | 6 | 4 | 5 | 2 | 2,66E-06 | 0,002933841 | 0,003242052 | 0,038332332 |
| 231152_at | AW452971 | | | | EG1 | 9 | 1 | 3 | 4 | 2,66E-06 | 0,002933841 | 0,003242052 | 0,038332332 |
| 242868_at | T70087 | Hs.468410 | endothelial PAS domain protein 1 | EPAS1 | EG1 | 6 | 4 | 6 | 1 | 2,66E-06 | 0,002933841 | 0,003242052 | 0,038332332 |
| 243829_at | AW613053 | Hs.490366 | v-raf murine sarcoma viral oncogene homolog B1 | BRAF | EG1 | 9 | 1 | 6 | 1 | 2,66E-06 | 0,002933841 | 0,003242052 | 0,038332332 |
| 36711_at | AL021977 | Hs.517617 | v-maf musculoaponeurotic fibrosarcoma oncogene ho | MAFF | EG1 | 7 | 3 | 4 | 3 | 2,66E-06 | 0,002933841 | 0,003242052 | 0,038332332 |
| AFFX-r2-Bs-ph | AFFX-R2-BS-PHE-M | | | | EG1 | 4 | 6 | 3 | 4 | 2,66E-06 | 0,002933841 | 0,003242052 | 0,038332332 |
| 1552302_at | NM_145041 | Hs.446690 | | MGC20235 | EG1 | 3 | 7 | 3 | 3 | 2,66E-06 | 0,002933841 | 0,014749214 | 0,111110101 |
| 1552703_s_at | NM_052889 | Hs.348365 | | COP1 | EG1 | 1 | 9 | 4 | 2 | 2,66E-06 | 0,002933841 | 0,014749214 | 0,111110101 |
| 1555892_s_at | BF476087 | Hs.131417, Hs.193725 | | LOC253039 | EG1 | 6 | 4 | 2 | 4 | 2,66E-06 | 0,002933841 | 0,014749214 | 0,111110101 |
| 202107_s_at | NM_004526 | Hs.477481 | MCM2 minichromosome maintenance deficient 2, mit | MCM2 | EG1 | 4 | 6 | 2 | 4 | 2,66E-06 | 0,002933841 | 0,014749214 | 0,111110101 |
| 202295_s_at | NM_004390 | Hs.148641 | cathepsin H | CTSH | EG1 | 2 | 8 | 2 | 4 | 2,66E-06 | 0,002933841 | 0,014749214 | 0,111110101 |
| 206548_at | NM_024880 | Hs.501080 | | FLJ23556 | EG1 | 5 | 5 | 5 | 1 | 2,66E-06 | 0,002933841 | 0,014749214 | 0,111110101 |
| 207030_s_at | NM_001321 | Hs.530904 | cysteine and glycine-rich protein 2 | CSRP2 | EG1 | 4 | 6 | 3 | 3 | 2,66E-06 | 0,002933841 | 0,014749214 | 0,111110101 |
| 208881_x_at | BC005247 | Hs.283652 | isopentenyl-diphosphate delta isomerase 1 | IDI1 | EG1 | 9 | 1 | 6 | 0 | 2,66E-06 | 0,002933841 | 0,014749214 | 0,111110101 |
| 210589_s_at | D13287 | Hs.282997, H | glucosidase, beta, acid (includes glucosylceramidase) | GBA | EG1 | 1 | 9 | 4 | 2 | 2,66E-06 | 0,002933841 | 0,014749214 | 0,111110101 |
| 210845_s_at | U08839 | Hs.466871 | plasminogen activator, urokinase receptor | PLAUR | EG1 | 7 | 3 | 4 | 2 | 2,66E-06 | 0,002933841 | 0,014749214 | 0,111110101 |
| 211676_s_at | AF056979 | Hs.520414 | interferon gamma receptor 1 | IFNGR1 | EG1 | 9 | 1 | 5 | 1 | 2,66E-06 | 0,002933841 | 0,014749214 | 0,111110101 |
| 213302_at | AL044326 | Hs.88139 | phosphoribosylformylglycinamidine synthase (FGAR a | PFAS | EG1 | 5 | 5 | 3 | 3 | 2,66E-06 | 0,002933841 | 0,014749214 | 0,111110101 |
| 219854_at | NM_021030 | Hs.466261 | zinc finger protein 14 (KOX 6) | ZNF14 | EG1 | 7 | 3 | 4 | 2 | 2,66E-06 | 0,002933841 | 0,014749214 | 0,111110101 |
| 224558_s_at | AI446756 | Hs.187199, H | metastasis associated lung adenocarcinoma transcript | MALAT1 | EG1 | 8 | 2 | 5 | 1 | 2,66E-06 | 0,002933841 | 0,014749214 | 0,111110101 |
| 224973_at | AL078599 | Hs.10784 | family with sequence similarity 46, member A | FAM46A | EG1 | 7 | 3 | 5 | 1 | 2,66E-06 | 0,002933841 | 0,014749214 | 0,111110101 |
| 225227_at | AW294869 | | | | EG1 | 8 | 2 | 3 | 3 | 2,66E-06 | 0,002933841 | 0,014749214 | 0,111110101 |
| 227265_at | AW135176 | | | | EG1 | 5 | 5 | 5 | 1 | 2,66E-06 | 0,002933841 | 0,014749214 | 0,111110101 |
| 234995_at | AA668779 | Hs.477144 | | LOC152185 | EG1 | 4 | 6 | 3 | 3 | 2,66E-06 | 0,002933841 | 0,014749214 | 0,111110101 |
| 240572_s_at | BF436632 | Hs.396637 | | LOC374443 | EG1 | 3 | 7 | 4 | 2 | 2,66E-06 | 0,002933841 | 0,014749214 | 0,111110101 |
| 1553984_s_at | AF258562 | Hs.471873 | deoxythymidylate kinase (thymidylate kinase) | DTYMK | EG1 | 4 | 6 | 2 | 3 | 2,66E-06 | 0,002933841 | 0,054084783 | 0,274766231 |
| 1562836_at | AK021715 | Hs.408461 | DEAD (Asp-Glu-Ala-Asp) box polypeptide 6 | DDX6 | EG1 | 8 | 2 | 2 | 3 | 2,66E-06 | 0,002933841 | 0,054084783 | 0,274766231 |
| 1563469_at | AL832681 | Hs.535297 | AT rich interactive domain 5B (MRF1-like) | ARID5B | EG1 | 2 | 8 | 2 | 3 | 2,66E-06 | 0,002933841 | 0,054084783 | 0,274766231 |
| 202687_s_at | U57059 | Hs.478275 | tumor necrosis factor (ligand) superfamily, member 1 | TNFSF10 | EG1 | 2 | 8 | 1 | 4 | 2,66E-06 | 0,002933841 | 0,054084783 | 0,274766231 |
| 204170_s_at | NM_001827 | Hs.83758 | CDC28 protein kinase regulatory subunit 2 | CKS2 | EG1 | 9 | 1 | 2 | 3 | 2,66E-06 | 0,002933841 | 0,054084783 | 0,274766231 |
| 212514_x_at | R60068 | Hs.380774 | DEAD (Asp-Glu-Ala-Asp) box polypeptide 3, X-linked | DDX3X | EG1 | 10 | 0 | 3 | 2 | 2,66E-06 | 0,002933841 | 0,054084783 | 0,274766231 |
| 226444_at | AI700476 | Hs.413434 | solute carrier family 39 (zinc transporter), member 10 | SLC39A10 | EG1 | 8 | 2 | 5 | 0 | 2,66E-06 | 0,002933841 | 0,054084783 | 0,274766231 |
| 228742_at | AA745978 | | | | EG1 | 2 | 8 | 1 | 4 | 2,66E-06 | 0,002933841 | 0,054084783 | 0,274766231 |
| 229735_s_at | W15435 | Hs.523442 | NIPA-like domain containing 3 | NPAL3 | EG1 | 4 | 6 | 1 | 4 | 2,66E-06 | 0,002933841 | 0,054084783 | 0,274766231 |
| 230036_at | BE669858 | Hs.489118 | sterile alpha motif domain containing 9-like | SAMD9L | EG1 | 3 | 7 | 1 | 4 | 2,66E-06 | 0,002933841 | 0,054084783 | 0,274766231 |
| 231269_at | AU153330 | Hs.486031 | | DJ467N11.1 | EG1 | 9 | 1 | 4 | 1 | 2,66E-06 | 0,002933841 | 0,054084783 | 0,274766231 |
| 232978_at | AF339810 | Hs.125715 | muscleblind-like 2 (Drosophila) | MBNL2 | EG1 | 7 | 3 | 5 | 0 | 2,66E-06 | 0,002933841 | 0,054084783 | 0,274766231 |
| 233849_s_at | AK023014 | Hs.525287 | Rho GTPase activating protein 5 | ARHGAP5 | EG1 | 8 | 2 | 0 | 5 | 2,66E-06 | 0,002933841 | 0,054084783 | 0,274766231 |
| 236974_at | AA808018 | Hs.518827 | cyclin I | CCNI | EG1 | 8 | 2 | 5 | 0 | 2,66E-06 | 0,002933841 | 0,054084783 | 0,274766231 |
| 241627_x_at | AI640434 | Hs.35125 | | FLJ10357 | EG1 | 6 | 4 | 3 | 2 | 2,66E-06 | 0,002933841 | 0,054084783 | 0,274766231 |
| 1557813_at | BF724621 | Hs.102735 | single-stranded DNA binding protein 2 | SSBP2 | EG1 | 8 | 2 | 3 | 1 | 2,66E-06 | 0,002933841 | 0,158133505 | 0,559565828 |
| 205449_at | NM_013299 | Hs.23642 | SAC3 domain containing 1 | SAC3D1 | EG1 | 4 | 6 | 1 | 3 | 2,66E-06 | 0,002933841 | 0,158133505 | 0,559565828 |
| 214787_at | BE268538 | Hs.513817 | c-myc promoter binding protein | MYCPBP | EG1 | 10 | 0 | 1 | 3 | 2,66E-06 | 0,002933841 | 0,158133505 | 0,559565828 |

| | | | | | | | | | | | | | |
|---|---|---|---|---|---|---|---|---|---|---|---|---|---|
| 221428_s_at | NM_030921 | Hs.438970 | transducin (beta)-like 1X-linked receptor 1 | TBL1XR1 | EG1 | 7 | 3 | 1 | 3 | 2,66E-06 | 0,0029338410 | 0,1581335050 | 0,559565828 |
| 221763_at | AI694023 | Hs.413416, H | jumonji domain containing 1C | JMJD1C | EG1 | 9 | 1 | 2 | 2 | 2,66E-06 | 0,0029338410 | 0,1581335050 | 0,559565828 |
| 244811_at | AI561173 | Hs.511817 | pleckstrin homology domain interacting protein | PHIP | EG1 | 9 | 1 | 2 | 2 | 2,66E-06 | 0,0029338410 | 0,1581335050 | 0,559565828 |
| 1557522_x_at | AA653638 | Hs.472651 | bladder cancer associated protein | BLCAP | EG1 | 7 | 3 | 1 | 2 | 2,66E-06 | 0,0029338410 | 0,3636349230 | 0,911710613 |
| 204868_at | NM_001545 | Hs.407955 | immature colon carcinoma transcript 1 | ICT1 | EG1 | 3 | 7 | 1 | 2 | 2,66E-06 | 0,0029338410 | 0,3636349230 | 0,911710613 |
| 211752_s_at | BC005954 | Hs.211914 | NADH dehydrogenase (ubiquinone) Fe-S protein 7, 20 | NDUFS7 | EG1 | 2 | 8 | 1 | 2 | 2,66E-06 | 0,0029338410 | 0,3636349230 | 0,911710613 |
| 212037_at | BF508848 | Hs.409965 | pinin, desmosome associated protein | PNN | EG1 | 10 | 0 | 1 | 2 | 2,66E-06 | 0,0029338410 | 0,3636349230 | 0,911710613 |
| 218166_s_at | NM_016578 | Hs.420229 | hepatitis B virus x associated protein | HBXAP | EG1 | 9 | 1 | 2 | 1 | 2,66E-06 | 0,0029338410 | 0,3636349230 | 0,911710613 |
| 219470_x_at | NM_019084 | Hs.281348 | cyclin J | CCNJ | EG1 | 9 | 1 | 3 | 0 | 2,66E-06 | 0,0029338410 | 0,3636349230 | 0,911710613 |
| 228758_at | AW264036 | Hs.478589 | | LOC389185 | EG1 | 7 | 3 | 3 | 0 | 2,66E-06 | 0,0029338410 | 0,3636349230 | 0,911710613 |
| 232382_s_at | BE150929 | Hs.308480 | | LOC115294 | EG1 | 4 | 6 | 3 | 0 | 2,66E-06 | 0,0029338410 | 0,3636349230 | 0,911710613 |
| 234411_x_at | U94903 | Hs.502328 | CD44 antigen (homing function and Indian blood grou | CD44 | EG1 | 9 | 1 | 2 | 0 | 2,66E-06 | 0,0029338410 | 0,6490161710 | 0,911710613 |
| 238465_at | AI265933 | Hs.85950 | | MGC33648 | EG1 | 5 | 5 | 1 | 1 | 2,66E-06 | 0,0029338410 | 0,6490161710 | 0,911710613 |
| 239493_at | AI984074 | Hs.421257 | ribosomal protein L7 | RPL7 | EG1 | 9 | 1 | 1 | 1 | 2,66E-06 | 0,0029338410 | 0,6490161710 | 0,911710613 |
| 234881_at | L33988 | | | | EG1 | 3 | 7 | 1 | 0 | 2,66E-06 | 0,0029338410 | 0,8976818490 | 0,911710613 |
| 238456_at | AI567893 | | | | EG1 | 9 | 1 | 0 | 1 | 2,66E-06 | 0,0029338410 | 0,8976818490 | 0,911710613 |
| 1558401_at | AW953994 | | | | EG1 | 5 | 4 | 4 | 4 | 2,72E-05 | 0,0029338410 | 0,0005786180 | 0,011359908 |
| 1569157_s_at | BC037359 | Hs.546512 | | LOC162993 | EG1 | 2 | 7 | 2 | 6 | 2,72E-05 | 0,0029338410 | 0,0005786180 | 0,011359908 |
| 201136_at | NM_002668 | Hs.77422 | proteolipid protein 2 (colonic epithelium-enriched) | PLP2 | EG1 | 2 | 7 | 0 | 8 | 2,72E-05 | 0,0029338410 | 0,0005786180 | 0,011359908 |
| 201294_s_at | N24643 | Hs.446017 | WD repeat and SOCS box-containing 1 | WSB1 | EG1 | 5 | 4 | 4 | 4 | 2,72E-05 | 0,0029338410 | 0,0005786180 | 0,011359908 |
| 203022_at | NM_006397 | Hs.532851 | ribonuclease H2, large subunit | RNASEH2A | EG1 | 4 | 5 | 4 | 4 | 2,72E-05 | 0,0029338410 | 0,0005786180 | 0,011359908 |
| 206632_s_at | NM_004900 | Hs.226307 | apolipoprotein B mRNA editing enzyme, catalytic poly | APOBEC3B | EG1 | 5 | 4 | 2 | 6 | 2,72E-05 | 0,0029338410 | 0,0005786180 | 0,011359908 |
| 208116_s_at | NM_005907 | Hs.102788 | mannosidase, alpha, class 1A, member 1 | MAN1A1 | EG1 | 7 | 2 | 5 | 3 | 2,72E-05 | 0,0029338410 | 0,0005786180 | 0,011359908 |
| 208733_at | AW301641 | Hs.369017 | RAB2, member RAS oncogene family | RAB2 | EG1 | 4 | 5 | 4 | 4 | 2,72E-05 | 0,0029338410 | 0,0005786180 | 0,011359908 |
| 209164_s_at | BC002976 | Hs.355264 | cytochrome b-561 | CYB561 | EG1 | 2 | 7 | 3 | 5 | 2,72E-05 | 0,0029338410 | 0,0005786180 | 0,011359908 |
| 210172_at | D26121 | Hs.502829 | splicing factor 1 | SF1 | EG1 | 4 | 5 | 4 | 4 | 2,72E-05 | 0,0029338410 | 0,0005786180 | 0,011359908 |
| 212583_at | AB011132 | Hs.510958 | aquarius homolog (mouse) | AQR | EG1 | 4 | 5 | 7 | 1 | 2,72E-05 | 0,0029338410 | 0,0005786180 | 0,011359908 |
| 212847_at | AL036840 | Hs.269099 | far upstream element (FUSE) binding protein 1 | FUBP1 | EG1 | 8 | 1 | 5 | 3 | 2,72E-05 | 0,0029338410 | 0,0005786180 | 0,011359908 |
| 212907_at | AI972416 | Hs.519469 | solute carrier family 30 (zinc transporter), member 1 | SLC30A1 | EG1 | 9 | 0 | 8 | 0 | 2,72E-05 | 0,0029338410 | 0,0005786180 | 0,011359908 |
| 213182_x_at | R78668 | Hs.106070 | cyclin-dependent kinase inhibitor 1C (p57, Kip2) | CDKN1C | EG1 | 3 | 6 | 4 | 4 | 2,72E-05 | 0,0029338410 | 0,0005786180 | 0,011359908 |
| 219215_s_at | NM_017767 | Hs.521934 | solute carrier family 39 (zinc transporter), member 4 | SLC39A4 | EG1 | 2 | 7 | 4 | 4 | 2,72E-05 | 0,0029338410 | 0,0005786180 | 0,011359908 |
| 221555_x_at | AU145941 | Hs.40582 | CDC14 cell division cycle 14 homolog B (S. cerevisiae) | CDC14B | EG1 | 3 | 6 | 4 | 4 | 2,72E-05 | 0,0029338410 | 0,0005786180 | 0,011359908 |
| 222316_at | AW973253 | Hs.292689 | | VDP | EG1 | 7 | 2 | 6 | 2 | 2,72E-05 | 0,0029338410 | 0,0005786180 | 0,011359908 |
| 222662_at | W60806 | Hs.458513 | | LOC286044 | EG1 | 6 | 3 | 6 | 2 | 2,72E-05 | 0,0029338410 | 0,0005786180 | 0,011359908 |
| 223103_at | AF151810 | Hs.188606 | START domain containing 10 | STARD10 | EG1 | 3 | 6 | 0 | 8 | 2,72E-05 | 0,0029338410 | 0,0005786180 | 0,011359908 |
| 225834_at | AL135396 | Hs.534958 | | LOC440687 | EG1 | 4 | 5 | 3 | 5 | 2,72E-05 | 0,0029338410 | 0,0005786180 | 0,011359908 |
| 226682_at | AW006185 | Hs.512943 | | LOC283666 | EG1 | 4 | 5 | 5 | 3 | 2,72E-05 | 0,0029338410 | 0,0005786180 | 0,011359908 |
| 228153_at | AI953847 | Hs.148741 | IBR domain containing 2 | IBRDC2 | EG1 | 4 | 5 | 5 | 3 | 2,72E-05 | 0,0029338410 | 0,0005786180 | 0,011359908 |
| 229256_at | AV724329 | Hs.26612 | phosphoglucomutase 2-like 1 | PGM2L1 | EG1 | 6 | 3 | 3 | 5 | 2,72E-05 | 0,0029338410 | 0,0005786180 | 0,011359908 |
| 232573_at | AL389956 | Hs.403933 | F-box protein 32 | FBXO32 | EG1 | 7 | 2 | 6 | 2 | 2,72E-05 | 0,0029338410 | 0,0005786180 | 0,011359908 |
| 235440_at | BE780878 | Hs.268668 | | FLJ39441 | EG1 | 8 | 1 | 6 | 2 | 2,72E-05 | 0,0029338410 | 0,0005786180 | 0,011359908 |
| 235592_at | AW960145 | Hs.192221, H | elongation factor, RNA polymerase II, 2 | ELL2 | EG1 | 6 | 3 | 4 | 4 | 2,72E-05 | 0,0029338410 | 0,0005786180 | 0,011359908 |
| 238909_at | BF126155 | Hs.143873 | S100 calcium binding protein A10 (annexin II ligand, ca | S100A10 | EG1 | 1 | 8 | 6 | 2 | 2,72E-05 | 0,0029338410 | 0,0005786180 | 0,011359908 |
| 242691_at | AA829017 | | | | EG1 | 4 | 5 | 3 | 5 | 2,72E-05 | 0,0029338410 | 0,0005786180 | 0,011359908 |
| 244353_s_at | AI675682 | Hs.486508 | solute carrier family 2 (facilitated glucose transporter) | SLC2A12 | EG1 | 4 | 5 | 1 | 7 | 2,72E-05 | 0,0029338410 | 0,0005786180 | 0,011359908 |

| | | | | | | | | | | | | | |
|---|---|---|---|---|---|---|---|---|---|---|---|---|---|
| AFFX-r2-Bs-ph | AFFX-R2-BS-PHE-3 | | | | EG1 | 5 | 4 | 5 | 3 | 2,72E-05 | 0,002933841 | 0,000578618 | 0,011359908 |
| 1558102_at | AK055438 | Hs.513094 | transmembrane 6 superfamily member 1 | TM6SF1 | EG1 | 5 | 4 | 5 | 2 | 2,72E-05 | 0,002933841 | 0,003242052 | 0,038332332 |
| 1566901_at | AL832409 | Hs.373550 | TGFB-induced factor (TALE family homeobox) | TGIF | EG1 | 8 | 1 | 7 | 0 | 2,72E-05 | 0,002933841 | 0,003242052 | 0,038332332 |
| 201626_at | BG292233 | Hs.520819 | insulin induced gene 1 | INSIG1 | EG1 | 7 | 2 | 4 | 3 | 2,72E-05 | 0,002933841 | 0,003242052 | 0,038332332 |
| 201627_s_at | NM_005542 | Hs.520819 | insulin induced gene 1 | INSIG1 | EG1 | 7 | 2 | 4 | 3 | 2,72E-05 | 0,002933841 | 0,003242052 | 0,038332332 |
| 201702_s_at | AI492873 | Hs.106019 | protein phosphatase 1, regulatory subunit 10 | PPP1R10 | EG1 | 4 | 5 | 2 | 5 | 2,72E-05 | 0,002933841 | 0,003242052 | 0,038332332 |
| 202708_s_at | NM_003528 | Hs.2178 | histone 2, H2be | HIST2H2BE | EG1 | 8 | 1 | 4 | 3 | 2,72E-05 | 0,002933841 | 0,003242052 | 0,038332332 |
| 202869_at | NM_016816 | Hs.524760 | 2',5'-oligoadenylate synthetase 1, 40/46kDa | OAS1 | EG1 | 3 | 6 | 6 | 1 | 2,72E-05 | 0,002933841 | 0,003242052 | 0,038332332 |
| 202979_s_at | NM_021212 | Hs.535319 | | ZF | EG1 | 8 | 1 | 5 | 2 | 2,72E-05 | 0,002933841 | 0,003242052 | 0,038332332 |
| 203828_s_at | NM_004221 | Hs.943 | interleukin 32 | IL32 | EG1 | 2 | 7 | 1 | 6 | 2,72E-05 | 0,002933841 | 0,003242052 | 0,038332332 |
| 204502_at | NM_015474 | Hs.472630 | SAM domain and HD domain 1 | SAMHD1 | EG1 | 5 | 4 | 2 | 5 | 2,72E-05 | 0,002933841 | 0,003242052 | 0,038332332 |
| 205681_at | NM_004049 | Hs.227817 | BCL2-related protein A1 | BCL2A1 | EG1 | 5 | 4 | 4 | 3 | 2,72E-05 | 0,002933841 | 0,003242052 | 0,038332332 |
| 205798_at | NM_002185 | Hs.362807 | interleukin 7 receptor | IL7R | EG1 | 4 | 5 | 2 | 5 | 2,72E-05 | 0,002933841 | 0,003242052 | 0,038332332 |
| 208859_s_at | AI650257 | Hs.533526, H | alpha thalassemia/mental retardation syndrome X-lin | ATRX | EG1 | 2 | 7 | 5 | 2 | 2,72E-05 | 0,002933841 | 0,003242052 | 0,038332332 |
| 209417_s_at | BC001356 | Hs.50842 | interferon-induced protein 35 | IFI35 | EG1 | 2 | 7 | 3 | 4 | 2,72E-05 | 0,002933841 | 0,003242052 | 0,038332332 |
| 209512_at | BC004331 | Hs.59486 | hydroxysteroid dehydrogenase like 2 | HSDL2 | EG1 | 5 | 4 | 4 | 3 | 2,72E-05 | 0,002933841 | 0,003242052 | 0,038332332 |
| 209681_at | AF153330 | Hs.30246 | solute carrier family 19 (thiamine transporter), memb | SLC19A2 | EG1 | 9 | 0 | 4 | 3 | 2,72E-05 | 0,002933841 | 0,003242052 | 0,038332332 |
| 210778_s_at | BC002713 | Hs.102402 | MAX dimerization protein 4 | MXD4 | EG1 | 3 | 6 | 5 | 2 | 2,72E-05 | 0,002933841 | 0,003242052 | 0,038332332 |
| 212944_at | AK024896 | Hs.302742 | mitochondrial ribosomal protein S6 | MRPS6 | EG1 | 5 | 4 | 7 | 0 | 2,72E-05 | 0,002933841 | 0,003242052 | 0,038332332 |
| 213372_at | AW173157 | Hs.556026 | progestin and adipoQ receptor family member III | PAQR3 | EG1 | 8 | 1 | 6 | 1 | 2,72E-05 | 0,002933841 | 0,003242052 | 0,038332332 |
| 216766_at | AK025152 | Hs.97432 | protein kinase C, epsilon | PRKCE | EG1 | 5 | 4 | 4 | 3 | 2,72E-05 | 0,002933841 | 0,003242052 | 0,038332332 |
| 218585_s_at | NM_016448 | Hs.126774 | denticleless homolog (Drosophila) | DTL | EG1 | 5 | 4 | 4 | 3 | 2,72E-05 | 0,002933841 | 0,003242052 | 0,038332332 |
| 218918_at | NM_020379 | Hs.197043 | mannosidase, alpha, class 1C, member 1 | MAN1C1 | EG1 | 3 | 6 | 2 | 5 | 2,72E-05 | 0,002933841 | 0,003242052 | 0,038332332 |
| 219622_at | NM_017817 | Hs.508720 | RAB20, member RAS oncogene family | RAB20 | EG1 | 4 | 5 | 0 | 7 | 2,72E-05 | 0,002933841 | 0,003242052 | 0,038332332 |
| 219978_s_at | NM_018454 | Hs.511093 | nucleolar and spindle associated protein 1 | NUSAP1 | EG1 | 6 | 3 | 2 | 5 | 2,72E-05 | 0,002933841 | 0,003242052 | 0,038332332 |
| 220941_s_at | NM_017447 | Hs.293811 | chromosome 21 open reading frame 91 | C21orf91 | EG1 | 6 | 3 | 6 | 1 | 2,72E-05 | 0,002933841 | 0,003242052 | 0,038332332 |
| 221276_s_at | NM_030786 | Hs.16003 | syncoilin, intermediate filament 1 | SYNC1 | EG1 | 5 | 4 | 4 | 3 | 2,72E-05 | 0,002933841 | 0,003242052 | 0,038332332 |
| 221985_at | AW006750 | Hs.407709 | kelch-like 24 (Drosophila) | KLHL24 | EG1 | 7 | 2 | 3 | 4 | 2,72E-05 | 0,002933841 | 0,003242052 | 0,038332332 |
| 222592_s_at | AW173691 | Hs.11638 | acyl-CoA synthetase long-chain family member 5 | ACSL5 | EG1 | 1 | 8 | 3 | 4 | 2,72E-05 | 0,002933841 | 0,003242052 | 0,038332332 |
| 225107_at | AI963008 | Hs.535028 | | LOC442518 | EG1 | 8 | 1 | 3 | 4 | 2,72E-05 | 0,002933841 | 0,003242052 | 0,038332332 |
| 225876_at | T84558 | Hs.523442 | NIPA-like domain containing 3 | NPAL3 | EG1 | 2 | 7 | 3 | 4 | 2,72E-05 | 0,002933841 | 0,003242052 | 0,038332332 |
| 226158_at | AL120021 | Hs.407709 | kelch-like 24 (Drosophila) | KLHL24 | EG1 | 7 | 2 | 3 | 4 | 2,72E-05 | 0,002933841 | 0,003242052 | 0,038332332 |
| 226390_at | AA628398 | Hs.93842 | START domain containing 4, sterol regulated | STARD4 | EG1 | 7 | 2 | 6 | 1 | 2,72E-05 | 0,002933841 | 0,003242052 | 0,038332332 |
| 226542_at | AI300571 | | | | EG1 | 5 | 4 | 1 | 6 | 2,72E-05 | 0,002933841 | 0,003242052 | 0,038332332 |
| 227040_at | AI655763 | Hs.507783 | | LOC387921 | EG1 | 4 | 5 | 2 | 5 | 2,72E-05 | 0,002933841 | 0,003242052 | 0,038332332 |
| 228160_at | AI433706 | Hs.552607, Hs.553799 | | LOC400642 | EG1 | 5 | 4 | 3 | 4 | 2,72E-05 | 0,002933841 | 0,003242052 | 0,038332332 |
| 228468_at | BF108964 | Hs.276905 | microtubule associated serine/threonine kinase-like | MASTL | EG1 | 8 | 1 | 2 | 5 | 2,72E-05 | 0,002933841 | 0,003242052 | 0,038332332 |
| 228674_s_at | AA524507 | Hs.432438 | echinoderm microtubule associated protein like 4 | EML4 | EG1 | 4 | 5 | 2 | 5 | 2,72E-05 | 0,002933841 | 0,003242052 | 0,038332332 |
| 229574_at | AI268231 | Hs.445652 | | TRA2A | EG1 | 5 | 4 | 3 | 4 | 2,72E-05 | 0,002933841 | 0,003242052 | 0,038332332 |
| 229685_at | AI743207 | Hs.518611 | TBC1 domain family, member 14 | TBC1D14 | EG1 | 2 | 7 | 1 | 6 | 2,72E-05 | 0,002933841 | 0,003242052 | 0,038332332 |
| 230082_at | AW137053 | Hs.475319 | leucine rich repeat (in FLII) interacting protein 2 | LRRFIP2 | EG1 | 8 | 1 | 7 | 0 | 2,72E-05 | 0,002933841 | 0,003242052 | 0,038332332 |
| 234973_at | BG325630 | Hs.195155 | solute carrier family 38, member 5 | SLC38A5 | EG1 | 3 | 6 | 5 | 2 | 2,72E-05 | 0,002933841 | 0,003242052 | 0,038332332 |
| 235190_at | BF591288 | Hs.282410, H | calmodulin 2 (phosphorylase kinase, delta) | CALM2 | EG1 | 8 | 1 | 7 | 0 | 2,72E-05 | 0,002933841 | 0,003242052 | 0,038332332 |
| 236072_at | N64578 | Hs.435948 | ATPase family, AAA domain containing 1 | ATAD1 | EG1 | 5 | 4 | 4 | 3 | 2,72E-05 | 0,002933841 | 0,003242052 | 0,038332332 |
| 236989_at | AW293012 | Hs.405590 | eukaryotic translation initiation factor 3, subunit 6 48k | EIF3S6 | EG1 | 7 | 2 | 3 | 4 | 2,72E-05 | 0,002933841 | 0,003242052 | 0,038332332 |

| | | | | | | | | | | | | | |
|---|---|---|---|---|---|---|---|---|---|---|---|---|---|
| 238452_at | AI393356 | Hs.517422 | Fc receptor-like and mucin-like 2 | FCRLM2 | EG1 | 2 | 7 | 4 | 3 | 2,72E-05 | 0,0029338410 | 0,0032420520 | 0,038332332 |
| 238781_at | AI859076 | Hs.519347 | splicing factor, arginine/serine-rich 12 | SFRS12 | EG1 | 7 | 2 | 4 | 3 | 2,72E-05 | 0,0029338410 | 0,0032420520 | 0,038332332 |
| 242836_at | AI800470 | | | | EG1 | 5 | 4 | 4 | 3 | 2,72E-05 | 0,0029338410 | 0,0032420520 | 0,038332332 |
| 243016_at | AW271958 | Hs.369762 | thymidylate synthetase | TYMS | EG1 | 7 | 2 | 4 | 3 | 2,72E-05 | 0,0029338410 | 0,0032420520 | 0,038332332 |
| 1565703_at | AL832789 | Hs.75862 | SMAD, mothers against DPP homolog 4 (Drosophila) | SMAD4 | EG1 | 6 | 3 | 4 | 2 | 2,72E-05 | 0,0029338410 | 0,0147492140 | 0,111110101 |
| 1565951_s_at | AJ293392 | Hs.170129 | choroideremia-like (Rab escort protein 2) | CHML | EG1 | 9 | 0 | 3 | 3 | 2,72E-05 | 0,0029338410 | 0,0147492140 | 0,111110101 |
| 201755_at | NM_006739 | Hs.517582 | MCM5 minichromosome maintenance deficient 5, cell | MCM5 | EG1 | 3 | 6 | 2 | 4 | 2,72E-05 | 0,0029338410 | 0,0147492140 | 0,111110101 |
| 202464_s_at | NM_004566 | Hs.195471 | 6-phosphofructo-2-kinase/fructose-2,6-biphosphatas | PFKFB3 | EG1 | 4 | 5 | 3 | 3 | 2,72E-05 | 0,0029338410 | 0,0147492140 | 0,111110101 |
| 202779_s_at | NM_014501 | Hs.396393 | ubiquitin-conjugating enzyme E2S | UBE2S | EG1 | 7 | 2 | 3 | 3 | 2,72E-05 | 0,0029338410 | 0,0147492140 | 0,111110101 |
| 203836_s_at | D84476 | Hs.186486 | mitogen-activated protein kinase kinase kinase 5 | MAP3K5 | EG1 | 4 | 5 | 4 | 2 | 2,72E-05 | 0,0029338410 | 0,0147492140 | 0,111110101 |
| 204194_at | NM_001186 | Hs.154276 | BTB and CNC homology 1, basic leucine zipper transcri | BACH1 | EG1 | 7 | 2 | 3 | 3 | 2,72E-05 | 0,0029338410 | 0,0147492140 | 0,111110101 |
| 205139_s_at | NM_005715 | Hs.309250 | uronyl-2-sulfotransferase | UST | EG1 | 7 | 2 | 1 | 5 | 2,72E-05 | 0,0029338410 | 0,0147492140 | 0,111110101 |
| 205436_s_at | NM_002105 | Hs.477879 | H2A histone family, member X | H2AFX | EG1 | 7 | 2 | 3 | 3 | 2,72E-05 | 0,0029338410 | 0,0147492140 | 0,111110101 |
| 208744_x_at | BG403660 | Hs.36927 | heat shock 105kDa/110kDa protein 1 | HSPH1 | EG1 | 9 | 0 | 4 | 2 | 2,72E-05 | 0,0029338410 | 0,0147492140 | 0,111110101 |
| 209163_at | AL514271 | Hs.355264 | cytochrome b-561 | CYB561 | EG1 | 4 | 5 | 3 | 3 | 2,72E-05 | 0,0029338410 | 0,0147492140 | 0,111110101 |
| 209421_at | U04045 | Hs.156519 | mutS homolog 2, colon cancer, nonpolyposis type 1 (E. | MSH2 | EG1 | 3 | 6 | 2 | 4 | 2,72E-05 | 0,0029338410 | 0,0147492140 | 0,111110101 |
| 209840_s_at | AI221950 | Hs.3781 | leucine rich repeat neuronal 3 | LRRN3 | EG1 | 3 | 6 | 1 | 5 | 2,72E-05 | 0,0029338410 | 0,0147492140 | 0,111110101 |
| 210424_s_at | AF163441 | Hs.182982, H | golgi autoantigen, golgin subfamily a, 8A | GOLGA8A | EG1 | 6 | 3 | 5 | 1 | 2,72E-05 | 0,0029338410 | 0,0147492140 | 0,111110101 |
| 210793_s_at | U41815 | Hs.524750 | nucleoporin 98kDa | NUP98 | EG1 | 7 | 2 | 5 | 1 | 2,72E-05 | 0,0029338410 | 0,0147492140 | 0,111110101 |
| 211323_s_at | L38019 | Hs.374613 | inositol 1,4,5-triphosphate receptor, type 1 | ITPR1 | EG1 | 7 | 2 | 2 | 4 | 2,72E-05 | 0,0029338410 | 0,0147492140 | 0,111110101 |
| 211748_x_at | BC005939 | Hs.446429 | prostaglandin D2 synthase 21kDa (brain) | PTGDS | EG1 | 4 | 5 | 4 | 2 | 2,72E-05 | 0,0029338410 | 0,0147492140 | 0,111110101 |
| 212746_s_at | AA126789 | Hs.408293, H | KIAA0470 | KIAA0470 | EG1 | 6 | 3 | 2 | 4 | 2,72E-05 | 0,0029338410 | 0,0147492140 | 0,111110101 |
| 212890_at | BF663461 | Hs.352240 | | MGC15523 | EG1 | 0 | 9 | 4 | 2 | 2,72E-05 | 0,0029338410 | 0,0147492140 | 0,111110101 |
| 215011_at | AJ006835 | Hs.469723 | RNA, U17D small nucleolar | RNU17D | EG1 | 8 | 1 | 2 | 4 | 2,72E-05 | 0,0029338410 | 0,0147492140 | 0,111110101 |
| 217949_s_at | NM_024006 | Hs.324844 | vitamin K epoxide reductase complex, subunit 1 | VKORC1 | EG1 | 0 | 9 | 4 | 2 | 2,72E-05 | 0,0029338410 | 0,0147492140 | 0,111110101 |
| 218317_x_at | NM_024044 | Hs.460558 | GIY-YIG domain containing 2 | GIYD2 | EG1 | 1 | 8 | 1 | 5 | 2,72E-05 | 0,0029338410 | 0,0147492140 | 0,111110101 |
| 220148_at | NM_022568 | Hs.486520 | aldehyde dehydrogenase 8 family, member A1 | ALDH8A1 | EG1 | 7 | 2 | 1 | 5 | 2,72E-05 | 0,0029338410 | 0,0147492140 | 0,111110101 |
| 222429_at | AF077353 | Hs.436500 | drebrin-like | DBNL | EG1 | 2 | 7 | 2 | 4 | 2,72E-05 | 0,0029338410 | 0,0147492140 | 0,111110101 |
| 223276_at | AF313413 | Hs.29444 | | NID67 | EG1 | 2 | 7 | 5 | 1 | 2,72E-05 | 0,0029338410 | 0,0147492140 | 0,111110101 |
| 223407_at | AL136786 | Hs.307084 | chromosome 16 open reading frame 48 | C16orf48 | EG1 | 1 | 8 | 0 | 6 | 2,72E-05 | 0,0029338410 | 0,0147492140 | 0,111110101 |
| 223577_x_at | AA827878 | Hs.187199, H | metastasis associated lung adenocarcinoma transcript | MALAT1 | EG1 | 6 | 3 | 3 | 3 | 2,72E-05 | 0,0029338410 | 0,0147492140 | 0,111110101 |
| 225541_at | BE274422 | Hs.380933 | ribosomal protein L22-like 1 | RPL22L1 | EG1 | 5 | 4 | 3 | 3 | 2,72E-05 | 0,0029338410 | 0,0147492140 | 0,111110101 |
| 225899_x_at | AL040396 | Hs.484396 | | LOC440395 | EG1 | 5 | 4 | 2 | 4 | 2,72E-05 | 0,0029338410 | 0,0147492140 | 0,111110101 |
| 226922_at | BF215302 | Hs.199561 | RAN binding protein 2 | RANBP2 | EG1 | 8 | 1 | 5 | 1 | 2,72E-05 | 0,0029338410 | 0,0147492140 | 0,111110101 |
| 226923_at | AW205790 | Hs.302287 | sec1 family domain containing 2 | SCFD2 | EG1 | 3 | 6 | 3 | 3 | 2,72E-05 | 0,0029338410 | 0,0147492140 | 0,111110101 |
| 228193_s_at | AI744499 | Hs.507866 | | RGC32 | EG1 | 7 | 2 | 0 | 6 | 2,72E-05 | 0,0029338410 | 0,0147492140 | 0,111110101 |
| 228570_at | BF510581 | Hs.271272, H | BTB (POZ) domain containing 11 | BTBD11 | EG1 | 5 | 4 | 1 | 5 | 2,72E-05 | 0,0029338410 | 0,0147492140 | 0,111110101 |
| 229075_at | AI754871 | | | | EG1 | 6 | 3 | 2 | 4 | 2,72E-05 | 0,0029338410 | 0,0147492140 | 0,111110101 |
| 229393_at | AA088346 | Hs.486466 | l(3)mbt-like 3 (Drosophila) | L3MBTL3 | EG1 | 7 | 2 | 2 | 4 | 2,72E-05 | 0,0029338410 | 0,0147492140 | 0,111110101 |
| 233292_s_at | AK024189 | Hs.434219 | ankyrin repeat and KH domain containing 1 | ANKHD1 | EG1 | 8 | 1 | 4 | 2 | 2,72E-05 | 0,0029338410 | 0,0147492140 | 0,111110101 |
| 235787_at | T65394 | Hs.493361 | CDC37 cell division cycle 37 homolog (S. cerevisiae)-li | CDC37L1 | EG1 | 7 | 2 | 3 | 3 | 2,72E-05 | 0,0029338410 | 0,0147492140 | 0,111110101 |
| 235926_at | AI312527 | Hs.7101 | anaphase promoting complex subunit 5 | ANAPC5 | EG1 | 5 | 4 | 5 | 1 | 2,72E-05 | 0,0029338410 | 0,0147492140 | 0,111110101 |
| 236703_at | BF114733 | | | | EG1 | 5 | 4 | 4 | 2 | 2,72E-05 | 0,0029338410 | 0,0147492140 | 0,111110101 |
| 239228_at | AI298887 | Hs.446484, H | casein kinase 2, alpha 1 polypeptide | CSNK2A1 | EG1 | 4 | 5 | 5 | 1 | 2,72E-05 | 0,0029338410 | 0,0147492140 | 0,111110101 |
| 242110_at | AV735587 | Hs.525287 | Rho GTPase activating protein 5 | ARHGAP5 | EG1 | 6 | 3 | 4 | 2 | 2,72E-05 | 0,0029338410 | 0,0147492140 | 0,111110101 |

| | | | | | | | | | | | | | |
|---|---|---|---|---|---|---|---|---|---|---|---|---|---|
| 244054_at | AI760298 | Hs.445725 | SKI-like | SKIL | EG1 | 7 | 2 | 4 | 2 | 2,72E-05 | 0,0029338410 | 0,0147492140 | 0,111110101 |
| 1555247_a_at | AF394782 | Hs.483329 | Rap guanine nucleotide exchange factor (GEF) 6 | RAPGEF6 | EG1 | 6 | 3 | 0 | 5 | 2,72E-05 | 0,0029338410 | 0,0540847830 | 0,274766231 |
| 1558837_a_at | BQ024490 | | | | EG1 | 3 | 6 | 4 | 1 | 2,72E-05 | 0,0029338410 | 0,0540847830 | 0,274766231 |
| 1559401_a_at | BI052176 | Hs.170498 | zinc finger protein 609 | ZNF609 | EG1 | 4 | 5 | 1 | 4 | 2,72E-05 | 0,0029338410 | 0,0540847830 | 0,274766231 |
| 1570021_at | BC036226 | Hs.434961, Hs.558004 | | LOC360030 | EG1 | 1 | 8 | 4 | 1 | 2,72E-05 | 0,0029338410 | 0,0540847830 | 0,274766231 |
| 200832_s_at | AB032261 | Hs.368641 | stearoyl-CoA desaturase (delta-9-desaturase) | SCD | EG1 | 6 | 3 | 1 | 4 | 2,72E-05 | 0,0029338410 | 0,0540847830 | 0,274766231 |
| 201435_s_at | AW268640 | Hs.249718 | eukaryotic translation initiation factor 4E | EIF4E | EG1 | 7 | 2 | 3 | 2 | 2,72E-05 | 0,0029338410 | 0,0540847830 | 0,274766231 |
| 201601_x_at | NM_003641 | Hs.458414 | interferon induced transmembrane protein 1 (9-27) | IFITM1 | EG1 | 1 | 8 | 3 | 2 | 2,72E-05 | 0,0029338410 | 0,0540847830 | 0,274766231 |
| 201673_s_at | NM_002103 | Hs.386225 | glycogen synthase 1 (muscle) | GYS1 | EG1 | 2 | 7 | 2 | 3 | 2,72E-05 | 0,0029338410 | 0,0540847830 | 0,274766231 |
| 201712_s_at | NM_006267 | Hs.199561 | RAN binding protein 2 | RANBP2 | EG1 | 9 | 0 | 4 | 1 | 2,72E-05 | 0,0029338410 | 0,0540847830 | 0,274766231 |
| 203209_at | BC001866 | Hs.506989 | replication factor C (activator 1) 5, 36.5kDa | RFC5 | EG1 | 4 | 5 | 2 | 3 | 2,72E-05 | 0,0029338410 | 0,0540847830 | 0,274766231 |
| 204243_at | NM_012421 | Hs.205627 | rearranged L-myc fusion | RLF | EG1 | 6 | 3 | 2 | 3 | 2,72E-05 | 0,0029338410 | 0,0540847830 | 0,274766231 |
| 204270_at | AI568728 | Hs.555981 | | FLJ13941 | EG1 | 3 | 6 | 2 | 3 | 2,72E-05 | 0,0029338410 | 0,0540847830 | 0,274766231 |
| 204331_s_at | NM_021107 | Hs.411125 | mitochondrial ribosomal protein S12 | MRPS12 | EG1 | 2 | 7 | 3 | 2 | 2,72E-05 | 0,0029338410 | 0,0540847830 | 0,274766231 |
| 204545_at | NM_000287 | Hs.107410 | peroxisomal biogenesis factor 6 | PEX6 | EG1 | 3 | 6 | 0 | 5 | 2,72E-05 | 0,0029338410 | 0,0540847830 | 0,274766231 |
| 204857_at | NM_003550 | Hs.209128 | MAD1 mitotic arrest deficient-like 1 (yeast) | MAD1L1 | EG1 | 1 | 8 | 1 | 4 | 2,72E-05 | 0,0029338410 | 0,0540847830 | 0,274766231 |
| 207713_s_at | NM_006462 | Hs.247280 | chromosome 20 open reading frame 18 | C20orf18 | EG1 | 1 | 8 | 2 | 3 | 2,72E-05 | 0,0029338410 | 0,0540847830 | 0,274766231 |
| 210048_at | BC001889 | Hs.464622 | N-ethylmaleimide-sensitive factor attachment protei | NAPG | EG1 | 6 | 3 | 2 | 3 | 2,72E-05 | 0,0029338410 | 0,0540847830 | 0,274766231 |
| 217370_x_at | S75762 | Hs.513522 | fusion (involved in t(12, 16) in malignant liposarcoma) | FUS | EG1 | 7 | 2 | 3 | 2 | 2,72E-05 | 0,0029338410 | 0,0540847830 | 0,274766231 |
| 217936_at | AW044631 | Hs.525287 | Rho GTPase activating protein 5 | ARHGAP5 | EG1 | 7 | 2 | 0 | 5 | 2,72E-05 | 0,0029338410 | 0,0540847830 | 0,274766231 |
| 220934_s_at | NM_024084 | Hs.530463 | | MGC3196 | EG1 | 2 | 7 | 3 | 2 | 2,72E-05 | 0,0029338410 | 0,0540847830 | 0,274766231 |
| 221919_at | AW450929 | Hs.210850, H | heterogeneous nuclear ribonucleoprotein A1 | HNRPA1 | EG1 | 9 | 0 | 4 | 1 | 2,72E-05 | 0,0029338410 | 0,0540847830 | 0,274766231 |
| 222872_x_at | AU157541 | Hs.118183 | | FLJ22833 | EG1 | 6 | 3 | 3 | 2 | 2,72E-05 | 0,0029338410 | 0,0540847830 | 0,274766231 |
| 223025_s_at | AF290613 | Hs.71040 | adaptor-related protein complex 1, mu 1 subunit | AP1M1 | EG1 | 4 | 5 | 2 | 3 | 2,72E-05 | 0,0029338410 | 0,0540847830 | 0,274766231 |
| 223839_s_at | AF132203 | Hs.368641 | stearoyl-CoA desaturase (delta-9-desaturase) | SCD | EG1 | 3 | 6 | 1 | 4 | 2,72E-05 | 0,0029338410 | 0,0540847830 | 0,274766231 |
| 224315_at | AL133598 | Hs.485810 | DEAD (Asp-Glu-Ala-Asp) box polypeptide 20 | DDX20 | EG1 | 8 | 1 | 3 | 2 | 2,72E-05 | 0,0029338410 | 0,0540847830 | 0,274766231 |
| 225539_at | AP001745 | Hs.434947 | zinc finger protein 295 | ZNF295 | EG1 | 9 | 0 | 4 | 1 | 2,72E-05 | 0,0029338410 | 0,0540847830 | 0,274766231 |
| 225747_at | AL521634 | Hs.4096 | | FLJ32452 | EG1 | 6 | 3 | 4 | 1 | 2,72E-05 | 0,0029338410 | 0,0540847830 | 0,274766231 |
| 225766_s_at | AA527296 | Hs.482497 | transportin 1 | TNPO1 | EG1 | 7 | 2 | 2 | 3 | 2,72E-05 | 0,0029338410 | 0,0540847830 | 0,274766231 |
| 226018_at | W73230 | Hs.200100 | | Ells1 | EG1 | 6 | 3 | 2 | 3 | 2,72E-05 | 0,0029338410 | 0,0540847830 | 0,274766231 |
| 226541_at | AI808182 | Hs.421095 | F-box protein 30 | FBXO30 | EG1 | 9 | 0 | 4 | 1 | 2,72E-05 | 0,0029338410 | 0,0540847830 | 0,274766231 |
| 228812_at | AI652899 | | | | EG1 | 7 | 2 | 3 | 2 | 2,72E-05 | 0,0029338410 | 0,0540847830 | 0,274766231 |
| 229151_at | BE673587 | Hs.101307 | solute carrier family 14 (urea transporter), member 1 ( | SLC14A1 | EG1 | 6 | 3 | 4 | 1 | 2,72E-05 | 0,0029338410 | 0,0540847830 | 0,274766231 |
| 231252_at | AI680874 | Hs.122934 | | FLJ23861 | EG1 | 6 | 3 | 3 | 2 | 2,72E-05 | 0,0029338410 | 0,0540847830 | 0,274766231 |
| 231793_s_at | AA448956 | Hs.144114 | calcium/calmodulin-dependent protein kinase (CaM k | CAMK2D | EG1 | 7 | 2 | 3 | 2 | 2,72E-05 | 0,0029338410 | 0,0540847830 | 0,274766231 |
| 232092_at | AI268054 | Hs.46791 | mitochondrial carrier triple repeat 1 | MCART1 | EG1 | 8 | 1 | 3 | 2 | 2,72E-05 | 0,0029338410 | 0,0540847830 | 0,274766231 |
| 233406_at | AK022100 | Hs.9997 | | KIAA0256 | EG1 | 2 | 7 | 3 | 2 | 2,72E-05 | 0,0029338410 | 0,0540847830 | 0,274766231 |
| 235067_at | D81987 | Hs.44693, Hs | muskelin 1, intracellular mediator containing kelch m | MKLN1 | EG1 | 7 | 2 | 3 | 2 | 2,72E-05 | 0,0029338410 | 0,0540847830 | 0,274766231 |
| 235848_x_at | N35250 | | | | EG1 | 6 | 3 | 1 | 4 | 2,72E-05 | 0,0029338410 | 0,0540847830 | 0,274766231 |
| 239334_at | AI349167 | Hs.490795 | family with sequence similarity 62 (C2 domain contain | FAM62B | EG1 | 5 | 4 | 4 | 1 | 2,72E-05 | 0,0029338410 | 0,0540847830 | 0,274766231 |
| 239630_at | BF516583 | | | | EG1 | 4 | 5 | 3 | 2 | 2,72E-05 | 0,0029338410 | 0,0540847830 | 0,274766231 |
| 239835_at | AA669114 | Hs.116665 | kelch repeat and BTB (POZ) domain containing 8 | KBTBD8 | EG1 | 5 | 4 | 4 | 1 | 2,72E-05 | 0,0029338410 | 0,0540847830 | 0,274766231 |
| 239979_at | BE645480 | Hs.546467 | epithelial stromal interaction 1 (breast) | EPSTI1 | EG1 | 3 | 6 | 3 | 2 | 2,72E-05 | 0,0029338410 | 0,0540847830 | 0,274766231 |
| 240141_at | BF062399 | | | | EG1 | 4 | 5 | 4 | 1 | 2,72E-05 | 0,0029338410 | 0,0540847830 | 0,274766231 |
| 241388_at | AL567118 | | | | EG1 | 6 | 3 | 2 | 3 | 2,72E-05 | 0,0029338410 | 0,0540847830 | 0,274766231 |

| | | | | | | | | | | | | | |
|---|---|---|---|---|---|---|---|---|---|---|---|---|---|
| 241837_at | AI289774 | Hs.535297 | AT rich interactive domain 5B (MRF1-like) | ARID5B | EG1 | 4 | 5 | 3 | 2 | 2,72E-05 | 0,0029338410 | 0,0540847830 | 0,274766231 |
| 242539_at | AW665509 | Hs.471637 | | MGC42174 | EG1 | 5 | 4 | 2 | 3 | 2,72E-05 | 0,0029338410 | 0,0540847830 | 0,274766231 |
| 243659_at | N63876 | Hs.192221, H | elongation factor, RNA polymerase II, 2 | ELL2 | EG1 | 5 | 4 | 3 | 2 | 2,72E-05 | 0,0029338410 | 0,0540847830 | 0,274766231 |
| AFFX-PheX-5_ | AFFX-PHEX-5 | | | | EG1 | 5 | 4 | 2 | 3 | 2,72E-05 | 0,0029338410 | 0,0540847830 | 0,274766231 |
| 1555878_at | AK094613 | Hs.356794 | ribosomal protein S24 | RPS24 | EG1 | 8 | 1 | 3 | 1 | 2,72E-05 | 0,0029338410 | 0,1581335050 | 0,559565828 |
| 1561640_at | BC008637 | Hs.193725 | proteasome (prosome, macropain) 26S subunit, non-A | PSMD5 | EG1 | 6 | 3 | 2 | 2 | 2,72E-05 | 0,0029338410 | 0,1581335050 | 0,559565828 |
| 201660_at | AL525798 | Hs.471461 | acyl-CoA synthetase long-chain family member 3 | ACSL3 | EG1 | 9 | 0 | 1 | 3 | 2,72E-05 | 0,0029338410 | 0,1581335050 | 0,559565828 |
| 204824_at | NM_004435 | Hs.420106 | endonuclease G | ENDOG | EG1 | 2 | 7 | 1 | 3 | 2,72E-05 | 0,0029338410 | 0,1581335050 | 0,559565828 |
| 205241_at | NM_005138 | Hs.549099 | SCO cytochrome oxidase deficient homolog 2 (yeast) | SCO2 | EG1 | 2 | 7 | 3 | 1 | 2,72E-05 | 0,0029338410 | 0,1581335050 | 0,559565828 |
| 205249_at | NM_000399 | Hs.1395 | early growth response 2 (Krox-20 homolog, Drosophila | EGR2 | EG1 | 3 | 6 | 3 | 1 | 2,72E-05 | 0,0029338410 | 0,1581335050 | 0,559565828 |
| 206613_s_at | NM_005681 | Hs.153088 | TATA box binding protein (TBP)-associated factor, RN | TAF1A | EG1 | 7 | 2 | 2 | 2 | 2,72E-05 | 0,0029338410 | 0,1581335050 | 0,559565828 |
| 209482_at | BC001430 | Hs.416994 | processing of precursor 7, ribonuclease P subunit (S. c | POP7 | EG1 | 3 | 6 | 2 | 2 | 2,72E-05 | 0,0029338410 | 0,1581335050 | 0,559565828 |
| 209919_x_at | L20490 | Hs.287505, H | gamma-glutamyltransferase 1 | GGT1 | EG1 | 3 | 6 | 3 | 1 | 2,72E-05 | 0,0029338410 | 0,1581335050 | 0,559565828 |
| 210075_at | AF151074 | Hs.445113 | membrane-associated ring finger (C3HC4) 2 | MARCH2 | EG1 | 3 | 6 | 2 | 2 | 2,72E-05 | 0,0029338410 | 0,1581335050 | 0,559565828 |
| 210716_s_at | M97501 | Hs.524809 | restin (Reed-Steinberg cell-expressed intermediate fi | RSN | EG1 | 9 | 0 | 3 | 1 | 2,72E-05 | 0,0029338410 | 0,1581335050 | 0,559565828 |
| 212930_at | AW576457 | Hs.506276 | ATPase, Ca++ transporting, plasma membrane 1 | ATP2B1 | EG1 | 8 | 1 | 1 | 3 | 2,72E-05 | 0,0029338410 | 0,1581335050 | 0,559565828 |
| 215043_s_at | X83301 | Hs.519366, Hs.553981 | | SMA5 | EG1 | 2 | 7 | 2 | 2 | 2,72E-05 | 0,0029338410 | 0,1581335050 | 0,559565828 |
| 215567_at | AU144919 | Hs.343173 | chromosome 14 open reading frame 111 | C14orf111 | EG1 | 6 | 3 | 3 | 1 | 2,72E-05 | 0,0029338410 | 0,1581335050 | 0,559565828 |
| 217954_s_at | NM_015153 | Hs.348921 | PHD finger protein 3 | PHF3 | EG1 | 9 | 0 | 1 | 3 | 2,72E-05 | 0,0029338410 | 0,1581335050 | 0,559565828 |
| 218070_s_at | NM_013335 | Hs.27059 | GDP-mannose pyrophosphorylase A | GMPPA | EG1 | 1 | 8 | 2 | 2 | 2,72E-05 | 0,0029338410 | 0,1581335050 | 0,559565828 |
| 218399_s_at | NM_017955 | Hs.34045 | cell division cycle associated 4 | CDCA4 | EG1 | 5 | 4 | 2 | 2 | 2,72E-05 | 0,0029338410 | 0,1581335050 | 0,559565828 |
| 218529_at | NM_016579 | Hs.333427 | CD320 antigen | CD320 | EG1 | 3 | 6 | 3 | 1 | 2,72E-05 | 0,0029338410 | 0,1581335050 | 0,559565828 |
| 219164_s_at | NM_018036 | Hs.168241 | chromosome 14 open reading frame 103 | C14orf103 | EG1 | 7 | 2 | 2 | 2 | 2,72E-05 | 0,0029338410 | 0,1581335050 | 0,559565828 |
| 220368_s_at | NM_017936 | Hs.533887 | KIAA2010 | KIAA2010 | EG1 | 9 | 0 | 3 | 1 | 2,72E-05 | 0,0029338410 | 0,1581335050 | 0,559565828 |
| 221826_at | BE671941 | Hs.157078 | angel homolog 2 (Drosophila) | ANGEL2 | EG1 | 8 | 1 | 1 | 3 | 2,72E-05 | 0,0029338410 | 0,1581335050 | 0,559565828 |
| 224582_s_at | H09085 | Hs.213061 | nuclear casein kinase and cyclin-dependent kinase su | NUCKS1 | EG1 | 8 | 1 | 3 | 1 | 2,72E-05 | 0,0029338410 | 0,1581335050 | 0,559565828 |
| 225197_at | W58461 | Hs.221497 | | PRO0149 | EG1 | 8 | 1 | 0 | 4 | 2,72E-05 | 0,0029338410 | 0,1581335050 | 0,559565828 |
| 225870_s_at | BF569208 | Hs.432413 | trafficking protein particle complex 5 | TRAPPC5 | EG1 | 2 | 7 | 1 | 3 | 2,72E-05 | 0,0029338410 | 0,1581335050 | 0,559565828 |
| 225898_at | AK023015 | Hs.554894 | WD repeat domain 54 | WDR54 | EG1 | 1 | 8 | 4 | 0 | 2,72E-05 | 0,0029338410 | 0,1581335050 | 0,559565828 |
| 227031_at | AV681975 | Hs.487648 | sorting nexin 13 | SNX13 | EG1 | 8 | 1 | 2 | 2 | 2,72E-05 | 0,0029338410 | 0,1581335050 | 0,559565828 |
| 227751_at | AI817145 | Hs.443831, H | programmed cell death 5 | PDCD5 | EG1 | 5 | 4 | 3 | 1 | 2,72E-05 | 0,0029338410 | 0,1581335050 | 0,559565828 |
| 230850_at | AI652647 | Hs.179838 | formin-like 3 | FMNL3 | EG1 | 8 | 1 | 3 | 1 | 2,72E-05 | 0,0029338410 | 0,1581335050 | 0,559565828 |
| 230937_at | AW008207 | Hs.130838 | | LOC442200 | EG1 | 2 | 7 | 1 | 3 | 2,72E-05 | 0,0029338410 | 0,1581335050 | 0,559565828 |
| 235698_at | AA771779 | Hs.461074 | zinc finger protein 90 homolog (mouse) | ZFP90 | EG1 | 4 | 5 | 3 | 1 | 2,72E-05 | 0,0029338410 | 0,1581335050 | 0,559565828 |
| 235791_x_at | AU155298 | Hs.519474 | chromodomain helicase DNA binding protein 1 | CHD1 | EG1 | 8 | 1 | 2 | 2 | 2,72E-05 | 0,0029338410 | 0,1581335050 | 0,559565828 |
| 236059_at | AW168168 | Hs.146079 | | FLJ23342 | EG1 | 8 | 1 | 2 | 2 | 2,72E-05 | 0,0029338410 | 0,1581335050 | 0,559565828 |
| 239106_at | AI801563 | Hs.532326 | carbonic anhydrase VB-like | CA5BL | EG1 | 9 | 0 | 3 | 1 | 2,72E-05 | 0,0029338410 | 0,1581335050 | 0,559565828 |
| 239437_at | AI915827 | | | | EG1 | 8 | 1 | 3 | 1 | 2,72E-05 | 0,0029338410 | 0,1581335050 | 0,559565828 |
| 240600_at | AI342146 | Hs.532091 | adaptor-related protein complex 3, beta 1 subunit | AP3B1 | EG1 | 2 | 7 | 4 | 0 | 2,72E-05 | 0,0029338410 | 0,1581335050 | 0,559565828 |
| 243709_at | BG054799 | Hs.128696 | | FLJ90709 | EG1 | 3 | 6 | 2 | 2 | 2,72E-05 | 0,0029338410 | 0,1581335050 | 0,559565828 |
| 243788_at | AA789293 | Hs.369039 | PHD finger protein 11 | PHF11 | EG1 | 1 | 8 | 2 | 2 | 2,72E-05 | 0,0029338410 | 0,1581335050 | 0,559565828 |
| 1554229_at | AY174896 | Hs.484195 | adult retina protein | LOC153222 | EG1 | 4 | 5 | 2 | 1 | 2,72E-05 | 0,0029338410 | 0,3636349230 | 0,911710613 |
| 1559025_at | AL833770 | Hs.440932 | septin 9 | SEPT9 | EG1 | 7 | 2 | 3 | 0 | 2,72E-05 | 0,0029338410 | 0,3636349230 | 0,911710613 |
| 1563497_at | AL833500 | Hs.473370 | ubiquitin specific peptidase 25 | USP25 | EG1 | 5 | 4 | 2 | 1 | 2,72E-05 | 0,0029338410 | 0,3636349230 | 0,911710613 |
| 201284_s_at | NM_001640 | Hs.517969 | N-acylaminoacyl-peptide hydrolase | APEH | EG1 | 1 | 8 | 1 | 2 | 2,72E-05 | 0,0029338410 | 0,3636349230 | 0,911710613 |

| | | | | | | | | | | | | | |
|---|---|---|---|---|---|---|---|---|---|---|---|---|---|
| 202331_at | NM_000709 | Hs.433307 | branched chain keto acid dehydrogenase E1, alpha pol | BCKDHA | EG1 | 0 | 9 | 1 | 2 | 2,72E-05 | 0,002933841 | 0,363634923 | 0,911710613 |
| 202928_s_at | NM_024165 | Hs.166204 | PHD finger protein 1 | PHF1 | EG1 | 5 | 4 | 1 | 2 | 2,72E-05 | 0,002933841 | 0,363634923 | 0,911710613 |
| 203189_s_at | NM_002496 | Hs.90443 | NADH dehydrogenase (ubiquinone) Fe-S protein 8, 23 | NDUFS8 | EG1 | 2 | 7 | 0 | 3 | 2,72E-05 | 0,002933841 | 0,363634923 | 0,911710613 |
| 206976_s_at | NM_006644 | Hs.36927 | heat shock 105kDa/110kDa protein 1 | HSPH1 | EG1 | 9 | 0 | 2 | 1 | 2,72E-05 | 0,002933841 | 0,363634923 | 0,911710613 |
| 210950_s_at | BC003573 | Hs.546253 | farnesyl-diphosphate farnesyltransferase 1 | FDFT1 | EG1 | 2 | 7 | 1 | 2 | 2,72E-05 | 0,002933841 | 0,363634923 | 0,911710613 |
| 222569_at | AU153746 | Hs.34180 | UDP-glucose ceramide glucosyltransferase-like 1 | UGCGL1 | EG1 | 7 | 2 | 0 | 3 | 2,72E-05 | 0,002933841 | 0,363634923 | 0,911710613 |
| 223109_at | BC001457 | Hs.555929 | TruB pseudouridine (psi) synthase homolog 2 (E. coli) | TRUB2 | EG1 | 1 | 8 | 2 | 1 | 2,72E-05 | 0,002933841 | 0,363634923 | 0,911710613 |
| 223585_x_at | AF151831 | Hs.372541, H | kelch repeat and BTB (POZ) domain containing 2 | KBTBD2 | EG1 | 8 | 1 | 3 | 0 | 2,72E-05 | 0,002933841 | 0,363634923 | 0,911710613 |
| 224489_at | BC006271 | Hs.405514 | | LOC284058 | EG1 | 5 | 4 | 2 | 1 | 2,72E-05 | 0,002933841 | 0,363634923 | 0,911710613 |
| 225830_at | AI052247 | Hs.501149 | PDZ domain containing 8 | PDZK8 | EG1 | 8 | 1 | 3 | 0 | 2,72E-05 | 0,002933841 | 0,363634923 | 0,911710613 |
| 225878_at | AL135264 | Hs.97858 | kinesin family member 1B | KIF1B | EG1 | 9 | 0 | 2 | 1 | 2,72E-05 | 0,002933841 | 0,363634923 | 0,911710613 |
| 226370_at | BG149487 | Hs.495854 | kelch-like 15 (Drosophila) | KLHL15 | EG1 | 8 | 1 | 1 | 2 | 2,72E-05 | 0,002933841 | 0,363634923 | 0,911710613 |
| 226942_at | AI742668 | Hs.304362 | PHD finger protein 20-like 1 | PHF20L1 | EG1 | 8 | 1 | 2 | 1 | 2,72E-05 | 0,002933841 | 0,363634923 | 0,911710613 |
| 228655_at | BE466077 | | | | EG1 | 6 | 3 | 2 | 1 | 2,72E-05 | 0,002933841 | 0,363634923 | 0,911710613 |
| 230970_at | AA975530 | Hs.335205 | slingshot homolog 2 (Drosophila) | SSH2 | EG1 | 4 | 5 | 1 | 2 | 2,72E-05 | 0,002933841 | 0,363634923 | 0,911710613 |
| 232628_at | AI740629 | Hs.97270, Hs | family with sequence similarity 13, member A1 | FAM13A1 | EG1 | 4 | 5 | 2 | 1 | 2,72E-05 | 0,002933841 | 0,363634923 | 0,911710613 |
| 235390_at | AA398321 | Hs.519343 | | P18SRP | EG1 | 9 | 0 | 3 | 0 | 2,72E-05 | 0,002933841 | 0,363634923 | 0,911710613 |
| 235557_at | AW082827 | Hs.348629 | | LOC150763 | EG1 | 5 | 4 | 0 | 3 | 2,72E-05 | 0,002933841 | 0,363634923 | 0,911710613 |
| 235860_at | AA719526 | Hs.345694 | potassium channel modulatory factor 1 | KCMF1 | EG1 | 8 | 1 | 3 | 0 | 2,72E-05 | 0,002933841 | 0,363634923 | 0,911710613 |
| 236310_at | BE645334 | Hs.278064, H | zinc finger protein 37b (KOX 21) | ZNF37B | EG1 | 8 | 1 | 2 | 1 | 2,72E-05 | 0,002933841 | 0,363634923 | 0,911710613 |
| 238032_at | T68858 | Hs.289347 | dehydrogenase/reductase (SDR family) member 3 | DHRS3 | EG1 | 4 | 5 | 0 | 3 | 2,72E-05 | 0,002933841 | 0,363634923 | 0,911710613 |
| 238496_at | AA741074 | Hs.32099 | Wolf-Hirschhorn syndrome candidate 1-like 1 | WHSC1L1 | EG1 | 6 | 3 | 1 | 2 | 2,72E-05 | 0,002933841 | 0,363634923 | 0,911710613 |
| 239070_at | AI434790 | | | | EG1 | 2 | 7 | 0 | 3 | 2,72E-05 | 0,002933841 | 0,363634923 | 0,911710613 |
| AFFX-LysX-3_a | AFFX-LYSX-3 | | | | EG1 | 4 | 5 | 2 | 1 | 2,72E-05 | 0,002933841 | 0,363634923 | 0,911710613 |
| 1559946_s_at | AI860098 | Hs.515846 | RuvB-like 2 (E. coli) | RUVBL2 | EG1 | 2 | 7 | 1 | 1 | 2,72E-05 | 0,002933841 | 0,649016171 | 0,911710613 |
| 200822_x_at | NM_000365 | Hs.524219 | triosephosphate isomerase 1 | TPI1 | EG1 | 1 | 8 | 0 | 2 | 2,72E-05 | 0,002933841 | 0,649016171 | 0,911710613 |
| 204080_at | NM_025077 | Hs.288198 | target of EGR1, member 1 (nuclear) | TOE1 | EG1 | 4 | 5 | 2 | 0 | 2,72E-05 | 0,002933841 | 0,649016171 | 0,911710613 |
| 212436_at | AI967961 | Hs.26837 | tripartite motif-containing 33 | TRIM33 | EG1 | 7 | 2 | 2 | 0 | 2,72E-05 | 0,002933841 | 0,649016171 | 0,911710613 |
| 213672_at | AA621558 | Hs.355867 | methionine-tRNA synthetase | MARS | EG1 | 7 | 2 | 2 | 0 | 2,72E-05 | 0,002933841 | 0,649016171 | 0,911710613 |
| 216960_s_at | AL049646 | Hs.472221 | zinc finger protein 133 (clone pHZ-13) | ZNF133 | EG1 | 0 | 9 | 2 | 0 | 2,72E-05 | 0,002933841 | 0,649016171 | 0,911710613 |
| 219433_at | NM_017745 | Hs.186424 | BCL6 co-repressor | BCOR | EG1 | 7 | 2 | 2 | 0 | 2,72E-05 | 0,002933841 | 0,649016171 | 0,911710613 |
| 222133_s_at | AK022280 | Hs.304362 | PHD finger protein 20-like 1 | PHF20L1 | EG1 | 8 | 1 | 1 | 1 | 2,72E-05 | 0,002933841 | 0,649016171 | 0,911710613 |
| 222580_at | AK023596 | Hs.173001 | zinc finger protein 644 | ZNF644 | EG1 | 8 | 1 | 1 | 1 | 2,72E-05 | 0,002933841 | 0,649016171 | 0,911710613 |
| 223515_s_at | AL136726 | Hs.299208 | coenzyme Q3 homolog, methyltransferase (yeast) | COQ3 | EG1 | 7 | 2 | 1 | 1 | 2,72E-05 | 0,002933841 | 0,649016171 | 0,911710613 |
| 224956_at | AI743689 | Hs.462598 | | 182-FIP | EG1 | 9 | 0 | 2 | 0 | 2,72E-05 | 0,002933841 | 0,649016171 | 0,911710613 |
| 225565_at | AA769455 | Hs.516646 | cAMP responsive element binding protein 1 | CREB1 | EG1 | 1 | 8 | 1 | 1 | 2,72E-05 | 0,002933841 | 0,649016171 | 0,911710613 |
| 225743_at | AI125996 | Hs.33719 | RNA pseudouridylate synthase domain containing 3 | RPUSD3 | EG1 | 3 | 6 | 2 | 0 | 2,72E-05 | 0,002933841 | 0,649016171 | 0,911710613 |
| 225821_s_at | AI744726 | Hs.106204 | | KIAA1327 | EG1 | 8 | 1 | 1 | 1 | 2,72E-05 | 0,002933841 | 0,649016171 | 0,911710613 |
| 226934_at | AU149663 | Hs.369606 | cleavage and polyadenylation specific factor 6, 68kDa | CPSF6 | EG1 | 8 | 1 | 0 | 2 | 2,72E-05 | 0,002933841 | 0,649016171 | 0,911710613 |
| 228764_s_at | AI813313 | Hs.220963 | | MGC5987 | EG1 | 2 | 7 | 0 | 2 | 2,72E-05 | 0,002933841 | 0,649016171 | 0,911710613 |
| 230120_s_at | AI088455 | Hs.528525 | | LOC400966 | EG1 | 7 | 2 | 1 | 1 | 2,72E-05 | 0,002933841 | 0,649016171 | 0,911710613 |
| 235689_at | AL563572 | Hs.531615 | mitochondrial methionyl-tRNA formyltransferase | MTFMT | EG1 | 3 | 6 | 1 | 1 | 2,72E-05 | 0,002933841 | 0,649016171 | 0,911710613 |
| 239946_at | AA776723 | Hs.205572 | | KIAA0922 | EG1 | 7 | 2 | 0 | 2 | 2,72E-05 | 0,002933841 | 0,649016171 | 0,911710613 |
| 200776_s_at | AL518328 | Hs.529231 | | LOC151579 | EG1 | 9 | 0 | 1 | 0 | 2,72E-05 | 0,002933841 | 0,897681849 | 0,911710613 |
| 229914_at | AU148141 | Hs.485420 | | FLJ38717 | EG1 | 8 | 1 | 0 | 1 | 2,72E-05 | 0,002933841 | 0,897681849 | 0,911710613 |

| | | | | | | | | | | | | | |
|---|---|---|---|---|---|---|---|---|---|---|---|---|---|
| 231319_x_at | AI657069 | Hs.373947 | kinesin family member 9 | KIF9 | EG1 | 8 | 1 | 0 | 1 | 2,72E-05 | 0,002933841 | 0,897681849 | 0,911710613 |
| 232709_at | AU156167 | | | | EG1 | 9 | 0 | 1 | 0 | 2,72E-05 | 0,002933841 | 0,897681849 | 0,911710613 |
| 236629_at | AW243177 | Hs.237017 | chromosome 1 open reading frame 69 | C1orf69 | EG1 | 6 | 3 | 1 | 0 | 2,72E-05 | 0,002933841 | 0,897681849 | 0,911710613 |
| 1555858_at | CA430188 | | | | EG1 + EG2 | 12 | 3 | 7 | 2 | 5,34E-13 | 0,002933841 | 8,42E-05 | 0,002977773 |
| 202503_s_at | NM_014736 | Hs.81892 | KIAA0101 | KIAA0101 | EG1 + EG2 | 7 | 7 | 6 | 7 | 2,15E-11 | 0,002933841 | 4,56E-09 | 0,001562433 |
| 209773_s_at | BC001886 | Hs.226390 | ribonucleotide reductase M2 polypeptide | RRM2 | EG1 + EG2 | 8 | 6 | 3 | 7 | 2,15E-11 | 0,002933841 | 9,97E-06 | 0,001562433 |
| 210567_s_at | BC001441 | Hs.23348 | S-phase kinase-associated protein 2 (p45) | SKP2 | EG1 + EG2 | 5 | 9 | 3 | 7 | 2,15E-11 | 0,002933841 | 9,97E-06 | 0,001562433 |
| 1554020_at | BC010091 | Hs.505202 | bicaudal D homolog 1 (Drosophila) | BICD1 | EG1 + EG2 | 10 | 3 | 7 | 4 | 6,10E-10 | 0,002933841 | 9,59E-07 | 0,001562433 |
| 201890_at | BE966236 | Hs.226390 | ribonucleotide reductase M2 polypeptide | RRM2 | EG1 + EG2 | 7 | 5 | 4 | 9 | 1,28E-08 | 0,002933841 | 4,56E-09 | 0,001562433 |
| 242245_at | N90719 | Hs.533853 | synapse defective 1, Rho GTPase, homolog 2 (C. elega | SYDE2 | EG1 + EG2 | 7 | 5 | 5 | 8 | 1,28E-08 | 0,002933841 | 4,56E-09 | 0,001562433 |
| 205822_s_at | NM_002130 | Hs.397729 | 3-hydroxy-3-methylglutaryl-Coenzyme A synthase 1 (s | HMGCS1 | EG1 + EG2 | 9 | 3 | 9 | 3 | 1,28E-08 | 0,002933841 | 7,43E-08 | 0,001562433 |
| 208078_s_at | NM_030751 | Hs.124503 | transcription factor 8 (represses interleukin 2 expressi | TCF8 | EG1 + EG2 | 6 | 6 | 6 | 6 | 1,28E-08 | 0,002933841 | 7,43E-08 | 0,001562433 |
| 225687_at | BC001068 | Hs.472716 | chromosome 20 open reading frame 129 | C20orf129 | EG1 + EG2 | 11 | 1 | 7 | 4 | 1,28E-08 | 0,002933841 | 9,59E-07 | 0,001562433 |
| 238542_at | AA831769 | Hs.250618 | UL16 binding protein 2 | ULBP2 | EG1 + EG2 | 10 | 2 | 7 | 4 | 1,28E-08 | 0,002933841 | 9,59E-07 | 0,001562433 |
| 202705_at | NM_004701 | Hs.194698 | cyclin B2 | CCNB2 | EG1 + EG2 | 5 | 7 | 3 | 7 | 1,28E-08 | 0,002933841 | 9,97E-06 | 0,001562433 |
| 211367_s_at | U13699 | Hs.2490 | caspase 1, apoptosis-related cysteine peptidase (inter | CASP1 | EG1 + EG2 | 2 | 10 | 7 | 3 | 1,28E-08 | 0,002933841 | 9,97E-06 | 0,001562433 |
| 212143_s_at | BF340228 | Hs.450230 | insulin-like growth factor binding protein 3 | IGFBP3 | EG1 + EG2 | 4 | 8 | 7 | 3 | 1,28E-08 | 0,002933841 | 9,97E-06 | 0,001562433 |
| 222118_at | AK023669 | Hs.283532 | | BM039 | EG1 + EG2 | 10 | 2 | 3 | 7 | 1,28E-08 | 0,002933841 | 9,97E-06 | 0,001562433 |
| 1559096_x_at | AK095307 | Hs.216653 | F-box protein 9 | FBXO9 | EG1 + EG2 | 8 | 4 | 8 | 1 | 1,28E-08 | 0,002933841 | 8,42E-05 | 0,002977773 |
| 201694_s_at | NM_001964 | Hs.326035 | early growth response 1 | EGR1 | EG1 + EG2 | 6 | 6 | 7 | 2 | 1,28E-08 | 0,002933841 | 8,42E-05 | 0,002977773 |
| 206102_at | NM_021067 | Hs.360033 | | PSF1 | EG1 + EG2 | 5 | 7 | 3 | 6 | 1,28E-08 | 0,002933841 | 8,42E-05 | 0,002977773 |
| 222846_at | AB038995 | Hs.389733 | RAB8B, member RAS oncogene family | RAB8B | EG1 + EG2 | 10 | 2 | 6 | 3 | 1,28E-08 | 0,002933841 | 8,42E-05 | 0,002977773 |
| 201008_s_at | AA812232 | Hs.533977 | thioredoxin interacting protein | TXNIP | EG1 + EG2 | 2 | 9 | 7 | 7 | 2,08E-07 | 0,002933841 | 2,17E-10 | 0,001562433 |
| 201009_s_at | AI439556 | Hs.533977 | thioredoxin interacting protein | TXNIP | EG1 + EG2 | 2 | 9 | 7 | 7 | 2,08E-07 | 0,002933841 | 2,17E-10 | 0,001562433 |
| 201170_s_at | NM_003670 | Hs.171825 | basic helix-loop-helix domain containing, class B, 2 | BHLHB2 | EG1 + EG2 | 5 | 6 | 10 | 3 | 2,08E-07 | 0,002933841 | 4,56E-09 | 0,001562433 |
| 216607_s_at | U40053 | Hs.417077 | cytochrome P450, family 51, subfamily A, polypeptide | CYP51A1 | EG1 + EG2 | 10 | 1 | 10 | 3 | 2,08E-07 | 0,002933841 | 4,56E-09 | 0,001562433 |
| 1555938_x_at | AI520969 | Hs.533317 | vimentin | VIM | EG1 + EG2 | 8 | 3 | 10 | 2 | 2,08E-07 | 0,002933841 | 7,43E-08 | 0,001562433 |
| 1557527_at | BU789637 | Hs.149261, H | runt-related transcription factor 1 (acute myeloid leuk | RUNX1 | EG1 + EG2 | 2 | 9 | 9 | 3 | 2,08E-07 | 0,002933841 | 7,43E-08 | 0,001562433 |
| 203799_at | NM_014880 | Hs.130014 | CD302 antigen | CD302 | EG1 + EG2 | 7 | 4 | 6 | 6 | 2,08E-07 | 0,002933841 | 7,43E-08 | 0,001562433 |
| 1556821_x_at | H48516 | Hs.508041 | deleted in lymphocytic leukemia, 2 | DLEU2 | EG1 + EG2 | 7 | 4 | 5 | 6 | 2,08E-07 | 0,002933841 | 9,59E-07 | 0,001562433 |
| 213348_at | N33167 | Hs.106070 | cyclin-dependent kinase inhibitor 1C (p57, Kip2) | CDKN1C | EG1 + EG2 | 5 | 6 | 5 | 6 | 2,08E-07 | 0,002933841 | 9,59E-07 | 0,001562433 |
| 214657_s_at | AU134977 | Hs.523789 | | TncRNA | EG1 + EG2 | 8 | 3 | 6 | 5 | 2,08E-07 | 0,002933841 | 9,59E-07 | 0,001562433 |
| 218009_s_at | NM_003981 | Hs.459362 | protein regulator of cytokinesis 1 | PRC1 | EG1 + EG2 | 10 | 1 | 5 | 6 | 2,08E-07 | 0,002933841 | 9,59E-07 | 0,001562433 |
| 223079_s_at | AI828035 | Hs.116448 | glutaminase | GLS | EG1 + EG2 | 10 | 1 | 7 | 4 | 2,08E-07 | 0,002933841 | 9,59E-07 | 0,001562433 |
| 228069_at | AL138828 | Hs.121536 | family with sequence similarity 54, member A | FAM54A | EG1 + EG2 | 9 | 2 | 2 | 9 | 2,08E-07 | 0,002933841 | 9,59E-07 | 0,001562433 |
| 230494_at | AI671885 | Hs.187946 | solute carrier family 20 (phosphate transporter), mem | SLC20A1 | EG1 + EG2 | 7 | 4 | 8 | 3 | 2,08E-07 | 0,002933841 | 9,59E-07 | 0,001562433 |
| 201663_s_at | NM_005496 | Hs.58992 | SMC4 structural maintenance of chromosomes 4-like 1 | SMC4L1 | EG1 + EG2 | 10 | 1 | 5 | 5 | 2,08E-07 | 0,002933841 | 9,97E-06 | 0,001562433 |
| 201790_s_at | AW150953 | Hs.503134 | 7-dehydrocholesterol reductase | DHCR7 | EG1 + EG2 | 7 | 4 | 7 | 3 | 2,08E-07 | 0,002933841 | 9,97E-06 | 0,001562433 |
| 203362_s_at | NM_002358 | Hs.509523, H | MAD2 mitotic arrest deficient-like 1 (yeast) | MAD2L1 | EG1 + EG2 | 7 | 4 | 3 | 7 | 2,08E-07 | 0,002933841 | 9,97E-06 | 0,001562433 |
| 205321_at | NM_001415 | Hs.539684 | eukaryotic translation initiation factor 2, subunit 3 ga | EIF2S3 | EG1 + EG2 | 10 | 1 | 3 | 7 | 2,08E-07 | 0,002933841 | 9,97E-06 | 0,001562433 |
| 208079_s_at | NM_003158 | Hs.250822 | serine/threonine kinase 6 | STK6 | EG1 + EG2 | 9 | 2 | 3 | 7 | 2,08E-07 | 0,002933841 | 9,97E-06 | 0,001562433 |
| 217591_at | BF725121 | Hs.445725 | SKI-like | SKIL | EG1 + EG2 | 9 | 2 | 7 | 3 | 2,08E-07 | 0,002933841 | 9,97E-06 | 0,001562433 |
| 218039_at | NM_016359 | Hs.511093 | nucleolar and spindle associated protein 1 | NUSAP1 | EG1 + EG2 | 6 | 5 | 3 | 7 | 2,08E-07 | 0,002933841 | 9,97E-06 | 0,001562433 |
| 226568_at | AI478747 | Hs.200230 | | LOC284611 | EG1 + EG2 | 9 | 2 | 5 | 5 | 2,08E-07 | 0,002933841 | 9,97E-06 | 0,001562433 |
| 226713_at | AI247881 | Hs.478682 | chromosome 3 open reading frame 6 | C3orf6 | EG1 + EG2 | 5 | 6 | 9 | 1 | 2,08E-07 | 0,002933841 | 9,97E-06 | 0,001562433 |

| | | | | | | | | | | | | | | |
|---|---|---|---|---|---|---|---|---|---|---|---|---|---|---|
| 202095_s_at | NM_001168 | Hs.514527 | baculoviral IAP repeat-containing 5 (survivin) | BIRC5 | EG1 + EG2 | 6 | 5 | 3 | 6 | 2,08E-07 | 0,002933841 | 8,42E-05 | 0,002977773 |
| 209408_at | U63743 | Hs.69360 | kinesin family member 2C | KIF2C | EG1 + EG2 | 5 | 6 | 3 | 6 | 2,08E-07 | 0,002933841 | 8,42E-05 | 0,002977773 |
| 218723_s_at | NM_014059 | Hs.507866 | | RGC32 | EG1 + EG2 | 8 | 3 | 1 | 8 | 2,08E-07 | 0,002933841 | 8,42E-05 | 0,002977773 |
| 220789_s_at | NM_004749 | Hs.231411 | transforming growth factor beta regulator 4 | TBRG4 | EG1 + EG2 | 2 | 9 | 3 | 6 | 2,08E-07 | 0,002933841 | 8,42E-05 | 0,002977773 |
| 223229_at | AB032931 | Hs.5199 | ubiquitin-conjugating enzyme E2T (putative) | UBE2T | EG1 + EG2 | 5 | 6 | 2 | 7 | 2,08E-07 | 0,002933841 | 8,42E-05 | 0,002977773 |
| 224566_at | AI042152 | Hs.523789 | | TncRNA | EG1 + EG2 | 7 | 4 | 7 | 2 | 2,08E-07 | 0,002933841 | 8,42E-05 | 0,002977773 |
| 227044_at | AV705507 | | | | EG1 + EG2 | 6 | 5 | 5 | 4 | 2,08E-07 | 0,002933841 | 8,42E-05 | 0,002977773 |
| 229715_at | AW006182 | | | | EG1 + EG2 | 8 | 3 | 8 | 1 | 2,08E-07 | 0,002933841 | 8,42E-05 | 0,002977773 |
| 232338_at | AU144815 | Hs.285657 | zinc finger protein 431 | ZNF431 | EG1 + EG2 | 8 | 3 | 5 | 4 | 2,08E-07 | 0,002933841 | 8,42E-05 | 0,002977773 |
| 235117_at | AI191897 | Hs.40763 | ankyrin repeat and SOCS box-containing 3 | ASB3 | EG1 + EG2 | 9 | 2 | 7 | 2 | 2,08E-07 | 0,002933841 | 8,42E-05 | 0,002977773 |
| 204205_at | NM_021822 | Hs.474853 | apolipoprotein B mRNA editing enzyme, catalytic poly | APOBEC3G | EG1 + EG2 | 0 | 10 | 8 | 8 | 2,66E-06 | 0,002933841 | 1,94E-13 | 0,001562433 |
| 204439_at | NM_006820 | Hs.389724 | interferon-induced protein 44-like | IFI44L | EG1 + EG2 | 3 | 7 | 8 | 5 | 2,66E-06 | 0,002933841 | 4,56E-09 | 0,001562433 |
| 226388_at | AI675780 | Hs.148105, H | transcription elongation factor A (SII), 3 | TCEA3 | EG1 + EG2 | 2 | 8 | 4 | 9 | 2,66E-06 | 0,002933841 | 4,56E-09 | 0,001562433 |
| 227236_at | AI743596 | Hs.310458 | tetraspanin 2 | TSPAN2 | EG1 + EG2 | 4 | 6 | 7 | 6 | 2,66E-06 | 0,002933841 | 4,56E-09 | 0,001562433 |
| 203973_s_at | NM_005195 | Hs.440829 | CCAAT/enhancer binding protein (C/EBP), delta | CEBPD | EG1 + EG2 | 1 | 9 | 3 | 9 | 2,66E-06 | 0,002933841 | 7,43E-08 | 0,001562433 |
| 204026_s_at | NM_007057 | Hs.42650 | ZW10 interactor | ZWINT | EG1 + EG2 | 5 | 5 | 4 | 8 | 2,66E-06 | 0,002933841 | 7,43E-08 | 0,001562433 |
| 221521_s_at | BC003186 | Hs.433180 | | Pfs2 | EG1 + EG2 | 4 | 6 | 2 | 10 | 2,66E-06 | 0,002933841 | 7,43E-08 | 0,001562433 |
| 224559_at | AF001540 | Hs.187199, H | metastasis associated lung adenocarcinoma transcript | MALAT1 | EG1 + EG2 | 6 | 4 | 5 | 7 | 2,66E-06 | 0,002933841 | 7,43E-08 | 0,001562433 |
| 227099_s_at | AW276078 | Hs.530443 | | LOC387763 | EG1 + EG2 | 7 | 3 | 9 | 3 | 2,66E-06 | 0,002933841 | 7,43E-08 | 0,001562433 |
| 228003_at | AW294640 | Hs.40758 | RAB30, member RAS oncogene family | RAB30 | EG1 + EG2 | 10 | 0 | 5 | 7 | 2,66E-06 | 0,002933841 | 7,43E-08 | 0,001562433 |
| 230664_at | H09657 | Hs.496530 | | MGC39900 | EG1 + EG2 | 6 | 4 | 2 | 10 | 2,66E-06 | 0,002933841 | 7,43E-08 | 0,001562433 |
| 205659_at | NM_014707 | Hs.196054 | histone deacetylase 9 | HDAC9 | EG1 + EG2 | 4 | 6 | 7 | 4 | 2,66E-06 | 0,002933841 | 9,59E-07 | 0,001562433 |
| 214710_s_at | BE407516 | Hs.23960 | cyclin B1 | CCNB1 | EG1 + EG2 | 5 | 5 | 4 | 7 | 2,66E-06 | 0,002933841 | 9,59E-07 | 0,001562433 |
| 227609_at | AA633203 | Hs.546467 | epithelial stromal interaction 1 (breast) | EPSTI1 | EG1 + EG2 | 2 | 8 | 2 | 9 | 2,66E-06 | 0,002933841 | 9,59E-07 | 0,001562433 |
| 238013_at | BF347859 | Hs.369123 | pleckstrin homology domain containing, family A (pho | PLEKHA2 | EG1 + EG2 | 4 | 6 | 5 | 6 | 2,66E-06 | 0,002933841 | 9,59E-07 | 0,001562433 |
| 1553994_at | BC015940 | Hs.153952 | 5'-nucleotidase, ecto (CD73) | NT5E | EG1 + EG2 | 5 | 5 | 5 | 5 | 2,66E-06 | 0,002933841 | 9,97E-06 | 0,001562433 |
| 201250_s_at | NM_006516 | Hs.473721 | solute carrier family 2 (facilitated glucose transporter) | SLC2A1 | EG1 + EG2 | 6 | 4 | 7 | 3 | 2,66E-06 | 0,002933841 | 9,97E-06 | 0,001562433 |
| 201291_s_at | AU159942 | Hs.156346 | topoisomerase (DNA) II alpha 170kDa | TOP2A | EG1 + EG2 | 6 | 4 | 3 | 7 | 2,66E-06 | 0,002933841 | 9,97E-06 | 0,001562433 |
| 202450_s_at | NM_000396 | Hs.523594 | cathepsin K (pycnodysostosis) | CTSK | EG1 + EG2 | 0 | 10 | 9 | 1 | 2,66E-06 | 0,002933841 | 9,97E-06 | 0,001562433 |
| 207564_x_at | NM_003605 | Hs.405410 | O-linked N-acetylglucosamine (GlcNAc) transferase (U | OGT | EG1 + EG2 | 9 | 1 | 7 | 3 | 2,66E-06 | 0,002933841 | 9,97E-06 | 0,001562433 |
| 213158_at | AA045174 | | | | EG1 + EG2 | 8 | 2 | 3 | 7 | 2,66E-06 | 0,002933841 | 9,97E-06 | 0,001562433 |
| 213527_s_at | AI095896 | Hs.301463 | zinc finger protein 688 | ZNF688 | EG1 + EG2 | 2 | 8 | 6 | 4 | 2,66E-06 | 0,002933841 | 9,97E-06 | 0,001562433 |
| 227404_s_at | AI459194 | Hs.326035 | early growth response 1 | EGR1 | EG1 + EG2 | 5 | 5 | 7 | 3 | 2,66E-06 | 0,002933841 | 9,97E-06 | 0,001562433 |
| 227510_x_at | AL037917 | Hs.187199, H | metastasis associated lung adenocarcinoma transcript | MALAT1 | EG1 + EG2 | 7 | 3 | 5 | 5 | 2,66E-06 | 0,002933841 | 9,97E-06 | 0,001562433 |
| 205967_at | NM_003542 | Hs.46423 | histone 1, H4c | HIST1H4C | EG1 + EG2 | 3 | 7 | 3 | 6 | 2,66E-06 | 0,002933841 | 8,42E-05 | 0,002977773 |
| 209970_x_at | M87507 | Hs.2490 | caspase 1, apoptosis-related cysteine peptidase (inter | CASP1 | EG1 + EG2 | 2 | 8 | 7 | 2 | 2,66E-06 | 0,002933841 | 8,42E-05 | 0,002977773 |
| 210052_s_at | AF098158 | Hs.244580 | TPX2, microtubule-associated, homolog (Xenopus lae | TPX2 | EG1 + EG2 | 5 | 5 | 3 | 6 | 2,66E-06 | 0,002933841 | 8,42E-05 | 0,002977773 |
| 213645_at | AF305057 | Hs.180433 | enolase superfamily member 1 | ENOSF1 | EG1 + EG2 | 8 | 2 | 6 | 3 | 2,66E-06 | 0,002933841 | 8,42E-05 | 0,002977773 |
| 215629_s_at | AA905286 | Hs.269768 | | KIAA1799 | EG1 + EG2 | 6 | 4 | 4 | 5 | 2,66E-06 | 0,002933841 | 8,42E-05 | 0,002977773 |
| 226750_at | AI767732 | Hs.480712 | La ribonucleoprotein domain family, member 2 | LARP2 | EG1 + EG2 | 9 | 1 | 7 | 2 | 2,66E-06 | 0,002933841 | 8,42E-05 | 0,002977773 |
| 227164_at | AL521786 | Hs.68714 | splicing factor, arginine/serine-rich 1 (splicing factor 2 | SFRS1 | EG1 + EG2 | 10 | 0 | 3 | 6 | 2,66E-06 | 0,002933841 | 8,42E-05 | 0,002977773 |
| 227961_at | AA130998 | Hs.520898 | cathepsin B | CTSB | EG1 + EG2 | 3 | 7 | 4 | 5 | 2,66E-06 | 0,002933841 | 8,42E-05 | 0,002977773 |
| 228964_at | AI692659 | Hs.436023 | PR domain containing 1, with ZNF domain | PRDM1 | EG1 + EG2 | 0 | 10 | 4 | 5 | 2,66E-06 | 0,002933841 | 8,42E-05 | 0,002977773 |
| 237400_at | AI308101 | Hs.438489 | ATP synthase, H+ transporting, mitochondrial F0 comp | ATP5S | EG1 + EG2 | 4 | 6 | 5 | 4 | 2,66E-06 | 0,002933841 | 8,42E-05 | 0,002977773 |
| 242727_at | BG032269 | Hs.25362 | ADP-ribosylation factor-like 8 | ARL8 | EG1 + EG2 | 9 | 1 | 4 | 5 | 2,66E-06 | 0,002933841 | 8,42E-05 | 0,002977773 |

| | | | | | | | | | | | | | |
|---|---|---|---|---|---|---|---|---|---|---|---|---|---|
| 202016_at | NM_002402 | Hs.270978 | mesoderm specific transcript homolog (mouse) | MEST | EG1 + EG2 | 4 | 5 | 8 | 8 | 2,72E-05 | 0,002933841 | 1,94E-13 | 0,001562433 |
| 219304_s_at | NM_025208 | Hs.352298 | platelet derived growth factor D | PDGFD | EG1 + EG2 | 5 | 4 | 6 | 8 | 2,72E-05 | 0,002933841 | 2,17E-10 | 0,001562433 |
| 201010_s_at | NM_006472 | Hs.533977 | thioredoxin interacting protein | TXNIP | EG1 + EG2 | 2 | 7 | 7 | 6 | 2,72E-05 | 0,002933841 | 4,56E-09 | 0,001562433 |
| 201195_s_at | AB018009 | Hs.513797 | solute carrier family 7 (cationic amino acid transporter | SLC7A5 | EG1 + EG2 | 7 | 2 | 9 | 4 | 2,72E-05 | 0,002933841 | 4,56E-09 | 0,001562433 |
| 219529_at | NM_004669 | Hs.64746 | chloride intracellular channel 3 | CLIC3 | EG1 + EG2 | 0 | 9 | 7 | 6 | 2,72E-05 | 0,002933841 | 4,56E-09 | 0,001562433 |
| 223220_s_at | AF307338 | Hs.518200 | poly (ADP-ribose) polymerase family, member 9 | PARP9 | EG1 + EG2 | 1 | 8 | 4 | 9 | 2,72E-05 | 0,002933841 | 4,56E-09 | 0,001562433 |
| 225606_at | AI949179 | Hs.469658 | BCL2-like 11 (apoptosis facilitator) | BCL2L11 | EG1 + EG2 | 5 | 4 | 7 | 6 | 2,72E-05 | 0,002933841 | 4,56E-09 | 0,001562433 |
| 228582_x_at | AI475544 | Hs.187199, H | metastasis associated lung adenocarcinoma transcript | MALAT1 | EG1 + EG2 | 6 | 3 | 4 | 9 | 2,72E-05 | 0,002933841 | 4,56E-09 | 0,001562433 |
| 201596_x_at | NM_000224 | Hs.406013 | keratin 18 | KRT18 | EG1 + EG2 | 2 | 7 | 4 | 8 | 2,72E-05 | 0,002933841 | 7,43E-08 | 0,001562433 |
| 201688_s_at | BG389015 | Hs.368433 | tumor protein D52 | TPD52 | EG1 + EG2 | 1 | 8 | 9 | 3 | 2,72E-05 | 0,002933841 | 7,43E-08 | 0,001562433 |
| 205807_s_at | NM_020127 | Hs.489922 | tuftelin 1 | TUFT1 | EG1 + EG2 | 6 | 3 | 4 | 8 | 2,72E-05 | 0,002933841 | 7,43E-08 | 0,001562433 |
| 206085_s_at | NM_001902 | Hs.19904 | cystathionase (cystathionine gamma-lyase) | CTH | EG1 + EG2 | 7 | 2 | 8 | 4 | 2,72E-05 | 0,002933841 | 7,43E-08 | 0,001562433 |
| 209765_at | Y13786 | Hs.483944 | ADAM metallopeptidase domain 19 (meltrin beta) | ADAM19 | EG1 + EG2 | 2 | 7 | 3 | 9 | 2,72E-05 | 0,002933841 | 7,43E-08 | 0,001562433 |
| 221841_s_at | BF514079 | Hs.376206 | Kruppel-like factor 4 (gut) | KLF4 | EG1 + EG2 | 3 | 6 | 7 | 5 | 2,72E-05 | 0,002933841 | 7,43E-08 | 0,001562433 |
| 223044_at | AL136944 | Hs.529285 | solute carrier family 40 (iron-regulated transporter), | SLC40A1 | EG1 + EG2 | 2 | 7 | 3 | 9 | 2,72E-05 | 0,002933841 | 7,43E-08 | 0,001562433 |
| 235593_at | AL546529 | Hs.34871 | zinc finger homeobox 1b | ZFHX1B | EG1 + EG2 | 8 | 1 | 7 | 5 | 2,72E-05 | 0,002933841 | 7,43E-08 | 0,001562433 |
| 1555860_x_at | CA430188 | | | | EG1 + EG2 | 8 | 1 | 8 | 3 | 2,72E-05 | 0,002933841 | 9,59E-07 | 0,001562433 |
| 1556035_s_at | AI201248 | Hs.500775 | zinc finger protein 207 | ZNF207 | EG1 + EG2 | 7 | 2 | 8 | 3 | 2,72E-05 | 0,002933841 | 9,59E-07 | 0,001562433 |
| 204334_at | AA488672 | Hs.471221 | Kruppel-like factor 7 (ubiquitous) | KLF7 | EG1 + EG2 | 3 | 6 | 3 | 8 | 2,72E-05 | 0,002933841 | 9,59E-07 | 0,001562433 |
| 204825_at | NM_014791 | Hs.184339 | maternal embryonic leucine zipper kinase | MELK | EG1 + EG2 | 5 | 4 | 3 | 8 | 2,72E-05 | 0,002933841 | 9,59E-07 | 0,001562433 |
| 204908_s_at | NM_005178 | Hs.31210 | B-cell CLL/lymphoma 3 | BCL3 | EG1 + EG2 | 7 | 2 | 4 | 7 | 2,72E-05 | 0,002933841 | 9,59E-07 | 0,001562433 |
| 205282_at | NM_004631 | Hs.444637 | low density lipoprotein receptor-related protein 8, ap | LRP8 | EG1 + EG2 | 7 | 2 | 5 | 6 | 2,72E-05 | 0,002933841 | 9,59E-07 | 0,001562433 |
| 206108_s_at | NM_006275 | Hs.6891 | splicing factor, arginine/serine-rich 6 | SFRS6 | EG1 + EG2 | 6 | 3 | 1 | 10 | 2,72E-05 | 0,002933841 | 9,59E-07 | 0,001562433 |
| 206385_s_at | NM_020987 | Hs.499725 | ankyrin 3, node of Ranvier (ankyrin G) | ANK3 | EG1 + EG2 | 8 | 1 | 3 | 8 | 2,72E-05 | 0,002933841 | 9,59E-07 | 0,001562433 |
| 207563_s_at | U77413 | Hs.405410 | O-linked N-acetylglucosamine (GlcNAc) transferase (U | OGT | EG1 + EG2 | 7 | 2 | 8 | 3 | 2,72E-05 | 0,002933841 | 9,59E-07 | 0,001562433 |
| 212446_s_at | AI658534 | Hs.506829 | LAG1 longevity assurance homolog 6 (S. cerevisiae) | LASS6 | EG1 + EG2 | 6 | 3 | 5 | 6 | 2,72E-05 | 0,002933841 | 9,59E-07 | 0,001562433 |
| 218782_s_at | NM_014109 | Hs.370834 | ATPase family, AAA domain containing 2 | ATAD2 | EG1 + EG2 | 8 | 1 | 4 | 7 | 2,72E-05 | 0,002933841 | 9,59E-07 | 0,001562433 |
| 223062_s_at | BC004863 | Hs.494261 | phosphoserine aminotransferase 1 | PSAT1 | EG1 + EG2 | 7 | 2 | 5 | 6 | 2,72E-05 | 0,002933841 | 9,59E-07 | 0,001562433 |
| 227478_at | BF739885 | Hs.435458 | SET binding protein 1 | SETBP1 | EG1 + EG2 | 3 | 6 | 6 | 5 | 2,72E-05 | 0,002933841 | 9,59E-07 | 0,001562433 |
| 227618_at | AI250910 | Hs.496303 | | FLJ44635 | EG1 + EG2 | 4 | 5 | 8 | 3 | 2,72E-05 | 0,002933841 | 9,59E-07 | 0,001562433 |
| 228390_at | AA489100 | | | | EG1 + EG2 | 7 | 2 | 2 | 9 | 2,72E-05 | 0,002933841 | 9,59E-07 | 0,001562433 |
| 235476_at | AW182459 | Hs.212957 | tripartite motif-containing 59 | TRIM59 | EG1 + EG2 | 3 | 6 | 4 | 7 | 2,72E-05 | 0,002933841 | 9,59E-07 | 0,001562433 |
| 201292_at | AL561834 | Hs.156346 | topoisomerase (DNA) II alpha 170kDa | TOP2A | EG1 + EG2 | 6 | 3 | 4 | 6 | 2,72E-05 | 0,002933841 | 9,97E-06 | 0,001562433 |
| 201650_at | NM_002276 | Hs.514167 | keratin 19 | KRT19 | EG1 + EG2 | 4 | 5 | 5 | 5 | 2,72E-05 | 0,002933841 | 9,97E-06 | 0,001562433 |
| 203158_s_at | AF097493 | Hs.116448 | glutaminase | GLS | EG1 + EG2 | 7 | 2 | 7 | 3 | 2,72E-05 | 0,002933841 | 9,97E-06 | 0,001562433 |
| 203186_s_at | NM_002961 | Hs.81256 | S100 calcium binding protein A4 (calcium protein, calv | S100A4 | EG1 + EG2 | 1 | 8 | 5 | 5 | 2,72E-05 | 0,002933841 | 9,97E-06 | 0,001562433 |
| 203554_x_at | NM_004219 | Hs.350966 | pituitary tumor-transforming 1 | PTTG1 | EG1 + EG2 | 4 | 5 | 6 | 4 | 2,72E-05 | 0,002933841 | 9,97E-06 | 0,001562433 |
| 206114_at | NM_004438 | Hs.371218 | EPH receptor A4 | EPHA4 | EG1 + EG2 | 3 | 6 | 2 | 8 | 2,72E-05 | 0,002933841 | 9,97E-06 | 0,001562433 |
| 208891_at | BC003143 | Hs.298654 | dual specificity phosphatase 6 | DUSP6 | EG1 + EG2 | 6 | 3 | 8 | 2 | 2,72E-05 | 0,002933841 | 9,97E-06 | 0,001562433 |
| 212706_at | AB011110 | Hs.520599 | RAS p21 protein activator 4 | RASA4 | EG1 + EG2 | 1 | 8 | 4 | 6 | 2,72E-05 | 0,002933841 | 9,97E-06 | 0,001562433 |
| 213629_x_at | BF246115 | Hs.513626 | metallothionein 1F (functional) | MT1F | EG1 + EG2 | 2 | 7 | 6 | 4 | 2,72E-05 | 0,002933841 | 9,97E-06 | 0,001562433 |
| 215252_at | AW814026 | Hs.273621, H | DnaJ (Hsp40) homolog, subfamily C, member 7 | DNAJC7 | EG1 + EG2 | 7 | 2 | 6 | 4 | 2,72E-05 | 0,002933841 | 9,97E-06 | 0,001562433 |
| 216252_x_at | Z70519 | Hs.244139 | Fas (TNF receptor superfamily, member 6) | FAS | EG1 + EG2 | 4 | 5 | 8 | 2 | 2,72E-05 | 0,002933841 | 9,97E-06 | 0,001562433 |
| 218894_s_at | NM_018048 | Hs.104650 | | FLJ10292 | EG1 + EG2 | 4 | 5 | 3 | 7 | 2,72E-05 | 0,002933841 | 9,97E-06 | 0,001562433 |
| 218921_at | NM_021805 | Hs.501624 | | SIGIRR | EG1 + EG2 | 0 | 9 | 1 | 9 | 2,72E-05 | 0,002933841 | 9,97E-06 | 0,001562433 |

| | | | | | | | | | | | | | |
|---|---|---|---|---|---|---|---|---|---|---|---|---|---|
| 218983_at | NM_016546 | Hs.525264 | complement component 1, r subcomponent-like | C1RL | EG1 + EG2 | 1 | 8 | 2 | 8 | 2,72E-05 | 0,002933841 | 9,97E-06 | 0,001562433 |
| 221892_at | AK024548 | Hs.463511 | hexose-6-phosphate dehydrogenase (glucose 1-dehy | H6PD | EG1 + EG2 | 0 | 9 | 3 | 7 | 2,72E-05 | 0,002933841 | 9,97E-06 | 0,001562433 |
| 222217_s_at | BC003654 | Hs.438723 | solute carrier family 27 (fatty acid transporter), memb | SLC27A3 | EG1 + EG2 | 2 | 7 | 4 | 6 | 2,72E-05 | 0,002933841 | 9,97E-06 | 0,001562433 |
| 222740_at | AI925583 | Hs.370834 | ATPase family, AAA domain containing 2 | ATAD2 | EG1 + EG2 | 9 | 0 | 3 | 7 | 2,72E-05 | 0,002933841 | 9,97E-06 | 0,001562433 |
| 225341_at | BF697312 | Hs.5009 | MTERF domain containing 3 | MTERFD3 | EG1 + EG2 | 8 | 1 | 5 | 5 | 2,72E-05 | 0,002933841 | 9,97E-06 | 0,001562433 |
| 225485_at | AJ278890 | Hs.368315 | testis specific, 14 | TSGA14 | EG1 + EG2 | 5 | 4 | 3 | 7 | 2,72E-05 | 0,002933841 | 9,97E-06 | 0,001562433 |
| 226666_at | BG434703 | Hs.19156 | dishevelled associated activator of morphogenesis 1 | DAAM1 | EG1 + EG2 | 4 | 5 | 3 | 7 | 2,72E-05 | 0,002933841 | 9,97E-06 | 0,001562433 |
| 227212_s_at | BE544837 | Hs.460124 | PHD finger protein 19 | PHF19 | EG1 + EG2 | 6 | 3 | 4 | 6 | 2,72E-05 | 0,002933841 | 9,97E-06 | 0,001562433 |
| 228793_at | BF002296 | Hs.413416, H | jumonji domain containing 1C | JMJD1C | EG1 + EG2 | 7 | 2 | 7 | 3 | 2,72E-05 | 0,002933841 | 9,97E-06 | 0,001562433 |
| 229007_at | N50864 | | | LOC283788 | EG1 + EG2 | 5 | 4 | 6 | 4 | 2,72E-05 | 0,002933841 | 9,97E-06 | 0,001562433 |
| 229221_at | BE467023 | Hs.502328 | CD44 antigen (homing function and Indian blood grou | CD44 | EG1 + EG2 | 7 | 2 | 5 | 5 | 2,72E-05 | 0,002933841 | 9,97E-06 | 0,001562433 |
| 232369_at | AF339768 | Hs.125715 | muscleblind-like 2 (Drosophila) | MBNL2 | EG1 + EG2 | 3 | 6 | 9 | 1 | 2,72E-05 | 0,002933841 | 9,97E-06 | 0,001562433 |
| 239487_at | AI743261 | Hs.468140 | | DKFZP564F0522 | EG1 + EG2 | 4 | 5 | 7 | 3 | 2,72E-05 | 0,002933841 | 9,97E-06 | 0,001562433 |
| 241755_at | AI961429 | Hs.528803 | ubiquinol-cytochrome c reductase core protein II | UQCRC2 | EG1 + EG2 | 5 | 4 | 4 | 6 | 2,72E-05 | 0,002933841 | 9,97E-06 | 0,001562433 |
| 244871_s_at | AW268357 | Hs.132868 | ubiquitin specific peptidase 32 | USP32 | EG1 + EG2 | 3 | 6 | 7 | 3 | 2,72E-05 | 0,002933841 | 9,97E-06 | 0,001562433 |
| 1555419_a_at | BC016828 | Hs.527412 | N-acylsphingosine amidohydrolase (acid ceramidase) | ASAH1 | EG1 + EG2 | 5 | 4 | 7 | 2 | 2,72E-05 | 0,002933841 | 8,42E-05 | 0,002977773 |
| 1560814_a_at | AK098781 | Hs.146520 | | MGC20481 | EG1 + EG2 | 5 | 4 | 6 | 3 | 2,72E-05 | 0,002933841 | 8,42E-05 | 0,002977773 |
| 201631_s_at | NM_003897 | Hs.76095 | immediate early response 3 | IER3 | EG1 + EG2 | 4 | 5 | 8 | 1 | 2,72E-05 | 0,002933841 | 8,42E-05 | 0,002977773 |
| 201664_at | AL136877 | Hs.58992 | SMC4 structural maintenance of chromosomes 4-like 1 | SMC4L1 | EG1 + EG2 | 7 | 2 | 4 | 5 | 2,72E-05 | 0,002933841 | 8,42E-05 | 0,002977773 |
| 202022_at | NM_005165 | Hs.155247 | aldolase C, fructose-bisphosphate | ALDOC | EG1 + EG2 | 1 | 8 | 5 | 4 | 2,72E-05 | 0,002933841 | 8,42E-05 | 0,002977773 |
| 202668_at | BF001670 | Hs.149239 | ephrin-B2 | EFNB2 | EG1 + EG2 | 7 | 2 | 2 | 7 | 2,72E-05 | 0,002933841 | 8,42E-05 | 0,002977773 |
| 203868_s_at | NM_001078 | Hs.109225 | vascular cell adhesion molecule 1 | VCAM1 | EG1 + EG2 | 6 | 3 | 5 | 4 | 2,72E-05 | 0,002933841 | 8,42E-05 | 0,002977773 |
| 204768_s_at | NM_004111 | Hs.409065 | flap structure-specific endonuclease 1 | FEN1 | EG1 + EG2 | 4 | 5 | 4 | 5 | 2,72E-05 | 0,002933841 | 8,42E-05 | 0,002977773 |
| 205691_at | NM_004209 | Hs.435277 | synaptogyrin 3 | SYNGR3 | EG1 + EG2 | 2 | 7 | 6 | 3 | 2,72E-05 | 0,002933841 | 8,42E-05 | 0,002977773 |
| 205842_s_at | AF001362 | Hs.434374 | Janus kinase 2 (a protein tyrosine kinase) | JAK2 | EG1 + EG2 | 6 | 3 | 4 | 5 | 2,72E-05 | 0,002933841 | 8,42E-05 | 0,002977773 |
| 210461_s_at | BC002448 | Hs.438236, H | actin binding LIM protein 1 | ABLIM1 | EG1 + EG2 | 2 | 7 | 2 | 7 | 2,72E-05 | 0,002933841 | 8,42E-05 | 0,002977773 |
| 213693_s_at | AI610869 | Hs.89603 | mucin 1, transmembrane | MUC1 | EG1 + EG2 | 1 | 8 | 0 | 9 | 2,72E-05 | 0,002933841 | 8,42E-05 | 0,002977773 |
| 214290_s_at | AI313324 | Hs.530461 | histone 2, H2aa | HIST2H2AA | EG1 + EG2 | 4 | 5 | 4 | 5 | 2,72E-05 | 0,002933841 | 8,42E-05 | 0,002977773 |
| 218793_s_at | NM_006746 | Hs.109655 | sex comb on midleg-like 1 (Drosophila) | SCML1 | EG1 + EG2 | 9 | 0 | 3 | 6 | 2,72E-05 | 0,002933841 | 8,42E-05 | 0,002977773 |
| 219787_s_at | NM_018098 | Hs.518299 | epithelial cell transforming sequence 2 oncogene | ECT2 | EG1 + EG2 | 6 | 3 | 4 | 5 | 2,72E-05 | 0,002933841 | 8,42E-05 | 0,002977773 |
| 221750_at | BG035985 | Hs.397729 | 3-hydroxy-3-methylglutaryl-Coenzyme A synthase 1 (s | HMGCS1 | EG1 + EG2 | 7 | 2 | 6 | 3 | 2,72E-05 | 0,002933841 | 8,42E-05 | 0,002977773 |
| 221823_at | AL565741 | Hs.482976 | | LOC90355 | EG1 + EG2 | 9 | 0 | 6 | 3 | 2,72E-05 | 0,002933841 | 8,42E-05 | 0,002977773 |
| 222077_s_at | AU153848 | Hs.505469 | Rac GTPase activating protein 1 | RACGAP1 | EG1 + EG2 | 4 | 5 | 3 | 6 | 2,72E-05 | 0,002933841 | 8,42E-05 | 0,002977773 |
| 224753_at | BE614410 | Hs.434886 | cell division cycle associated 5 | CDCA5 | EG1 + EG2 | 5 | 4 | 3 | 6 | 2,72E-05 | 0,002933841 | 8,42E-05 | 0,002977773 |
| 225521_at | AL137586 | Hs.529280 | anaphase promoting complex subunit 7 | ANAPC7 | EG1 + EG2 | 4 | 5 | 5 | 4 | 2,72E-05 | 0,002933841 | 8,42E-05 | 0,002977773 |
| 226333_at | AV700030 | Hs.135087 | interleukin 6 receptor | IL6R | EG1 + EG2 | 3 | 6 | 3 | 6 | 2,72E-05 | 0,002933841 | 8,42E-05 | 0,002977773 |
| 226350_at | AU155565 | Hs.409081, H | opsin 3 (encephalopsin, panopsin) | OPN3 | EG1 + EG2 | 7 | 2 | 3 | 6 | 2,72E-05 | 0,002933841 | 8,42E-05 | 0,002977773 |
| 226936_at | BG492359 | Hs.486401 | chromosome 6 open reading frame 173 | C6orf173 | EG1 + EG2 | 4 | 5 | 2 | 7 | 2,72E-05 | 0,002933841 | 8,42E-05 | 0,002977773 |
| 227606_s_at | AI638611 | Hs.16229 | | AMSH-LP | EG1 + EG2 | 5 | 4 | 5 | 4 | 2,72E-05 | 0,002933841 | 8,42E-05 | 0,002977773 |
| 228729_at | N90191 | Hs.23960 | cyclin B1 | CCNB1 | EG1 + EG2 | 6 | 3 | 4 | 5 | 2,72E-05 | 0,002933841 | 8,42E-05 | 0,002977773 |
| 229704_at | BF111072 | Hs.369263, H | androgen-induced proliferation inhibitor | APRIN | EG1 + EG2 | 6 | 3 | 7 | 2 | 2,72E-05 | 0,002933841 | 8,42E-05 | 0,002977773 |
| 230505_at | AU158212 | Hs.85077 | | LOC145474 | EG1 + EG2 | 4 | 5 | 7 | 2 | 2,72E-05 | 0,002933841 | 8,42E-05 | 0,002977773 |
| 231769_at | AF129536 | Hs.464419 | F-box protein 6 | FBXO6 | EG1 + EG2 | 3 | 6 | 4 | 5 | 2,72E-05 | 0,002933841 | 8,42E-05 | 0,002977773 |
| 232194_at | AA764787 | Hs.126888 | methyltransferase like 4 | METTL4 | EG1 + EG2 | 7 | 2 | 3 | 6 | 2,72E-05 | 0,002933841 | 8,42E-05 | 0,002977773 |
| 235693_at | D81004 | | | | EG1 + EG2 | 0 | 9 | 6 | 3 | 2,72E-05 | 0,002933841 | 8,42E-05 | 0,002977773 |

| | | | | | | | | | | | | | |
|---|---|---|---|---|---|---|---|---|---|---|---|---|---|
| 236364_at | AA156297 | | | | EG1 + EG2 | 2 | 7 | 5 | 4 | 2,72E-05 | 0,002933841 | 8,42E-05 | 0,002977773 |
| 241769_at | AW962458 | Hs.436873 | integrin, alpha V (vitronectin receptor, alpha polypept | ITGAV | EG1 + EG2 | 3 | 6 | 6 | 3 | 2,72E-05 | 0,002933841 | 8,42E-05 | 0,002977773 |
| 242471_at | AI916641 | | | | EG1 + EG2 | 5 | 4 | 7 | 2 | 2,72E-05 | 0,002933841 | 8,42E-05 | 0,002977773 |
| 204748_at | NM_000963 | Hs.196384 | prostaglandin-endoperoxide synthase 2 (prostaglandi | PTGS2 | EG2 | 1 | 7 | 8 | 6 | 0,000223499 | 0,008816118 | 2,17E-10 | 0,001562433 |
| 201340_s_at | AF010314 | Hs.104925 | ectodermal-neural cortex (with BTB-like domain) | ENC1 | EG2 | 6 | 2 | 9 | 4 | 0,000223499 | 0,008816118 | 4,56E-09 | 0,001562433 |
| 201867_s_at | AW968555 | Hs.495656 | transducin (beta)-like 1X-linked | TBL1X | EG2 | 2 | 6 | 6 | 7 | 0,000223499 | 0,008816118 | 4,56E-09 | 0,001562433 |
| 204529_s_at | AI961231 | Hs.491805 | | TOX | EG2 | 6 | 2 | 4 | 9 | 0,000223499 | 0,008816118 | 4,56E-09 | 0,001562433 |
| 204612_at | NM_006823 | Hs.433700 | protein kinase (cAMP-dependent, catalytic) inhibitor | PKIA | EG2 | 4 | 4 | 8 | 5 | 0,000223499 | 0,008816118 | 4,56E-09 | 0,001562433 |
| 210299_s_at | AF063002 | Hs.435369 | four and a half LIM domains 1 | FHL1 | EG2 | 3 | 5 | 1 | 12 | 0,000223499 | 0,008816118 | 4,56E-09 | 0,001562433 |
| 226610_at | AA722878 | Hs.433422 | proline rich 6 | PRR6 | EG2 | 7 | 1 | 5 | 8 | 0,000223499 | 0,008816118 | 4,56E-09 | 0,001562433 |
| 238599_at | AI168124 | Hs.556018 | interleukin-1 receptor-associated kinase 1 binding pro | IRAK1BP1 | EG2 | 3 | 5 | 3 | 10 | 0,000223499 | 0,008816118 | 4,56E-09 | 0,001562433 |
| 201313_at | NM_001975 | Hs.511915 | enolase 2 (gamma, neuronal) | ENO2 | EG2 | 2 | 6 | 5 | 7 | 0,000223499 | 0,008816118 | 7,43E-08 | 0,001562433 |
| 202086_at | NM_002462 | Hs.517307 | myxovirus (influenza virus) resistance 1, interferon-in | MX1 | EG2 | 1 | 7 | 2 | 10 | 0,000223499 | 0,008816118 | 7,43E-08 | 0,001562433 |
| 202291_s_at | NM_000900 | Hs.365706 | matrix Gla protein | MGP | EG2 | 2 | 6 | 4 | 8 | 0,000223499 | 0,008816118 | 7,43E-08 | 0,001562433 |
| 204279_at | NM_002800 | Hs.381081 | proteasome (prosome, macropain) subunit, beta type | PSMB9 | EG2 | 3 | 5 | 4 | 8 | 0,000223499 | 0,008816118 | 7,43E-08 | 0,001562433 |
| 204457_s_at | NM_002048 | Hs.65029 | growth arrest-specific 1 | GAS1 | EG2 | 3 | 5 | 6 | 6 | 0,000223499 | 0,008816118 | 7,43E-08 | 0,001562433 |
| 207826_s_at | NM_002167 | Hs.76884 | inhibitor of DNA binding 3, dominant negative helix-l | ID3 | EG2 | 6 | 2 | 6 | 6 | 0,000223499 | 0,008816118 | 7,43E-08 | 0,001562433 |
| 208442_s_at | NM_000051 | Hs.435561 | ataxia telangiectasia mutated (includes complementa | ATM | EG2 | 7 | 1 | 7 | 5 | 0,000223499 | 0,008816118 | 7,43E-08 | 0,001562433 |
| 210095_s_at | M31159 | Hs.450230 | insulin-like growth factor binding protein 3 | IGFBP3 | EG2 | 4 | 4 | 8 | 4 | 0,000223499 | 0,008816118 | 7,43E-08 | 0,001562433 |
| 214731_at | AB037854 | Hs.485899 | CTTNBP2 N-terminal like | CTTNBP2NL | EG2 | 5 | 3 | 11 | 1 | 0,000223499 | 0,008816118 | 7,43E-08 | 0,001562433 |
| 218486_at | AA149594 | Hs.12229 | Kruppel-like factor 11 | KLF11 | EG2 | 7 | 1 | 7 | 5 | 0,000223499 | 0,008816118 | 7,43E-08 | 0,001562433 |
| 223578_x_at | AF113016 | Hs.187199, Hs.556259 | | PRO1073 | EG2 | 5 | 3 | 7 | 5 | 0,000223499 | 0,008816118 | 7,43E-08 | 0,001562433 |
| 227449_at | AI799018 | Hs.371218 | EPH receptor A4 | EPHA4 | EG2 | 4 | 4 | 4 | 8 | 0,000223499 | 0,008816118 | 7,43E-08 | 0,001562433 |
| 229899_s_at | AI625235 | Hs.356766, Hs.401929 | | HSUP1 | EG2 | 8 | 0 | 4 | 8 | 0,000223499 | 0,008816118 | 7,43E-08 | 0,001562433 |
| 242273_at | AA747287 | | | | EG2 | 5 | 3 | 3 | 9 | 0,000223499 | 0,008816118 | 7,43E-08 | 0,001562433 |
| 203628_at | H05812 | Hs.20573 | insulin-like growth factor 1 receptor | IGF1R | EG2 | 3 | 5 | 1 | 10 | 0,000223499 | 0,008816118 | 9,59E-07 | 0,001562433 |
| 203919_at | NM_003195 | Hs.505004 | transcription elongation factor A (SII), 2 | TCEA2 | EG2 | 0 | 8 | 2 | 9 | 0,000223499 | 0,008816118 | 9,59E-07 | 0,001562433 |
| 204415_at | NM_022873 | Hs.523847 | interferon, alpha-inducible protein (clone IFI-6-16) | G1P3 | EG2 | 0 | 8 | 3 | 8 | 0,000223499 | 0,008816118 | 9,59E-07 | 0,001562433 |
| 209031_at | AL519710 | Hs.370510 | immunoglobulin superfamily, member 4 | IGSF4 | EG2 | 3 | 5 | 9 | 2 | 0,000223499 | 0,008816118 | 9,59E-07 | 0,001562433 |
| 213164_at | AI867198 | Hs.302742 | mitochondrial ribosomal protein S6 | MRPS6 | EG2 | 6 | 2 | 11 | 0 | 0,000223499 | 0,008816118 | 9,59E-07 | 0,001562433 |
| 213459_at | AU155515 | Hs.433701 | ribosomal protein L37a | RPL37A | EG2 | 7 | 1 | 5 | 6 | 0,000223499 | 0,008816118 | 9,59E-07 | 0,001562433 |
| 214995_s_at | BF508948 | Hs.24656 | KIAA0907 | KIAA0907 | EG2 | 2 | 6 | 5 | 6 | 0,000223499 | 0,008816118 | 9,59E-07 | 0,001562433 |
| 224724_at | AL133001 | Hs.162016 | sulfatase 2 | SULF2 | EG2 | 0 | 8 | 7 | 4 | 0,000223499 | 0,008816118 | 9,59E-07 | 0,001562433 |
| 226876_at | AI961778 | Hs.345588 | | MGC45871 | EG2 | 1 | 7 | 6 | 5 | 0,000223499 | 0,008816118 | 9,59E-07 | 0,001562433 |
| 228846_at | AW071793 | Hs.468908 | MAX dimerization protein 1 | MXD1 | EG2 | 7 | 1 | 8 | 3 | 0,000223499 | 0,008816118 | 9,59E-07 | 0,001562433 |
| 229072_at | BF968097 | | | | EG2 | 6 | 2 | 1 | 10 | 0,000223499 | 0,008816118 | 9,59E-07 | 0,001562433 |
| 232914_s_at | AB046817 | Hs.369520 | synaptotagmin-like 2 | SYTL2 | EG2 | 4 | 4 | 6 | 5 | 0,000223499 | 0,008816118 | 9,59E-07 | 0,001562433 |
| 238931_at | AL045793 | Hs.224187 | | LOC284009 | EG2 | 5 | 3 | 5 | 6 | 0,000223499 | 0,008816118 | 9,59E-07 | 0,001562433 |
| 244766_at | BG180003 | Hs.444600, Hs.460558 | | LAT1-3TM | EG2 | 6 | 2 | 8 | 3 | 0,000223499 | 0,008816118 | 9,59E-07 | 0,001562433 |
| 201169_s_at | BG326045 | Hs.171825 | basic helix-loop-helix domain containing, class B, 2 | BHLHB2 | EG2 | 5 | 3 | 7 | 3 | 0,000223499 | 0,008816118 | 9,97E-06 | 0,001562433 |
| 201925_s_at | NM_000574 | Hs.527653 | decay accelerating factor for complement (CD55, Cro | DAF | EG2 | 5 | 3 | 4 | 6 | 0,000223499 | 0,008816118 | 9,97E-06 | 0,001562433 |
| 202479_s_at | BC002637 | Hs.467751 | tribbles homolog 2 (Drosophila) | TRIB2 | EG2 | 3 | 5 | 5 | 5 | 0,000223499 | 0,008816118 | 9,97E-06 | 0,001562433 |
| 202748_at | NM_004120 | Hs.386567 | guanylate binding protein 2, interferon-inducible | GBP2 | EG2 | 0 | 8 | 1 | 9 | 0,000223499 | 0,008816118 | 9,97E-06 | 0,001562433 |
| 203716_s_at | M80536 | Hs.368912 | dipeptidylpeptidase 4 (CD26, adenosine deaminase c | DPP4 | EG2 | 4 | 4 | 3 | 7 | 0,000223499 | 0,008816118 | 9,97E-06 | 0,001562433 |
| 203764_at | NM_014750 | Hs.77695 | discs, large homolog 7 (Drosophila) | DLG7 | EG2 | 5 | 3 | 3 | 7 | 0,000223499 | 0,008816118 | 9,97E-06 | 0,001562433 |

| | | | | | | | | | | | | | |
|---|---|---|---|---|---|---|---|---|---|---|---|---|---|
| 204784_s_at | NM_022443 | Hs.85195 | myeloid leukemia factor 1 | MLF1 | EG2 | 6 | 2 | 8 | 2 | 0,0002234990,008816118 | | 9,97E-06 | 0,001562433 |
| 206448_at | NM_014951 | Hs.22653 | zinc finger protein 365 | ZNF365 | EG2 | 3 | 5 | 5 | 5 | 0,0002234990,008816118 | | 9,97E-06 | 0,001562433 |
| 208892_s_at | BC003143 | Hs.298654 | dual specificity phosphatase 6 | DUSP6 | EG2 | 5 | 3 | 9 | 1 | 0,0002234990,008816118 | | 9,97E-06 | 0,001562433 |
| 209184_s_at | BF700086 | Hs.442344 | insulin receptor substrate 2 | IRS2 | EG2 | 6 | 2 | 2 | 8 | 0,0002234990,008816118 | | 9,97E-06 | 0,001562433 |
| 209238_at | BE966922 | Hs.530733 | syntaxin 3A | STX3A | EG2 | 4 | 4 | 9 | 1 | 0,0002234990,008816118 | | 9,97E-06 | 0,001562433 |
| 210512_s_at | AF022375 | Hs.73793 | vascular endothelial growth factor | VEGF | EG2 | 4 | 4 | 9 | 1 | 0,0002234990,008816118 | | 9,97E-06 | 0,001562433 |
| 213222_at | AL049593 | Hs.431173 | phospholipase C, beta 1 (phosphoinositide-specific) | PLCB1 | EG2 | 5 | 3 | 3 | 7 | 0,0002234990,008816118 | | 9,97E-06 | 0,001562433 |
| 213226_at | AI346350 | Hs.91728 | exosome component 9 | EXOSC9 | EG2 | 6 | 2 | 4 | 6 | 0,0002234990,008816118 | | 9,97E-06 | 0,001562433 |
| 216041_x_at | AK023348 | Hs.514220 | granulin | GRN | EG2 | 0 | 8 | 8 | 2 | 0,0002234990,008816118 | | 9,97E-06 | 0,001562433 |
| 216894_x_at | D64137 | Hs.106070 | cyclin-dependent kinase inhibitor 1C (p57, Kip2) | CDKN1C | EG2 | 6 | 2 | 4 | 6 | 0,0002234990,008816118 | | 9,97E-06 | 0,001562433 |
| 219024_at | NM_021622 | Hs.287830 | pleckstrin homology domain containing, family A (pho | PLEKHA1 | EG2 | 4 | 4 | 8 | 2 | 0,0002234990,008816118 | | 9,97E-06 | 0,001562433 |
| 220739_s_at | NM_017623 | Hs.150895 | cyclin M3 | CNNM3 | EG2 | 0 | 8 | 7 | 3 | 0,0002234990,008816118 | | 9,97E-06 | 0,001562433 |
| 221484_at | BF691447 | Hs.370487 | UDP-Gal:betaGlcNAc beta 1,4- galactosyltransferase, p | B4GALT5 | EG2 | 5 | 3 | 7 | 3 | 0,0002234990,008816118 | | 9,97E-06 | 0,001562433 |
| 224428_s_at | AY029179 | Hs.470654 | cell division cycle associated 7 | CDCA7 | EG2 | 5 | 3 | 3 | 7 | 0,0002234990,008816118 | | 9,97E-06 | 0,001562433 |
| 225123_at | BE883841 | Hs.120633 | sestrin 3 | SESN3 | EG2 | 6 | 2 | 6 | 4 | 0,0002234990,008816118 | | 9,97E-06 | 0,001562433 |
| 227004_at | AI611074 | Hs.435570 | cyclin-dependent kinase-like 5 | CDKL5 | EG2 | 5 | 3 | 4 | 6 | 0,0002234990,008816118 | | 9,97E-06 | 0,001562433 |
| 227856_at | AI110850 | Hs.23439 | | FLJ39370 | EG2 | 5 | 3 | 6 | 4 | 0,0002234990,008816118 | | 9,97E-06 | 0,001562433 |
| 228157_at | AI125646 | Hs.500775 | zinc finger protein 207 | ZNF207 | EG2 | 7 | 1 | 6 | 4 | 0,0002234990,008816118 | | 9,97E-06 | 0,001562433 |
| 235197_s_at | AV713913 | Hs.226780 | osteopetrosis associated transmembrane protein 1 | OSTM1 | EG2 | 7 | 1 | 8 | 2 | 0,0002234990,008816118 | | 9,97E-06 | 0,001562433 |
| 238623_at | AI633559 | Hs.390428 | mitogen-activated protein kinase kinase kinase 4 | MAP3K4 | EG2 | 7 | 1 | 9 | 1 | 0,0002234990,008816118 | | 9,97E-06 | 0,001562433 |
| 241036_at | AA215451 | Hs.477898 | Hermansky-Pudlak syndrome 3 | HPS3 | EG2 | 6 | 2 | 8 | 2 | 0,0002234990,008816118 | | 9,97E-06 | 0,001562433 |
| 242134_at | AI733194 | | | | EG2 | 8 | 0 | 3 | 7 | 0,0002234990,008816118 | | 9,97E-06 | 0,001562433 |
| 45714_at | AA436930 | Hs.412304, H | host cell factor C1 regulator 1 (XPO1 dependant) | HCFC1R1 | EG2 | 0 | 8 | 4 | 6 | 0,0002234990,008816118 | | 9,97E-06 | 0,001562433 |
| 1552619_a_at | NM_018685 | Hs.62180 | anillin, actin binding protein (scraps homolog, Drosop | ANLN | EG2 | 6 | 2 | 3 | 6 | 0,0002234990,008816118 | | 8,42E-05 | 0,002977773 |
| 1555486_a_at | BC008922 | Hs.19987 | | FLJ14213 | EG2 | 0 | 8 | 7 | 2 | 0,0002234990,008816118 | | 8,42E-05 | 0,002977773 |
| 1555978_s_at | AW467415 | Hs.233936 | | LOC440476 | EG2 | 5 | 3 | 4 | 5 | 0,0002234990,008816118 | | 8,42E-05 | 0,002977773 |
| 1559094_at | AK095307 | Hs.216653 | F-box protein 9 | FBXO9 | EG2 | 6 | 2 | 6 | 3 | 0,0002234990,008816118 | | 8,42E-05 | 0,002977773 |
| 1559190_s_at | AL833150 | Hs.327631 | retinol dehydrogenase 13 (all-trans and 9-cis) | RDH13 | EG2 | 3 | 5 | 1 | 8 | 0,0002234990,008816118 | | 8,42E-05 | 0,002977773 |
| 200632_s_at | NM_006096 | Hs.372914 | N-myc downstream regulated gene 1 | NDRG1 | EG2 | 1 | 7 | 5 | 4 | 0,0002234990,008816118 | | 8,42E-05 | 0,002977773 |
| 200678_x_at | NM_002087 | Hs.514220 | granulin | GRN | EG2 | 0 | 8 | 7 | 2 | 0,0002234990,008816118 | | 8,42E-05 | 0,002977773 |
| 201282_at | NM_002541 | Hs.488181 | oxoglutarate (alpha-ketoglutarate) dehydrogenase (li | OGDH | EG2 | 4 | 4 | 5 | 4 | 0,0002234990,008816118 | | 8,42E-05 | 0,002977773 |
| 202499_s_at | NM_006931 | Hs.210227, H | solute carrier family 2 (facilitated glucose transporter) | SLC2A3 | EG2 | 4 | 4 | 5 | 4 | 0,0002234990,008816118 | | 8,42E-05 | 0,002977773 |
| 203017_s_at | R52678 | Hs.22587 | synovial sarcoma, X breakpoint 2 interacting protein | SSX2IP | EG2 | 7 | 1 | 7 | 2 | 0,0002234990,008816118 | | 8,42E-05 | 0,002977773 |
| 203037_s_at | NM_014751 | Hs.336994 | metastasis suppressor 1 | MTSS1 | EG2 | 2 | 6 | 8 | 1 | 0,0002234990,008816118 | | 8,42E-05 | 0,002977773 |
| 203213_at | AL524035 | Hs.334562 | cell division cycle 2, G1 to S and G2 to M | CDC2 | EG2 | 5 | 3 | 3 | 6 | 0,0002234990,008816118 | | 8,42E-05 | 0,002977773 |
| 203276_at | NM_005573 | Hs.89497 | lamin B1 | LMNB1 | EG2 | 6 | 2 | 2 | 7 | 0,0002234990,008816118 | | 8,42E-05 | 0,002977773 |
| 203755_at | NM_001211 | Hs.36708 | BUB1 budding uninhibited by benzimidazoles 1 homol | BUB1B | EG2 | 5 | 3 | 3 | 6 | 0,0002234990,008816118 | | 8,42E-05 | 0,002977773 |
| 204507_s_at | NM_000945 | Hs.280604 | protein phosphatase 3 (formerly 2B), regulatory subu | PPP3R1 | EG2 | 5 | 3 | 5 | 4 | 0,0002234990,008816118 | | 8,42E-05 | 0,002977773 |
| 204510_at | NM_003503 | Hs.533573 | CDC7 cell division cycle 7 (S. cerevisiae) | CDC7 | EG2 | 5 | 3 | 4 | 5 | 0,0002234990,008816118 | | 8,42E-05 | 0,002977773 |
| 205171_at | NM_002830 | Hs.469809 | protein tyrosine phosphatase, non-receptor type 4 (m | PTPN4 | EG2 | 5 | 3 | 7 | 2 | 0,0002234990,008816118 | | 8,42E-05 | 0,002977773 |
| 205339_at | NM_003035 | Hs.525198 | TAL1 (SCL) interrupting locus | SIL | EG2 | 5 | 3 | 3 | 6 | 0,0002234990,008816118 | | 8,42E-05 | 0,002977773 |
| 206011_at | AI719655 | Hs.2490 | caspase 1, apoptosis-related cysteine peptidase (inter | CASP1 | EG2 | 2 | 6 | 7 | 2 | 0,0002234990,008816118 | | 8,42E-05 | 0,002977773 |
| 208070_s_at | NM_002912 | Hs.232021 | REV3-like, catalytic subunit of DNA polymerase zeta (y | REV3L | EG2 | 6 | 2 | 2 | 7 | 0,0002234990,008816118 | | 8,42E-05 | 0,002977773 |
| 209267_s_at | AB040120 | Hs.288034 | solute carrier family 39 (zinc transporter), member 8 | SLC39A8 | EG2 | 2 | 6 | 1 | 8 | 0,0002234990,008816118 | | 8,42E-05 | 0,002977773 |
| 210559_s_at | D88357 | Hs.334562 | cell division cycle 2, G1 to S and G2 to M | CDC2 | EG2 | 6 | 2 | 3 | 6 | 0,0002234990,008816118 | | 8,42E-05 | 0,002977773 |

| | | | | | | | | | | | | | |
|---|---|---|---|---|---|---|---|---|---|---|---|---|---|
| 211991_s_at | M27487 | Hs.347270 | major histocompatibility complex, class II, DP alpha 1 | HLA-DPA1 | EG2 | 2 | 6 | 7 | 2 | 0,0002234999 | 0,008816118 | 8,42E-05 | 0,002977773 |
| 212141_at | AA604621 | Hs.460184 | MCM4 minichromosome maintenance deficient 4 (S. c | MCM4 | EG2 | 4 | 4 | 4 | 5 | 0,0002234999 | 0,008816118 | 8,42E-05 | 0,002977773 |
| 212188_at | AA551075 | Hs.109438 | potassium channel tetramerisation domain containing | KCTD12 | EG2 | 4 | 4 | 2 | 7 | 0,0002234999 | 0,008816118 | 8,42E-05 | 0,002977773 |
| 212822_at | AA121502 | Hs.477420 | HEG homolog 1 (zebrafish) | HEG1 | EG2 | 3 | 5 | 7 | 2 | 0,0002234999 | 0,008816118 | 8,42E-05 | 0,002977773 |
| 213046_at | AI130920 | Hs.117176 | poly(A) binding protein, nuclear 1 | PABPN1 | EG2 | 7 | 1 | 5 | 4 | 0,0002234999 | 0,008816118 | 8,42E-05 | 0,002977773 |
| 213906_at | AW592266 | Hs.445898 | v-myb myeloblastosis viral oncogene homolog (avian) | MYBL1 | EG2 | 6 | 2 | 4 | 5 | 0,0002234999 | 0,008816118 | 8,42E-05 | 0,002977773 |
| 214012_at | BE551138 | Hs.436186, Hs.440961 | | ARTS-1 | EG2 | 5 | 3 | 2 | 7 | 0,0002234999 | 0,008816118 | 8,42E-05 | 0,002977773 |
| 215388_s_at | X56210 | Hs.154224 | complement factor H-related 1 pseudogene | CFHL1P | EG2 | 1 | 7 | 4 | 5 | 0,0002234999 | 0,008816118 | 8,42E-05 | 0,002977773 |
| 217627_at | BE515346 | Hs.290703 | zinc finger protein 573 | ZNF573 | EG2 | 4 | 4 | 2 | 7 | 0,0002234999 | 0,008816118 | 8,42E-05 | 0,002977773 |
| 217966_s_at | NM_022083 | Hs.518662 | chromosome 1 open reading frame 24 | C1orf24 | EG2 | 3 | 5 | 5 | 4 | 0,0002234999 | 0,008816118 | 8,42E-05 | 0,002977773 |
| 218308_at | NM_006342 | Hs.104019 | transforming, acidic coiled-coil containing protein 3 | TACC3 | EG2 | 4 | 4 | 2 | 7 | 0,0002234999 | 0,008816118 | 8,42E-05 | 0,002977773 |
| 218755_at | NM_005733 | Hs.73625 | kinesin family member 20A | KIF20A | EG2 | 5 | 3 | 2 | 7 | 0,0002234999 | 0,008816118 | 8,42E-05 | 0,002977773 |
| 220987_s_at | NM_030952 | Hs.497512 | NUAK family, SNF1-like kinase, 2 | NUAK2 | EG2 | 7 | 1 | 4 | 5 | 0,0002234999 | 0,008816118 | 8,42E-05 | 0,002977773 |
| 222439_s_at | BE967048 | Hs.160211 | thyroid hormone receptor associated protein 3 | THRAP3 | EG2 | 1 | 7 | 5 | 4 | 0,0002234999 | 0,008816118 | 8,42E-05 | 0,002977773 |
| 222608_s_at | AK023208 | Hs.62180 | anillin, actin binding protein (scraps homolog, Drosop | ANLN | EG2 | 6 | 2 | 2 | 7 | 0,0002234999 | 0,008816118 | 8,42E-05 | 0,002977773 |
| 223307_at | BC002551 | Hs.524216 | cell division cycle associated 3 | CDCA3 | EG2 | 4 | 4 | 2 | 7 | 0,0002234999 | 0,008816118 | 8,42E-05 | 0,002977773 |
| 223940_x_at | AF132202 | Hs.187199, H | metastasis associated lung adenocarcinoma transcript | MALAT1 | EG2 | 6 | 2 | 5 | 4 | 0,0002234999 | 0,008816118 | 8,42E-05 | 0,002977773 |
| 225875_s_at | T84558 | Hs.523442 | NIPA-like domain containing 3 | NPAL3 | EG2 | 2 | 6 | 4 | 5 | 0,0002234999 | 0,008816118 | 8,42E-05 | 0,002977773 |
| 227100_at | AI569766 | Hs.13205 | | B3GTL | EG2 | 4 | 4 | 6 | 3 | 0,0002234999 | 0,008816118 | 8,42E-05 | 0,002977773 |
| 227517_s_at | AI056992 | Hs.531856 | growth arrest-specific 5 | GAS5 | EG2 | 8 | 0 | 5 | 4 | 0,0002234999 | 0,008816118 | 8,42E-05 | 0,002977773 |
| 228049_x_at | AA523172 | | | | EG2 | 6 | 2 | 3 | 6 | 0,0002234999 | 0,008816118 | 8,42E-05 | 0,002977773 |
| 228937_at | AI659800 | Hs.210586 | | FLJ38725 | EG2 | 5 | 3 | 3 | 6 | 0,0002234999 | 0,008816118 | 8,42E-05 | 0,002977773 |
| 229943_at | BF939833 | Hs.436922 | ret finger protein 2 | RFP2 | EG2 | 3 | 5 | 5 | 4 | 0,0002234999 | 0,008816118 | 8,42E-05 | 0,002977773 |
| 230180_at | AA521056 | Hs.528305 | DEAD (Asp-Glu-Ala-Asp) box polypeptide 17 | DDX17 | EG2 | 4 | 4 | 3 | 6 | 0,0002234999 | 0,008816118 | 8,42E-05 | 0,002977773 |
| 230560_at | N21096 | Hs.508958 | syntaxin binding protein 6 (amisyn) | STXBP6 | EG2 | 1 | 7 | 3 | 6 | 0,0002234999 | 0,008816118 | 8,42E-05 | 0,002977773 |
| 231042_s_at | AA809487 | Hs.144114 | calcium/calmodulin-dependent protein kinase (CaM k | CAMK2D | EG2 | 7 | 1 | 3 | 6 | 0,0002234999 | 0,008816118 | 8,42E-05 | 0,002977773 |
| 231817_at | H25097 | Hs.431081 | ubiquitin specific peptidase 53 | USP53 | EG2 | 8 | 0 | 2 | 7 | 0,0002234999 | 0,008816118 | 8,42E-05 | 0,002977773 |
| 231863_at | AF161419 | Hs.489811 | inhibitor of growth family, member 3 | ING3 | EG2 | 6 | 2 | 6 | 3 | 0,0002234999 | 0,008816118 | 8,42E-05 | 0,002977773 |
| 242059_at | AW976631 | Hs.240056 | ethanolamine kinase 1 | ETNK1 | EG2 | 6 | 2 | 6 | 3 | 0,0002234999 | 0,008816118 | 8,42E-05 | 0,002977773 |
| 242389_at | BE887449 | Hs.130293, Hs.534387 | | CROP | EG2 | 5 | 3 | 6 | 3 | 0,0002234999 | 0,008816118 | 8,42E-05 | 0,002977773 |
| 244447_at | AW292830 | Hs.435001 | Kruppel-like factor 10 | KLF10 | EG2 | 5 | 3 | 9 | 0 | 0,0002234999 | 0,008816118 | 8,42E-05 | 0,002977773 |
| 39248_at | N74607 | Hs.234642 | aquaporin 3 | AQP3 | EG2 | 2 | 6 | 2 | 7 | 0,0002234999 | 0,008816118 | 8,42E-05 | 0,002977773 |
| 212810_s_at | W72527 | Hs.323878 | solute carrier family 1 (glutamate/neutral amino acid t | SLC1A4 | EG2 | 6 | 1 | 11 | 6 | 0,0014790570 | 0,028294149 | 3,06E-15 | 0,001562433 |
| 225647_s_at | AI246687 | Hs.128065 | cathepsin C | CTSC | EG2 | 2 | 5 | 10 | 5 | 0,0014790570 | 0,028294149 | 7,74E-12 | 0,001562433 |
| 201397_at | NM_006623 | Hs.487296 | phosphoglycerate dehydrogenase | PHGDH | EG2 | 2 | 5 | 4 | 10 | 0,0014790570 | 0,028294149 | 2,17E-10 | 0,001562433 |
| 220576_at | NM_024989 | Hs.229988 | | PGAP1 | EG2 | 6 | 1 | 8 | 6 | 0,0014790570 | 0,028294149 | 2,17E-10 | 0,001562433 |
| 225842_at | AK026181 | Hs.484885 | pleckstrin homology-like domain, family A, member 1 | PHLDA1 | EG2 | 6 | 1 | 10 | 4 | 0,0014790570 | 0,028294149 | 2,17E-10 | 0,001562433 |
| 229450_at | AI075407 | Hs.47338 | interferon-induced protein with tetratricopeptide rep | IFIT3 | EG2 | 2 | 5 | 7 | 7 | 0,0014790570 | 0,028294149 | 2,17E-10 | 0,001562433 |
| 202934_at | AI761561 | Hs.76090, Hs | hexokinase 2 | HK2 | EG2 | 6 | 1 | 7 | 6 | 0,0014790570 | 0,028294149 | 4,56E-09 | 0,001562433 |
| 209189_at | BC004490 | Hs.25647 | v-fos FBJ murine osteosarcoma viral oncogene homol | FOS | EG2 | 1 | 6 | 6 | 7 | 0,0014790570 | 0,028294149 | 4,56E-09 | 0,001562433 |
| 215177_s_at | AV733308 | Hs.133397 | integrin, alpha 6 | ITGA6 | EG2 | 6 | 1 | 6 | 7 | 0,0014790570 | 0,028294149 | 4,56E-09 | 0,001562433 |
| 221011_s_at | NM_030915 | Hs.468044 | | LBH | EG2 | 3 | 4 | 5 | 8 | 0,0014790570 | 0,028294149 | 4,56E-09 | 0,001562433 |
| 201079_at | NM_004710 | Hs.464210 | synaptogyrin 2 | SYNGR2 | EG2 | 2 | 5 | 6 | 6 | 0,0014790570 | 0,028294149 | 7,43E-08 | 0,001562433 |
| 202528_at | NM_000403 | Hs.76057 | UDP-galactose-4-epimerase | GALE | EG2 | 2 | 5 | 11 | 1 | 0,0014790570 | 0,028294149 | 7,43E-08 | 0,001562433 |
| 202847_at | NM_004563 | Hs.75812 | phosphoenolpyruvate carboxykinase 2 (mitochondrial | PCK2 | EG2 | 0 | 7 | 4 | 8 | 0,0014790570 | 0,028294149 | 7,43E-08 | 0,001562433 |

| | | | | | | | | | | | | | |
|---|---|---|---|---|---|---|---|---|---|---|---|---|---|
| 203139_at | NM_004938 | Hs.380277 | death-associated protein kinase 1 | DAPK1 | EG2 | 2 | 5 | 7 | 5 | 0,0014790570,028294149 | | 7,43E-08 | 0,001562433 |
| 203358_s_at | NM_004456 | Hs.444082 | enhancer of zeste homolog 2 (Drosophila) | EZH2 | EG2 | 5 | 2 | 4 | 8 | 0,0014790570,028294149 | | 7,43E-08 | 0,001562433 |
| 203498_at | NM_005822 | Hs.440168 | Down syndrome critical region gene 1-like 1 | DSCR1L1 | EG2 | 5 | 2 | 5 | 7 | 0,0014790570,028294149 | | 7,43E-08 | 0,001562433 |
| 204011_at | NM_005842 | Hs.18676 | sprouty homolog 2 (Drosophila) | SPRY2 | EG2 | 4 | 3 | 10 | 2 | 0,0014790570,028294149 | | 7,43E-08 | 0,001562433 |
| 204589_at | NM_014840 | Hs.524692 | NUAK family, SNF1-like kinase, 1 | NUAK1 | EG2 | 3 | 4 | 8 | 4 | 0,0014790570,028294149 | | 7,43E-08 | 0,001562433 |
| 210001_s_at | AB005043 | Hs.50640 | suppressor of cytokine signaling 1 | SOCS1 | EG2 | 4 | 3 | 2 | 10 | 0,0014790570,028294149 | | 7,43E-08 | 0,001562433 |
| 228702_at | AL569506 | Hs.150556 | | FLJ43663 | EG2 | 6 | 1 | 11 | 1 | 0,0014790570,028294149 | | 7,43E-08 | 0,001562433 |
| 229744_at | AL556611 | Hs.196983 | sperm specific antigen 2 | SSFA2 | EG2 | 6 | 1 | 9 | 3 | 0,0014790570,028294149 | | 7,43E-08 | 0,001562433 |
| 230179_at | N52572 | Hs.435694 | | LOC285812 | EG2 | 5 | 2 | 11 | 1 | 0,0014790570,028294149 | | 7,43E-08 | 0,001562433 |
| 230930_at | AI435839 | Hs.558072 | | LOC338620 | EG2 | 3 | 4 | 7 | 5 | 0,0014790570,028294149 | | 7,43E-08 | 0,001562433 |
| 231775_at | W65310 | Hs.401745 | tumor necrosis factor receptor superfamily, member 1 | TNFRSF10A | EG2 | 4 | 3 | 6 | 6 | 0,0014790570,028294149 | | 7,43E-08 | 0,001562433 |
| 233020_at | AU154125 | Hs.534212 | SEC22 vesicle trafficking protein-like 1 (S. cerevisiae) | SEC22L1 | EG2 | 3 | 4 | 9 | 3 | 0,0014790570,028294149 | | 7,43E-08 | 0,001562433 |
| 241763_s_at | BF244402 | Hs.403933 | F-box protein 32 | FBXO32 | EG2 | 5 | 2 | 4 | 8 | 0,0014790570,028294149 | | 7,43E-08 | 0,001562433 |
| 1553995_a_at | BC015940 | Hs.153952 | 5'-nucleotidase, ecto (CD73) | NT5E | EG2 | 5 | 2 | 7 | 4 | 0,0014790570,028294149 | | 9,59E-07 | 0,001562433 |
| 1555827_at | AY034790 | Hs.4859 | cyclin L1 | CCNL1 | EG2 | 6 | 1 | 4 | 7 | 0,0014790570,028294149 | | 9,59E-07 | 0,001562433 |
| 1555847_a_at | BU617052 | Hs.436426 | | LOC284454 | EG2 | 6 | 1 | 10 | 1 | 0,0014790570,028294149 | | 9,59E-07 | 0,001562433 |
| 200962_at | AI348010 | Hs.442657 | TBC1 domain family, member 8 (with GRAM domain) | TBC1D8 | EG2 | 7 | 0 | 3 | 8 | 0,0014790570,028294149 | | 9,59E-07 | 0,001562433 |
| 203404_at | NM_014782 | Hs.48924 | armadillo repeat containing, X-linked 2 | ARMCX2 | EG2 | 3 | 4 | 5 | 6 | 0,0014790570,028294149 | | 9,59E-07 | 0,001562433 |
| 204162_at | NM_006101 | Hs.414407 | kinetochore associated 2 | KNTC2 | EG2 | 4 | 3 | 4 | 7 | 0,0014790570,028294149 | | 9,59E-07 | 0,001562433 |
| 205081_at | NM_001311 | Hs.70327 | cysteine-rich protein 1 (intestinal) | CRIP1 | EG2 | 0 | 7 | 4 | 7 | 0,0014790570,028294149 | | 9,59E-07 | 0,001562433 |
| 208690_s_at | BC000915 | Hs.368525 | PDZ and LIM domain 1 (elfin) | PDLIM1 | EG2 | 2 | 5 | 5 | 6 | 0,0014790570,028294149 | | 9,59E-07 | 0,001562433 |
| 209016_s_at | BC002700 | Hs.411501 | keratin 7 | KRT7 | EG2 | 3 | 4 | 5 | 6 | 0,0014790570,028294149 | | 9,59E-07 | 0,001562433 |
| 209891_at | AF225416 | Hs.421956 | spindle pole body component 25 homolog (S. cerevisi | SPBC25 | EG2 | 4 | 3 | 2 | 9 | 0,0014790570,028294149 | | 9,59E-07 | 0,001562433 |
| 209911_x_at | BC002842 | Hs.130853 | histone 1, H2bd | HIST1H2BD | EG2 | 5 | 2 | 6 | 5 | 0,0014790570,028294149 | | 9,59E-07 | 0,001562433 |
| 210524_x_at | AF078844 | | | | EG2 | 1 | 6 | 7 | 4 | 0,0014790570,028294149 | | 9,59E-07 | 0,001562433 |
| 213865_at | AI378788 | Hs.203691 | discoidin, CUB and LCCL domain containing 2 | DCBLD2 | EG2 | 6 | 1 | 10 | 1 | 0,0014790570,028294149 | | 9,59E-07 | 0,001562433 |
| 215071_s_at | AL353759 | Hs.484950 | histone 1, H2ac | HIST1H2AC | EG2 | 5 | 2 | 5 | 6 | 0,0014790570,028294149 | | 9,59E-07 | 0,001562433 |
| 217979_at | NM_014399 | Hs.364544 | tetraspanin 13 | TSPAN13 | EG2 | 6 | 1 | 9 | 2 | 0,0014790570,028294149 | | 9,59E-07 | 0,001562433 |
| 221778_at | BE217882 | Hs.308710 | | KIAA1718 | EG2 | 5 | 2 | 6 | 5 | 0,0014790570,028294149 | | 9,59E-07 | 0,001562433 |
| 225415_at | AA577672 | Hs.518201 | deltex 3-like (Drosophila) | DTX3L | EG2 | 2 | 5 | 4 | 7 | 0,0014790570,028294149 | | 9,59E-07 | 0,001562433 |
| 227223_at | BE466173 | Hs.282901 | RNA-binding region (RNP1, RRM) containing 2 | RNPC2 | EG2 | 1 | 6 | 6 | 5 | 0,0014790570,028294149 | | 9,59E-07 | 0,001562433 |
| 227467_at | AV697515 | Hs.244940 | retinol dehydrogenase 10 (all-trans) | RDH10 | EG2 | 2 | 5 | 5 | 6 | 0,0014790570,028294149 | | 9,59E-07 | 0,001562433 |
| 228948_at | T15545 | Hs.371218 | EPH receptor A4 | EPHA4 | EG2 | 5 | 2 | 3 | 8 | 0,0014790570,028294149 | | 9,59E-07 | 0,001562433 |
| 230746_s_at | AW003173 | Hs.25590 | stanniocalcin 1 | STC1 | EG2 | 3 | 4 | 9 | 2 | 0,0014790570,028294149 | | 9,59E-07 | 0,001562433 |
| 235652_at | AI431345 | Hs.109655 | sex comb on midleg-like 1 (Drosophila) | SCML1 | EG2 | 5 | 2 | 5 | 6 | 0,0014790570,028294149 | | 9,59E-07 | 0,001562433 |
| 235803_at | AA843122 | Hs.370168 | cytokine receptor-like factor 3 | CRLF3 | EG2 | 4 | 3 | 6 | 5 | 0,0014790570,028294149 | | 9,59E-07 | 0,001562433 |
| 236769_at | BE504242 | Hs.153799 | | LOC158402 | EG2 | 3 | 4 | 4 | 7 | 0,0014790570,028294149 | | 9,59E-07 | 0,001562433 |
| 239108_at | H16791 | Hs.298851 | male sterility domain containing 1 | MLSTD1 | EG2 | 1 | 6 | 1 | 10 | 0,0014790570,028294149 | | 9,59E-07 | 0,001562433 |
| 1553185_at | NM_152573 | Hs.129136 | RAS and EF-hand domain containing | RASEF | EG2 | 2 | 5 | 5 | 5 | 0,0014790570,028294149 | | 9,97E-06 | 0,001562433 |
| 1559883_s_at | AF147427 | Hs.472630 | SAM domain and HD domain 1 | SAMHD1 | EG2 | 2 | 5 | 4 | 6 | 0,0014790570,028294149 | | 9,97E-06 | 0,001562433 |
| 200974_at | NM_001613 | Hs.500483 | actin, alpha 2, smooth muscle, aorta | ACTA2 | EG2 | 0 | 7 | 6 | 4 | 0,0014790570,028294149 | | 9,97E-06 | 0,001562433 |
| 201392_s_at | BG031974 | Hs.487062 | insulin-like growth factor 2 receptor | IGF2R | EG2 | 1 | 6 | 8 | 2 | 0,0014790570,028294149 | | 9,97E-06 | 0,001562433 |
| 201752_s_at | AI763123 | Hs.501012 | adducin 3 (gamma) | ADD3 | EG2 | 2 | 5 | 4 | 6 | 0,0014790570,028294149 | | 9,97E-06 | 0,001562433 |
| 202393_s_at | NM_005655 | Hs.435001 | Kruppel-like factor 10 | KLF10 | EG2 | 5 | 2 | 9 | 1 | 0,0014790570,028294149 | | 9,97E-06 | 0,001562433 |
| 202481_at | NM_004753 | Hs.289347 | dehydrogenase/reductase (SDR family) member 3 | DHRS3 | EG2 | 2 | 5 | 2 | 8 | 0,0014790570,028294149 | | 9,97E-06 | 0,001562433 |

| | | | | | | | | | | | | | |
|---|---|---|---|---|---|---|---|---|---|---|---|---|---|
| 204444_at | NM_004523 | Hs.8878 | kinesin family member 11 | KIF11 | EG2 | 4 | 3 | 3 | 7 | 0,001479057 | 0,028294149 | 9,97E-06 | 0,001562433 |
| 204793_at | NM_014710 | Hs.522730 | G protein-coupled receptor associated sorting protein | GPRASP1 | EG2 | 6 | 1 | 1 | 9 | 0,001479057 | 0,028294149 | 9,97E-06 | 0,001562433 |
| 205884_at | NM_000885 | Hs.555880 | integrin, alpha 4 (antigen CD49D, alpha 4 subunit of VL | ITGA4 | EG2 | 5 | 2 | 1 | 9 | 0,001479057 | 0,028294149 | 9,97E-06 | 0,001562433 |
| 209255_at | D87454 | Hs.520710 | | KIAA0265 | EG2 | 4 | 3 | 7 | 3 | 0,001479057 | 0,028294149 | 9,97E-06 | 0,001562433 |
| 209304_x_at | AF087853 | Hs.110571 | growth arrest and DNA-damage-inducible, beta | GADD45B | EG2 | 3 | 4 | 5 | 5 | 0,001479057 | 0,028294149 | 9,97E-06 | 0,001562433 |
| 210868_s_at | BC001305 | Hs.412939 | ELOVL family member 6, elongation of long chain fatty | ELOVL6 | EG2 | 6 | 1 | 4 | 6 | 0,001479057 | 0,028294149 | 9,97E-06 | 0,001562433 |
| 212099_at | AI263909 | Hs.502876 | ras homolog gene family, member B | RHOB | EG2 | 4 | 3 | 7 | 3 | 0,001479057 | 0,028294149 | 9,97E-06 | 0,001562433 |
| 212307_s_at | BF001665 | Hs.405410 | O-linked N-acetylglucosamine (GlcNAc) transferase (U | OGT | EG2 | 6 | 1 | 7 | 3 | 0,001479057 | 0,028294149 | 9,97E-06 | 0,001562433 |
| 215191_at | AW836210 | Hs.124147 | F-box and leucine-rich repeat protein 11 | FBXL11 | EG2 | 4 | 3 | 6 | 4 | 0,001479057 | 0,028294149 | 9,97E-06 | 0,001562433 |
| 215719_x_at | X83493 | Hs.244139 | Fas (TNF receptor superfamily, member 6) | FAS | EG2 | 3 | 4 | 8 | 2 | 0,001479057 | 0,028294149 | 9,97E-06 | 0,001562433 |
| 217997_at | AI795908 | Hs.484885 | pleckstrin homology-like domain, family A, member 1 | PHLDA1 | EG2 | 6 | 1 | 8 | 2 | 0,001479057 | 0,028294149 | 9,97E-06 | 0,001562433 |
| 218273_s_at | NM_018444 | Hs.22265 | protein phosphatase 2C, magnesium-dependent, cata | PPM2C | EG2 | 4 | 3 | 4 | 6 | 0,001479057 | 0,028294149 | 9,97E-06 | 0,001562433 |
| 218724_s_at | NM_021809 | Hs.292281 | TGFB-induced factor 2 (TALE family homeobox) | TGIF2 | EG2 | 5 | 2 | 1 | 9 | 0,001479057 | 0,028294149 | 9,97E-06 | 0,001562433 |
| 219038_at | NM_024657 | Hs.496544 | MORC family CW-type zinc finger 4 | MORC4 | EG2 | 4 | 3 | 9 | 1 | 0,001479057 | 0,028294149 | 9,97E-06 | 0,001562433 |
| 221766_s_at | AW246673 | Hs.10784 | family with sequence similarity 46, member A | FAM46A | EG2 | 5 | 2 | 7 | 3 | 0,001479057 | 0,028294149 | 9,97E-06 | 0,001562433 |
| 222696_at | BF684446 | Hs.156527 | axin 2 (conductin, axil) | AXIN2 | EG2 | 3 | 4 | 1 | 9 | 0,001479057 | 0,028294149 | 9,97E-06 | 0,001562433 |
| 226269_at | BF002104 | Hs.168950 | ganglioside-induced differentiation-associated protei | GDAP1 | EG2 | 5 | 2 | 2 | 8 | 0,001479057 | 0,028294149 | 9,97E-06 | 0,001562433 |
| 229881_at | R41200 | Hs.373857 | Kruppel-like factor 12 | KLF12 | EG2 | 6 | 1 | 4 | 6 | 0,001479057 | 0,028294149 | 9,97E-06 | 0,001562433 |
| 230958_s_at | BE670797 | | | | EG2 | 6 | 1 | 5 | 5 | 0,001479057 | 0,028294149 | 9,97E-06 | 0,001562433 |
| 234989_at | AV699657 | Hs.523789 | | TncRNA | EG2 | 5 | 2 | 7 | 3 | 0,001479057 | 0,028294149 | 9,97E-06 | 0,001562433 |
| 235061_at | AV706522 | Hs.291000 | protein phosphatase 1K (PP2C domain containing) | PPM1K | EG2 | 7 | 0 | 5 | 5 | 0,001479057 | 0,028294149 | 9,97E-06 | 0,001562433 |
| 235463_s_at | AI081356 | Hs.506829 | LAG1 longevity assurance homolog 6 (S. cerevisiae) | LASS6 | EG2 | 5 | 2 | 4 | 6 | 0,001479057 | 0,028294149 | 9,97E-06 | 0,001562433 |
| 236600_at | AI651603 | Hs.440414 | spastic paraplegia 20, spartin (Troyer syndrome) | SPG20 | EG2 | 6 | 1 | 4 | 6 | 0,001479057 | 0,028294149 | 9,97E-06 | 0,001562433 |
| 238002_at | BF342391 | Hs.143600 | golgi phosphoprotein 4 | GOLPH4 | EG2 | 7 | 0 | 7 | 3 | 0,001479057 | 0,028294149 | 9,97E-06 | 0,001562433 |
| 239251_at | AW963634 | Hs.429581 | reticulon 4 | RTN4 | EG2 | 4 | 3 | 8 | 2 | 0,001479057 | 0,028294149 | 9,97E-06 | 0,001562433 |
| 241722_x_at | BF724558 | | | | EG2 | 3 | 4 | 7 | 3 | 0,001479057 | 0,028294149 | 9,97E-06 | 0,001562433 |
| AFFX-HUMRG | AFFX-HUMRGE/M10098_5 | | | | EG2 | 3 | 4 | 2 | 8 | 0,001479057 | 0,028294149 | 9,97E-06 | 0,001562433 |
| 1553575_at | NM_173714 | | | | EG2 | 1 | 6 | 3 | 6 | 0,001479057 | 0,028294149 | 8,42E-05 | 0,002977773 |
| 1554408_a_at | BC007986 | Hs.515122 | thymidine kinase 1, soluble | TK1 | EG2 | 3 | 4 | 3 | 6 | 0,001479057 | 0,028294149 | 8,42E-05 | 0,002977773 |
| 1554478_a_at | BC033077 | Hs.313917 | | FLJ20718 | EG2 | 5 | 2 | 2 | 7 | 0,001479057 | 0,028294149 | 8,42E-05 | 0,002977773 |
| 1555852_at | AI375915 | Hs.352018 | transporter 1, ATP-binding cassette, sub-family B (MD | TAP1 | EG2 | 2 | 5 | 2 | 7 | 0,001479057 | 0,028294149 | 8,42E-05 | 0,002977773 |
| 201666_at | NM_003254 | Hs.522632 | TIMP metallopeptidase inhibitor 1 | TIMP1 | EG2 | 0 | 7 | 6 | 3 | 0,001479057 | 0,028294149 | 8,42E-05 | 0,002977773 |
| 202269_x_at | BC002666 | Hs.62661, Hs | guanylate binding protein 1, interferon-inducible, 67k | GBP1 | EG2 | 1 | 6 | 2 | 7 | 0,001479057 | 0,028294149 | 8,42E-05 | 0,002977773 |
| 202270_at | NM_002053 | Hs.62661, Hs | guanylate binding protein 1, interferon-inducible, 67k | GBP1 | EG2 | 1 | 6 | 2 | 7 | 0,001479057 | 0,028294149 | 8,42E-05 | 0,002977773 |
| 202534_x_at | NM_000791 | Hs.83765, Hs | dihydrofolate reductase | DHFR | EG2 | 3 | 4 | 2 | 7 | 0,001479057 | 0,028294149 | 8,42E-05 | 0,002977773 |
| 203373_at | NM_003877 | Hs.485572 | suppressor of cytokine signaling 2 | SOCS2 | EG2 | 2 | 5 | 6 | 3 | 0,001479057 | 0,028294149 | 8,42E-05 | 0,002977773 |
| 203586_s_at | NM_001661 | Hs.183153 | ADP-ribosylation factor 4-like | ARF4L | EG2 | 6 | 1 | 5 | 4 | 0,001479057 | 0,028294149 | 8,42E-05 | 0,002977773 |
| 203665_at | NM_002133 | Hs.517581 | heme oxygenase (decycling) 1 | HMOX1 | EG2 | 4 | 3 | 8 | 1 | 0,001479057 | 0,028294149 | 8,42E-05 | 0,002977773 |
| 204780_s_at | AA164751 | Hs.244139 | Fas (TNF receptor superfamily, member 6) | FAS | EG2 | 2 | 5 | 7 | 2 | 0,001479057 | 0,028294149 | 8,42E-05 | 0,002977773 |
| 205443_at | NM_003082 | Hs.179312 | small nuclear RNA activating complex, polypeptide 1, | SNAPC1 | EG2 | 4 | 3 | 4 | 5 | 0,001479057 | 0,028294149 | 8,42E-05 | 0,002977773 |
| 205518_s_at | NM_003570 | Hs.484918 | cytidine monophosphate-N-acetylneuraminic acid hy | CMAH | EG2 | 3 | 4 | 2 | 7 | 0,001479057 | 0,028294149 | 8,42E-05 | 0,002977773 |
| 205608_s_at | U83508 | Hs.369675 | angiopoietin 1 | ANGPT1 | EG2 | 7 | 0 | 4 | 5 | 0,001479057 | 0,028294149 | 8,42E-05 | 0,002977773 |
| 206561_s_at | NM_020299 | Hs.116724 | aldo-keto reductase family 1, member B10 (aldose red | AKR1B10 | EG2 | 6 | 1 | 5 | 4 | 0,001479057 | 0,028294149 | 8,42E-05 | 0,002977773 |
| 208960_s_at | BE675435 | Hs.4055 | Kruppel-like factor 6 | KLF6 | EG2 | 2 | 5 | 5 | 4 | 0,001479057 | 0,028294149 | 8,42E-05 | 0,002977773 |
| 209147_s_at | AB000888 | Hs.435122 | phosphatidic acid phosphatase type 2A | PPAP2A | EG2 | 2 | 5 | 4 | 5 | 0,001479057 | 0,028294149 | 8,42E-05 | 0,002977773 |

| | | | | | | | | | | | | | |
|---|---|---|---|---|---|---|---|---|---|---|---|---|---|
| 209193_at | M24779 | Hs.81170 | pim-1 oncogene | PIM1 | EG2 | 2 | 5 | 1 | 8 | 0,0014790570 | 0,028294149 | 8,42E-05 | 0,002977773 |
| 209581_at | BC001387 | Hs.502775 | HRAS-like suppressor 3 | HRASLS3 | EG2 | 4 | 3 | 6 | 3 | 0,0014790570 | 0,028294149 | 8,42E-05 | 0,002977773 |
| 209744_x_at | AB056663 | Hs.472509 | itchy homolog E3 ubiquitin protein ligase (mouse) | ITCH | EG2 | 4 | 3 | 6 | 3 | 0,0014790570 | 0,028294149 | 8,42E-05 | 0,002977773 |
| 209806_at | BC000893 | Hs.437275 | histone 1, H2bk | HIST1H2BK | EG2 | 4 | 3 | 3 | 6 | 0,0014790570 | 0,028294149 | 8,42E-05 | 0,002977773 |
| 209862_s_at | BC001233 | Hs.101014 | | PIG8 | EG2 | 5 | 2 | 5 | 4 | 0,0014790570 | 0,028294149 | 8,42E-05 | 0,002977773 |
| 212196_at | AW242916 | Hs.454699, H | interleukin 6 signal transducer (gp130, oncostatin M re | IL6ST | EG2 | 7 | 0 | 2 | 7 | 0,0014790570 | 0,028294149 | 8,42E-05 | 0,002977773 |
| 212730_at | AK026420 | Hs.207106 | desmuslin | DMN | EG2 | 4 | 3 | 5 | 4 | 0,0014790570 | 0,028294149 | 8,42E-05 | 0,002977773 |
| 212841_s_at | AI692180 | Hs.16019 | PTPRF interacting protein, binding protein 2 (liprin be | PPFIBP2 | EG2 | 4 | 3 | 2 | 7 | 0,0014790570 | 0,028294149 | 8,42E-05 | 0,002977773 |
| 216028_at | AL049980 | Hs.184216 | | DKFZP564C152 | EG2 | 4 | 3 | 5 | 4 | 0,0014790570 | 0,028294149 | 8,42E-05 | 0,002977773 |
| 218485_s_at | NM_018389 | Hs.12211 | solute carrier family 35, member C1 | SLC35C1 | EG2 | 1 | 6 | 3 | 6 | 0,0014790570 | 0,028294149 | 8,42E-05 | 0,002977773 |
| 218662_s_at | NM_022346 | Hs.446201, Hs.479270 | | HCAP-G | EG2 | 4 | 3 | 3 | 6 | 0,0014790570 | 0,028294149 | 8,42E-05 | 0,002977773 |
| 218795_at | NM_016361 | Hs.528084 | acid phosphatase 6, lysophosphatidic | ACP6 | EG2 | 0 | 7 | 3 | 6 | 0,0014790570 | 0,028294149 | 8,42E-05 | 0,002977773 |
| 218935_at | NM_014600 | Hs.368808 | EH-domain containing 3 | EHD3 | EG2 | 1 | 6 | 6 | 3 | 0,0014790570 | 0,028294149 | 8,42E-05 | 0,002977773 |
| 219000_s_at | NM_024094 | Hs.315167 | | DCC1 | EG2 | 3 | 4 | 3 | 6 | 0,0014790570 | 0,028294149 | 8,42E-05 | 0,002977773 |
| 219117_s_at | NM_016594 | Hs.438695 | FK506 binding protein 11, 19 kDa | FKBP11 | EG2 | 6 | 1 | 7 | 2 | 0,0014790570 | 0,028294149 | 8,42E-05 | 0,002977773 |
| 219148_at | NM_018492 | Hs.104741 | PDZ binding kinase | PBK | EG2 | 5 | 2 | 3 | 6 | 0,0014790570 | 0,028294149 | 8,42E-05 | 0,002977773 |
| 220940_at | NM_025190 | Hs.503490, H | KIAA1641 | KIAA1641 | EG2 | 3 | 4 | 5 | 4 | 0,0014790570 | 0,028294149 | 8,42E-05 | 0,002977773 |
| 222640_at | N26002 | Hs.515840 | DNA (cytosine-5-)-methyltransferase 3 alpha | DNMT3A | EG2 | 1 | 6 | 2 | 7 | 0,0014790570 | 0,028294149 | 8,42E-05 | 0,002977773 |
| 223222_at | BC001075 | Hs.514470 | solute carrier family 25 (mitochondrial deoxynucleoti | SLC25A19 | EG2 | 4 | 3 | 5 | 4 | 0,0014790570 | 0,028294149 | 8,42E-05 | 0,002977773 |
| 223319_at | AF272663 | Hs.208765 | gephyrin | GPHN | EG2 | 6 | 1 | 3 | 6 | 0,0014790570 | 0,028294149 | 8,42E-05 | 0,002977773 |
| 223464_at | AL136918 | Hs.436166 | oxysterol binding protein-like 5 | OSBPL5 | EG2 | 4 | 3 | 5 | 4 | 0,0014790570 | 0,028294149 | 8,42E-05 | 0,002977773 |
| 224836_at | AL109824 | Hs.516994 | tumor protein p53 inducible nuclear protein 2 | TP53INP2 | EG2 | 6 | 1 | 5 | 4 | 0,0014790570 | 0,028294149 | 8,42E-05 | 0,002977773 |
| 225129_at | AW170571 | Hs.339809 | copine II | CPNE2 | EG2 | 1 | 6 | 0 | 9 | 0,0014790570 | 0,028294149 | 8,42E-05 | 0,002977773 |
| 225613_at | AI096389 | Hs.133539 | microtubule associated serine/threonine kinase famil | MAST4 | EG2 | 3 | 4 | 2 | 7 | 0,0014790570 | 0,028294149 | 8,42E-05 | 0,002977773 |
| 226583_at | AL560962 | Hs.44817 | | FLJ40142 | EG2 | 3 | 4 | 4 | 5 | 0,0014790570 | 0,028294149 | 8,42E-05 | 0,002977773 |
| 226718_at | AA001423 | Hs.12264 | adhesion molecule with Ig-like domain 1 | AMIGO1 | EG2 | 1 | 6 | 0 | 9 | 0,0014790570 | 0,028294149 | 8,42E-05 | 0,002977773 |
| 228370_at | BF114870 | Hs.525700 | small nuclear ribonucleoprotein polypeptide N | SNRPN | EG2 | 7 | 0 | 5 | 4 | 0,0014790570 | 0,028294149 | 8,42E-05 | 0,002977773 |
| 228667_at | AI733330 | Hs.353175 | 1-acylglycerol-3-phosphate O-acyltransferase 4 (lysop | AGPAT4 | EG2 | 3 | 4 | 9 | 0 | 0,0014790570 | 0,028294149 | 8,42E-05 | 0,002977773 |
| 228829_at | AI279868 | Hs.12286 | activating transcription factor 7 | ATF7 | EG2 | 2 | 5 | 9 | 0 | 0,0014790570 | 0,028294149 | 8,42E-05 | 0,002977773 |
| 229189_s_at | BF672306 | | | | EG2 | 0 | 7 | 5 | 4 | 0,0014790570 | 0,028294149 | 8,42E-05 | 0,002977773 |
| 229193_at | AA005430 | Hs.130293, Hs.534387 | | CROP | EG2 | 6 | 1 | 6 | 3 | 0,0014790570 | 0,028294149 | 8,42E-05 | 0,002977773 |
| 229490_s_at | AW271106 | Hs.133294 | IQ motif containing GTPase activating protein 3 | IQGAP3 | EG2 | 4 | 3 | 2 | 7 | 0,0014790570 | 0,028294149 | 8,42E-05 | 0,002977773 |
| 229553_at | AA736452 | Hs.26612 | phosphoglucomutase 2-like 1 | PGM2L1 | EG2 | 5 | 2 | 2 | 7 | 0,0014790570 | 0,028294149 | 8,42E-05 | 0,002977773 |
| 229748_x_at | AI380156 | | | | EG2 | 1 | 6 | 5 | 4 | 0,0014790570 | 0,028294149 | 8,42E-05 | 0,002977773 |
| 230083_at | AW188464 | Hs.431081 | ubiquitin specific peptidase 53 | USP53 | EG2 | 5 | 2 | 2 | 7 | 0,0014790570 | 0,028294149 | 8,42E-05 | 0,002977773 |
| 230097_at | AI207338 | Hs.473648 | phosphoribosylglycinamide formyltransferase, phosp | GART | EG2 | 6 | 1 | 6 | 3 | 0,0014790570 | 0,028294149 | 8,42E-05 | 0,002977773 |
| 230300_at | N22849 | | | | EG2 | 4 | 3 | 6 | 3 | 0,0014790570 | 0,028294149 | 8,42E-05 | 0,002977773 |
| 230305_at | AW000995 | | | | EG2 | 6 | 1 | 5 | 4 | 0,0014790570 | 0,028294149 | 8,42E-05 | 0,002977773 |
| 230906_at | AK024931 | Hs.34421, Hs | UDP-N-acetyl-alpha-D-galactosamine:polypeptide N- | GALNT10 | EG2 | 4 | 3 | 8 | 1 | 0,0014790570 | 0,028294149 | 8,42E-05 | 0,002977773 |
| 231577_s_at | AW014593 | Hs.62661, Hs | guanylate binding protein 1, interferon-inducible, 67k | GBP1 | EG2 | 1 | 6 | 2 | 7 | 0,0014790570 | 0,028294149 | 8,42E-05 | 0,002977773 |
| 235113_at | AA742244 | Hs.556013 | peptidylprolyl isomerase (cyclophilin)-like 5 | PPIL5 | EG2 | 4 | 3 | 5 | 4 | 0,0014790570 | 0,028294149 | 8,42E-05 | 0,002977773 |
| 235315_at | AI809519 | Hs.450972, H | TSC22 domain family, member 1 | TSC22D1 | EG2 | 7 | 0 | 3 | 6 | 0,0014790570 | 0,028294149 | 8,42E-05 | 0,002977773 |
| 238902_at | T85248 | Hs.308480 | | LOC115294 | EG2 | 5 | 2 | 4 | 5 | 0,0014790570 | 0,028294149 | 8,42E-05 | 0,002977773 |
| 239208_s_at | AW945538 | | | | EG2 | 2 | 5 | 2 | 7 | 0,0014790570 | 0,028294149 | 8,42E-05 | 0,002977773 |
| 240165_at | AI678013 | | | | EG2 | 4 | 3 | 8 | 1 | 0,0014790570 | 0,028294149 | 8,42E-05 | 0,002977773 |

| | | | | | | | | | | | | | |
|---|---|---|---|---|---|---|---|---|---|---|---|---|---|
| 242907_at | BF509371 | Hs.386567 | guanylate binding protein 2, interferon-inducible | GBP2 | EG2 | 1 | 6 | 0 | 9 | 0,001479057 | 0,028294149 | 8,42E-05 | 0,002977773 |
| 52940_at | AA085764 | Hs.501624 | | SIGIRR | EG2 | 0 | 7 | 1 | 8 | 0,001479057 | 0,028294149 | 8,42E-05 | 0,002977773 |
| AFFX-r2-Bs-lys | AFFX-R2-BS-LYS-5 | | | | EG2 | 2 | 5 | 2 | 7 | 0,001479057 | 0,028294149 | 8,42E-05 | 0,002977773 |
| 209610_s_at | BF340083 | Hs.323878 | solute carrier family 1 (glutamate/neutral amino acid t | SLC1A4 | EG2 | 5 | 1 | 11 | 7 | 0,007867576 | 0,079901528 | 2,28E-17 | 0,001562433 |
| 207761_s_at | NM_014033 | Hs.288771 | | DKFZP586A0522 | EG2 | 3 | 3 | 10 | 5 | 0,007867576 | 0,079901528 | 7,74E-12 | 0,001562433 |
| 226142_at | AV682252 | Hs.553516 | GLI pathogenesis-related 1 (glioma) | GLIPR1 | EG2 | 5 | 1 | 7 | 8 | 0,007867576 | 0,079901528 | 7,74E-12 | 0,001562433 |
| 228708_at | BF438386 | Hs.25318 | RAB27B, member RAS oncogene family | RAB27B | EG2 | 3 | 3 | 11 | 4 | 0,007867576 | 0,079901528 | 7,74E-12 | 0,001562433 |
| 226225_at | BE967311 | Hs.483104 | mutated in colorectal cancers | MCC | EG2 | 3 | 3 | 3 | 11 | 0,007867576 | 0,079901528 | 2,17E-10 | 0,001562433 |
| 201487_at | NM_001814 | Hs.128065 | cathepsin C | CTSC | EG2 | 1 | 5 | 8 | 5 | 0,007867576 | 0,079901528 | 4,56E-09 | 0,001562433 |
| 204256_at | NM_024090 | Hs.412939 | ELOVL family member 6, elongation of long chain fatty | ELOVL6 | EG2 | 5 | 1 | 7 | 6 | 0,007867576 | 0,079901528 | 4,56E-09 | 0,001562433 |
| 204749_at | NM_004538 | Hs.21365 | nucleosome assembly protein 1-like 3 | NAP1L3 | EG2 | 5 | 1 | 9 | 4 | 0,007867576 | 0,079901528 | 4,56E-09 | 0,001562433 |
| 208926_at | U84246 | Hs.520037 | sialidase 1 (lysosomal sialidase) | NEU1 | EG2 | 4 | 2 | 11 | 2 | 0,007867576 | 0,079901528 | 4,56E-09 | 0,001562433 |
| 216236_s_at | AL110298 | Hs.210227, H | solute carrier family 2 (facilitated glucose transporter) | SLC2A3 | EG2 | 4 | 2 | 7 | 6 | 0,007867576 | 0,079901528 | 4,56E-09 | 0,001562433 |
| 217996_at | AA576961 | Hs.484885 | pleckstrin homology-like domain, family A, member 1 | PHLDA1 | EG2 | 4 | 2 | 8 | 5 | 0,007867576 | 0,079901528 | 4,56E-09 | 0,001562433 |
| 222862_s_at | BG169832 | Hs.18268 | adenylate kinase 5 | AK5 | EG2 | 4 | 2 | 6 | 7 | 0,007867576 | 0,079901528 | 4,56E-09 | 0,001562433 |
| 227224_at | AW003297 | Hs.496222 | Ral GEF with PH domain and SH3 binding motif 2 | RALGPS2 | EG2 | 4 | 2 | 3 | 10 | 0,007867576 | 0,079901528 | 4,56E-09 | 0,001562433 |
| 227486_at | AI086864 | Hs.153952 | 5'-nucleotidase, ecto (CD73) | NT5E | EG2 | 4 | 2 | 9 | 4 | 0,007867576 | 0,079901528 | 4,56E-09 | 0,001562433 |
| 228033_at | AI341146 | Hs.416375 | E2F transcription factor 7 | E2F7 | EG2 | 3 | 3 | 11 | 2 | 0,007867576 | 0,079901528 | 4,56E-09 | 0,001562433 |
| 239614_x_at | AW173003 | Hs.116448 | glutaminase | GLS | EG2 | 4 | 2 | 6 | 7 | 0,007867576 | 0,079901528 | 4,56E-09 | 0,001562433 |
| 242051_at | AI695695 | | | | EG2 | 3 | 3 | 6 | 7 | 0,007867576 | 0,079901528 | 4,56E-09 | 0,001562433 |
| 201341_at | NM_003633 | Hs.104925 | ectodermal-neural cortex (with BTB-like domain) | ENC1 | EG2 | 5 | 1 | 8 | 4 | 0,007867576 | 0,079901528 | 7,43E-08 | 0,001562433 |
| 201540_at | NM_001449 | Hs.435369 | four and a half LIM domains 1 | FHL1 | EG2 | 1 | 5 | 1 | 11 | 0,007867576 | 0,079901528 | 7,43E-08 | 0,001562433 |
| 201739_at | NM_005627 | Hs.296323 | serum/glucocorticoid regulated kinase | SGK | EG2 | 4 | 2 | 4 | 8 | 0,007867576 | 0,079901528 | 7,43E-08 | 0,001562433 |
| 202887_s_at | NM_019058 | Hs.523012 | DNA-damage-inducible transcript 4 | DDIT4 | EG2 | 0 | 6 | 4 | 8 | 0,007867576 | 0,079901528 | 7,43E-08 | 0,001562433 |
| 202912_at | NM_001124 | Hs.441047 | adrenomedullin | ADM | EG2 | 5 | 1 | 5 | 7 | 0,007867576 | 0,079901528 | 7,43E-08 | 0,001562433 |
| 203851_at | NM_002178 | Hs.274313 | insulin-like growth factor binding protein 6 | IGFBP6 | EG2 | 1 | 5 | 4 | 8 | 0,007867576 | 0,079901528 | 7,43E-08 | 0,001562433 |
| 204596_s_at | U46768 | Hs.25590 | stanniocalcin 1 | STC1 | EG2 | 3 | 3 | 10 | 2 | 0,007867576 | 0,079901528 | 7,43E-08 | 0,001562433 |
| 205803_s_at | NM_003304 | Hs.250687 | transient receptor potential cation channel, subfamily | TRPC1 | EG2 | 2 | 4 | 4 | 8 | 0,007867576 | 0,079901528 | 7,43E-08 | 0,001562433 |
| 209383_at | BC003637 | Hs.505777 | DNA-damage-inducible transcript 3 | DDIT3 | EG2 | 5 | 1 | 10 | 2 | 0,007867576 | 0,079901528 | 7,43E-08 | 0,001562433 |
| 217967_s_at | AF288391 | Hs.518662 | chromosome 1 open reading frame 24 | C1orf24 | EG2 | 2 | 4 | 6 | 6 | 0,007867576 | 0,079901528 | 7,43E-08 | 0,001562433 |
| 219118_at | NM_016594 | Hs.438695 | FK506 binding protein 11, 19 kDa | FKBP11 | EG2 | 3 | 3 | 8 | 4 | 0,007867576 | 0,079901528 | 7,43E-08 | 0,001562433 |
| 221973_at | AI983904 | | | | EG2 | 4 | 2 | 7 | 5 | 0,007867576 | 0,079901528 | 7,43E-08 | 0,001562433 |
| 221974_at | AW770748 | Hs.525700 | small nuclear ribonucleoprotein polypeptide N | SNRPN | EG2 | 5 | 1 | 6 | 6 | 0,007867576 | 0,079901528 | 7,43E-08 | 0,001562433 |
| 222931_s_at | AI809864 | Hs.555980 | threonine synthase-like 1 (bacterial) | THNSL1 | EG2 | 3 | 3 | 6 | 6 | 0,007867576 | 0,079901528 | 7,43E-08 | 0,001562433 |
| 223196_s_at | AL136551 | Hs.469543 | sestrin 2 | SESN2 | EG2 | 5 | 1 | 10 | 2 | 0,007867576 | 0,079901528 | 7,43E-08 | 0,001562433 |
| 225496_s_at | N21426 | Hs.369520 | synaptotagmin-like 2 | SYTL2 | EG2 | 2 | 4 | 7 | 5 | 0,007867576 | 0,079901528 | 7,43E-08 | 0,001562433 |
| 227326_at | BE966768 | Hs.250723, H | matrix-remodelling associated 7 | MXRA7 | EG2 | 3 | 3 | 8 | 4 | 0,007867576 | 0,079901528 | 7,43E-08 | 0,001562433 |
| 227345_at | AI738556 | Hs.213467 | tumor necrosis factor receptor superfamily, member 1 | TNFRSF10D | EG2 | 4 | 2 | 5 | 7 | 0,007867576 | 0,079901528 | 7,43E-08 | 0,001562433 |
| 228754_at | BG150485 | Hs.517819 | glutamate receptor interacting protein 2 | GRIP2 | EG2 | 3 | 3 | 9 | 3 | 0,007867576 | 0,079901528 | 7,43E-08 | 0,001562433 |
| 228774_at | AW268594 | Hs.374421 | chromosome 9 open reading frame 81 | C9orf81 | EG2 | 4 | 2 | 10 | 2 | 0,007867576 | 0,079901528 | 7,43E-08 | 0,001562433 |
| 231798_at | AL575177 | Hs.248201 | noggin | NOG | EG2 | 1 | 5 | 6 | 6 | 0,007867576 | 0,079901528 | 7,43E-08 | 0,001562433 |
| 232344_at | AK021812 | Hs.553501 | RAS p21 protein activator (GTPase activating protein) 1 | RASA1 | EG2 | 2 | 4 | 5 | 7 | 0,007867576 | 0,079901528 | 7,43E-08 | 0,001562433 |
| 242751_at | N55072 | Hs.120 | peroxiredoxin 6 | PRDX6 | EG2 | 3 | 3 | 12 | 0 | 0,007867576 | 0,079901528 | 7,43E-08 | 0,001562433 |
| 1552622_s_at | BQ613856 | Hs.535030, Hs.556000 | | MGC13098 | EG2 | 2 | 4 | 4 | 7 | 0,007867576 | 0,079901528 | 9,59E-07 | 0,001562433 |
| 202600_s_at | AI824012 | Hs.155017 | nuclear receptor interacting protein 1 | NRIP1 | EG2 | 5 | 1 | 1 | 10 | 0,007867576 | 0,079901528 | 9,59E-07 | 0,001562433 |

| | | | | | | | | | | | | |
|---|---|---|---|---|---|---|---|---|---|---|---|---|
| 203989_x_at | NM_001992 | Hs.482562 | coagulation factor II (thrombin) receptor | F2R | EG2 | 1 | 5 | 6 | 5 | 0,0078675760,079901528 | 9,59E-07 | 0,001562433 |
| 204497_at | AB011092 | Hs.391860 | adenylate cyclase 9 | ADCY9 | EG2 | 1 | 5 | 4 | 7 | 0,0078675760,079901528 | 9,59E-07 | 0,001562433 |
| 204620_s_at | NM_004385 | Hs.443681 | chondroitin sulfate proteoglycan 2 (versican) | CSPG2 | EG2 | 3 | 3 | 6 | 5 | 0,0078675760,079901528 | 9,59E-07 | 0,001562433 |
| 204863_s_at | BE856546 | Hs.454699, H | interleukin 6 signal transducer (gp130, oncostatin M re | IL6ST | EG2 | 6 | 0 | 2 | 9 | 0,0078675760,079901528 | 9,59E-07 | 0,001562433 |
| 205771_s_at | AL137063 | Hs.486483 | A kinase (PRKA) anchor protein 7 | AKAP7 | EG2 | 4 | 2 | 3 | 8 | 0,0078675760,079901528 | 9,59E-07 | 0,001562433 |
| 206744_s_at | NM_014242 | Hs.530988 | zinc finger, MYM-type 5 | ZMYM5 | EG2 | 1 | 5 | 7 | 4 | 0,0078675760,079901528 | 9,59E-07 | 0,001562433 |
| 208003_s_at | NM_006599 | Hs.371987 | nuclear factor of activated T-cells 5, tonicity-responsiv | NFAT5 | EG2 | 2 | 4 | 5 | 6 | 0,0078675760,079901528 | 9,59E-07 | 0,001562433 |
| 208998_at | U94592 | Hs.80658 | uncoupling protein 2 (mitochondrial, proton carrier) | UCP2 | EG2 | 3 | 3 | 4 | 7 | 0,0078675760,079901528 | 9,59E-07 | 0,001562433 |
| 209706_at | AF247704 | Hs.55999 | NK3 transcription factor related, locus 1 (Drosophila) | NKX3-1 | EG2 | 5 | 1 | 5 | 6 | 0,0078675760,079901528 | 9,59E-07 | 0,001562433 |
| 212624_s_at | BF339445 | Hs.380138 | chimerin (chimaerin) 1 | CHN1 | EG2 | 3 | 3 | 3 | 8 | 0,0078675760,079901528 | 9,59E-07 | 0,001562433 |
| 213506_at | BE965369 | Hs.154299 | coagulation factor II (thrombin) receptor-like 1 | F2RL1 | EG2 | 0 | 6 | 2 | 9 | 0,0078675760,079901528 | 9,59E-07 | 0,001562433 |
| 214196_s_at | AA602532 | Hs.523454 | tripeptidyl peptidase I | TPP1 | EG2 | 5 | 1 | 10 | 1 | 0,0078675760,079901528 | 9,59E-07 | 0,001562433 |
| 219587_at | NM_017868 | Hs.288772 | tetratricopeptide repeat domain 12 | TTC12 | EG2 | 3 | 3 | 4 | 7 | 0,0078675760,079901528 | 9,59E-07 | 0,001562433 |
| 221731_x_at | BF218922 | Hs.443681 | chondroitin sulfate proteoglycan 2 (versican) | CSPG2 | EG2 | 3 | 3 | 6 | 5 | 0,0078675760,079901528 | 9,59E-07 | 0,001562433 |
| 223125_s_at | AL563236 | Hs.497159, H | chromosome 1 open reading frame 21 | C1orf21 | EG2 | 4 | 2 | 4 | 7 | 0,0078675760,079901528 | 9,59E-07 | 0,001562433 |
| 223199_at | AA404592 | Hs.515032 | MAP kinase interacting serine/threonine kinase 2 | MKNK2 | EG2 | 5 | 1 | 6 | 5 | 0,0078675760,079901528 | 9,59E-07 | 0,001562433 |
| 223484_at | AF228422 | Hs.112242 | | NMES1 | EG2 | 2 | 4 | 6 | 5 | 0,0078675760,079901528 | 9,59E-07 | 0,001562433 |
| 225081_s_at | AK022955 | Hs.520245 | cell division cycle associated 7-like | CDCA7L | EG2 | 3 | 3 | 5 | 6 | 0,0078675760,079901528 | 9,59E-07 | 0,001562433 |
| 225421_at | AI654133 | Hs.356247 | aminoacylase 1-like 2 | ACY1L2 | EG2 | 4 | 2 | 2 | 9 | 0,0078675760,079901528 | 9,59E-07 | 0,001562433 |
| 226085_at | AA181060 | Hs.349283 | chromobox homolog 5 (HP1 alpha homolog, Drosophil | CBX5 | EG2 | 4 | 2 | 3 | 8 | 0,0078675760,079901528 | 9,59E-07 | 0,001562433 |
| 226707_at | AI925574 | Hs.493164 | nicotinate phosphoribosyltransferase domain contain | NAPRT1 | EG2 | 0 | 6 | 3 | 8 | 0,0078675760,079901528 | 9,59E-07 | 0,001562433 |
| 227917_at | AW192692 | | | | EG2 | 0 | 6 | 2 | 9 | 0,0078675760,079901528 | 9,59E-07 | 0,001562433 |
| 228497_at | AI279062 | Hs.125482 | solute carrier family 22 (organic cation transporter), m | SLC22A15 | EG2 | 1 | 5 | 10 | 1 | 0,0078675760,079901528 | 9,59E-07 | 0,001562433 |
| 229515_at | AA632147 | Hs.406074 | PRKC, apoptosis, WT1, regulator | PAWR | EG2 | 5 | 1 | 4 | 7 | 0,0078675760,079901528 | 9,59E-07 | 0,001562433 |
| 235028_at | BG288330 | | | | EG2 | 5 | 1 | 6 | 5 | 0,0078675760,079901528 | 9,59E-07 | 0,001562433 |
| 235106_at | AW847318 | Hs.428214 | mastermind-like 2 (Drosophila) | MAML2 | EG2 | 4 | 2 | 1 | 10 | 0,0078675760,079901528 | 9,59E-07 | 0,001562433 |
| 238021_s_at | AA954994 | Hs.237396 | | LOC388279 | EG2 | 5 | 1 | 7 | 4 | 0,0078675760,079901528 | 9,59E-07 | 0,001562433 |
| 240690_at | AI494291 | Hs.29068 | | DKFZp761P0423 | EG2 | 3 | 3 | 5 | 6 | 0,0078675760,079901528 | 9,59E-07 | 0,001562433 |
| 242467_at | BF433200 | Hs.529862 | casein kinase 1, alpha 1 | CSNK1A1 | EG2 | 5 | 1 | 8 | 3 | 0,0078675760,079901528 | 9,59E-07 | 0,001562433 |
| 1552733_at | NM_172193 | Hs.509258 | kelch domain containing 1 | KLHDC1 | EG2 | 4 | 2 | 5 | 5 | 0,0078675760,079901528 | 9,97E-06 | 0,001562433 |
| 1557270_at | AA632049 | | | | EG2 | 6 | 0 | 3 | 7 | 0,0078675760,079901528 | 9,97E-06 | 0,001562433 |
| 1564796_at | BC017854 | Hs.436298 | epithelial membrane protein 1 | EMP1 | EG2 | 3 | 3 | 8 | 2 | 0,0078675760,079901528 | 9,97E-06 | 0,001562433 |
| 1569312_at | BE383308 | Hs.301819 | zinc finger protein 146 | ZNF146 | EG2 | 5 | 1 | 7 | 3 | 0,0078675760,079901528 | 9,97E-06 | 0,001562433 |
| 201422_at | NM_006332 | Hs.14623 | interferon, gamma-inducible protein 30 | IFI30 | EG2 | 1 | 5 | 8 | 2 | 0,0078675760,079901528 | 9,97E-06 | 0,001562433 |
| 201427_s_at | NM_005410 | Hs.275775 | selenoprotein P, plasma, 1 | SEPP1 | EG2 | 4 | 2 | 6 | 4 | 0,0078675760,079901528 | 9,97E-06 | 0,001562433 |
| 202497_x_at | AI631159 | Hs.210227, H | solute carrier family 2 (facilitated glucose transporter) | SLC2A3 | EG2 | 4 | 2 | 6 | 4 | 0,0078675760,079901528 | 9,97E-06 | 0,001562433 |
| 202498_s_at | BE550486 | Hs.210227, H | solute carrier family 2 (facilitated glucose transporter) | SLC2A3 | EG2 | 4 | 2 | 5 | 5 | 0,0078675760,079901528 | 9,97E-06 | 0,001562433 |
| 203408_s_at | NM_002971 | Hs.517717 | special AT-rich sequence binding protein 1 (binds to n | SATB1 | EG2 | 2 | 4 | 4 | 6 | 0,0078675760,079901528 | 9,97E-06 | 0,001562433 |
| 203946_s_at | U75667 | Hs.553485 | arginase, type II | ARG2 | EG2 | 5 | 1 | 6 | 4 | 0,0078675760,079901528 | 9,97E-06 | 0,001562433 |
| 204783_at | AI911434 | Hs.85195 | myeloid leukemia factor 1 | MLF1 | EG2 | 5 | 1 | 7 | 3 | 0,0078675760,079901528 | 9,97E-06 | 0,001562433 |
| 205128_x_at | NM_000962 | Hs.201978 | prostaglandin-endoperoxide synthase 1 (prostaglandi | PTGS1 | EG2 | 4 | 2 | 6 | 4 | 0,0078675760,079901528 | 9,97E-06 | 0,001562433 |
| 205756_s_at | NM_000132 | Hs.413083 | coagulation factor VIII, procoagulant component (hem | F8 | EG2 | 2 | 4 | 5 | 5 | 0,0078675760,079901528 | 9,97E-06 | 0,001562433 |
| 207231_at | NM_014648 | Hs.409210 | | DZIP3 | EG2 | 2 | 4 | 3 | 7 | 0,0078675760,079901528 | 9,97E-06 | 0,001562433 |
| 207813_s_at | NM_004110 | Hs.69745 | ferredoxin reductase | FDXR | EG2 | 1 | 5 | 4 | 6 | 0,0078675760,079901528 | 9,97E-06 | 0,001562433 |
| 207980_s_at | NM_006079 | Hs.82071 | Cbp/p300-interacting transactivator, with Glu/Asp-rich | CITED2 | EG2 | 1 | 5 | 3 | 7 | 0,0078675760,079901528 | 9,97E-06 | 0,001562433 |

| | | | | | | | | | | | | | |
|---|---|---|---|---|---|---|---|---|---|---|---|---|---|
| 208190_s_at | NM_015925 | Hs.466507 | | LISCH7 | EG2 | 3 | 3 | 2 | 8 | 0,007867576 | 0,079901528 | 9,97E-06 | 0,001562433 |
| 209754_s_at | AF113682 | Hs.11355 | thymopoietin | TMPO | EG2 | 3 | 3 | 3 | 7 | 0,007867576 | 0,079901528 | 9,97E-06 | 0,001562433 |
| 209969_s_at | BC002704 | Hs.470943 | signal transducer and activator of transcription 1, 91kD | STAT1 | EG2 | 1 | 5 | 3 | 7 | 0,007867576 | 0,079901528 | 9,97E-06 | 0,001562433 |
| 210538_s_at | U37546 | Hs.127799 | baculoviral IAP repeat-containing 3 | BIRC3 | EG2 | 6 | 0 | 3 | 7 | 0,007867576 | 0,079901528 | 9,97E-06 | 0,001562433 |
| 211085_s_at | Z25430 | Hs.472838 | serine/threonine kinase 4 | STK4 | EG2 | 1 | 5 | 3 | 7 | 0,007867576 | 0,079901528 | 9,97E-06 | 0,001562433 |
| 214085_x_at | AI912583 | Hs.205558 | HIV-1 rev binding protein 2 | HRB2 | EG2 | 6 | 0 | 5 | 5 | 0,007867576 | 0,079901528 | 9,97E-06 | 0,001562433 |
| 214252_s_at | AV700514 | Hs.30213 | ceroid-lipofuscinosis, neuronal 5 | CLN5 | EG2 | 3 | 3 | 4 | 6 | 0,007867576 | 0,079901528 | 9,97E-06 | 0,001562433 |
| 215646_s_at | R94644 | Hs.443681 | chondroitin sulfate proteoglycan 2 (versican) | CSPG2 | EG2 | 4 | 2 | 5 | 5 | 0,007867576 | 0,079901528 | 9,97E-06 | 0,001562433 |
| 216870_x_at | AF264787 | Hs.508041 | deleted in lymphocytic leukemia, 2 | DLEU2 | EG2 | 5 | 1 | 4 | 6 | 0,007867576 | 0,079901528 | 9,97E-06 | 0,001562433 |
| 217127_at | AL354872 | Hs.19904 | cystathionase (cystathionine gamma-lyase) | CTH | EG2 | 6 | 0 | 7 | 3 | 0,007867576 | 0,079901528 | 9,97E-06 | 0,001562433 |
| 217165_x_at | M10943 | Hs.513626 | metallothionein 1F (functional) | MT1F | EG2 | 2 | 4 | 5 | 5 | 0,007867576 | 0,079901528 | 9,97E-06 | 0,001562433 |
| 217208_s_at | AL121981 | Hs.292549 | discs, large homolog 1 (Drosophila) | DLG1 | EG2 | 4 | 2 | 6 | 4 | 0,007867576 | 0,079901528 | 9,97E-06 | 0,001562433 |
| 218701_at | NM_016027 | Hs.118554 | lactamase, beta 2 | LACTB2 | EG2 | 3 | 3 | 4 | 6 | 0,007867576 | 0,079901528 | 9,97E-06 | 0,001562433 |
| 218726_at | NM_018410 | Hs.532968 | | DKFZp762E1312 | EG2 | 5 | 1 | 3 | 7 | 0,007867576 | 0,079901528 | 9,97E-06 | 0,001562433 |
| 219209_at | NM_022168 | Hs.163173 | interferon induced with helicase C domain 1 | IFIH1 | EG2 | 2 | 4 | 2 | 8 | 0,007867576 | 0,079901528 | 9,97E-06 | 0,001562433 |
| 219250_s_at | NM_013281 | Hs.41296 | fibronectin leucine rich transmembrane protein 3 | FLRT3 | EG2 | 0 | 6 | 4 | 6 | 0,007867576 | 0,079901528 | 9,97E-06 | 0,001562433 |
| 219493_at | NM_024745 | Hs.123253 | SHC SH2-domain binding protein 1 | SHCBP1 | EG2 | 5 | 1 | 3 | 7 | 0,007867576 | 0,079901528 | 9,97E-06 | 0,001562433 |
| 219684_at | NM_022147 | Hs.43388 | | IFRG28 | EG2 | 0 | 6 | 0 | 10 | 0,007867576 | 0,079901528 | 9,97E-06 | 0,001562433 |
| 221207_s_at | NM_015678 | Hs.491172 | neurobeachin | NBEA | EG2 | 4 | 2 | 2 | 8 | 0,007867576 | 0,079901528 | 9,97E-06 | 0,001562433 |
| 221922_at | AW195581 | Hs.489353 | G-protein signalling modulator 2 (AGS3-like, C. elegan | GPSM2 | EG2 | 3 | 3 | 4 | 6 | 0,007867576 | 0,079901528 | 9,97E-06 | 0,001562433 |
| 222787_s_at | AV705186 | Hs.396358 | | FLJ11273 | EG2 | 2 | 4 | 2 | 8 | 0,007867576 | 0,079901528 | 9,97E-06 | 0,001562433 |
| 223681_s_at | AB044807 | Hs.478125 | InaD-like (Drosophila) | INADL | EG2 | 2 | 4 | 0 | 10 | 0,007867576 | 0,079901528 | 9,97E-06 | 0,001562433 |
| 225626_at | AK000680 | Hs.266175, H | phosphoprotein associated with glycosphingolipid mi | PAG1 | EG2 | 4 | 2 | 6 | 4 | 0,007867576 | 0,079901528 | 9,97E-06 | 0,001562433 |
| 226218_at | BE217880 | Hs.362807 | interleukin 7 receptor | IL7R | EG2 | 4 | 2 | 4 | 6 | 0,007867576 | 0,079901528 | 9,97E-06 | 0,001562433 |
| 226239_at | AL573722 | Hs.356187 | | FLJ90024 | EG2 | 0 | 6 | 8 | 2 | 0,007867576 | 0,079901528 | 9,97E-06 | 0,001562433 |
| 226939_at | AI202327 | Hs.374216 | cytoplasmic polyadenylation element binding protein | CPEB2 | EG2 | 6 | 0 | 10 | 0 | 0,007867576 | 0,079901528 | 9,97E-06 | 0,001562433 |
| 227124_at | AI870473 | | | | EG2 | 6 | 0 | 8 | 2 | 0,007867576 | 0,079901528 | 9,97E-06 | 0,001562433 |
| 227156_at | AK025872 | Hs.3327 | trinucleotide repeat containing 8 | TNRC8 | EG2 | 4 | 2 | 1 | 9 | 0,007867576 | 0,079901528 | 9,97E-06 | 0,001562433 |
| 227458_at | AI608902 | Hs.521989 | CD274 antigen | CD274 | EG2 | 4 | 2 | 9 | 1 | 0,007867576 | 0,079901528 | 9,97E-06 | 0,001562433 |
| 227462_at | BE889628 | Hs.482910 | | LRAP | EG2 | 3 | 3 | 3 | 7 | 0,007867576 | 0,079901528 | 9,97E-06 | 0,001562433 |
| 228171_s_at | AI056683 | Hs.188781 | pleckstrin homology domain containing, family G (wit | PLEKHG4 | EG2 | 2 | 4 | 4 | 6 | 0,007867576 | 0,079901528 | 9,97E-06 | 0,001562433 |
| 228480_at | AW296039 | Hs.165195 | VAMP (vesicle-associated membrane protein)-associa | VAPA | EG2 | 1 | 5 | 5 | 5 | 0,007867576 | 0,079901528 | 9,97E-06 | 0,001562433 |
| 232235_at | AK021539 | Hs.124673 | chromosome 18 open reading frame 4 | C18orf4 | EG2 | 4 | 2 | 2 | 8 | 0,007867576 | 0,079901528 | 9,97E-06 | 0,001562433 |
| 234994_at | AA088177 | Hs.172870 | KIAA1913 | KIAA1913 | EG2 | 3 | 3 | 5 | 5 | 0,007867576 | 0,079901528 | 9,97E-06 | 0,001562433 |
| 235274_at | AA740632 | | | | EG2 | 5 | 1 | 3 | 7 | 0,007867576 | 0,079901528 | 9,97E-06 | 0,001562433 |
| 235547_at | BG548427 | Hs.507680 | | PFAAP5 | EG2 | 5 | 1 | 5 | 5 | 0,007867576 | 0,079901528 | 9,97E-06 | 0,001562433 |
| 236046_at | AA781880 | Hs.293833 | | FLJ44896 | EG2 | 2 | 4 | 4 | 6 | 0,007867576 | 0,079901528 | 9,97E-06 | 0,001562433 |
| 238458_at | AI868167 | Hs.403594 | EF-hand domain family, member A2 | EFHA2 | EG2 | 2 | 4 | 3 | 7 | 0,007867576 | 0,079901528 | 9,97E-06 | 0,001562433 |
| 238587_at | AI927919 | Hs.444075 | | STS-1 | EG2 | 3 | 3 | 4 | 6 | 0,007867576 | 0,079901528 | 9,97E-06 | 0,001562433 |
| 239413_at | AI130715 | Hs.443005 | | Cep152 | EG2 | 6 | 0 | 5 | 5 | 0,007867576 | 0,079901528 | 9,97E-06 | 0,001562433 |
| 239866_at | AA705933 | | | | EG2 | 2 | 4 | 4 | 6 | 0,007867576 | 0,079901528 | 9,97E-06 | 0,001562433 |
| 240146_at | AW418562 | Hs.446123 | capping protein (actin filament) muscle Z-line, alpha 2 | CAPZA2 | EG2 | 4 | 2 | 6 | 4 | 0,007867576 | 0,079901528 | 9,97E-06 | 0,001562433 |
| 240307_at | N54783 | Hs.269099 | far upstream element (FUSE) binding protein 1 | FUBP1 | EG2 | 2 | 4 | 6 | 4 | 0,007867576 | 0,079901528 | 9,97E-06 | 0,001562433 |
| 241879_at | AW511222 | | | | EG2 | 6 | 0 | 5 | 5 | 0,007867576 | 0,079901528 | 9,97E-06 | 0,001562433 |
| 1557081_at | AA580691 | Hs.531106 | RNA binding motif protein 25 | RBM25 | EG2 | 3 | 3 | 4 | 5 | 0,007867576 | 0,079901528 | 8,42E-05 | 0,002977773 |

| | | | | | | | | | | | | | |
|---|---|---|---|---|---|---|---|---|---|---|---|---|---|
| 1558522_at | BC013077 | Hs.533355 | membrane protein, palmitoylated 6 (MAGUK p55 subf | MPP6 | EG2 | 4 | 2 | 4 | 5 | 0,007867576 | 0,079901528 | 8,42E-05 | 0,002977773 |
| 200897_s_at | NM_016081 | Hs.151220 | | KIAA0992 | EG2 | 4 | 2 | 5 | 4 | 0,007867576 | 0,079901528 | 8,42E-05 | 0,002977773 |
| 201389_at | NM_002205 | Hs.505654 | integrin, alpha 5 (fibronectin receptor, alpha polypept | ITGA5 | EG2 | 3 | 3 | 5 | 4 | 0,007867576 | 0,079901528 | 8,42E-05 | 0,002977773 |
| 201810_s_at | AL562152 | Hs.257761 | SH3-domain binding protein 5 (BTK-associated) | SH3BP5 | EG2 | 0 | 6 | 5 | 4 | 0,007867576 | 0,079901528 | 8,42E-05 | 0,002977773 |
| 202284_s_at | NM_000389 | Hs.370771 | cyclin-dependent kinase inhibitor 1A (p21, Cip1) | CDKN1A | EG2 | 6 | 0 | 6 | 3 | 0,007867576 | 0,079901528 | 8,42E-05 | 0,002977773 |
| 202409_at | X07868 | Hs.523414, Hs.556244 | | LOC492304 | EG2 | 2 | 4 | 2 | 7 | 0,007867576 | 0,079901528 | 8,42E-05 | 0,002977773 |
| 202870_s_at | NM_001255 | Hs.524947 | CDC20 cell division cycle 20 homolog (S. cerevisiae) | CDC20 | EG2 | 3 | 3 | 3 | 6 | 0,007867576 | 0,079901528 | 8,42E-05 | 0,002977773 |
| 202973_x_at | NM_014883 | Hs.97270, Hs | family with sequence similarity 13, member A1 | FAM13A1 | EG2 | 2 | 4 | 5 | 4 | 0,007867576 | 0,079901528 | 8,42E-05 | 0,002977773 |
| 203016_s_at | AK001710 | Hs.22587 | synovial sarcoma, X breakpoint 2 interacting protein | SSX2IP | EG2 | 5 | 1 | 7 | 2 | 0,007867576 | 0,079901528 | 8,42E-05 | 0,002977773 |
| 203603_s_at | NM_014795 | Hs.34871 | zinc finger homeobox 1b | ZFHX1B | EG2 | 5 | 1 | 6 | 3 | 0,007867576 | 0,079901528 | 8,42E-05 | 0,002977773 |
| 203680_at | NM_002736 | Hs.433068 | protein kinase, cAMP-dependent, regulatory, type II, | PRKAR2B | EG2 | 5 | 1 | 6 | 3 | 0,007867576 | 0,079901528 | 8,42E-05 | 0,002977773 |
| 203867_s_at | NM_018096 | Hs.85570 | notchless homolog 1 (Drosophila) | NLE1 | EG2 | 2 | 4 | 6 | 3 | 0,007867576 | 0,079901528 | 8,42E-05 | 0,002977773 |
| 204015_s_at | BC002671 | Hs.417962 | dual specificity phosphatase 4 | DUSP4 | EG2 | 2 | 4 | 3 | 6 | 0,007867576 | 0,079901528 | 8,42E-05 | 0,002977773 |
| 204066_s_at | NM_014914 | Hs.435039 | centaurin, gamma 2 | CENTG2 | EG2 | 1 | 5 | 6 | 3 | 0,007867576 | 0,079901528 | 8,42E-05 | 0,002977773 |
| 204506_at | AL544951 | Hs.280604 | protein phosphatase 3 (formerly 2B), regulatory subu | PPP3R1 | EG2 | 5 | 1 | 2 | 7 | 0,007867576 | 0,079901528 | 8,42E-05 | 0,002977773 |
| 204686_at | NM_005544 | Hs.471508 | insulin receptor substrate 1 | IRS1 | EG2 | 3 | 3 | 3 | 6 | 0,007867576 | 0,079901528 | 8,42E-05 | 0,002977773 |
| 204796_at | AI825937 | Hs.12451 | echinoderm microtubule associated protein like 1 | EML1 | EG2 | 3 | 3 | 5 | 4 | 0,007867576 | 0,079901528 | 8,42E-05 | 0,002977773 |
| 204900_x_at | NM_003864 | Hs.413835 | sin3-associated polypeptide, 30kDa | SAP30 | EG2 | 3 | 3 | 6 | 3 | 0,007867576 | 0,079901528 | 8,42E-05 | 0,002977773 |
| 205259_at | NM_000901 | Hs.163924 | nuclear receptor subfamily 3, group C, member 2 | NR3C2 | EG2 | 3 | 3 | 2 | 7 | 0,007867576 | 0,079901528 | 8,42E-05 | 0,002977773 |
| 205398_s_at | NM_005902 | Hs.36915, Hs | SMAD, mothers against DPP homolog 3 (Drosophila) | SMAD3 | EG2 | 1 | 5 | 5 | 4 | 0,007867576 | 0,079901528 | 8,42E-05 | 0,002977773 |
| 205802_at | NM_003304 | Hs.250687 | transient receptor potential cation channel, subfamily | TRPC1 | EG2 | 3 | 3 | 2 | 7 | 0,007867576 | 0,079901528 | 8,42E-05 | 0,002977773 |
| 207029_at | NM_000899 | Hs.1048 | KIT ligand | KITLG | EG2 | 5 | 1 | 4 | 5 | 0,007867576 | 0,079901528 | 8,42E-05 | 0,002977773 |
| 207417_s_at | NM_003451 | Hs.172979 | zinc finger protein 177 | ZNF177 | EG2 | 1 | 5 | 4 | 5 | 0,007867576 | 0,079901528 | 8,42E-05 | 0,002977773 |
| 207426_s_at | NM_003326 | Hs.181097 | tumor necrosis factor (ligand) superfamily, member 4 | TNFSF4 | EG2 | 2 | 4 | 6 | 3 | 0,007867576 | 0,079901528 | 8,42E-05 | 0,002977773 |
| 208534_s_at | NM_006989 | Hs.520599 | RAS p21 protein activator 4 | RASA4 | EG2 | 1 | 5 | 4 | 5 | 0,007867576 | 0,079901528 | 8,42E-05 | 0,002977773 |
| 208663_s_at | AI652848 | Hs.368214 | tetratricopeptide repeat domain 3 | TTC3 | EG2 | 2 | 4 | 6 | 3 | 0,007867576 | 0,079901528 | 8,42E-05 | 0,002977773 |
| 208710_s_at | AI424923 | Hs.512815 | adaptor-related protein complex 3, delta 1 subunit | AP3D1 | EG2 | 0 | 6 | 3 | 6 | 0,007867576 | 0,079901528 | 8,42E-05 | 0,002977773 |
| 209278_s_at | L27624 | Hs.438231 | tissue factor pathway inhibitor 2 | TFPI2 | EG2 | 2 | 4 | 4 | 5 | 0,007867576 | 0,079901528 | 8,42E-05 | 0,002977773 |
| 209714_s_at | AF213033 | Hs.84113 | cyclin-dependent kinase inhibitor 3 (CDK2-associated | CDKN3 | EG2 | 4 | 2 | 3 | 6 | 0,007867576 | 0,079901528 | 8,42E-05 | 0,002977773 |
| 209782_s_at | U79283 | Hs.414480 | D site of albumin promoter (albumin D-box) binding p | DBP | EG2 | 0 | 6 | 0 | 9 | 0,007867576 | 0,079901528 | 8,42E-05 | 0,002977773 |
| 210785_s_at | AB035482 | Hs.10649 | chromosome 1 open reading frame 38 | C1orf38 | EG2 | 2 | 4 | 6 | 3 | 0,007867576 | 0,079901528 | 8,42E-05 | 0,002977773 |
| 211115_x_at | AB037703 | Hs.533862 | survival of motor neuron protein interacting protein 1 | SIP1 | EG2 | 5 | 1 | 6 | 3 | 0,007867576 | 0,079901528 | 8,42E-05 | 0,002977773 |
| 211302_s_at | L20966 | Hs.198072 | phosphodiesterase 4B, cAMP-specific (phosphodieste | PDE4B | EG2 | 4 | 2 | 5 | 4 | 0,007867576 | 0,079901528 | 8,42E-05 | 0,002977773 |
| 211343_s_at | M33653 | Hs.211933 | collagen, type XIII, alpha 1 | COL13A1 | EG2 | 3 | 3 | 6 | 3 | 0,007867576 | 0,079901528 | 8,42E-05 | 0,002977773 |
| 211596_s_at | AB050468 | Hs.518055 | leucine-rich repeats and immunoglobulin-like domain | LRIG1 | EG2 | 1 | 5 | 2 | 7 | 0,007867576 | 0,079901528 | 8,42E-05 | 0,002977773 |
| 211602_s_at | U31110 | Hs.250687 | transient receptor potential cation channel, subfamily | TRPC1 | EG2 | 4 | 2 | 5 | 4 | 0,007867576 | 0,079901528 | 8,42E-05 | 0,002977773 |
| 211653_x_at | M33376 | Hs.460260, H | aldo-keto reductase family 1, member C2 (dihydrodiol | AKR1C2 | EG2 | 3 | 3 | 5 | 4 | 0,007867576 | 0,079901528 | 8,42E-05 | 0,002977773 |
| 212022_s_at | BF001806 | Hs.80976 | antigen identified by monoclonal antibody Ki-67 | MKI67 | EG2 | 4 | 2 | 3 | 6 | 0,007867576 | 0,079901528 | 8,42E-05 | 0,002977773 |
| 212543_at | U83115 | Hs.486074 | absent in melanoma 1 | AIM1 | EG2 | 4 | 2 | 3 | 6 | 0,007867576 | 0,079901528 | 8,42E-05 | 0,002977773 |
| 212758_s_at | AI373166 | Hs.124503 | transcription factor 8 (represses interleukin 2 expressi | TCF8 | EG2 | 4 | 2 | 2 | 7 | 0,007867576 | 0,079901528 | 8,42E-05 | 0,002977773 |
| 212812_at | AI700633 | | | | EG2 | 5 | 1 | 2 | 7 | 0,007867576 | 0,079901528 | 8,42E-05 | 0,002977773 |
| 212913_at | BE674960 | Hs.371225 | mutS homolog 5 (E. coli) | MSH5 | EG2 | 6 | 0 | 4 | 5 | 0,007867576 | 0,079901528 | 8,42E-05 | 0,002977773 |
| 213006_at | AV655640 | Hs.440829 | CCAAT/enhancer binding protein (C/EBP), delta | CEBPD | EG2 | 1 | 5 | 3 | 6 | 0,007867576 | 0,079901528 | 8,42E-05 | 0,002977773 |
| 213035_at | AI081194 | Hs.335239 | ankyrin repeat domain 28 | ANKRD28 | EG2 | 3 | 3 | 7 | 2 | 0,007867576 | 0,079901528 | 8,42E-05 | 0,002977773 |
| 213338_at | BF062629 | Hs.35861 | | RIS1 | EG2 | 4 | 2 | 8 | 1 | 0,007867576 | 0,079901528 | 8,42E-05 | 0,002977773 |

| | | | | | | | | | | | | | |
|---|---|---|---|---|---|---|---|---|---|---|---|---|---|
| 213908_at | AI824078 | Hs.212670, Hs.513595 | | LOC339005 | EG2 | 4 | 2 | 4 | 5 | 0,0078675760,079901528 | | 8,42E-05 | 0,002977773 |
| 214255_at | N35112 | Hs.128041 | ATPase, Class V, type 10A | ATP10A | EG2 | 1 | 5 | 4 | 5 | 0,0078675760,079901528 | | 8,42E-05 | 0,002977773 |
| 214933_at | AA769818 | Hs.408449 | calcium channel, voltage-dependent, P/Q type, alpha | CACNA1A | EG2 | 1 | 5 | 6 | 3 | 0,0078675760,079901528 | | 8,42E-05 | 0,002977773 |
| 217838_s_at | NM_016337 | Hs.125867 | Enah/Vasp-like | EVL | EG2 | 0 | 6 | 2 | 7 | 0,0078675760,079901528 | | 8,42E-05 | 0,002977773 |
| 218475_at | NM_022727 | Hs.552590 | | HTF9C | EG2 | 0 | 6 | 4 | 5 | 0,0078675760,079901528 | | 8,42E-05 | 0,002977773 |
| 218729_at | NM_020169 | Hs.478067 | latexin | LXN | EG2 | 0 | 6 | 2 | 7 | 0,0078675760,079901528 | | 8,42E-05 | 0,002977773 |
| 218747_s_at | NM_018009 | Hs.504597 | TAP binding protein-like | TAPBPL | EG2 | 0 | 6 | 6 | 3 | 0,0078675760,079901528 | | 8,42E-05 | 0,002977773 |
| 219918_s_at | NM_018123 | Hs.121028 | asp (abnormal spindle)-like, microcephaly associated | ASPM | EG2 | 3 | 3 | 3 | 6 | 0,0078675760,079901528 | | 8,42E-05 | 0,002977773 |
| 221561_at | L21934 | Hs.496383 | sterol O-acyltransferase (acyl-Coenzyme A: cholester | SOAT1 | EG2 | 4 | 2 | 3 | 6 | 0,0078675760,079901528 | | 8,42E-05 | 0,002977773 |
| 222680_s_at | AK001261 | Hs.126774 | denticleless homolog (Drosophila) | DTL | EG2 | 3 | 3 | 4 | 5 | 0,0078675760,079901528 | | 8,42E-05 | 0,002977773 |
| 223194_s_at | AL512737 | Hs.132340 | chromosome 6 open reading frame 85 | C6orf85 | EG2 | 3 | 3 | 1 | 8 | 0,0078675760,079901528 | | 8,42E-05 | 0,002977773 |
| 223257_at | AI823905 | Hs.509008 | KIAA1333 | KIAA1333 | EG2 | 6 | 0 | 5 | 4 | 0,0078675760,079901528 | | 8,42E-05 | 0,002977773 |
| 224828_at | AV704132 | Hs.127126 | cytoplasmic polyadenylation element binding protein | CPEB4 | EG2 | 4 | 2 | 8 | 1 | 0,0078675760,079901528 | | 8,42E-05 | 0,002977773 |
| 224840_at | AI753747 | Hs.407190 | FK506 binding protein 5 | FKBP5 | EG2 | 5 | 1 | 3 | 6 | 0,0078675760,079901528 | | 8,42E-05 | 0,002977773 |
| 224860_at | AL137489 | Hs.7517 | chromosome 9 open reading frame 123 | C9orf123 | EG2 | 2 | 4 | 5 | 4 | 0,0078675760,079901528 | | 8,42E-05 | 0,002977773 |
| 224901_at | AL571375 | Hs.379191 | stearoyl-CoA desaturase 5 | SCD5 | EG2 | 2 | 4 | 7 | 2 | 0,0078675760,079901528 | | 8,42E-05 | 0,002977773 |
| 225177_at | AA143793 | Hs.191179 | RAB11 family interacting protein 1 (class I) | RAB11FIP1 | EG2 | 3 | 3 | 2 | 7 | 0,0078675760,079901528 | | 8,42E-05 | 0,002977773 |
| 225214_at | AI762915 | Hs.213470 | proteasome (prosome, macropain) subunit, beta type | PSMB7 | EG2 | 4 | 2 | 7 | 2 | 0,0078675760,079901528 | | 8,42E-05 | 0,002977773 |
| 225582_at | AA425726 | Hs.523252 | KIAA1754 | KIAA1754 | EG2 | 4 | 2 | 8 | 1 | 0,0078675760,079901528 | | 8,42E-05 | 0,002977773 |
| 226200_at | BE222664 | Hs.102910 | valyl-tRNA synthetase like | VARSL | EG2 | 0 | 6 | 5 | 4 | 0,0078675760,079901528 | | 8,42E-05 | 0,002977773 |
| 226275_at | AI188653 | Hs.468908 | MAX dimerization protein 1 | MXD1 | EG2 | 5 | 1 | 8 | 1 | 0,0078675760,079901528 | | 8,42E-05 | 0,002977773 |
| 226456_at | AW138157 | Hs.347524 | | MGC24665 | EG2 | 3 | 3 | 2 | 7 | 0,0078675760,079901528 | | 8,42E-05 | 0,002977773 |
| 226905_at | BG036514 | Hs.345588 | | MGC45871 | EG2 | 3 | 3 | 5 | 4 | 0,0078675760,079901528 | | 8,42E-05 | 0,002977773 |
| 226982_at | AI745624 | Hs.192221, H | elongation factor, RNA polymerase II, 2 | ELL2 | EG2 | 6 | 0 | 5 | 4 | 0,0078675760,079901528 | | 8,42E-05 | 0,002977773 |
| 227020_at | BE502982 | Hs.463613 | yippee-like 2 (Drosophila) | YPEL2 | EG2 | 1 | 5 | 1 | 8 | 0,0078675760,079901528 | | 8,42E-05 | 0,002977773 |
| 227062_at | AU155361 | Hs.523789 | | TncRNA | EG2 | 3 | 3 | 4 | 5 | 0,0078675760,079901528 | | 8,42E-05 | 0,002977773 |
| 227176_at | AL565362 | Hs.249829 | | FLJ40126 | EG2 | 5 | 1 | 6 | 3 | 0,0078675760,079901528 | | 8,42E-05 | 0,002977773 |
| 227501_at | AI377135 | Hs.446017 | WD repeat and SOCS box-containing 1 | WSB1 | EG2 | 4 | 2 | 6 | 3 | 0,0078675760,079901528 | | 8,42E-05 | 0,002977773 |
| 227755_at | AA042983 | | | | EG2 | 2 | 4 | 7 | 2 | 0,0078675760,079901528 | | 8,42E-05 | 0,002977773 |
| 227793_at | AA969238 | Hs.213065 | | LOC158257 | EG2 | 5 | 1 | 5 | 4 | 0,0078675760,079901528 | | 8,42E-05 | 0,002977773 |
| 227868_at | AI928764 | Hs.511547, Hs.532236 | | LOC154761 | EG2 | 0 | 6 | 3 | 6 | 0,0078675760,079901528 | | 8,42E-05 | 0,002977773 |
| 228287_at | BG054893 | Hs.471876, H | inhibitor of growth family, member 5 | ING5 | EG2 | 4 | 2 | 4 | 5 | 0,0078675760,079901528 | | 8,42E-05 | 0,002977773 |
| 228393_s_at | BF508739 | Hs.436350 | zinc finger protein 302 | ZNF302 | EG2 | 3 | 3 | 5 | 4 | 0,0078675760,079901528 | | 8,42E-05 | 0,002977773 |
| 229800_at | AI129626 | Hs.507755 | doublecortin and CaM kinase-like 1 | DCAMKL1 | EG2 | 3 | 3 | 4 | 5 | 0,0078675760,079901528 | | 8,42E-05 | 0,002977773 |
| 230629_s_at | AI809582 | Hs.507307 | E1A binding protein p400 | EP400 | EG2 | 1 | 5 | 3 | 6 | 0,0078675760,079901528 | | 8,42E-05 | 0,002977773 |
| 230733_at | H98113 | Hs.190086 | | MRCL3 | EG2 | 4 | 2 | 9 | 0 | 0,0078675760,079901528 | | 8,42E-05 | 0,002977773 |
| 230793_at | BE671038 | Hs.116470 | leucine rich repeat containing 16 | LRRC16 | EG2 | 5 | 1 | 3 | 6 | 0,0078675760,079901528 | | 8,42E-05 | 0,002977773 |
| 231035_s_at | AI697976 | Hs.499042 | OTU domain containing 1 | OTUD1 | EG2 | 2 | 4 | 6 | 3 | 0,0078675760,079901528 | | 8,42E-05 | 0,002977773 |
| 231727_s_at | NM_020679 | Hs.325631 | | AD023 | EG2 | 3 | 3 | 7 | 2 | 0,0078675760,079901528 | | 8,42E-05 | 0,002977773 |
| 232021_at | AI864273 | Hs.259347 | | LOC283464 | EG2 | 4 | 2 | 6 | 3 | 0,0078675760,079901528 | | 8,42E-05 | 0,002977773 |
| 232535_at | AL133570 | | | | EG2 | 5 | 1 | 3 | 6 | 0,0078675760,079901528 | | 8,42E-05 | 0,002977773 |
| 233401_at | BF723605 | Hs.21422 | neuronal cell adhesion molecule | NRCAM | EG2 | 4 | 2 | 4 | 5 | 0,0078675760,079901528 | | 8,42E-05 | 0,002977773 |
| 235085_at | BF739767 | Hs.29068 | | DKFZp761P0423 | EG2 | 1 | 5 | 4 | 5 | 0,0078675760,079901528 | | 8,42E-05 | 0,002977773 |
| 235289_at | AV747725 | Hs.164144 | eukaryotic translation initiation factor 5A2 | EIF5A2 | EG2 | 5 | 1 | 7 | 2 | 0,0078675760,079901528 | | 8,42E-05 | 0,002977773 |
| 236075_s_at | AI693543 | Hs.279914 | zinc finger protein 232 | ZNF232 | EG2 | 3 | 3 | 3 | 6 | 0,0078675760,079901528 | | 8,42E-05 | 0,002977773 |

| | | | | | | | | | | | | | |
|---|---|---|---|---|---|---|---|---|---|---|---|---|---|
| 236599_at | AA703280 | Hs.38449 | serpin peptidase inhibitor, clade E (nexin, plasminoge | SERPINE2 | EG2 | 3 | 3 | 9 | 0 | 0,0078675760,079901528 | | 8,42E-05 | 0,002977773 |
| 236834_at | AI025103 | Hs.302287 | sec1 family domain containing 2 | SCFD2 | EG2 | 2 | 4 | 6 | 3 | 0,0078675760,079901528 | | 8,42E-05 | 0,002977773 |
| 239944_at | AA431379 | | | | EG2 | 2 | 4 | 6 | 3 | 0,0078675760,079901528 | | 8,42E-05 | 0,002977773 |
| 240282_at | AW770902 | Hs.128548 | WD repeat domain 1 | WDR1 | EG2 | 3 | 3 | 7 | 2 | 0,0078675760,079901528 | | 8,42E-05 | 0,002977773 |
| 242029_at | N32832 | Hs.159430 | fibronectin type III domain containing 3B | FNDC3B | EG2 | 5 | 1 | 8 | 1 | 0,0078675760,079901528 | | 8,42E-05 | 0,002977773 |
| 242463_x_at | AI620827 | Hs.166312 | zinc finger protein 600 | ZNF600 | EG2 | 5 | 1 | 8 | 1 | 0,0078675760,079901528 | | 8,42E-05 | 0,002977773 |
| 243747_at | AI222019 | Hs.368375 | zinc finger protein 599 | ZNF599 | EG2 | 3 | 3 | 3 | 6 | 0,0078675760,079901528 | | 8,42E-05 | 0,002977773 |
| 244433_at | AI950023 | | | | EG2 | 3 | 3 | 8 | 1 | 0,0078675760,079901528 | | 8,42E-05 | 0,002977773 |
| 244623_at | BF513800 | | | | EG2 | 4 | 2 | 7 | 2 | 0,0078675760,079901528 | | 8,42E-05 | 0,002977773 |
| 37152_at | L07592 | Hs.485196 | peroxisome proliferative activated receptor, delta | PPARD | EG2 | 0 | 6 | 0 | 9 | 0,0078675760,079901528 | | 8,42E-05 | 0,002977773 |
| AFFX-r2-Bs-lys | AFFX-R2-BS-LYS-M | | | | EG2 | 3 | 3 | 4 | 5 | 0,0078675760,079901528 | | 8,42E-05 | 0,002977773 |
| 222912_at | BE207758 | Hs.503284 | arrestin, beta 1 | ARRB1 | EG2 | 0 | 5 | 8 | 9 | 0,03340802 | 0,201243287 | 3,06E-15 | 0,001562433 |
| 212811_x_at | AI889380 | Hs.323878 | solute carrier family 1 (glutamate/neutral amino acid t | SLC1A4 | EG2 | 4 | 1 | 9 | 7 | 0,03340802 | 0,201243287 | 1,94E-13 | 0,001562433 |
| 218145_at | NM_021158 | Hs.516826 | tribbles homolog 3 (Drosophila) | TRIB3 | EG2 | 0 | 5 | 10 | 5 | 0,03340802 | 0,201243287 | 7,74E-12 | 0,001562433 |
| 203542_s_at | AI690205 | Hs.150557 | Kruppel-like factor 9 | KLF9 | EG2 | 3 | 2 | 3 | 11 | 0,03340802 | 0,201243287 | 2,17E-10 | 0,001562433 |
| 212675_s_at | AB011154 | Hs.146007 | KIAA0582 | KIAA0582 | EG2 | 1 | 4 | 3 | 11 | 0,03340802 | 0,201243287 | 2,17E-10 | 0,001562433 |
| 213524_s_at | NM_015714 | Hs.432132 | G0/G1switch 2 | G0S2 | EG2 | 2 | 3 | 9 | 4 | 0,03340802 | 0,201243287 | 4,56E-09 | 0,001562433 |
| 225646_at | AI246687 | Hs.128065 | cathepsin C | CTSC | EG2 | 2 | 3 | 8 | 5 | 0,03340802 | 0,201243287 | 4,56E-09 | 0,001562433 |
| 235408_x_at | AW058673 | Hs.250693 | zinc finger protein 117 (HPF9) | ZNF117 | EG2 | 3 | 2 | 3 | 10 | 0,03340802 | 0,201243287 | 4,56E-09 | 0,001562433 |
| 1555950_a_at | CA448665 | Hs.527653 | decay accelerating factor for complement (CD55, Cro | DAF | EG2 | 3 | 2 | 6 | 6 | 0,03340802 | 0,201243287 | 7,43E-08 | 0,001562433 |
| 200907_s_at | AU157932 | Hs.151220 | | KIAA0992 | EG2 | 4 | 1 | 6 | 6 | 0,03340802 | 0,201243287 | 7,43E-08 | 0,001562433 |
| 202388_at | NM_002923 | Hs.78944 | regulator of G-protein signalling 2, 24kDa | RGS2 | EG2 | 1 | 4 | 7 | 5 | 0,03340802 | 0,201243287 | 7,43E-08 | 0,001562433 |
| 204472_at | NM_005261 | Hs.345139 | GTP binding protein overexpressed in skeletal muscle | GEM | EG2 | 5 | 0 | 12 | 0 | 0,03340802 | 0,201243287 | 7,43E-08 | 0,001562433 |
| 204595_s_at | AI300520 | Hs.25590 | stanniocalcin 1 | STC1 | EG2 | 2 | 3 | 10 | 2 | 0,03340802 | 0,201243287 | 7,43E-08 | 0,001562433 |
| 205505_at | NM_001490 | Hs.521568 | glucosaminyl (N-acetyl) transferase 1, core 2 (beta-1,6 | GCNT1 | EG2 | 1 | 4 | 6 | 6 | 0,03340802 | 0,201243287 | 7,43E-08 | 0,001562433 |
| 213112_s_at | N30649 | Hs.529892 | sequestosome 1 | SQSTM1 | EG2 | 5 | 0 | 10 | 2 | 0,03340802 | 0,201243287 | 7,43E-08 | 0,001562433 |
| 213850_s_at | AI984932 | Hs.210367 | splicing factor, arginine/serine-rich 2, interacting prot | SFRS2IP | EG2 | 2 | 3 | 7 | 5 | 0,03340802 | 0,201243287 | 7,43E-08 | 0,001562433 |
| 226136_at | N32834 | | | | EG2 | 5 | 0 | 7 | 5 | 0,03340802 | 0,201243287 | 7,43E-08 | 0,001562433 |
| 227354_at | BF589359 | Hs.266175, H | phosphoprotein associated with glycosphingolipid mi | PAG1 | EG2 | 3 | 2 | 7 | 5 | 0,03340802 | 0,201243287 | 7,43E-08 | 0,001562433 |
| 229374_at | AI758962 | Hs.371218 | EPH receptor A4 | EPHA4 | EG2 | 4 | 1 | 3 | 9 | 0,03340802 | 0,201243287 | 7,43E-08 | 0,001562433 |
| 1558173_a_at | AK093016 | Hs.257900, H | leucine zipper protein 1 | LUZP1 | EG2 | 1 | 4 | 6 | 5 | 0,03340802 | 0,201243287 | 9,59E-07 | 0,001562433 |
| 201212_at | D55696 | Hs.18069 | legumain | LGMN | EG2 | 4 | 1 | 4 | 7 | 0,03340802 | 0,201243287 | 9,59E-07 | 0,001562433 |
| 202206_at | AW450363 | Hs.111554 | ADP-ribosylation factor-like 7 | ARL7 | EG2 | 2 | 3 | 7 | 4 | 0,03340802 | 0,201243287 | 9,59E-07 | 0,001562433 |
| 203387_s_at | NM_014832 | Hs.210891 | TBC1 domain family, member 4 | TBC1D4 | EG2 | 2 | 3 | 4 | 7 | 0,03340802 | 0,201243287 | 9,59E-07 | 0,001562433 |
| 204014_at | NM_001394 | Hs.417962 | dual specificity phosphatase 4 | DUSP4 | EG2 | 2 | 3 | 3 | 8 | 0,03340802 | 0,201243287 | 9,59E-07 | 0,001562433 |
| 205027_s_at | NM_005204 | Hs.432453 | mitogen-activated protein kinase kinase kinase 8 | MAP3K8 | EG2 | 1 | 4 | 3 | 8 | 0,03340802 | 0,201243287 | 9,59E-07 | 0,001562433 |
| 206693_at | NM_000880 | Hs.536926 | interleukin 7 | IL7 | EG2 | 0 | 5 | 7 | 4 | 0,03340802 | 0,201243287 | 9,59E-07 | 0,001562433 |
| 208893_s_at | BC005047 | Hs.298654 | dual specificity phosphatase 6 | DUSP6 | EG2 | 3 | 2 | 9 | 2 | 0,03340802 | 0,201243287 | 9,59E-07 | 0,001562433 |
| 210715_s_at | AF027205 | Hs.31439 | serine peptidase inhibitor, Kunitz type, 2 | SPINT2 | EG2 | 3 | 2 | 3 | 8 | 0,03340802 | 0,201243287 | 9,59E-07 | 0,001562433 |
| 211668_s_at | K03226 | Hs.77274 | plasminogen activator, urokinase | PLAU | EG2 | 2 | 3 | 10 | 1 | 0,03340802 | 0,201243287 | 9,59E-07 | 0,001562433 |
| 212190_at | AL541302 | Hs.38449 | serpin peptidase inhibitor, clade E (nexin, plasminoge | SERPINE2 | EG2 | 2 | 3 | 7 | 4 | 0,03340802 | 0,201243287 | 9,59E-07 | 0,001562433 |
| 212444_at | AA156240 | | | | EG2 | 3 | 2 | 4 | 7 | 0,03340802 | 0,201243287 | 9,59E-07 | 0,001562433 |
| 216598_s_at | S69738 | Hs.303649 | chemokine (C-C motif) ligand 2 | CCL2 | EG2 | 3 | 2 | 5 | 6 | 0,03340802 | 0,201243287 | 9,59E-07 | 0,001562433 |
| 218409_s_at | NM_022365 | Hs.499000 | DnaJ (Hsp40) homolog, subfamily C, member 1 | DNAJC1 | EG2 | 0 | 5 | 9 | 2 | 0,03340802 | 0,201243287 | 9,59E-07 | 0,001562433 |
| 219352_at | NM_017912 | Hs.529317 | hect domain and RLD 6 | HERC6 | EG2 | 0 | 5 | 1 | 10 | 0,03340802 | 0,201243287 | 9,59E-07 | 0,001562433 |

| | | | | | | | | | | | | | | |
|---|---|---|---|---|---|---|---|---|---|---|---|---|---|---|
| 221757_at | BE042976 | Hs.26670 | | MGC17330 | EG2 | 1 | 4 | 4 | 7 | 0,03340802 | 0,201243287 | 9,59E-07 | 0,001562433 |
| 221865_at | BF969986 | Hs.522357 | chromosome 9 open reading frame 91 | C9orf91 | EG2 | 2 | 3 | 4 | 7 | 0,03340802 | 0,201243287 | 9,59E-07 | 0,001562433 |
| 223195_s_at | BF131886 | Hs.469543 | sestrin 2 | SESN2 | EG2 | 4 | 1 | 9 | 2 | 0,03340802 | 0,201243287 | 9,59E-07 | 0,001562433 |
| 223712_at | AL136721 | Hs.527213 | 6-pyruvoyl-tetrahydropterin synthase/dimerization c | PCBD2 | EG2 | 2 | 3 | 6 | 5 | 0,03340802 | 0,201243287 | 9,59E-07 | 0,001562433 |
| 226348_at | AK026764 | | | | EG2 | 0 | 5 | 9 | 2 | 0,03340802 | 0,201243287 | 9,59E-07 | 0,001562433 |
| 228423_at | AI887898 | Hs.549331 | | LOC389237 | EG2 | 5 | 0 | 4 | 7 | 0,03340802 | 0,201243287 | 9,59E-07 | 0,001562433 |
| 229071_at | AI681419 | Hs.262480 | | LOC388327 | EG2 | 2 | 3 | 3 | 8 | 0,03340802 | 0,201243287 | 9,59E-07 | 0,001562433 |
| 229307_at | N32051 | Hs.335239 | ankyrin repeat domain 28 | ANKRD28 | EG2 | 2 | 3 | 6 | 5 | 0,03340802 | 0,201243287 | 9,59E-07 | 0,001562433 |
| 230147_at | AI378647 | Hs.42502 | coagulation factor II (thrombin) receptor-like 2 | F2RL2 | EG2 | 1 | 4 | 8 | 3 | 0,03340802 | 0,201243287 | 9,59E-07 | 0,001562433 |
| 230508_at | AL569601 | Hs.292156 | dickkopf homolog 3 (Xenopus laevis) | DKK3 | EG2 | 2 | 3 | 6 | 5 | 0,03340802 | 0,201243287 | 9,59E-07 | 0,001562433 |
| 235266_at | AI139629 | Hs.370834 | ATPase family, AAA domain containing 2 | ATAD2 | EG2 | 2 | 3 | 3 | 8 | 0,03340802 | 0,201243287 | 9,59E-07 | 0,001562433 |
| 235836_at | N51413 | Hs.250723, H | matrix-remodelling associated 7 | MXRA7 | EG2 | 2 | 3 | 9 | 2 | 0,03340802 | 0,201243287 | 9,59E-07 | 0,001562433 |
| 236545_at | AA532718 | Hs.435512 | protein phosphatase 3 (formerly 2B), catalytic subunit | PPP3CA | EG2 | 2 | 3 | 9 | 2 | 0,03340802 | 0,201243287 | 9,59E-07 | 0,001562433 |
| 236907_at | AI760366 | Hs.387804 | poly(A) binding protein, cytoplasmic 1 | PABPC1 | EG2 | 3 | 2 | 3 | 8 | 0,03340802 | 0,201243287 | 9,59E-07 | 0,001562433 |
| 239049_at | BF514509 | | | | EG2 | 1 | 4 | 5 | 6 | 0,03340802 | 0,201243287 | 9,59E-07 | 0,001562433 |
| 242121_at | AW973232 | Hs.122121 | ring finger protein 12 | RNF12 | EG2 | 2 | 3 | 5 | 6 | 0,03340802 | 0,201243287 | 9,59E-07 | 0,001562433 |
| 244341_at | AA827728 | Hs.269528 | Mak3 homolog (S. cerevisiae) | MAK3 | EG2 | 2 | 3 | 7 | 4 | 0,03340802 | 0,201243287 | 9,59E-07 | 0,001562433 |
| 244753_at | BF000430 | Hs.270291 | actinin, alpha 4 | ACTN4 | EG2 | 3 | 2 | 10 | 1 | 0,03340802 | 0,201243287 | 9,59E-07 | 0,001562433 |
| 1568611_at | CA418310 | Hs.519568 | procollagen-proline, 2-oxoglutarate 4-dioxygenase (p | P4HA2 | EG2 | 0 | 5 | 6 | 4 | 0,03340802 | 0,201243287 | 9,97E-06 | 0,001562433 |
| 200906_s_at | AK025843 | Hs.151220 | | KIAA0992 | EG2 | 4 | 1 | 3 | 7 | 0,03340802 | 0,201243287 | 9,97E-06 | 0,001562433 |
| 202205_at | NM_003370 | Hs.515469 | vasodilator-stimulated phosphoprotein | VASP | EG2 | 4 | 1 | 4 | 6 | 0,03340802 | 0,201243287 | 9,97E-06 | 0,001562433 |
| 202350_s_at | NM_002380 | Hs.189445 | matrilin 2 | MATN2 | EG2 | 1 | 4 | 2 | 8 | 0,03340802 | 0,201243287 | 9,97E-06 | 0,001562433 |
| 202859_x_at | NM_000584 | Hs.624 | interleukin 8 | IL8 | EG2 | 3 | 2 | 9 | 1 | 0,03340802 | 0,201243287 | 9,97E-06 | 0,001562433 |
| 203397_s_at | BF063271 | Hs.170986 | UDP-N-acetyl-alpha-D-galactosamine:polypeptide N- | GALNT3 | EG2 | 4 | 1 | 8 | 2 | 0,03340802 | 0,201243287 | 9,97E-06 | 0,001562433 |
| 203823_at | NM_021106 | Hs.494875 | regulator of G-protein signalling 3 | RGS3 | EG2 | 0 | 5 | 8 | 2 | 0,03340802 | 0,201243287 | 9,97E-06 | 0,001562433 |
| 204221_x_at | U16307 | Hs.553516 | GLI pathogenesis-related 1 (glioma) | GLIPR1 | EG2 | 5 | 0 | 6 | 4 | 0,03340802 | 0,201243287 | 9,97E-06 | 0,001562433 |
| 204326_x_at | NM_002450 | Hs.374950 | metallothionein 1X | MT1X | EG2 | 2 | 3 | 5 | 5 | 0,03340802 | 0,201243287 | 9,97E-06 | 0,001562433 |
| 204407_at | AF080255 | Hs.486818 | transcription termination factor, RNA polymerase II | TTF2 | EG2 | 3 | 2 | 1 | 9 | 0,03340802 | 0,201243287 | 9,97E-06 | 0,001562433 |
| 204597_x_at | NM_003155 | Hs.25590 | stanniocalcin 1 | STC1 | EG2 | 2 | 3 | 9 | 1 | 0,03340802 | 0,201243287 | 9,97E-06 | 0,001562433 |
| 204638_at | NM_001611 | Hs.1211 | acid phosphatase 5, tartrate resistant | ACP5 | EG2 | 2 | 3 | 7 | 3 | 0,03340802 | 0,201243287 | 9,97E-06 | 0,001562433 |
| 205200_at | NM_003278 | Hs.476092 | C-type lectin domain family 3, member B | CLEC3B | EG2 | 0 | 5 | 3 | 7 | 0,03340802 | 0,201243287 | 9,97E-06 | 0,001562433 |
| 205559_s_at | NM_006200 | Hs.368542 | proprotein convertase subtilisin/kexin type 5 | PCSK5 | EG2 | 3 | 2 | 4 | 6 | 0,03340802 | 0,201243287 | 9,97E-06 | 0,001562433 |
| 206133_at | NM_017523 | Hs.441975 | | BIRC4BP | EG2 | 2 | 3 | 4 | 6 | 0,03340802 | 0,201243287 | 9,97E-06 | 0,001562433 |
| 206554_x_at | NM_006515 | Hs.475300 | SET domain and mariner transposase fusion gene | SETMAR | EG2 | 2 | 3 | 1 | 9 | 0,03340802 | 0,201243287 | 9,97E-06 | 0,001562433 |
| 206584_at | NM_015364 | Hs.69328 | lymphocyte antigen 96 | LY96 | EG2 | 2 | 3 | 5 | 5 | 0,03340802 | 0,201243287 | 9,97E-06 | 0,001562433 |
| 206686_at | NM_002610 | Hs.470633 | pyruvate dehydrogenase kinase, isoenzyme 1 | PDK1 | EG2 | 0 | 5 | 7 | 3 | 0,03340802 | 0,201243287 | 9,97E-06 | 0,001562433 |
| 206756_at | NM_019886 | Hs.138155 | carbohydrate (N-acetylglucosamine 6-O) sulfotransfer | CHST7 | EG2 | 0 | 5 | 9 | 1 | 0,03340802 | 0,201243287 | 9,97E-06 | 0,001562433 |
| 208527_x_at | NM_003523 | Hs.534369 | histone 1, H2be | HIST1H2BE | EG2 | 3 | 2 | 2 | 8 | 0,03340802 | 0,201243287 | 9,97E-06 | 0,001562433 |
| 208961_s_at | AB017493 | Hs.4055 | Kruppel-like factor 6 | KLF6 | EG2 | 1 | 4 | 6 | 4 | 0,03340802 | 0,201243287 | 9,97E-06 | 0,001562433 |
| 209258_s_at | AI373676 | Hs.24485 | chondroitin sulfate proteoglycan 6 (bamacan) | CSPG6 | EG2 | 1 | 4 | 6 | 4 | 0,03340802 | 0,201243287 | 9,97E-06 | 0,001562433 |
| 209276_s_at | AF162769 | Hs.28988 | glutaredoxin (thioltransferase) | GLRX | EG2 | 1 | 4 | 8 | 2 | 0,03340802 | 0,201243287 | 9,97E-06 | 0,001562433 |
| 209822_s_at | L22431 | Hs.370422 | very low density lipoprotein receptor | VLDLR | EG2 | 0 | 5 | 5 | 5 | 0,03340802 | 0,201243287 | 9,97E-06 | 0,001562433 |
| 212621_at | AB006624 | Hs.533787 | | KIAA0286 | EG2 | 5 | 0 | 6 | 4 | 0,03340802 | 0,201243287 | 9,97E-06 | 0,001562433 |
| 214169_at | BE615699 | Hs.438072 | unc-84 homolog A (C. elegans) | UNC84A | EG2 | 2 | 3 | 5 | 5 | 0,03340802 | 0,201243287 | 9,97E-06 | 0,001562433 |
| 215092_s_at | AJ005683 | Hs.371987 | nuclear factor of activated T-cells 5, tonicity-responsiv | NFAT5 | EG2 | 3 | 2 | 4 | 6 | 0,03340802 | 0,201243287 | 9,97E-06 | 0,001562433 |

| | | | | | | | | | | | | | |
|---|---|---|---|---|---|---|---|---|---|---|---|---|---|
| 218383_at | NM_017815 | Hs.442782 | chromosome 14 open reading frame 94 | C14orf94 | EG2 | 0 | 5 | 3 | 7 | 0,03340802 | 0,201243287 | 9,97E-06 | 0,001562433 |
| 219315_s_at | NM_024600 | Hs.459652 | chromosome 16 open reading frame 30 | C16orf30 | EG2 | 0 | 5 | 0 | 10 | 0,03340802 | 0,201243287 | 9,97E-06 | 0,001562433 |
| 219806_s_at | NM_020179 | Hs.438064 | | FN5 | EG2 | 1 | 4 | 5 | 5 | 0,03340802 | 0,201243287 | 9,97E-06 | 0,001562433 |
| 219938_s_at | NM_024430 | Hs.368623 | proline-serine-threonine phosphatase interacting pro | PSTPIP2 | EG2 | 2 | 3 | 8 | 2 | 0,03340802 | 0,201243287 | 9,97E-06 | 0,001562433 |
| 221833_at | AI971258 | Hs.295923 | seven in absentia homolog 1 (Drosophila) | SIAH1 | EG2 | 0 | 5 | 2 | 8 | 0,03340802 | 0,201243287 | 9,97E-06 | 0,001562433 |
| 221986_s_at | AW006750 | Hs.407709 | kelch-like 24 (Drosophila) | KLHL24 | EG2 | 5 | 0 | 4 | 6 | 0,03340802 | 0,201243287 | 9,97E-06 | 0,001562433 |
| 222736_s_at | BC000049 | Hs.411925 | transmembrane protein 38B | TMEM38B | EG2 | 5 | 0 | 8 | 2 | 0,03340802 | 0,201243287 | 9,97E-06 | 0,001562433 |
| 224496_s_at | BC006292 | Hs.513933 | | MGC10744 | EG2 | 3 | 2 | 5 | 5 | 0,03340802 | 0,201243287 | 9,97E-06 | 0,001562433 |
| 224707_at | AL522667 | Hs.483561 | | ORF1-FL49 | EG2 | 5 | 0 | 6 | 4 | 0,03340802 | 0,201243287 | 9,97E-06 | 0,001562433 |
| 225412_at | AA761169 | Hs.469690 | | FLJ14681 | EG2 | 3 | 2 | 5 | 5 | 0,03340802 | 0,201243287 | 9,97E-06 | 0,001562433 |
| 225611_at | AI922968 | Hs.133539 | microtubule associated serine/threonine kinase famil | MAST4 | EG2 | 3 | 2 | 0 | 10 | 0,03340802 | 0,201243287 | 9,97E-06 | 0,001562433 |
| 225855_at | AB046768 | Hs.369232 | erythrocyte membrane protein band 4.1 like 5 | EPB41L5 | EG2 | 4 | 1 | 3 | 7 | 0,03340802 | 0,201243287 | 9,97E-06 | 0,001562433 |
| 226051_at | BF973568 | Hs.55940 | | SELM | EG2 | 1 | 4 | 4 | 6 | 0,03340802 | 0,201243287 | 9,97E-06 | 0,001562433 |
| 226264_at | AL137432 | Hs.494827 | sushi domain containing 1 | SUSD1 | EG2 | 0 | 5 | 4 | 6 | 0,03340802 | 0,201243287 | 9,97E-06 | 0,001562433 |
| 227674_at | AA910852 | Hs.390568 | zinc finger protein 585A | ZNF585A | EG2 | 1 | 4 | 6 | 4 | 0,03340802 | 0,201243287 | 9,97E-06 | 0,001562433 |
| 227722_at | AW043594 | Hs.527193 | ribosomal protein S23 | RPS23 | EG2 | 3 | 2 | 8 | 2 | 0,03340802 | 0,201243287 | 9,97E-06 | 0,001562433 |
| 227911_at | AI935647 | Hs.183114 | Rho GTPase activating protein 28 | ARHGAP28 | EG2 | 1 | 4 | 1 | 9 | 0,03340802 | 0,201243287 | 9,97E-06 | 0,001562433 |
| 228490_at | AW292816 | Hs.549108 | abhydrolase domain containing 2 | ABHD2 | EG2 | 3 | 2 | 10 | 0 | 0,03340802 | 0,201243287 | 9,97E-06 | 0,001562433 |
| 229963_at | AV726956 | Hs.47209 | NGFRAP1-like 1 | NGFRAP1L1 | EG2 | 4 | 1 | 6 | 4 | 0,03340802 | 0,201243287 | 9,97E-06 | 0,001562433 |
| 230206_at | AI692645 | Hs.195403 | dedicator of cytokinesis 5 | DOCK5 | EG2 | 2 | 3 | 9 | 1 | 0,03340802 | 0,201243287 | 9,97E-06 | 0,001562433 |
| 230256_at | AW009436 | Hs.131810 | chromosome 1 open reading frame 104 | C1orf104 | EG2 | 0 | 5 | 6 | 4 | 0,03340802 | 0,201243287 | 9,97E-06 | 0,001562433 |
| 230292_at | AA868809 | Hs.25447 | regulator of chromosome condensation (RCC1) and BT | RCBTB2 | EG2 | 3 | 2 | 5 | 5 | 0,03340802 | 0,201243287 | 9,97E-06 | 0,001562433 |
| 231862_at | AK023520 | Hs.349283 | chromobox homolog 5 (HP1 alpha homolog, Drosophil | CBX5 | EG2 | 4 | 1 | 4 | 6 | 0,03340802 | 0,201243287 | 9,97E-06 | 0,001562433 |
| 233303_at | AL110175 | Hs.472031, H | ubiquitin-conjugating enzyme E2D 3 (UBC4/5 homolog | UBE2D3 | EG2 | 4 | 1 | 6 | 4 | 0,03340802 | 0,201243287 | 9,97E-06 | 0,001562433 |
| 235171_at | AI354636 | | | | EG2 | 2 | 3 | 6 | 4 | 0,03340802 | 0,201243287 | 9,97E-06 | 0,001562433 |
| 235609_at | BF056791 | Hs.532799 | BRCA1 interacting protein C-terminal helicase 1 | BRIP1 | EG2 | 4 | 1 | 3 | 7 | 0,03340802 | 0,201243287 | 9,97E-06 | 0,001562433 |
| 236524_at | AA737437 | Hs.276876 | TM2 domain containing 1 | TM2D1 | EG2 | 2 | 3 | 6 | 4 | 0,03340802 | 0,201243287 | 9,97E-06 | 0,001562433 |
| 237563_s_at | AI286239 | Hs.181156 | | LOC440731 | EG2 | 2 | 3 | 3 | 7 | 0,03340802 | 0,201243287 | 9,97E-06 | 0,001562433 |
| 1554153_a_at | BC015714 | Hs.502458 | PHD finger protein 21A | PHF21A | EG2 | 3 | 2 | 6 | 3 | 0,03340802 | 0,201243287 | 8,42E-05 | 0,002977773 |
| 1555976_s_at | BU676221 | Hs.233936 | | LOC440476 | EG2 | 5 | 0 | 7 | 2 | 0,03340802 | 0,201243287 | 8,42E-05 | 0,002977773 |
| 1556346_at | AJ227860 | Hs.289092 | coactosin-like 1 (Dictyostelium) | COTL1 | EG2 | 2 | 3 | 6 | 3 | 0,03340802 | 0,201243287 | 8,42E-05 | 0,002977773 |
| 1558290_a_at | BG200951 | Hs.133107 | | LOC441378 | EG2 | 0 | 5 | 4 | 5 | 0,03340802 | 0,201243287 | 8,42E-05 | 0,002977773 |
| 1559232_a_at | BC029450 | Hs.478031 | solute carrier family 33 (acetyl-CoA transporter), mem | SLC33A1 | EG2 | 4 | 1 | 9 | 0 | 0,03340802 | 0,201243287 | 8,42E-05 | 0,002977773 |
| 201171_at | NM_003945 | Hs.484188 | ATPase, H+ transporting, lysosomal 9kDa, V0 subunit e | ATP6V0E | EG2 | 1 | 4 | 5 | 4 | 0,03340802 | 0,201243287 | 8,42E-05 | 0,002977773 |
| 201482_at | NM_002826 | Hs.518374 | quiescin Q6 | QSCN6 | EG2 | 1 | 4 | 5 | 4 | 0,03340802 | 0,201243287 | 8,42E-05 | 0,002977773 |
| 201506_at | NM_000358 | Hs.369397 | transforming growth factor, beta-induced, 68kDa | TGFBI | EG2 | 2 | 3 | 6 | 3 | 0,03340802 | 0,201243287 | 8,42E-05 | 0,002977773 |
| 201795_at | NM_002296 | Hs.435166 | lamin B receptor | LBR | EG2 | 3 | 2 | 5 | 4 | 0,03340802 | 0,201243287 | 8,42E-05 | 0,002977773 |
| 202241_at | NM_025195 | Hs.444947 | tribbles homolog 1 (Drosophila) | TRIB1 | EG2 | 1 | 4 | 8 | 1 | 0,03340802 | 0,201243287 | 8,42E-05 | 0,002977773 |
| 202263_at | NM_016243 | Hs.334832 | cytochrome b5 reductase 1 | CYB5R1 | EG2 | 1 | 4 | 6 | 3 | 0,03340802 | 0,201243287 | 8,42E-05 | 0,002977773 |
| 202419_at | NM_002035 | Hs.74050 | follicular lymphoma variant translocation 1 | FVT1 | EG2 | 4 | 1 | 3 | 6 | 0,03340802 | 0,201243287 | 8,42E-05 | 0,002977773 |
| 202458_at | NM_007173 | Hs.25338 | protease, serine, 23 | PRSS23 | EG2 | 1 | 4 | 9 | 0 | 0,03340802 | 0,201243287 | 8,42E-05 | 0,002977773 |
| 202478_at | NM_021643 | Hs.467751 | tribbles homolog 2 (Drosophila) | TRIB2 | EG2 | 1 | 4 | 3 | 6 | 0,03340802 | 0,201243287 | 8,42E-05 | 0,002977773 |
| 203216_s_at | NM_004999 | Hs.149387 | myosin VI | MYO6 | EG2 | 5 | 0 | 5 | 4 | 0,03340802 | 0,201243287 | 8,42E-05 | 0,002977773 |
| 203231_s_at | AW235612 | Hs.434961 | ataxin 1 | ATXN1 | EG2 | 2 | 3 | 3 | 6 | 0,03340802 | 0,201243287 | 8,42E-05 | 0,002977773 |
| 203414_at | NM_012329 | Hs.463483 | monocyte to macrophage differentiation-associated | MMD | EG2 | 4 | 1 | 3 | 6 | 0,03340802 | 0,201243287 | 8,42E-05 | 0,002977773 |

| | | | | | | | | | | | | | |
|---|---|---|---|---|---|---|---|---|---|---|---|---|---|
| 203865_s_at | NM_015833 | Hs.474018 | adenosine deaminase, RNA-specific, B1 (RED1 homolo | ADARB1 | EG2 | 0 | 5 | 0 | 9 | 0,03340802 | 0,201243287 | 8,42E-05 | 0,002977773 |
| 203881_s_at | NM_004010 | Hs.495912 | dystrophin (muscular dystrophy, Duchenne and Becke | DMD | EG2 | 1 | 4 | 2 | 7 | 0,03340802 | 0,201243287 | 8,42E-05 | 0,002977773 |
| 204160_s_at | AW194947 | Hs.54037 | ectonucleotide pyrophosphatase/phosphodiesterase | ENPP4 | EG2 | 4 | 1 | 8 | 1 | 0,03340802 | 0,201243287 | 8,42E-05 | 0,002977773 |
| 204396_s_at | NM_005308 | Hs.524625 | G protein-coupled receptor kinase 5 | GRK5 | EG2 | 1 | 4 | 3 | 6 | 0,03340802 | 0,201243287 | 8,42E-05 | 0,002977773 |
| 204418_x_at | NM_000848 | Hs.279837 | glutathione S-transferase M2 (muscle) | GSTM2 | EG2 | 3 | 2 | 4 | 5 | 0,03340802 | 0,201243287 | 8,42E-05 | 0,002977773 |
| 204759_at | NM_001268 | Hs.25447 | regulator of chromosome condensation (RCC1) and BT | RCBTB2 | EG2 | 3 | 2 | 6 | 3 | 0,03340802 | 0,201243287 | 8,42E-05 | 0,002977773 |
| 204774_at | NM_014210 | Hs.553491 | ecotropic viral integration site 2A | EVI2A | EG2 | 4 | 1 | 6 | 3 | 0,03340802 | 0,201243287 | 8,42E-05 | 0,002977773 |
| 204971_at | NM_005213 | Hs.518198 | cystatin A (stefin A) | CSTA | EG2 | 2 | 3 | 9 | 0 | 0,03340802 | 0,201243287 | 8,42E-05 | 0,002977773 |
| 206307_s_at | NM_004472 | Hs.519385 | forkhead box D1 | FOXD1 | EG2 | 2 | 3 | 8 | 1 | 0,03340802 | 0,201243287 | 8,42E-05 | 0,002977773 |
| 207992_s_at | NM_000480 | Hs.501890 | adenosine monophosphate deaminase (isoform E) | AMPD3 | EG2 | 2 | 3 | 0 | 9 | 0,03340802 | 0,201243287 | 8,42E-05 | 0,002977773 |
| 208025_s_at | NM_003483 | Hs.505924 | high mobility group AT-hook 2 | HMGA2 | EG2 | 3 | 2 | 6 | 3 | 0,03340802 | 0,201243287 | 8,42E-05 | 0,002977773 |
| 208579_x_at | NM_017445 | Hs.473961 | H2B histone family, member S | H2BFS | EG2 | 3 | 2 | 1 | 8 | 0,03340802 | 0,201243287 | 8,42E-05 | 0,002977773 |
| 209447_at | AF043290 | Hs.12967 | spectrin repeat containing, nuclear envelope 1 | SYNE1 | EG2 | 2 | 3 | 9 | 0 | 0,03340802 | 0,201243287 | 8,42E-05 | 0,002977773 |
| 211000_s_at | AB015706 | Hs.454699, H | interleukin 6 signal transducer (gp130, oncostatin M re | IL6ST | EG2 | 4 | 1 | 1 | 8 | 0,03340802 | 0,201243287 | 8,42E-05 | 0,002977773 |
| 211571_s_at | D32039 | Hs.443681 | chondroitin sulfate proteoglycan 2 (versican) | CSPG2 | EG2 | 4 | 1 | 6 | 3 | 0,03340802 | 0,201243287 | 8,42E-05 | 0,002977773 |
| 212224_at | NM_000689 | Hs.76392 | aldehyde dehydrogenase 1 family, member A1 | ALDH1A1 | EG2 | 3 | 2 | 2 | 7 | 0,03340802 | 0,201243287 | 8,42E-05 | 0,002977773 |
| 212442_s_at | BG289001 | Hs.506829 | LAG1 longevity assurance homolog 6 (S. cerevisiae) | LASS6 | EG2 | 4 | 1 | 3 | 6 | 0,03340802 | 0,201243287 | 8,42E-05 | 0,002977773 |
| 212828_at | AA191573 | Hs.434494 | synaptojanin 2 | SYNJ2 | EG2 | 3 | 2 | 2 | 7 | 0,03340802 | 0,201243287 | 8,42E-05 | 0,002977773 |
| 212989_at | AI377497 | Hs.386215 | transmembrane protein 23 | TMEM23 | EG2 | 4 | 1 | 1 | 8 | 0,03340802 | 0,201243287 | 8,42E-05 | 0,002977773 |
| 213029_at | BG478428 | Hs.370359 | nuclear factor I/B | NFIB | EG2 | 3 | 2 | 4 | 5 | 0,03340802 | 0,201243287 | 8,42E-05 | 0,002977773 |
| 213286_at | BF445199 | Hs.435231 | zinc finger RNA binding protein | ZFR | EG2 | 4 | 1 | 6 | 3 | 0,03340802 | 0,201243287 | 8,42E-05 | 0,002977773 |
| 213328_at | AI936517 | Hs.481181 | NIMA (never in mitosis gene a)-related kinase 1 | NEK1 | EG2 | 1 | 4 | 5 | 4 | 0,03340802 | 0,201243287 | 8,42E-05 | 0,002977773 |
| 214305_s_at | AW003030 | Hs.471011 | splicing factor 3b, subunit 1, 155kDa | SF3B1 | EG2 | 2 | 3 | 6 | 3 | 0,03340802 | 0,201243287 | 8,42E-05 | 0,002977773 |
| 214375_at | AI962377 | Hs.172445 | PTPRF interacting protein, binding protein 1 (liprin be | PPFIBP1 | EG2 | 1 | 4 | 6 | 3 | 0,03340802 | 0,201243287 | 8,42E-05 | 0,002977773 |
| 216250_s_at | X77598 | Hs.125474 | leupaxin | LPXN | EG2 | 2 | 3 | 7 | 2 | 0,03340802 | 0,201243287 | 8,42E-05 | 0,002977773 |
| 218128_at | AU151875 | Hs.84928 | nuclear transcription factor Y, beta | NFYB | EG2 | 1 | 4 | 2 | 7 | 0,03340802 | 0,201243287 | 8,42E-05 | 0,002977773 |
| 218170_at | NM_016048 | Hs.483296 | isochorismatase domain containing 1 | ISOC1 | EG2 | 3 | 2 | 4 | 5 | 0,03340802 | 0,201243287 | 8,42E-05 | 0,002977773 |
| 218522_s_at | NM_018174 | Hs.66048 | BPY2 interacting protein 1 | BPY2IP1 | EG2 | 0 | 5 | 3 | 6 | 0,03340802 | 0,201243287 | 8,42E-05 | 0,002977773 |
| 218694_at | NM_016608 | Hs.9728 | armadillo repeat containing, X-linked 1 | ARMCX1 | EG2 | 3 | 2 | 4 | 5 | 0,03340802 | 0,201243287 | 8,42E-05 | 0,002977773 |
| 218772_x_at | NM_018112 | Hs.411925 | transmembrane protein 38B | TMEM38B | EG2 | 5 | 0 | 7 | 2 | 0,03340802 | 0,201243287 | 8,42E-05 | 0,002977773 |
| 219014_at | NM_016619 | Hs.546392 | placenta-specific 8 | PLAC8 | EG2 | 1 | 4 | 3 | 6 | 0,03340802 | 0,201243287 | 8,42E-05 | 0,002977773 |
| 219104_at | NM_016422 | Hs.44685 | ring finger protein 141 | RNF141 | EG2 | 2 | 3 | 1 | 8 | 0,03340802 | 0,201243287 | 8,42E-05 | 0,002977773 |
| 219131_at | NM_013319 | Hs.522933 | UbiA prenyltransferase domain containing 1 | UBIAD1 | EG2 | 2 | 3 | 8 | 1 | 0,03340802 | 0,201243287 | 8,42E-05 | 0,002977773 |
| 219132_at | NM_021255 | Hs.105103 | pellino homolog 2 (Drosophila) | PELI2 | EG2 | 4 | 1 | 4 | 5 | 0,03340802 | 0,201243287 | 8,42E-05 | 0,002977773 |
| 219308_s_at | NM_012093 | Hs.18268 | adenylate kinase 5 | AK5 | EG2 | 4 | 1 | 6 | 3 | 0,03340802 | 0,201243287 | 8,42E-05 | 0,002977773 |
| 221593_s_at | BC001663 | Hs.469473, H | ribosomal protein L31 | RPL31 | EG2 | 4 | 1 | 7 | 2 | 0,03340802 | 0,201243287 | 8,42E-05 | 0,002977773 |
| 221756_at | AL540260 | Hs.26670 | | MGC17330 | EG2 | 0 | 5 | 3 | 6 | 0,03340802 | 0,201243287 | 8,42E-05 | 0,002977773 |
| 222587_s_at | BF699855 | Hs.127407 | UDP-N-acetyl-alpha-D-galactosamine:polypeptide N- | GALNT7 | EG2 | 2 | 3 | 4 | 5 | 0,03340802 | 0,201243287 | 8,42E-05 | 0,002977773 |
| 223339_at | BC001867 | Hs.241336 | ATPase inhibitory factor 1 | ATPIF1 | EG2 | 4 | 1 | 5 | 4 | 0,03340802 | 0,201243287 | 8,42E-05 | 0,002977773 |
| 223622_s_at | AF284751 | Hs.473855 | hydroxypyruvate isomerase homolog (E. coli) | HYI | EG2 | 1 | 4 | 3 | 6 | 0,03340802 | 0,201243287 | 8,42E-05 | 0,002977773 |
| 223672_at | AL136561 | Hs.132121 | SH3-domain GRB2-like (endophilin) interacting protei | SGIP1 | EG2 | 4 | 1 | 5 | 4 | 0,03340802 | 0,201243287 | 8,42E-05 | 0,002977773 |
| 223769_x_at | AF284750 | Hs.473855 | hydroxypyruvate isomerase homolog (E. coli) | HYI | EG2 | 3 | 2 | 3 | 6 | 0,03340802 | 0,201243287 | 8,42E-05 | 0,002977773 |
| 224944_at | AL566034 | Hs.11355 | thymopoietin | TMPO | EG2 | 4 | 1 | 4 | 5 | 0,03340802 | 0,201243287 | 8,42E-05 | 0,002977773 |
| 225330_at | AL044092 | Hs.20573 | insulin-like growth factor 1 receptor | IGF1R | EG2 | 3 | 2 | 1 | 8 | 0,03340802 | 0,201243287 | 8,42E-05 | 0,002977773 |
| 225592_at | D81048 | Hs.519993 | nurim (nuclear envelope membrane protein) | NRM | EG2 | 2 | 3 | 5 | 4 | 0,03340802 | 0,201243287 | 8,42E-05 | 0,002977773 |

| | | | | | | | | | | | | | |
|---|---|---|---|---|---|---|---|---|---|---|---|---|---|
| 225816_at | AW138134 | Hs.12420 | PHD finger protein 17 | PHF17 | EG2 | 3 | 2 | 4 | 5 | 0,03340802 | 0,201243287 | 8,42E-05 | 0,002977773 |
| 226021_at | AW150720 | Hs.244940 | retinol dehydrogenase 10 (all-trans) | RDH10 | EG2 | 3 | 2 | 4 | 5 | 0,03340802 | 0,201243287 | 8,42E-05 | 0,002977773 |
| 226037_s_at | AL049589 | Hs.171723, H | TAF9-like RNA polymerase II, TATA box binding protei | TAF9L | EG2 | 5 | 0 | 3 | 6 | 0,03340802 | 0,201243287 | 8,42E-05 | 0,002977773 |
| 226272_at | N25986 | | | | EG2 | 3 | 2 | 1 | 8 | 0,03340802 | 0,201243287 | 8,42E-05 | 0,002977773 |
| 226384_at | BE858787 | Hs.437179 | phosphatidic acid phosphatase type 2 domain containi | PPAPDC1B | EG2 | 4 | 1 | 7 | 2 | 0,03340802 | 0,201243287 | 8,42E-05 | 0,002977773 |
| 226550_at | AI672159 | | | | EG2 | 4 | 1 | 4 | 5 | 0,03340802 | 0,201243287 | 8,42E-05 | 0,002977773 |
| 227114_at | BG435876 | Hs.380110 | | DKFZp547C195 | EG2 | 0 | 5 | 5 | 4 | 0,03340802 | 0,201243287 | 8,42E-05 | 0,002977773 |
| 227607_at | AI638611 | Hs.16229 | | AMSH-LP | EG2 | 2 | 3 | 3 | 6 | 0,03340802 | 0,201243287 | 8,42E-05 | 0,002977773 |
| 228333_at | AI912571 | | | | EG2 | 3 | 2 | 6 | 3 | 0,03340802 | 0,201243287 | 8,42E-05 | 0,002977773 |
| 228955_at | AL041761 | | | | EG2 | 4 | 1 | 4 | 5 | 0,03340802 | 0,201243287 | 8,42E-05 | 0,002977773 |
| 229359_at | BE552208 | | | | EG2 | 3 | 2 | 6 | 3 | 0,03340802 | 0,201243287 | 8,42E-05 | 0,002977773 |
| 229491_at | BF433180 | Hs.546482 | | LOC133308 | EG2 | 3 | 2 | 7 | 2 | 0,03340802 | 0,201243287 | 8,42E-05 | 0,002977773 |
| 229538_s_at | AW271106 | Hs.133294 | IQ motif containing GTPase activating protein 3 | IQGAP3 | EG2 | 3 | 2 | 3 | 6 | 0,03340802 | 0,201243287 | 8,42E-05 | 0,002977773 |
| 229692_at | AW135003 | Hs.435771 | apoptosis inhibitor 5 | API5 | EG2 | 4 | 1 | 8 | 1 | 0,03340802 | 0,201243287 | 8,42E-05 | 0,002977773 |
| 229850_at | N30152 | | | | EG2 | 5 | 0 | 3 | 6 | 0,03340802 | 0,201243287 | 8,42E-05 | 0,002977773 |
| 229865_at | AW058617 | Hs.159430 | fibronectin type III domain containing 3B | FNDC3B | EG2 | 4 | 1 | 5 | 4 | 0,03340802 | 0,201243287 | 8,42E-05 | 0,002977773 |
| 230345_at | AI654547 | | | | EG2 | 1 | 4 | 6 | 3 | 0,03340802 | 0,201243287 | 8,42E-05 | 0,002977773 |
| 231929_at | AI458439 | | | | EG2 | 3 | 2 | 4 | 5 | 0,03340802 | 0,201243287 | 8,42E-05 | 0,002977773 |
| 232164_s_at | AL137725 | Hs.200412 | epiplakin 1 | EPPK1 | EG2 | 3 | 2 | 1 | 8 | 0,03340802 | 0,201243287 | 8,42E-05 | 0,002977773 |
| 232889_at | AU147591 | | | LOC389281 | EG2 | 1 | 4 | 4 | 5 | 0,03340802 | 0,201243287 | 8,42E-05 | 0,002977773 |
| 233543_s_at | AK021582 | Hs.334772 | | FLJ13614 | EG2 | 3 | 2 | 5 | 4 | 0,03340802 | 0,201243287 | 8,42E-05 | 0,002977773 |
| 235046_at | AA456099 | | | | EG2 | 5 | 0 | 1 | 8 | 0,03340802 | 0,201243287 | 8,42E-05 | 0,002977773 |
| 235276_at | AA781795 | Hs.546467 | epithelial stromal interaction 1 (breast) | EPSTI1 | EG2 | 0 | 5 | 3 | 6 | 0,03340802 | 0,201243287 | 8,42E-05 | 0,002977773 |
| 235309_at | AA126311 | | | | EG2 | 3 | 2 | 6 | 3 | 0,03340802 | 0,201243287 | 8,42E-05 | 0,002977773 |
| 235653_s_at | BF685315 | Hs.479971 | THAP domain containing 6 | THAP6 | EG2 | 0 | 5 | 4 | 5 | 0,03340802 | 0,201243287 | 8,42E-05 | 0,002977773 |
| 236449_at | AI885390 | Hs.695 | cystatin B (stefin B) | CSTB | EG2 | 2 | 3 | 5 | 4 | 0,03340802 | 0,201243287 | 8,42E-05 | 0,002977773 |
| 237459_at | AA811257 | Hs.506415 | PCTAIRE protein kinase 2 | PCTK2 | EG2 | 1 | 4 | 9 | 0 | 0,03340802 | 0,201243287 | 8,42E-05 | 0,002977773 |
| 238320_at | AV659198 | Hs.523789 | | TncRNA | EG2 | 2 | 3 | 5 | 4 | 0,03340802 | 0,201243287 | 8,42E-05 | 0,002977773 |
| 238462_at | AI418293 | Hs.444075 | | STS-1 | EG2 | 4 | 1 | 3 | 6 | 0,03340802 | 0,201243287 | 8,42E-05 | 0,002977773 |
| 238673_at | AV724325 | | | | EG2 | 4 | 1 | 1 | 8 | 0,03340802 | 0,201243287 | 8,42E-05 | 0,002977773 |
| 239147_at | AI243677 | Hs.163914 | | DKFZp313G1735 | EG2 | 1 | 4 | 5 | 4 | 0,03340802 | 0,201243287 | 8,42E-05 | 0,002977773 |
| 239331_at | AW954199 | | | | EG2 | 3 | 2 | 5 | 4 | 0,03340802 | 0,201243287 | 8,42E-05 | 0,002977773 |
| 241017_at | AA779927 | Hs.469473, H | ribosomal protein L31 | RPL31 | EG2 | 3 | 2 | 2 | 7 | 0,03340802 | 0,201243287 | 8,42E-05 | 0,002977773 |
| 241359_at | AA729232 | | | | EG2 | 1 | 4 | 2 | 7 | 0,03340802 | 0,201243287 | 8,42E-05 | 0,002977773 |
| 241838_at | AA699443 | | | | EG2 | 3 | 2 | 1 | 8 | 0,03340802 | 0,201243287 | 8,42E-05 | 0,002977773 |
| 242201_at | AI418253 | Hs.397073 | postmeiotic segregation increased 2-like 5 | PMS2L5 | EG2 | 1 | 4 | 7 | 2 | 0,03340802 | 0,201243287 | 8,42E-05 | 0,002977773 |
| 242429_at | BF435183 | Hs.412517 | zinc finger protein 567 | ZNF567 | EG2 | 0 | 5 | 5 | 4 | 0,03340802 | 0,201243287 | 8,42E-05 | 0,002977773 |
| 242750_at | AA678047 | Hs.117747 | methylmalonic aciduria (cobalamin deficiency) cblA ty | MMAA | EG2 | 4 | 1 | 7 | 2 | 0,03340802 | 0,201243287 | 8,42E-05 | 0,002977773 |
| 242856_at | AI291804 | | | | EG2 | 2 | 3 | 5 | 4 | 0,03340802 | 0,201243287 | 8,42E-05 | 0,002977773 |
| 244025_at | BF590917 | | | | EG2 | 4 | 1 | 8 | 1 | 0,03340802 | 0,201243287 | 8,42E-05 | 0,002977773 |
| 91703_at | AA149545 | Hs.502867 | EH domain binding protein 1-like 1 | EHBP1L1 | EG2 | 1 | 4 | 5 | 4 | 0,03340802 | 0,201243287 | 8,42E-05 | 0,002977773 |
| 200742_s_at | BG231932 | Hs.523454 | tripeptidyl peptidase I | TPP1 | EG2 | 0 | 4 | 11 | 3 | 0,111952074 | 0,440114659 | 2,17E-10 | 0,001562433 |
| 221539_at | AB044548 | Hs.411641 | eukaryotic translation initiation factor 4E binding prot | EIF4EBP1 | EG2 | 0 | 4 | 8 | 6 | 0,111952074 | 0,440114659 | 2,17E-10 | 0,001562433 |
| 221911_at | BE881590 | Hs.22634 | ets variant gene 1 | ETV1 | EG2 | 2 | 2 | 9 | 5 | 0,111952074 | 0,440114659 | 2,17E-10 | 0,001562433 |
| 1553613_s_at | NM_001453 | Hs.348883 | forkhead box C1 | FOXC1 | EG2 | 1 | 3 | 3 | 10 | 0,111952074 | 0,440114659 | 4,56E-09 | 0,001562433 |

| | | | | | | | | | | | | | |
|---|---|---|---|---|---|---|---|---|---|---|---|---|---|
| 202679_at | NM_000271 | Hs.464779 | Niemann-Pick disease, type C1 | NPC1 | EG2 | 1 | 3 | 12 | 1 | 0,111952074 | 0,440114659 | 4,56E-09 | 0,001562433 |
| 204070_at | NM_004585 | Hs.17466 | retinoic acid receptor responder (tazarotene induced) | RARRES3 | EG2 | 2 | 2 | 4 | 9 | 0,111952074 | 0,440114659 | 4,56E-09 | 0,001562433 |
| 209459_s_at | AF237813 | Hs.336768 | 4-aminobutyrate aminotransferase | ABAT | EG2 | 2 | 2 | 6 | 7 | 0,111952074 | 0,440114659 | 4,56E-09 | 0,001562433 |
| 213067_at | AI382123 | Hs.16355 | myosin, heavy polypeptide 10, non-muscle | MYH10 | EG2 | 1 | 3 | 2 | 11 | 0,111952074 | 0,440114659 | 4,56E-09 | 0,001562433 |
| 224851_at | AW274756 | Hs.119882 | cyclin-dependent kinase 6 | CDK6 | EG2 | 2 | 2 | 5 | 8 | 0,111952074 | 0,440114659 | 4,56E-09 | 0,001562433 |
| 226757_at | AA131041 | Hs.437609 | interferon-induced protein with tetratricopeptide rep | IFIT2 | EG2 | 2 | 2 | 8 | 5 | 0,111952074 | 0,440114659 | 4,56E-09 | 0,001562433 |
| 242560_at | AA579890 | Hs.208388 | Fanconi anemia, complementation group D2 | FANCD2 | EG2 | 3 | 1 | 3 | 10 | 0,111952074 | 0,440114659 | 4,56E-09 | 0,001562433 |
| 203386_at | AI650848 | Hs.210891 | TBC1 domain family, member 4 | TBC1D4 | EG2 | 2 | 2 | 5 | 7 | 0,111952074 | 0,440114659 | 7,43E-08 | 0,001562433 |
| 207057_at | NM_004731 | Hs.439643 | solute carrier family 16 (monocarboxylic acid transpor | SLC16A7 | EG2 | 2 | 2 | 3 | 9 | 0,111952074 | 0,440114659 | 7,43E-08 | 0,001562433 |
| 209160_at | AB018580 | Hs.78183 | aldo-keto reductase family 1, member C3 (3-alpha hyd | AKR1C3 | EG2 | 1 | 3 | 6 | 6 | 0,111952074 | 0,440114659 | 7,43E-08 | 0,001562433 |
| 217998_at | NM_007350 | Hs.484885 | pleckstrin homology-like domain, family A, member 1 | PHLDA1 | EG2 | 2 | 2 | 10 | 2 | 0,111952074 | 0,440114659 | 7,43E-08 | 0,001562433 |
| 217999_s_at | NM_007350 | Hs.484885 | pleckstrin homology-like domain, family A, member 1 | PHLDA1 | EG2 | 3 | 1 | 10 | 2 | 0,111952074 | 0,440114659 | 7,43E-08 | 0,001562433 |
| 1554016_a_at | BC006291 | Hs.408702 | | FLJ13154 | EG2 | 0 | 4 | 7 | 4 | 0,111952074 | 0,440114659 | 9,59E-07 | 0,001562433 |
| 200879_s_at | NM_001430 | Hs.468410 | endothelial PAS domain protein 1 | EPAS1 | EG2 | 4 | 0 | 8 | 3 | 0,111952074 | 0,440114659 | 9,59E-07 | 0,001562433 |
| 201107_s_at | AI812030 | Hs.164226 | thrombospondin 1 | THBS1 | EG2 | 2 | 2 | 5 | 6 | 0,111952074 | 0,440114659 | 9,59E-07 | 0,001562433 |
| 201580_s_at | AL544094 | Hs.169358 | thioredoxin domain containing 13 | TXNDC13 | EG2 | 0 | 4 | 7 | 4 | 0,111952074 | 0,440114659 | 9,59E-07 | 0,001562433 |
| 201858_s_at | J03223 | Hs.1908 | proteoglycan 1, secretory granule | PRG1 | EG2 | 3 | 1 | 6 | 5 | 0,111952074 | 0,440114659 | 9,59E-07 | 0,001562433 |
| 202669_s_at | U16797 | Hs.149239 | ephrin-B2 | EFNB2 | EG2 | 2 | 2 | 0 | 11 | 0,111952074 | 0,440114659 | 9,59E-07 | 0,001562433 |
| 202838_at | NM_000147 | Hs.370858 | fucosidase, alpha-L- 1, tissue | FUCA1 | EG2 | 1 | 3 | 9 | 2 | 0,111952074 | 0,440114659 | 9,59E-07 | 0,001562433 |
| 204224_s_at | NM_000161 | Hs.86724 | GTP cyclohydrolase 1 (dopa-responsive dystonia) | GCH1 | EG2 | 2 | 2 | 7 | 4 | 0,111952074 | 0,440114659 | 9,59E-07 | 0,001562433 |
| 204338_s_at | NM_005613 | Hs.386726 | regulator of G-protein signalling 4 | RGS4 | EG2 | 3 | 1 | 9 | 2 | 0,111952074 | 0,440114659 | 9,59E-07 | 0,001562433 |
| 205047_s_at | NM_001673 | Hs.489207 | asparagine synthetase | ASNS | EG2 | 4 | 0 | 5 | 6 | 0,111952074 | 0,440114659 | 9,59E-07 | 0,001562433 |
| 206237_s_at | NM_013957 | Hs.453951 | neuregulin 1 | NRG1 | EG2 | 2 | 2 | 9 | 2 | 0,111952074 | 0,440114659 | 9,59E-07 | 0,001562433 |
| 212979_s_at | AW293343 | Hs.406492 | | KIAA0738 | EG2 | 2 | 2 | 4 | 7 | 0,111952074 | 0,440114659 | 9,59E-07 | 0,001562433 |
| 215813_s_at | S36219 | Hs.201978 | prostaglandin-endoperoxide synthase 1 (prostaglandi | PTGS1 | EG2 | 1 | 3 | 6 | 5 | 0,111952074 | 0,440114659 | 9,59E-07 | 0,001562433 |
| 218590_at | NM_021830 | Hs.22678 | progressive external ophthalmoplegia 1 | PEO1 | EG2 | 1 | 3 | 4 | 7 | 0,111952074 | 0,440114659 | 9,59E-07 | 0,001562433 |
| 219326_s_at | NM_006577 | Hs.173203 | UDP-GlcNAc:betaGal beta-1,3-N-acetylglucosaminyltr | B3GNT1 | EG2 | 4 | 0 | 6 | 5 | 0,111952074 | 0,440114659 | 9,59E-07 | 0,001562433 |
| 219675_s_at | NM_025076 | Hs.469561 | UDP-glucuronate decarboxylase 1 | UXS1 | EG2 | 2 | 2 | 4 | 7 | 0,111952074 | 0,440114659 | 9,59E-07 | 0,001562433 |
| 221864_at | AW517464 | Hs.460617 | | MGC13024 | EG2 | 0 | 4 | 6 | 5 | 0,111952074 | 0,440114659 | 9,59E-07 | 0,001562433 |
| 222572_at | BG542521 | Hs.22265 | protein phosphatase 2C, magnesium-dependent, cata | PPM2C | EG2 | 4 | 0 | 4 | 7 | 0,111952074 | 0,440114659 | 9,59E-07 | 0,001562433 |
| 223217_s_at | BE646573 | Hs.319171 | nuclear factor of kappa light polypeptide gene enhanc | NFKBIZ | EG2 | 0 | 4 | 4 | 7 | 0,111952074 | 0,440114659 | 9,59E-07 | 0,001562433 |
| 224480_s_at | BC006236 | Hs.99196 | | MGC11324 | EG2 | 3 | 1 | 5 | 6 | 0,111952074 | 0,440114659 | 9,59E-07 | 0,001562433 |
| 225798_at | AL047908 | Hs.368944 | | JAZF1 | EG2 | 1 | 3 | 6 | 5 | 0,111952074 | 0,440114659 | 9,59E-07 | 0,001562433 |
| 225929_s_at | AA233374 | Hs.195642 | chromosome 17 open reading frame 27 | C17orf27 | EG2 | 0 | 4 | 4 | 7 | 0,111952074 | 0,440114659 | 9,59E-07 | 0,001562433 |
| 226460_at | AB040883 | Hs.556658 | | KIAA1450 | EG2 | 2 | 2 | 6 | 5 | 0,111952074 | 0,440114659 | 9,59E-07 | 0,001562433 |
| 228244_at | BF062383 | Hs.103902 | biogenesis of lysosome-related organelles complex-1 | BLOC1S3 | EG2 | 0 | 4 | 7 | 4 | 0,111952074 | 0,440114659 | 9,59E-07 | 0,001562433 |
| 228416_at | AI149508 | Hs.470174 | origin recognition complex, subunit 4-like (yeast) | ORC4L | EG2 | 2 | 2 | 7 | 4 | 0,111952074 | 0,440114659 | 9,59E-07 | 0,001562433 |
| 228461_at | AL566989 | Hs.535157 | | LOC442040 | EG2 | 3 | 1 | 0 | 11 | 0,111952074 | 0,440114659 | 9,59E-07 | 0,001562433 |
| 228574_at | AI862551 | Hs.444240 | | DKFZp762A217 | EG2 | 1 | 3 | 0 | 11 | 0,111952074 | 0,440114659 | 9,59E-07 | 0,001562433 |
| 230109_at | AI638433 | Hs.126980 | phosphodiesterase 7B | PDE7B | EG2 | 2 | 2 | 2 | 9 | 0,111952074 | 0,440114659 | 9,59E-07 | 0,001562433 |
| 232031_s_at | AK023817 | Hs.514843 | KIAA1632 | KIAA1632 | EG2 | 3 | 1 | 11 | 0 | 0,111952074 | 0,440114659 | 9,59E-07 | 0,001562433 |
| 235405_at | N79662 | Hs.485557 | glutathione S-transferase A4 | GSTA4 | EG2 | 3 | 1 | 5 | 6 | 0,111952074 | 0,440114659 | 9,59E-07 | 0,001562433 |
| 235542_at | BF675754 | Hs.516107 | | MGC22014 | EG2 | 2 | 2 | 3 | 8 | 0,111952074 | 0,440114659 | 9,59E-07 | 0,001562433 |
| 236356_at | AI808395 | Hs.471207 | NADH dehydrogenase (ubiquinone) Fe-S protein 1, 75 | NDUFS1 | EG2 | 2 | 2 | 6 | 5 | 0,111952074 | 0,440114659 | 9,59E-07 | 0,001562433 |
| 238940_at | BE328128 | Hs.373857 | Kruppel-like factor 12 | KLF12 | EG2 | 4 | 0 | 4 | 7 | 0,111952074 | 0,440114659 | 9,59E-07 | 0,001562433 |

| | | | | | | | | | | | | | |
|---|---|---|---|---|---|---|---|---|---|---|---|---|---|
| 240383_at | AI239832 | Hs.472031, H | ubiquitin-conjugating enzyme E2D 3 (UBC4/5 homolog | UBE2D3 | EG2 | 2 | 2 | 6 | 5 | 0,1119520740 | 0,440114659 | 9,59E-07 | 0,001562433 |
| 242767_at | N95437 | Hs.475353 | LIM and cysteine-rich domains 1 | LMCD1 | EG2 | 2 | 2 | 5 | 6 | 0,1119520740 | 0,440114659 | 9,59E-07 | 0,001562433 |
| 244546_at | AI760495 | Hs.437060 | cytochrome c, somatic | CYCS | EG2 | 1 | 3 | 7 | 4 | 0,1119520740 | 0,440114659 | 9,59E-07 | 0,001562433 |
| 1557852_at | AW418842 | Hs.524271 | polyhomeotic-like 2 (Drosophila) | PHC2 | EG2 | 1 | 3 | 8 | 2 | 0,1119520740 | 0,440114659 | 9,97E-06 | 0,001562433 |
| 1559332_at | BC016339 | Hs.332847 | cysteine rich transmembrane BMP regulator 1 (chordi | CRIM1 | EG2 | 1 | 3 | 8 | 2 | 0,1119520740 | 0,440114659 | 9,97E-06 | 0,001562433 |
| 1565651_at | BI868311 | | | | EG2 | 0 | 4 | 9 | 1 | 0,1119520740 | 0,440114659 | 9,97E-06 | 0,001562433 |
| 203505_at | AF285167 | Hs.429294 | ATP-binding cassette, sub-family A (ABC1), member 1 | ABCA1 | EG2 | 4 | 0 | 3 | 7 | 0,1119520740 | 0,440114659 | 9,97E-06 | 0,001562433 |
| 203725_at | NM_001924 | Hs.80409 | growth arrest and DNA-damage-inducible, alpha | GADD45A | EG2 | 4 | 0 | 5 | 5 | 0,1119520740 | 0,440114659 | 9,97E-06 | 0,001562433 |
| 203817_at | W93728 | Hs.77890 | guanylate cyclase 1, soluble, beta 3 | GUCY1B3 | EG2 | 2 | 2 | 3 | 7 | 0,1119520740 | 0,440114659 | 9,97E-06 | 0,001562433 |
| 204352_at | NM_004619 | Hs.523930 | TNF receptor-associated factor 5 | TRAF5 | EG2 | 1 | 3 | 2 | 8 | 0,1119520740 | 0,440114659 | 9,97E-06 | 0,001562433 |
| 204646_at | NM_000110 | Hs.335034 | dihydropyrimidine dehydrogenase | DPYD | EG2 | 2 | 2 | 2 | 8 | 0,1119520740 | 0,440114659 | 9,97E-06 | 0,001562433 |
| 205885_s_at | L12002 | Hs.555880 | integrin, alpha 4 (antigen CD49D, alpha 4 subunit of VL | ITGA4 | EG2 | 2 | 2 | 3 | 7 | 0,1119520740 | 0,440114659 | 9,97E-06 | 0,001562433 |
| 205891_at | NM_000676 | Hs.167046 | adenosine A2b receptor | ADORA2B | EG2 | 3 | 1 | 6 | 4 | 0,1119520740 | 0,440114659 | 9,97E-06 | 0,001562433 |
| 205966_at | NM_005645 | Hs.502508 | TAF13 RNA polymerase II, TATA box binding protein (T | TAF13 | EG2 | 4 | 0 | 3 | 7 | 0,1119520740 | 0,440114659 | 9,97E-06 | 0,001562433 |
| 207574_s_at | NM_015675 | Hs.110571 | growth arrest and DNA-damage-inducible, beta | GADD45B | EG2 | 2 | 2 | 5 | 5 | 0,1119520740 | 0,440114659 | 9,97E-06 | 0,001562433 |
| 209305_s_at | AF078077 | Hs.110571 | growth arrest and DNA-damage-inducible, beta | GADD45B | EG2 | 2 | 2 | 7 | 3 | 0,1119520740 | 0,440114659 | 9,97E-06 | 0,001562433 |
| 209561_at | L38969 | Hs.169875 | thrombospondin 3 | THBS3 | EG2 | 1 | 3 | 4 | 6 | 0,1119520740 | 0,440114659 | 9,97E-06 | 0,001562433 |
| 210298_x_at | AF098518 | Hs.435369 | four and a half LIM domains 1 | FHL1 | EG2 | 1 | 3 | 1 | 9 | 0,1119520740 | 0,440114659 | 9,97E-06 | 0,001562433 |
| 211317_s_at | AF041461 | Hs.390736 | CASP8 and FADD-like apoptosis regulator | CFLAR | EG2 | 0 | 4 | 5 | 5 | 0,1119520740 | 0,440114659 | 9,97E-06 | 0,001562433 |
| 212092_at | BE858180 | Hs.147492 | paternally expressed 10 | PEG10 | EG2 | 3 | 1 | 4 | 6 | 0,1119520740 | 0,440114659 | 9,97E-06 | 0,001562433 |
| 213416_at | BG532690 | Hs.440955 | ceramide kinase-like | CERKL | EG2 | 1 | 3 | 0 | 10 | 0,1119520740 | 0,440114659 | 9,97E-06 | 0,001562433 |
| 213836_s_at | AW052084 | Hs.463964 | | WIPI49 | EG2 | 2 | 2 | 7 | 3 | 0,1119520740 | 0,440114659 | 9,97E-06 | 0,001562433 |
| 218543_s_at | NM_022750 | Hs.12646 | poly (ADP-ribose) polymerase family, member 12 | PARP12 | EG2 | 2 | 2 | 2 | 8 | 0,1119520740 | 0,440114659 | 9,97E-06 | 0,001562433 |
| 218613_at | NM_018422 | Hs.434255 | pleckstrin and Sec7 domain containing 3 | PSD3 | EG2 | 2 | 2 | 4 | 6 | 0,1119520740 | 0,440114659 | 9,97E-06 | 0,001562433 |
| 219073_s_at | NM_017784 | Hs.150122 | oxysterol binding protein-like 10 | OSBPL10 | EG2 | 1 | 3 | 4 | 6 | 0,1119520740 | 0,440114659 | 9,97E-06 | 0,001562433 |
| 219279_at | NM_017718 | Hs.46578 | dedicator of cytokinesis 10 | DOCK10 | EG2 | 4 | 0 | 3 | 7 | 0,1119520740 | 0,440114659 | 9,97E-06 | 0,001562433 |
| 220260_at | NM_018317 | Hs.479403 | TBC1 domain family, member 19 | TBC1D19 | EG2 | 3 | 1 | 6 | 4 | 0,1119520740 | 0,440114659 | 9,97E-06 | 0,001562433 |
| 220892_s_at | NM_021154 | Hs.494261 | phosphoserine aminotransferase 1 | PSAT1 | EG2 | 4 | 0 | 5 | 5 | 0,1119520740 | 0,440114659 | 9,97E-06 | 0,001562433 |
| 224847_at | AW051349 | Hs.119882 | cyclin-dependent kinase 6 | CDK6 | EG2 | 3 | 1 | 5 | 5 | 0,1119520740 | 0,440114659 | 9,97E-06 | 0,001562433 |
| 225373_at | BE271644 | Hs.47382, Hs.546421 | | C10orf54 | EG2 | 0 | 4 | 4 | 6 | 0,1119520740 | 0,440114659 | 9,97E-06 | 0,001562433 |
| 225529_at | AI492175 | Hs.535257 | centaurin, beta 5 | CENTB5 | EG2 | 1 | 3 | 6 | 4 | 0,1119520740 | 0,440114659 | 9,97E-06 | 0,001562433 |
| 225931_s_at | AI954660 | Hs.195642 | chromosome 17 open reading frame 27 | C17orf27 | EG2 | 0 | 4 | 5 | 5 | 0,1119520740 | 0,440114659 | 9,97E-06 | 0,001562433 |
| 226279_at | AW471145 | Hs.25338 | protease, serine, 23 | PRSS23 | EG2 | 3 | 1 | 10 | 0 | 0,1119520740 | 0,440114659 | 9,97E-06 | 0,001562433 |
| 226419_s_at | AA046439 | Hs.68714 | splicing factor, arginine/serine-rich 1 (splicing factor 2 | SFRS1 | EG2 | 4 | 0 | 6 | 4 | 0,1119520740 | 0,440114659 | 9,97E-06 | 0,001562433 |
| 226558_at | BE856637 | | | | EG2 | 2 | 2 | 5 | 5 | 0,1119520740 | 0,440114659 | 9,97E-06 | 0,001562433 |
| 227059_at | AI651255 | Hs.444329 | glypican 6 | GPC6 | EG2 | 2 | 2 | 5 | 5 | 0,1119520740 | 0,440114659 | 9,97E-06 | 0,001562433 |
| 227566_at | AW085558 | Hs.504352 | | HNT | EG2 | 2 | 2 | 8 | 2 | 0,1119520740 | 0,440114659 | 9,97E-06 | 0,001562433 |
| 228617_at | AA142842 | Hs.441975 | | BIRC4BP | EG2 | 1 | 3 | 5 | 5 | 0,1119520740 | 0,440114659 | 9,97E-06 | 0,001562433 |
| 228762_at | AW151924 | Hs.159142 | lunatic fringe homolog (Drosophila) | LFNG | EG2 | 1 | 3 | 6 | 4 | 0,1119520740 | 0,440114659 | 9,97E-06 | 0,001562433 |
| 229371_at | BF940010 | Hs.491611 | chromosome 8 open reading frame 40 | C8orf40 | EG2 | 4 | 0 | 5 | 5 | 0,1119520740 | 0,440114659 | 9,97E-06 | 0,001562433 |
| 230703_at | AA001543 | Hs.437831 | chromosome 14 open reading frame 32 | C14orf32 | EG2 | 3 | 1 | 5 | 5 | 0,1119520740 | 0,440114659 | 9,97E-06 | 0,001562433 |
| 230886_at | AW069499 | | | | EG2 | 3 | 1 | 5 | 5 | 0,1119520740 | 0,440114659 | 9,97E-06 | 0,001562433 |
| 231057_at | AU144266 | Hs.181326 | myotubularin related protein 2 | MTMR2 | EG2 | 3 | 1 | 7 | 3 | 0,1119520740 | 0,440114659 | 9,97E-06 | 0,001562433 |
| 231890_at | AK022804 | | | | EG2 | 3 | 1 | 7 | 3 | 0,1119520740 | 0,440114659 | 9,97E-06 | 0,001562433 |
| 232412_at | AK024690 | Hs.462946 | F-box and leucine-rich repeat protein 20 | FBXL20 | EG2 | 2 | 2 | 4 | 6 | 0,1119520740 | 0,440114659 | 9,97E-06 | 0,001562433 |

| | | | | | | | | | | | | | |
|---|---|---|---|---|---|---|---|---|---|---|---|---|---|
| 235919_at | AA227879 | | | | EG2 | 4 | 0 | 7 | 3 | 0,1119520740,440114659 | | 9,97E-06 | 0,001562433 |
| 236229_at | AW014345 | Hs.467370 | | LOC400721 | EG2 | 2 | 2 | 5 | 5 | 0,1119520740,440114659 | | 9,97E-06 | 0,001562433 |
| 236492_at | AI934447 | Hs.146339 | protein phosphatase 2 (formerly 2A), regulatory subu | PPP2R2A | EG2 | 4 | 0 | 8 | 2 | 0,1119520740,440114659 | | 9,97E-06 | 0,001562433 |
| 238449_at | BG534511 | Hs.528462, Hs.531664, Hs.547315, Hs.556385 | | DKFZp547E087 | EG2 | 4 | 0 | 5 | 5 | 0,1119520740,440114659 | | 9,97E-06 | 0,001562433 |
| 238824_at | BF844863 | | | | EG2 | 2 | 2 | 5 | 5 | 0,1119520740,440114659 | | 9,97E-06 | 0,001562433 |
| 242191_at | AI701905 | Hs.445080, Hs.454852 | | AE01 | EG2 | 3 | 1 | 6 | 4 | 0,1119520740,440114659 | | 9,97E-06 | 0,001562433 |
| 1552643_at | NM_145297 | Hs.50158 | zinc finger protein 626 | ZNF626 | EG2 | 2 | 2 | 5 | 4 | 0,1119520740,440114659 | | 8,42E-05 | 0,002977773 |
| 1557685_at | AK054840 | Hs.106015 | development and differentiation enhancing factor 1 | DDEF1 | EG2 | 2 | 2 | 3 | 6 | 0,1119520740,440114659 | | 8,42E-05 | 0,002977773 |
| 1558143_a_at | AK027160 | Hs.469658 | BCL2-like 11 (apoptosis facilitator) | BCL2L11 | EG2 | 4 | 0 | 7 | 2 | 0,1119520740,440114659 | | 8,42E-05 | 0,002977773 |
| 1566482_at | AL833114 | Hs.368986 | | LOC400047 | EG2 | 2 | 2 | 5 | 4 | 0,1119520740,440114659 | | 8,42E-05 | 0,002977773 |
| 1566557_at | AK096609 | Hs.448889 | | FLJ90757 | EG2 | 0 | 4 | 0 | 9 | 0,1119520740,440114659 | | 8,42E-05 | 0,002977773 |
| 1569022_a_at | BC040952 | Hs.175343 | phosphoinositide-3-kinase, class 2, alpha polypeptide | PIK3C2A | EG2 | 3 | 1 | 6 | 3 | 0,1119520740,440114659 | | 8,42E-05 | 0,002977773 |
| 200965_s_at | NM_006720 | Hs.438236, H | actin binding LIM protein 1 | ABLIM1 | EG2 | 2 | 2 | 2 | 7 | 0,1119520740,440114659 | | 8,42E-05 | 0,002977773 |
| 202345_s_at | NM_001444 | Hs.408061 | fatty acid binding protein 5 (psoriasis-associated) | FABP5 | EG2 | 4 | 0 | 4 | 5 | 0,1119520740,440114659 | | 8,42E-05 | 0,002977773 |
| 202742_s_at | NM_002731 | Hs.487325 | protein kinase, cAMP-dependent, catalytic, beta | PRKACB | EG2 | 4 | 0 | 5 | 4 | 0,1119520740,440114659 | | 8,42E-05 | 0,002977773 |
| 202761_s_at | NM_015180 | Hs.525392 | spectrin repeat containing, nuclear envelope 2 | SYNE2 | EG2 | 4 | 0 | 3 | 6 | 0,1119520740,440114659 | | 8,42E-05 | 0,002977773 |
| 202897_at | AB023430 | Hs.128846 | protein tyrosine phosphatase, non-receptor type subs | PTPNS1 | EG2 | 1 | 3 | 5 | 4 | 0,1119520740,440114659 | | 8,42E-05 | 0,002977773 |
| 203128_at | NM_004863 | Hs.435661 | serine palmitoyltransferase, long chain base subunit 2 | SPTLC2 | EG2 | 0 | 4 | 7 | 2 | 0,1119520740,440114659 | | 8,42E-05 | 0,002977773 |
| 203492_x_at | AA918224 | Hs.101014 | | PIG8 | EG2 | 3 | 1 | 4 | 5 | 0,1119520740,440114659 | | 8,42E-05 | 0,002977773 |
| 203493_s_at | AL525206 | Hs.101014 | | PIG8 | EG2 | 3 | 1 | 4 | 5 | 0,1119520740,440114659 | | 8,42E-05 | 0,002977773 |
| 203753_at | NM_003199 | Hs.200285 | transcription factor 4 | TCF4 | EG2 | 0 | 4 | 4 | 5 | 0,1119520740,440114659 | | 8,42E-05 | 0,002977773 |
| 203821_at | NM_001945 | Hs.799 | heparin-binding EGF-like growth factor | HBEGF | EG2 | 4 | 0 | 9 | 0 | 0,1119520740,440114659 | | 8,42E-05 | 0,002977773 |
| 203921_at | NM_004267 | Hs.8786 | carbohydrate (N-acetylglucosamine-6-O) sulfotransfe | CHST2 | EG2 | 2 | 2 | 7 | 2 | 0,1119520740,440114659 | | 8,42E-05 | 0,002977773 |
| 204088_at | NM_002560 | Hs.321709 | purinergic receptor P2X, ligand-gated ion channel, 4 | P2RX4 | EG2 | 1 | 3 | 3 | 6 | 0,1119520740,440114659 | | 8,42E-05 | 0,002977773 |
| 204363_at | NM_001993 | Hs.62192 | coagulation factor III (thromboplastin, tissue factor) | F3 | EG2 | 1 | 3 | 2 | 7 | 0,1119520740,440114659 | | 8,42E-05 | 0,002977773 |
| 204663_at | NM_006680 | Hs.199743 | malic enzyme 3, NADP(+)-dependent, mitochondrial | ME3 | EG2 | 0 | 4 | 8 | 1 | 0,1119520740,440114659 | | 8,42E-05 | 0,002977773 |
| 204720_s_at | AV729634 | Hs.536063 | DnaJ (Hsp40) homolog, subfamily C, member 6 | DNAJC6 | EG2 | 3 | 1 | 6 | 3 | 0,1119520740,440114659 | | 8,42E-05 | 0,002977773 |
| 204862_s_at | NM_002513 | Hs.514065 | non-metastatic cells 3, protein expressed in | NME3 | EG2 | 1 | 3 | 5 | 4 | 0,1119520740,440114659 | | 8,42E-05 | 0,002977773 |
| 205379_at | NM_001236 | Hs.154510 | carbonyl reductase 3 | CBR3 | EG2 | 2 | 2 | 1 | 8 | 0,1119520740,440114659 | | 8,42E-05 | 0,002977773 |
| 205648_at | NM_003391 | Hs.128553 | wingless-type MMTV integration site family member 2 | WNT2 | EG2 | 0 | 4 | 1 | 8 | 0,1119520740,440114659 | | 8,42E-05 | 0,002977773 |
| 206025_s_at | AW188198 | Hs.437322 | tumor necrosis factor, alpha-induced protein 6 | TNFAIP6 | EG2 | 2 | 2 | 8 | 1 | 0,1119520740,440114659 | | 8,42E-05 | 0,002977773 |
| 207304_at | NM_003425 | Hs.381285 | zinc finger protein 45 | ZNF45 | EG2 | 0 | 4 | 7 | 2 | 0,1119520740,440114659 | | 8,42E-05 | 0,002977773 |
| 209015_s_at | BC002446 | Hs.490745 | DnaJ (Hsp40) homolog, subfamily B, member 6 | DNAJB6 | EG2 | 4 | 0 | 3 | 6 | 0,1119520740,440114659 | | 8,42E-05 | 0,002977773 |
| 209292_at | AL022726 | Hs.519601 | inhibitor of DNA binding 4, dominant negative helix-l | ID4 | EG2 | 3 | 1 | 8 | 1 | 0,1119520740,440114659 | | 8,42E-05 | 0,002977773 |
| 209829_at | AB002384 | Hs.484915 | chromosome 6 open reading frame 32 | C6orf32 | EG2 | 3 | 1 | 0 | 9 | 0,1119520740,440114659 | | 8,42E-05 | 0,002977773 |
| 211981_at | NM_001845 | Hs.17441 | collagen, type IV, alpha 1 | COL4A1 | EG2 | 2 | 2 | 4 | 5 | 0,1119520740,440114659 | | 8,42E-05 | 0,002977773 |
| 212070_at | AL554008 | Hs.513633 | G protein-coupled receptor 56 | GPR56 | EG2 | 0 | 4 | 9 | 0 | 0,1119520740,440114659 | | 8,42E-05 | 0,002977773 |
| 212136_at | AW517686 | Hs.343522 | ATPase, Ca++ transporting, plasma membrane 4 | ATP2B4 | EG2 | 2 | 2 | 5 | 4 | 0,1119520740,440114659 | | 8,42E-05 | 0,002977773 |
| 212607_at | N32526 | Hs.498292 | v-akt murine thymoma viral oncogene homolog 3 (pro | AKT3 | EG2 | 4 | 0 | 3 | 6 | 0,1119520740,440114659 | | 8,42E-05 | 0,002977773 |
| 213069_at | AI148659 | Hs.477420 | HEG homolog 1 (zebrafish) | HEG1 | EG2 | 1 | 3 | 6 | 3 | 0,1119520740,440114659 | | 8,42E-05 | 0,002977773 |
| 213435_at | AB028957 | Hs.516617 | SATB family member 2 | SATB2 | EG2 | 3 | 1 | 7 | 2 | 0,1119520740,440114659 | | 8,42E-05 | 0,002977773 |
| 213449_at | D31765 | Hs.252828 | processing of precursor 1, ribonuclease P/MRP subuni | POP1 | EG2 | 2 | 2 | 6 | 3 | 0,1119520740,440114659 | | 8,42E-05 | 0,002977773 |
| 213652_at | AU152579 | Hs.368542 | proprotein convertase subtilisin/kexin type 5 | PCSK5 | EG2 | 3 | 1 | 3 | 6 | 0,1119520740,440114659 | | 8,42E-05 | 0,002977773 |
| 216035_x_at | AV721430 | Hs.501080 | transcription factor 7-like 2 (T-cell specific, HMG-box) | TCF7L2 | EG2 | 2 | 2 | 6 | 3 | 0,1119520740,440114659 | | 8,42E-05 | 0,002977773 |
| 217452_s_at | Y15014 | Hs.518834 | UDP-Gal:betaGlcNAc beta 1,3-galactosyltransferase, p | B3GALT2 | EG2 | 3 | 1 | 3 | 6 | 0,1119520740,440114659 | | 8,42E-05 | 0,002977773 |

| | | | | | | | | | | | | | |
|---|---|---|---|---|---|---|---|---|---|---|---|---|---|
| 218537_at | NM_017885 | Hs.412304, H | host cell factor C1 regulator 1 (XPO1 dependant) | HCFC1R1 | EG2 | 0 | 4 | 5 | 4 | 0,1119520740 | 0,440114659 | 8,42E-05 | 0,002977773 |
| 219737_s_at | AI524125 | Hs.407643 | protocadherin 9 | PCDH9 | EG2 | 2 | 2 | 7 | 2 | 0,1119520740 | 0,440114659 | 8,42E-05 | 0,002977773 |
| 220651_s_at | NM_018518 | Hs.198363 | MCM10 minichromosome maintenance deficient 10 (S | MCM10 | EG2 | 3 | 1 | 4 | 5 | 0,1119520740 | 0,440114659 | 8,42E-05 | 0,002977773 |
| 221127_s_at | NM_006394 | Hs.292156 | | RIG | EG2 | 0 | 4 | 3 | 6 | 0,1119520740 | 0,440114659 | 8,42E-05 | 0,002977773 |
| 221423_s_at | NM_030799 | Hs.372050 | Yip1 domain family, member 5 | YIPF5 | EG2 | 3 | 1 | 5 | 4 | 0,1119520740 | 0,440114659 | 8,42E-05 | 0,002977773 |
| 222044_at | AI199589 | Hs.472856 | chromosome 20 open reading frame 67 | C20orf67 | EG2 | 2 | 2 | 6 | 3 | 0,1119520740 | 0,440114659 | 8,42E-05 | 0,002977773 |
| 222557_at | AL353715 | Hs.348326 | stathmin-like 3 | STMN3 | EG2 | 0 | 4 | 3 | 6 | 0,1119520740 | 0,440114659 | 8,42E-05 | 0,002977773 |
| 222853_at | N71923 | Hs.41296 | fibronectin leucine rich transmembrane protein 3 | FLRT3 | EG2 | 0 | 4 | 4 | 5 | 0,1119520740 | 0,440114659 | 8,42E-05 | 0,002977773 |
| 223315_at | AF278532 | Hs.201034 | netrin 4 | NTN4 | EG2 | 2 | 2 | 1 | 8 | 0,1119520740 | 0,440114659 | 8,42E-05 | 0,002977773 |
| 223614_at | AL136588 | Hs.492187 | | DKFZp761D112 | EG2 | 2 | 2 | 7 | 2 | 0,1119520740 | 0,440114659 | 8,42E-05 | 0,002977773 |
| 223805_at | AF323728 | Hs.318775 | oxysterol binding protein-like 6 | OSBPL6 | EG2 | 3 | 1 | 6 | 3 | 0,1119520740 | 0,440114659 | 8,42E-05 | 0,002977773 |
| 225724_at | AW136120 | Hs.531089 | proteasome (prosome, macropain) subunit, alpha typ | PSMA3 | EG2 | 3 | 1 | 5 | 4 | 0,1119520740 | 0,440114659 | 8,42E-05 | 0,002977773 |
| 225879_at | BF345244 | Hs.378501 | tRNA splicing endonuclease 54 homolog (SEN54, S. cer | TSEN54 | EG2 | 1 | 3 | 6 | 3 | 0,1119520740 | 0,440114659 | 8,42E-05 | 0,002977773 |
| 225957_at | AI307750 | Hs.484195 | adult retina protein | LOC153222 | EG2 | 2 | 2 | 5 | 4 | 0,1119520740 | 0,440114659 | 8,42E-05 | 0,002977773 |
| 226150_at | BF111651 | Hs.437179 | phosphatidic acid phosphatase type 2 domain containi | PPAPDC1B | EG2 | 3 | 1 | 8 | 1 | 0,1119520740 | 0,440114659 | 8,42E-05 | 0,002977773 |
| 226157_at | AI569747 | Hs.379018 | transcription factor Dp-2 (E2F dimerization partner 2) | TFDP2 | EG2 | 2 | 2 | 6 | 3 | 0,1119520740 | 0,440114659 | 8,42E-05 | 0,002977773 |
| 226210_s_at | AI291123 | Hs.525589, H | maternally expressed 3 | MEG3 | EG2 | 0 | 4 | 3 | 6 | 0,1119520740 | 0,440114659 | 8,42E-05 | 0,002977773 |
| 226670_s_at | AL109839 | Hs.411022 | chromosome 20 open reading frame 119 | C20orf119 | EG2 | 2 | 2 | 6 | 3 | 0,1119520740 | 0,440114659 | 8,42E-05 | 0,002977773 |
| 226869_at | AI655611 | | | | EG2 | 1 | 3 | 1 | 8 | 0,1119520740 | 0,440114659 | 8,42E-05 | 0,002977773 |
| 227051_at | AU157716 | | | | EG2 | 2 | 2 | 8 | 1 | 0,1119520740 | 0,440114659 | 8,42E-05 | 0,002977773 |
| 227088_at | BF221547 | Hs.370661 | phosphodiesterase 5A, cGMP-specific | PDE5A | EG2 | 3 | 1 | 2 | 7 | 0,1119520740 | 0,440114659 | 8,42E-05 | 0,002977773 |
| 227249_at | AI857685 | Hs.460107 | nudE nuclear distribution gene E homolog 1 (A. nidula | NDE1 | EG2 | 2 | 2 | 4 | 5 | 0,1119520740 | 0,440114659 | 8,42E-05 | 0,002977773 |
| 227605_at | AI762870 | Hs.480465 | small inducible cytokine subfamily E, member 1 (endo | SCYE1 | EG2 | 3 | 1 | 3 | 6 | 0,1119520740 | 0,440114659 | 8,42E-05 | 0,002977773 |
| 227649_s_at | AU144000 | Hs.497575 | SLIT-ROBO Rho GTPase activating protein 2 | SRGAP2 | EG2 | 2 | 2 | 6 | 3 | 0,1119520740 | 0,440114659 | 8,42E-05 | 0,002977773 |
| 228062_at | AW025330 | Hs.12554 | nucleosome assembly protein 1-like 5 | NAP1L5 | EG2 | 3 | 1 | 7 | 2 | 0,1119520740 | 0,440114659 | 8,42E-05 | 0,002977773 |
| 228885_at | AI862120 | Hs.127386 | MAM domain containing 2 | MAMDC2 | EG2 | 1 | 3 | 6 | 3 | 0,1119520740 | 0,440114659 | 8,42E-05 | 0,002977773 |
| 228927_at | AW291411 | Hs.464896 | zinc finger protein 397 | ZNF397 | EG2 | 4 | 0 | 3 | 6 | 0,1119520740 | 0,440114659 | 8,42E-05 | 0,002977773 |
| 229549_at | AA868461 | Hs.550465 | opsin 1 (cone pigments), short-wave-sensitive (color | OPN1SW | EG2 | 4 | 0 | 7 | 2 | 0,1119520740 | 0,440114659 | 8,42E-05 | 0,002977773 |
| 229693_at | AI952836 | Hs.462230 | | LOC388335 | EG2 | 1 | 3 | 2 | 7 | 0,1119520740 | 0,440114659 | 8,42E-05 | 0,002977773 |
| 229886_at | AA502768 | Hs.448041 | | FLJ32363 | EG2 | 3 | 1 | 3 | 6 | 0,1119520740 | 0,440114659 | 8,42E-05 | 0,002977773 |
| 230218_at | BF476403 | | | | EG2 | 2 | 2 | 2 | 7 | 0,1119520740 | 0,440114659 | 8,42E-05 | 0,002977773 |
| 231234_at | AV699565 | Hs.128065 | cathepsin C | CTSC | EG2 | 2 | 2 | 5 | 4 | 0,1119520740 | 0,440114659 | 8,42E-05 | 0,002977773 |
| 231513_at | BF111326 | Hs.1547 | potassium inwardly-rectifying channel, subfamily J, m | KCNJ2 | EG2 | 3 | 1 | 4 | 5 | 0,1119520740 | 0,440114659 | 8,42E-05 | 0,002977773 |
| 231779_at | AI246590 | Hs.449207 | interleukin-1 receptor-associated kinase 2 | IRAK2 | EG2 | 3 | 1 | 8 | 1 | 0,1119520740 | 0,440114659 | 8,42E-05 | 0,002977773 |
| 231807_at | AL157473 | Hs.445885 | KIAA1217 | KIAA1217 | EG2 | 1 | 3 | 1 | 8 | 0,1119520740 | 0,440114659 | 8,42E-05 | 0,002977773 |
| 231956_at | AA976354 | Hs.514554 | KIAA1618 | KIAA1618 | EG2 | 1 | 3 | 4 | 5 | 0,1119520740 | 0,440114659 | 8,42E-05 | 0,002977773 |
| 232038_at | AI052103 | Hs.121396 | chromosome 6 open reading frame 170 | C6orf170 | EG2 | 3 | 1 | 4 | 5 | 0,1119520740 | 0,440114659 | 8,42E-05 | 0,002977773 |
| 232238_at | AK001380 | Hs.121028 | asp (abnormal spindle)-like, microcephaly associated | ASPM | EG2 | 2 | 2 | 2 | 7 | 0,1119520740 | 0,440114659 | 8,42E-05 | 0,002977773 |
| 232589_at | AK021551 | | | | EG2 | 2 | 2 | 3 | 6 | 0,1119520740 | 0,440114659 | 8,42E-05 | 0,002977773 |
| 232865_at | N59653 | Hs.519313 | AF4/FMR2 family, member 4 | AFF4 | EG2 | 2 | 2 | 7 | 2 | 0,1119520740 | 0,440114659 | 8,42E-05 | 0,002977773 |
| 233223_at | AK000850 | Hs.37982 | neural precursor cell expressed, developmentally do | NEDD9 | EG2 | 1 | 3 | 8 | 1 | 0,1119520740 | 0,440114659 | 8,42E-05 | 0,002977773 |
| 236009_at | AI767250 | | | | EG2 | 3 | 1 | 7 | 2 | 0,1119520740 | 0,440114659 | 8,42E-05 | 0,002977773 |
| 236016_at | AI702962 | Hs.463010 | SWI/SNF related, matrix associated, actin dependent r | SMARCE1 | EG2 | 0 | 4 | 5 | 4 | 0,1119520740 | 0,440114659 | 8,42E-05 | 0,002977773 |
| 238469_at | BE620374 | Hs.368337 | chromosome 6 open reading frame 155 | C6orf155 | EG2 | 4 | 0 | 7 | 2 | 0,1119520740 | 0,440114659 | 8,42E-05 | 0,002977773 |
| 238860_at | AA398043 | Hs.227457 | chromosome 6 open reading frame 130 | C6orf130 | EG2 | 1 | 3 | 2 | 7 | 0,1119520740 | 0,440114659 | 8,42E-05 | 0,002977773 |

| | | | | | | | | | | | | | |
|---|---|---|---|---|---|---|---|---|---|---|---|---|---|
| 239043_at | AA084273 | Hs.76561 | zinc finger protein 404 | ZNF404 | EG2 | 1 | 3 | 6 | 3 | 0,1119520740,440114659 | | 8,42E-05 | 0,002977773 |
| 239231_at | BE464819 | | | | EG2 | 3 | 1 | 3 | 6 | 0,1119520740,440114659 | | 8,42E-05 | 0,002977773 |
| 239587_at | AI686890 | | | | EG2 | 2 | 2 | 7 | 2 | 0,1119520740,440114659 | | 8,42E-05 | 0,002977773 |
| 240145_at | AW628059 | | | | EG2 | 1 | 3 | 3 | 6 | 0,1119520740,440114659 | | 8,42E-05 | 0,002977773 |
| 241484_x_at | AI668696 | | | | EG2 | 2 | 2 | 7 | 2 | 0,1119520740,440114659 | | 8,42E-05 | 0,002977773 |
| 241820_at | BF666241 | Hs.536537 | RAP1 interacting factor homolog (yeast) | RIF1 | EG2 | 2 | 2 | 5 | 4 | 0,1119520740,440114659 | | 8,42E-05 | 0,002977773 |
| 242052_at | AW979272 | Hs.505202 | bicaudal D homolog 1 (Drosophila) | BICD1 | EG2 | 0 | 4 | 6 | 3 | 0,1119520740,440114659 | | 8,42E-05 | 0,002977773 |
| 37892_at | J04177 | Hs.523446 | collagen, type XI, alpha 1 | COL11A1 | EG2 | 0 | 4 | 3 | 6 | 0,1119520740,440114659 | | 8,42E-05 | 0,002977773 |
| 229872_s_at | AA532655 | Hs.549388 | | LOC440667 | EG2 | 0 | 3 | 3 | 11 | 0,2913855570,799386187 | | 2,17E-10 | 0,001562433 |
| 231907_at | AK025877 | Hs.159472 | v-abl Abelson murine leukemia viral oncogene homol | ABL2 | EG2 | 2 | 1 | 9 | 5 | 0,2913855570,799386187 | | 2,17E-10 | 0,001562433 |
| 1555788_a_at | AF250311 | Hs.516826 | tribbles homolog 3 (Drosophila) | TRIB3 | EG2 | 1 | 2 | 8 | 5 | 0,2913855570,799386187 | | 4,56E-09 | 0,001562433 |
| 204222_s_at | NM_006851 | Hs.553516 | GLI pathogenesis-related 1 (glioma) | GLIPR1 | EG2 | 3 | 0 | 7 | 6 | 0,2913855570,799386187 | | 4,56E-09 | 0,001562433 |
| 211272_s_at | AF064771 | Hs.524488 | diacylglycerol kinase, alpha 80kDa | DGKA | EG2 | 2 | 1 | 5 | 8 | 0,2913855570,799386187 | | 4,56E-09 | 0,001562433 |
| 215307_at | AL109722 | | | | EG2 | 2 | 1 | 6 | 7 | 0,2913855570,799386187 | | 4,56E-09 | 0,001562433 |
| 217914_at | NM_017901 | Hs.524763 | two pore segment channel 1 | TPCN1 | EG2 | 1 | 2 | 5 | 8 | 0,2913855570,799386187 | | 4,56E-09 | 0,001562433 |
| 218953_s_at | NM_024028 | Hs.483796 | | MGC3265 | EG2 | 2 | 1 | 6 | 7 | 0,2913855570,799386187 | | 4,56E-09 | 0,001562433 |
| 227530_at | BF511276 | Hs.371240 | A kinase (PRKA) anchor protein (gravin) 12 | AKAP12 | EG2 | 1 | 2 | 7 | 6 | 0,2913855570,799386187 | | 4,56E-09 | 0,001562433 |
| 228726_at | AW512196 | Hs.381167 | serpin peptidase inhibitor, clade B (ovalbumin), mem | SERPINB1 | EG2 | 2 | 1 | 5 | 8 | 0,2913855570,799386187 | | 4,56E-09 | 0,001562433 |
| 231993_at | AK026784 | | | | EG2 | 2 | 1 | 4 | 9 | 0,2913855570,799386187 | | 4,56E-09 | 0,001562433 |
| 203157_s_at | AB020645 | Hs.116448 | glutaminase | GLS | EG2 | 3 | 0 | 8 | 4 | 0,2913855570,799386187 | | 7,43E-08 | 0,001562433 |
| 203708_at | NM_002600 | Hs.198072 | phosphodiesterase 4B, cAMP-specific (phosphodieste | PDE4B | EG2 | 1 | 2 | 5 | 7 | 0,2913855570,799386187 | | 7,43E-08 | 0,001562433 |
| 206342_x_at | NM_006123 | Hs.460960 | iduronate 2-sulfatase (Hunter syndrome) | IDS | EG2 | 2 | 1 | 8 | 4 | 0,2913855570,799386187 | | 7,43E-08 | 0,001562433 |
| 210511_s_at | M13436 | Hs.28792 | inhibin, beta A (activin A, activin AB alpha polypeptid | INHBA | EG2 | 2 | 1 | 9 | 3 | 0,2913855570,799386187 | | 7,43E-08 | 0,001562433 |
| 212311_at | AA522514 | Hs.479384 | | KIAA0746 | EG2 | 1 | 2 | 9 | 3 | 0,2913855570,799386187 | | 7,43E-08 | 0,001562433 |
| 219773_at | NM_016931 | Hs.371036 | NADPH oxidase 4 | NOX4 | EG2 | 1 | 2 | 4 | 8 | 0,2913855570,799386187 | | 7,43E-08 | 0,001562433 |
| 221019_s_at | NM_030781 | Hs.464422 | collectin sub-family member 12 | COLEC12 | EG2 | 0 | 3 | 2 | 10 | 0,2913855570,799386187 | | 7,43E-08 | 0,001562433 |
| 225919_s_at | AI832598 | Hs.493639 | chromosome 9 open reading frame 72 | C9orf72 | EG2 | 3 | 0 | 8 | 4 | 0,2913855570,799386187 | | 7,43E-08 | 0,001562433 |
| 235629_at | AI333596 | Hs.203717 | fibronectin 1 | FN1 | EG2 | 2 | 1 | 6 | 6 | 0,2913855570,799386187 | | 7,43E-08 | 0,001562433 |
| 235944_at | BF446673 | Hs.58877 | hemicentin 1 | HMCN1 | EG2 | 2 | 1 | 5 | 7 | 0,2913855570,799386187 | | 7,43E-08 | 0,001562433 |
| 238622_at | AW269686 | Hs.98643 | RAP2B, member of RAS oncogene family | RAP2B | EG2 | 2 | 1 | 4 | 8 | 0,2913855570,799386187 | | 7,43E-08 | 0,001562433 |
| 201141_at | NM_002510 | Hs.190495 | glycoprotein (transmembrane) nmb | GPNMB | EG2 | 1 | 2 | 9 | 2 | 0,2913855570,799386187 | | 9,59E-07 | 0,001562433 |
| 201360_at | NM_000099 | Hs.304682 | cystatin C (amyloid angiopathy and cerebral hemorrha | CST3 | EG2 | 0 | 3 | 5 | 6 | 0,2913855570,799386187 | | 9,59E-07 | 0,001562433 |
| 203108_at | NM_003979 | Hs.546325 | G protein-coupled receptor, family C, group 5, membe | GPRC5A | EG2 | 1 | 2 | 4 | 7 | 0,2913855570,799386187 | | 9,59E-07 | 0,001562433 |
| 203908_at | NM_003759 | Hs.5462 | solute carrier family 4, sodium bicarbonate cotranspor | SLC4A4 | EG2 | 2 | 1 | 9 | 2 | 0,2913855570,799386187 | | 9,59E-07 | 0,001562433 |
| 204678_s_at | U90065 | Hs.208544 | potassium channel, subfamily K, member 1 | KCNK1 | EG2 | 1 | 2 | 9 | 2 | 0,2913855570,799386187 | | 9,59E-07 | 0,001562433 |
| 210534_s_at | BC002944 | Hs.462445 | | EPPB9 | EG2 | 1 | 2 | 8 | 3 | 0,2913855570,799386187 | | 9,59E-07 | 0,001562433 |
| 213032_at | AI186739 | Hs.370359 | nuclear factor I/B | NFIB | EG2 | 2 | 1 | 3 | 8 | 0,2913855570,799386187 | | 9,59E-07 | 0,001562433 |
| 213703_at | W95043 | Hs.531822 | | LOC150759 | EG2 | 3 | 0 | 9 | 2 | 0,2913855570,799386187 | | 9,59E-07 | 0,001562433 |
| 214701_s_at | AJ276395 | Hs.203717 | fibronectin 1 | FN1 | EG2 | 2 | 1 | 5 | 6 | 0,2913855570,799386187 | | 9,59E-07 | 0,001562433 |
| 226492_at | AL036088 | Hs.511265 | sema domain, transmembrane domain (TM), and cyto | SEMA6D | EG2 | 0 | 3 | 8 | 3 | 0,2913855570,799386187 | | 9,59E-07 | 0,001562433 |
| 227955_s_at | BE670307 | | | | EG2 | 2 | 1 | 6 | 5 | 0,2913855570,799386187 | | 9,59E-07 | 0,001562433 |
| 228057_at | AA528140 | Hs.480378 | DNA-damage-inducible transcript 4-like | DDIT4L | EG2 | 2 | 1 | 9 | 2 | 0,2913855570,799386187 | | 9,59E-07 | 0,001562433 |
| 235457_at | AI769569 | Hs.428214 | mastermind-like 2 (Drosophila) | MAML2 | EG2 | 3 | 0 | 2 | 9 | 0,2913855570,799386187 | | 9,59E-07 | 0,001562433 |
| 237411_at | N71063 | Hs.344757, H | ADAM metallopeptidase with thrombospondin type 1 | ADAMTS6 | EG2 | 1 | 2 | 10 | 1 | 0,2913855570,799386187 | | 9,59E-07 | 0,001562433 |
| 1555526_a_at | AF403061 | Hs.496666 | septin 6 | SEPT6 | EG2 | 3 | 0 | 3 | 7 | 0,2913855570,799386187 | | 9,97E-06 | 0,001562433 |

| | | | | | | | | | | | | | |
|---|---|---|---|---|---|---|---|---|---|---|---|---|---|
| 1558199_at | W73431 | Hs.203717 | fibronectin 1 | FN1 | EG2 | 3 | 0 | 6 | 4 | 0,291385557 | 0,799386187 | 9,97E-06 | 0,001562433 |
| 1568609_s_at | AL118843 | Hs.381235, H | phosphodiesterase 4D interacting protein (myomegali | PDE4DIP | EG2 | 1 | 2 | 5 | 5 | 0,291385557 | 0,799386187 | 9,97E-06 | 0,001562433 |
| 1569472_s_at | BC026260 | Hs.368214 | tetratricopeptide repeat domain 3 | TTC3 | EG2 | 2 | 1 | 6 | 4 | 0,291385557 | 0,799386187 | 9,97E-06 | 0,001562433 |
| 205347_s_at | NM_021992 | Hs.56145 | thymosin-like 8 | TMSL8 | EG2 | 3 | 0 | 4 | 6 | 0,291385557 | 0,799386187 | 9,97E-06 | 0,001562433 |
| 205381_at | NM_005824 | Hs.550454 | leucine rich repeat containing 17 | LRRC17 | EG2 | 0 | 3 | 3 | 7 | 0,291385557 | 0,799386187 | 9,97E-06 | 0,001562433 |
| 205397_x_at | U76622 | Hs.36915, Hs | SMAD, mothers against DPP homolog 3 (Drosophila) | SMAD3 | EG2 | 1 | 2 | 4 | 6 | 0,291385557 | 0,799386187 | 9,97E-06 | 0,001562433 |
| 207357_s_at | NM_017540 | Hs.34421, Hs | UDP-N-acetyl-alpha-D-galactosamine:polypeptide N- | GALNT10 | EG2 | 1 | 2 | 8 | 2 | 0,291385557 | 0,799386187 | 9,97E-06 | 0,001562433 |
| 209406_at | AF095192 | Hs.55220 | BCL2-associated athanogene 2 | BAG2 | EG2 | 2 | 1 | 5 | 5 | 0,291385557 | 0,799386187 | 9,97E-06 | 0,001562433 |
| 209598_at | AB020690 | Hs.521466 | paraneoplastic antigen MA2 | PNMA2 | EG2 | 0 | 3 | 4 | 6 | 0,291385557 | 0,799386187 | 9,97E-06 | 0,001562433 |
| 209760_at | AL136932 | Hs.205572 | | KIAA0922 | EG2 | 2 | 1 | 1 | 9 | 0,291385557 | 0,799386187 | 9,97E-06 | 0,001562433 |
| 210102_at | BC001234 | Hs.152944 | loss of heterozygosity, 11, chromosomal region 2, gen | LOH11CR2A | EG2 | 3 | 0 | 4 | 6 | 0,291385557 | 0,799386187 | 9,97E-06 | 0,001562433 |
| 210869_s_at | M29277 | Hs.511397 | melanoma cell adhesion molecule | MCAM | EG2 | 2 | 1 | 3 | 7 | 0,291385557 | 0,799386187 | 9,97E-06 | 0,001562433 |
| 211376_s_at | BC005212 | Hs.258798 | chromosome 10 open reading frame 86 | C10orf86 | EG2 | 1 | 2 | 3 | 7 | 0,291385557 | 0,799386187 | 9,97E-06 | 0,001562433 |
| 214587_at | BE877796 | Hs.134830 | collagen, type VIII, alpha 1 | COL8A1 | EG2 | 2 | 1 | 5 | 5 | 0,291385557 | 0,799386187 | 9,97E-06 | 0,001562433 |
| 216048_s_at | AK023621 | Hs.445030 | Rho-related BTB domain containing 3 | RHOBTB3 | EG2 | 2 | 1 | 5 | 5 | 0,291385557 | 0,799386187 | 9,97E-06 | 0,001562433 |
| 218717_s_at | NM_018192 | Hs.374191 | leprecan-like 1 | LEPREL1 | EG2 | 2 | 1 | 8 | 2 | 0,291385557 | 0,799386187 | 9,97E-06 | 0,001562433 |
| 219506_at | NM_024579 | Hs.91283 | chromosome 1 open reading frame 54 | C1orf54 | EG2 | 0 | 3 | 5 | 5 | 0,291385557 | 0,799386187 | 9,97E-06 | 0,001562433 |
| 221679_s_at | AF225418 | Hs.476454 | abhydrolase domain containing 6 | ABHD6 | EG2 | 2 | 1 | 8 | 2 | 0,291385557 | 0,799386187 | 9,97E-06 | 0,001562433 |
| 225583_at | AL573637 | Hs.469561 | UDP-glucuronate decarboxylase 1 | UXS1 | EG2 | 2 | 1 | 3 | 7 | 0,291385557 | 0,799386187 | 9,97E-06 | 0,001562433 |
| 227074_at | AA524669 | Hs.348418 | down-regulator of transcription 1, TBP-binding (negati | DR1 | EG2 | 1 | 2 | 3 | 7 | 0,291385557 | 0,799386187 | 9,97E-06 | 0,001562433 |
| 227290_at | AU157881 | | | | EG2 | 2 | 1 | 5 | 5 | 0,291385557 | 0,799386187 | 9,97E-06 | 0,001562433 |
| 227415_at | BF109303 | Hs.527910 | diacylglycerol kinase, eta | DGKH | EG2 | 3 | 0 | 3 | 7 | 0,291385557 | 0,799386187 | 9,97E-06 | 0,001562433 |
| 227487_s_at | AI359165 | Hs.38449 | serpin peptidase inhibitor, clade E (nexin, plasminoge | SERPINE2 | EG2 | 1 | 2 | 9 | 1 | 0,291385557 | 0,799386187 | 9,97E-06 | 0,001562433 |
| 227802_at | AI075999 | Hs.518801 | | LOC441022 | EG2 | 2 | 1 | 5 | 5 | 0,291385557 | 0,799386187 | 9,97E-06 | 0,001562433 |
| 228158_at | AI623211 | | | | EG2 | 2 | 1 | 4 | 6 | 0,291385557 | 0,799386187 | 9,97E-06 | 0,001562433 |
| 228191_at | AA206016 | | | | EG2 | 3 | 0 | 7 | 3 | 0,291385557 | 0,799386187 | 9,97E-06 | 0,001562433 |
| 228242_at | BF055201 | | | | EG2 | 3 | 0 | 3 | 7 | 0,291385557 | 0,799386187 | 9,97E-06 | 0,001562433 |
| 228566_at | AA778694 | Hs.464912 | | P15RS | EG2 | 3 | 0 | 7 | 3 | 0,291385557 | 0,799386187 | 9,97E-06 | 0,001562433 |
| 229285_at | AI669749 | Hs.518545 | ribonuclease L (2',5'-oligoisoadenylate synthetase-de | RNASEL | EG2 | 1 | 2 | 2 | 8 | 0,291385557 | 0,799386187 | 9,97E-06 | 0,001562433 |
| 229838_at | AI377271 | Hs.128686 | nucleobindin 2 | NUCB2 | EG2 | 3 | 0 | 3 | 7 | 0,291385557 | 0,799386187 | 9,97E-06 | 0,001562433 |
| 230722_at | AI377043 | Hs.435309 | basonuclin 2 | BNC2 | EG2 | 3 | 0 | 6 | 4 | 0,291385557 | 0,799386187 | 9,97E-06 | 0,001562433 |
| 232060_at | AK000776 | | | | EG2 | 2 | 1 | 2 | 8 | 0,291385557 | 0,799386187 | 9,97E-06 | 0,001562433 |
| 235088_at | AI276663 | Hs.380920 | | LOC201725 | EG2 | 3 | 0 | 5 | 5 | 0,291385557 | 0,799386187 | 9,97E-06 | 0,001562433 |
| 235320_at | AL138043 | Hs.373801 | ADP-ribosylation factor-like 6 | ARL6 | EG2 | 2 | 1 | 7 | 3 | 0,291385557 | 0,799386187 | 9,97E-06 | 0,001562433 |
| 239336_at | BF109732 | Hs.164226 | thrombospondin 1 | THBS1 | EG2 | 1 | 2 | 4 | 6 | 0,291385557 | 0,799386187 | 9,97E-06 | 0,001562433 |
| 1553962_s_at | BI668074 | Hs.502876 | ras homolog gene family, member B | RHOB | EG2 | 2 | 1 | 6 | 3 | 0,291385557 | 0,799386187 | 8,42E-05 | 0,002977773 |
| 1554250_s_at | BC033812 | Hs.511015, H | tripartite motif-containing 50B | TRIM50B | EG2 | 1 | 2 | 2 | 7 | 0,291385557 | 0,799386187 | 8,42E-05 | 0,002977773 |
| 1555870_at | AK056658 | Hs.444729 | chromosome 1 open reading frame 188 | C1orf188 | EG2 | 1 | 2 | 2 | 7 | 0,291385557 | 0,799386187 | 8,42E-05 | 0,002977773 |
| 1555920_at | BU683892 | Hs.381189 | chromobox homolog 3 (HP1 gamma homolog, Drosop | CBX3 | EG2 | 1 | 2 | 5 | 4 | 0,291385557 | 0,799386187 | 8,42E-05 | 0,002977773 |
| 1556641_at | AK094547 | Hs.42834 | | FLJ37228 | EG2 | 0 | 3 | 0 | 9 | 0,291385557 | 0,799386187 | 8,42E-05 | 0,002977773 |
| 1557905_s_at | AL552534 | Hs.502328 | CD44 antigen (homing function and Indian blood grou | CD44 | EG2 | 1 | 2 | 5 | 4 | 0,291385557 | 0,799386187 | 8,42E-05 | 0,002977773 |
| 1560296_at | AV702787 | Hs.485616 | dystonin | DST | EG2 | 1 | 2 | 3 | 6 | 0,291385557 | 0,799386187 | 8,42E-05 | 0,002977773 |
| 1567224_at | U29113 | Hs.505924 | high mobility group AT-hook 2 | HMGA2 | EG2 | 2 | 1 | 7 | 2 | 0,291385557 | 0,799386187 | 8,42E-05 | 0,002977773 |
| 1568619_s_at | BC015343 | Hs.530899 | | LOC162073 | EG2 | 0 | 3 | 6 | 3 | 0,291385557 | 0,799386187 | 8,42E-05 | 0,002977773 |
| 200766_at | NM_001909 | Hs.121575, H | cathepsin D (lysosomal aspartyl peptidase) | CTSD | EG2 | 2 | 1 | 7 | 2 | 0,291385557 | 0,799386187 | 8,42E-05 | 0,002977773 |

| | | | | | | | | | | | | | |
|---|---|---|---|---|---|---|---|---|---|---|---|---|---|
| 201262_s_at | NM_001711 | Hs.821 | biglycan | BGN | EG2 | 1 | 2 | 5 | 4 | 0,291385557 | 0,799386187 | 8,42E-05 | 0,002977773 |
| 201325_s_at | NM_001423 | Hs.436298 | epithelial membrane protein 1 | EMP1 | EG2 | 3 | 0 | 5 | 4 | 0,291385557 | 0,799386187 | 8,42E-05 | 0,002977773 |
| 201425_at | NM_000690 | Hs.436437 | aldehyde dehydrogenase 2 family (mitochondrial) | ALDH2 | EG2 | 0 | 3 | 5 | 4 | 0,291385557 | 0,799386187 | 8,42E-05 | 0,002977773 |
| 201539_s_at | U29538 | Hs.435369 | four and a half LIM domains 1 | FHL1 | EG2 | 1 | 2 | 1 | 8 | 0,291385557 | 0,799386187 | 8,42E-05 | 0,002977773 |
| 201581_at | BF572868 | Hs.169358 | thioredoxin domain containing 13 | TXNDC13 | EG2 | 3 | 0 | 5 | 4 | 0,291385557 | 0,799386187 | 8,42E-05 | 0,002977773 |
| 201818_at | NM_024830 | Hs.368853 | | FLJ12443 | EG2 | 1 | 2 | 8 | 1 | 0,291385557 | 0,799386187 | 8,42E-05 | 0,002977773 |
| 201869_s_at | BF593932 | Hs.495656 | transducin (beta)-like 1X-linked | TBL1X | EG2 | 2 | 1 | 5 | 4 | 0,291385557 | 0,799386187 | 8,42E-05 | 0,002977773 |
| 202341_s_at | AA149745 | Hs.435711 | tripartite motif-containing 2 | TRIM2 | EG2 | 1 | 2 | 3 | 6 | 0,291385557 | 0,799386187 | 8,42E-05 | 0,002977773 |
| 202382_s_at | NM_005471 | Hs.278500 | glucosamine-6-phosphate deaminase 1 | GNPDA1 | EG2 | 1 | 2 | 6 | 3 | 0,291385557 | 0,799386187 | 8,42E-05 | 0,002977773 |
| 203153_at | NM_001548 | Hs.20315 | interferon-induced protein with tetratricopeptide rep | IFIT1 | EG2 | 1 | 2 | 5 | 4 | 0,291385557 | 0,799386187 | 8,42E-05 | 0,002977773 |
| 203217_s_at | NM_003896 | Hs.415117 | ST3 beta-galactoside alpha-2,3-sialyltransferase 5 | ST3GAL5 | EG2 | 1 | 2 | 6 | 3 | 0,291385557 | 0,799386187 | 8,42E-05 | 0,002977773 |
| 204084_s_at | AI911687 | Hs.30213 | ceroid-lipofuscinosis, neuronal 5 | CLN5 | EG2 | 2 | 1 | 3 | 6 | 0,291385557 | 0,799386187 | 8,42E-05 | 0,002977773 |
| 204137_at | NM_003272 | Hs.498160 | transmembrane 7 superfamily member 1 (upregulate | TM7SF1 | EG2 | 2 | 1 | 7 | 2 | 0,291385557 | 0,799386187 | 8,42E-05 | 0,002977773 |
| 204140_at | NM_003596 | Hs.421194 | tyrosylprotein sulfotransferase 1 | TPST1 | EG2 | 2 | 1 | 4 | 5 | 0,291385557 | 0,799386187 | 8,42E-05 | 0,002977773 |
| 204493_at | NM_001196 | Hs.474150 | BH3 interacting domain death agonist | BID | EG2 | 1 | 2 | 3 | 6 | 0,291385557 | 0,799386187 | 8,42E-05 | 0,002977773 |
| 205407_at | NM_021111 | Hs.388918 | reversion-inducing-cysteine-rich protein with kazal m | RECK | EG2 | 1 | 2 | 2 | 7 | 0,291385557 | 0,799386187 | 8,42E-05 | 0,002977773 |
| 205540_s_at | NM_016656 | Hs.50282 | Ras-related GTP binding B | RRAGB | EG2 | 1 | 2 | 3 | 6 | 0,291385557 | 0,799386187 | 8,42E-05 | 0,002977773 |
| 206777_s_at | NM_000496 | Hs.373074 | crystallin, beta B2 | CRYBB2 | EG2 | 0 | 3 | 8 | 1 | 0,291385557 | 0,799386187 | 8,42E-05 | 0,002977773 |
| 206907_at | NM_003811 | Hs.1524 | tumor necrosis factor (ligand) superfamily, member 9 | TNFSF9 | EG2 | 2 | 1 | 6 | 3 | 0,291385557 | 0,799386187 | 8,42E-05 | 0,002977773 |
| 208230_s_at | NM_013960 | Hs.453951 | neuregulin 1 | NRG1 | EG2 | 2 | 1 | 9 | 0 | 0,291385557 | 0,799386187 | 8,42E-05 | 0,002977773 |
| 209035_at | M69148 | Hs.82045 | midkine (neurite growth-promoting factor 2) | MDK | EG2 | 0 | 3 | 1 | 8 | 0,291385557 | 0,799386187 | 8,42E-05 | 0,002977773 |
| 209146_at | AV704962 | Hs.105269 | sterol-C4-methyl oxidase-like | SC4MOL | EG2 | 2 | 1 | 7 | 2 | 0,291385557 | 0,799386187 | 8,42E-05 | 0,002977773 |
| 209277_at | AL574096 | Hs.438231 | tissue factor pathway inhibitor 2 | TFPI2 | EG2 | 2 | 1 | 4 | 5 | 0,291385557 | 0,799386187 | 8,42E-05 | 0,002977773 |
| 209373_at | BC003179 | Hs.185055 | mal, T-cell differentiation protein-like | MALL | EG2 | 2 | 1 | 5 | 4 | 0,291385557 | 0,799386187 | 8,42E-05 | 0,002977773 |
| 209409_at | D86962 | Hs.164060 | growth factor receptor-bound protein 10 | GRB10 | EG2 | 2 | 1 | 3 | 6 | 0,291385557 | 0,799386187 | 8,42E-05 | 0,002977773 |
| 209537_at | AF000416 | Hs.357637 | exostoses (multiple)-like 2 | EXTL2 | EG2 | 3 | 0 | 9 | 0 | 0,291385557 | 0,799386187 | 8,42E-05 | 0,002977773 |
| 209758_s_at | U37283 | Hs.512842 | microfibrillar associated protein 5 | MFAP5 | EG2 | 0 | 3 | 3 | 6 | 0,291385557 | 0,799386187 | 8,42E-05 | 0,002977773 |
| 210059_s_at | BC000433 | Hs.178695 | mitogen-activated protein kinase 13 | MAPK13 | EG2 | 0 | 3 | 7 | 2 | 0,291385557 | 0,799386187 | 8,42E-05 | 0,002977773 |
| 210732_s_at | AF342816 | Hs.4082 | lectin, galactoside-binding, soluble, 8 (galectin 8) | LGALS8 | EG2 | 2 | 1 | 7 | 2 | 0,291385557 | 0,799386187 | 8,42E-05 | 0,002977773 |
| 210916_s_at | AF098641 | Hs.502328 | CD44 antigen (homing function and Indian blood grou | CD44 | EG2 | 2 | 1 | 6 | 3 | 0,291385557 | 0,799386187 | 8,42E-05 | 0,002977773 |
| 211208_s_at | AB039327 | Hs.495984 | calcium/calmodulin-dependent serine protein kinase | CASK | EG2 | 2 | 1 | 1 | 8 | 0,291385557 | 0,799386187 | 8,42E-05 | 0,002977773 |
| 211825_s_at | AF327066 | Hs.374477 | Ewing sarcoma breakpoint region 1 | EWSR1 | EG2 | 3 | 0 | 5 | 4 | 0,291385557 | 0,799386187 | 8,42E-05 | 0,002977773 |
| 211980_at | AI922605 | Hs.17441 | collagen, type IV, alpha 1 | COL4A1 | EG2 | 1 | 2 | 4 | 5 | 0,291385557 | 0,799386187 | 8,42E-05 | 0,002977773 |
| 212959_s_at | AK001821 | Hs.46850 | N-acetylglucosamine-1-phosphate transferase, alpha | GNPTAB | EG2 | 2 | 1 | 8 | 1 | 0,291385557 | 0,799386187 | 8,42E-05 | 0,002977773 |
| 213764_s_at | AW665892 | Hs.512842 | microfibrillar associated protein 5 | MFAP5 | EG2 | 1 | 2 | 3 | 6 | 0,291385557 | 0,799386187 | 8,42E-05 | 0,002977773 |
| 214250_at | AI337584 | Hs.523873 | nuclear mitotic apparatus protein 1 | NUMA1 | EG2 | 1 | 2 | 2 | 7 | 0,291385557 | 0,799386187 | 8,42E-05 | 0,002977773 |
| 214505_s_at | AF220153 | Hs.435369 | four and a half LIM domains 1 | FHL1 | EG2 | 1 | 2 | 1 | 8 | 0,291385557 | 0,799386187 | 8,42E-05 | 0,002977773 |
| 214702_at | AJ276395 | Hs.203717 | fibronectin 1 | FN1 | EG2 | 1 | 2 | 4 | 5 | 0,291385557 | 0,799386187 | 8,42E-05 | 0,002977773 |
| 214723_x_at | AB046861 | Hs.503490, H | KIAA1641 | KIAA1641 | EG2 | 1 | 2 | 6 | 3 | 0,291385557 | 0,799386187 | 8,42E-05 | 0,002977773 |
| 214927_at | AL359052 | Hs.508597 | integrin, beta-like 1 (with EGF-like repeat domains) | ITGBL1 | EG2 | 2 | 1 | 4 | 5 | 0,291385557 | 0,799386187 | 8,42E-05 | 0,002977773 |
| 215775_at | BF084105 | Hs.164226 | thrombospondin 1 | THBS1 | EG2 | 0 | 3 | 3 | 6 | 0,291385557 | 0,799386187 | 8,42E-05 | 0,002977773 |
| 217785_s_at | NM_006555 | Hs.520794 | | YKT6 | EG2 | 2 | 1 | 6 | 3 | 0,291385557 | 0,799386187 | 8,42E-05 | 0,002977773 |
| 218051_s_at | NM_022908 | Hs.84753 | | FLJ12442 | EG2 | 1 | 2 | 3 | 6 | 0,291385557 | 0,799386187 | 8,42E-05 | 0,002977773 |
| 218934_s_at | NM_014424 | Hs.502612 | heat shock 27kDa protein family, member 7 (cardiovas | HSPB7 | EG2 | 2 | 1 | 4 | 5 | 0,291385557 | 0,799386187 | 8,42E-05 | 0,002977773 |
| 219501_at | NM_017993 | Hs.128258 | | FLJ10094 | EG2 | 3 | 0 | 7 | 2 | 0,291385557 | 0,799386187 | 8,42E-05 | 0,002977773 |

| | | | | | | | | | | | | | | |
|---|---|---|---|---|---|---|---|---|---|---|---|---|---|---|
| 220615_s_at | NM_018099 | Hs.298851 | male sterility domain containing 1 | MLSTD1 | EG2 | 1 | 2 | 1 | 8 | 0,2913855570 | 0,799386187 | 8,42E-05 | 0,002977773 |
| 221142_s_at | NM_018441 | Hs.281680 | peroxisomal trans-2-enoyl-CoA reductase | PECR | EG2 | 0 | 3 | 2 | 7 | 0,2913855570 | 0,799386187 | 8,42E-05 | 0,002977773 |
| 221882_s_at | AI636233 | Hs.288940 | transmembrane protein 8 (five membrane-spanning d | TMEM8 | EG2 | 0 | 3 | 4 | 5 | 0,2913855570 | 0,799386187 | 8,42E-05 | 0,002977773 |
| 223271_s_at | AF161543 | Hs.497967 | CTD (carboxy-terminal domain, RNA polymerase II, po | CTDSPL2 | EG2 | 3 | 0 | 4 | 5 | 0,2913855570 | 0,799386187 | 8,42E-05 | 0,002977773 |
| 223287_s_at | AF146696 | Hs.431498 | forkhead box P1 | FOXP1 | EG2 | 0 | 3 | 4 | 5 | 0,2913855570 | 0,799386187 | 8,42E-05 | 0,002977773 |
| 223374_s_at | AF154848 | Hs.418062 | UDP-Gal:betaGlcNAc beta 1,3-galactosyltransferase, p | B3GALT3 | EG2 | 2 | 1 | 6 | 3 | 0,2913855570 | 0,799386187 | 8,42E-05 | 0,002977773 |
| 223395_at | AB056106 | Hs.477015 | ABI gene family, member 3 (NESH) binding protein | ABI3BP | EG2 | 0 | 3 | 7 | 2 | 0,2913855570 | 0,799386187 | 8,42E-05 | 0,002977773 |
| 224755_at | BE621524 | Hs.500674 | | SMBP | EG2 | 3 | 0 | 4 | 5 | 0,2913855570 | 0,799386187 | 8,42E-05 | 0,002977773 |
| 225140_at | BF438116 | Hs.298658 | Kruppel-like factor 3 (basic) | KLF3 | EG2 | 2 | 1 | 8 | 1 | 0,2913855570 | 0,799386187 | 8,42E-05 | 0,002977773 |
| 225579_at | AL516202 | Hs.274415 | PQ loop repeat containing 3 | PQLC3 | EG2 | 0 | 3 | 4 | 5 | 0,2913855570 | 0,799386187 | 8,42E-05 | 0,002977773 |
| 225640_at | AA875998 | Hs.446271 | | LOC401504 | EG2 | 1 | 2 | 6 | 3 | 0,2913855570 | 0,799386187 | 8,42E-05 | 0,002977773 |
| 225742_at | W93501 | Hs.497492 | Mdm4, transformed 3T3 cell double minute 4, p53 bin | MDM4 | EG2 | 2 | 1 | 5 | 4 | 0,2913855570 | 0,799386187 | 8,42E-05 | 0,002977773 |
| 225871_at | BF680588 | Hs.489051 | six transmembrane epithelial antigen of the prostate | STEAP2 | EG2 | 1 | 2 | 7 | 2 | 0,2913855570 | 0,799386187 | 8,42E-05 | 0,002977773 |
| 226534_at | AI446414 | Hs.1048 | KIT ligand | KITLG | EG2 | 2 | 1 | 4 | 5 | 0,2913855570 | 0,799386187 | 8,42E-05 | 0,002977773 |
| 226908_at | AI627704 | Hs.253736 | leucine-rich repeats and immunoglobulin-like domain | LRIG3 | EG2 | 2 | 1 | 3 | 6 | 0,2913855570 | 0,799386187 | 8,42E-05 | 0,002977773 |
| 226912_at | AW003367 | Hs.21902 | zinc finger, DHHC-type containing 23 | ZDHHC23 | EG2 | 2 | 1 | 7 | 2 | 0,2913855570 | 0,799386187 | 8,42E-05 | 0,002977773 |
| 227408_s_at | AW008976 | Hs.369091 | sorting nexin 25 | SNX25 | EG2 | 3 | 0 | 4 | 5 | 0,2913855570 | 0,799386187 | 8,42E-05 | 0,002977773 |
| 227525_at | AA058770 | Hs.131673 | glucocorticoid induced transcript 1 | GLCCI1 | EG2 | 3 | 0 | 2 | 7 | 0,2913855570 | 0,799386187 | 8,42E-05 | 0,002977773 |
| 228314_at | BE877357 | | | | EG2 | 3 | 0 | 5 | 4 | 0,2913855570 | 0,799386187 | 8,42E-05 | 0,002977773 |
| 228613_at | BF183535 | Hs.531642 | RAB11 family interacting protein 3 (class II) | RAB11FIP3 | EG2 | 3 | 0 | 5 | 4 | 0,2913855570 | 0,799386187 | 8,42E-05 | 0,002977773 |
| 229010_at | AI807026 | Hs.504096 | Cas-Br-M (murine) ecotropic retroviral transforming s | CBL | EG2 | 2 | 1 | 2 | 7 | 0,2913855570 | 0,799386187 | 8,42E-05 | 0,002977773 |
| 229512_at | BE464337 | | | | EG2 | 2 | 1 | 3 | 6 | 0,2913855570 | 0,799386187 | 8,42E-05 | 0,002977773 |
| 230000_at | AI523817 | Hs.195642 | chromosome 17 open reading frame 27 | C17orf27 | EG2 | 1 | 2 | 4 | 5 | 0,2913855570 | 0,799386187 | 8,42E-05 | 0,002977773 |
| 230165_at | N31731 | Hs.44269 | shugoshin-like 2 (S. pombe) | SGOL2 | EG2 | 2 | 1 | 4 | 5 | 0,2913855570 | 0,799386187 | 8,42E-05 | 0,002977773 |
| 230424_at | AU144860 | Hs.483067 | chromosome 5 open reading frame 13 | C5orf13 | EG2 | 0 | 3 | 6 | 3 | 0,2913855570 | 0,799386187 | 8,42E-05 | 0,002977773 |
| 234863_x_at | AK026197 | Hs.520506 | F-box protein 5 | FBXO5 | EG2 | 2 | 1 | 6 | 3 | 0,2913855570 | 0,799386187 | 8,42E-05 | 0,002977773 |
| 235147_at | R56118 | Hs.528187 | | LOC440928 | EG2 | 2 | 1 | 7 | 2 | 0,2913855570 | 0,799386187 | 8,42E-05 | 0,002977773 |
| 235287_at | AW192700 | Hs.119882 | cyclin-dependent kinase 6 | CDK6 | EG2 | 1 | 2 | 3 | 6 | 0,2913855570 | 0,799386187 | 8,42E-05 | 0,002977773 |
| 235746_s_at | BE048919 | Hs.410477 | phospholipase A2 receptor 1, 180kDa | PLA2R1 | EG2 | 0 | 3 | 2 | 7 | 0,2913855570 | 0,799386187 | 8,42E-05 | 0,002977773 |
| 238722_x_at | AI460037 | Hs.324271 | | NAPE-PLD | EG2 | 1 | 2 | 3 | 6 | 0,2913855570 | 0,799386187 | 8,42E-05 | 0,002977773 |
| 241893_at | BE927766 | | | | EG2 | 2 | 1 | 3 | 6 | 0,2913855570 | 0,799386187 | 8,42E-05 | 0,002977773 |
| 243507_s_at | AV715251 | Hs.529340 | | FLJ25067 | EG2 | 2 | 1 | 2 | 7 | 0,2913855570 | 0,799386187 | 8,42E-05 | 0,002977773 |
| 243982_at | AA455180 | Hs.174682 | BTB (POZ) domain containing 5 | BTBD5 | EG2 | 2 | 1 | 7 | 2 | 0,2913855570 | 0,799386187 | 8,42E-05 | 0,002977773 |
| 244669_at | T16443 | Hs.292457 | | LOC441164 | EG2 | 3 | 0 | 2 | 7 | 0,2913855570 | 0,799386187 | 8,42E-05 | 0,002977773 |
| 212677_s_at | BG530481 | Hs.310645 | RAB1A, member RAS oncogene family | RAB1A | EG2 | 0 | 2 | 4 | 10 | 0,5783259580 | 0,896050827 | 2,17E-10 | 0,001562433 |
| 209217_s_at | BC000464 | Hs.156701 | WD repeat domain 45 | WDR45 | EG2 | 1 | 1 | 8 | 5 | 0,5783259580 | 0,896050827 | 4,56E-09 | 0,001562433 |
| 227140_at | AI343467 | Hs.28792 | inhibin, beta A (activin A, activin AB alpha polypeptid | INHBA | EG2 | 1 | 1 | 9 | 4 | 0,5783259580 | 0,896050827 | 4,56E-09 | 0,001562433 |
| 202625_at | AI356412 | Hs.491767 | v-yes-1 Yamaguchi sarcoma viral related oncogene ho | LYN | EG2 | 2 | 0 | 6 | 6 | 0,5783259580 | 0,896050827 | 7,43E-08 | 0,001562433 |
| 205463_s_at | NM_002607 | Hs.376032, H | platelet-derived growth factor alpha polypeptide | PDGFA | EG2 | 1 | 1 | 2 | 10 | 0,5783259580 | 0,896050827 | 7,43E-08 | 0,001562433 |
| 1557242_at | AI769729 | Hs.130838 | | LOC285835 | EG2 | 1 | 1 | 7 | 4 | 0,5783259580 | 0,896050827 | 9,59E-07 | 0,001562433 |
| 201578_at | NM_005397 | Hs.16426 | podocalyxin-like | PODXL | EG2 | 0 | 2 | 6 | 5 | 0,5783259580 | 0,896050827 | 9,59E-07 | 0,001562433 |
| 202975_s_at | N21138 | Hs.445030 | Rho-related BTB domain containing 3 | RHOBTB3 | EG2 | 1 | 1 | 7 | 4 | 0,5783259580 | 0,896050827 | 9,59E-07 | 0,001562433 |
| 204797_s_at | NM_004434 | Hs.12451 | echinoderm microtubule associated protein like 1 | EML1 | EG2 | 2 | 0 | 5 | 6 | 0,5783259580 | 0,896050827 | 9,59E-07 | 0,001562433 |
| 205634_x_at | NM_024298 | Hs.467279 | leukocyte receptor cluster (LRC) member 4 | LENG4 | EG2 | 1 | 1 | 8 | 3 | 0,5783259580 | 0,896050827 | 9,59E-07 | 0,001562433 |
| 206059_at | NM_003430 | Hs.531632 | zinc finger protein 91 (HPF7, HTF10) | ZNF91 | EG2 | 2 | 0 | 7 | 4 | 0,5783259580 | 0,896050827 | 9,59E-07 | 0,001562433 |

| | | | | | | | | | | | | | |
|---|---|---|---|---|---|---|---|---|---|---|---|---|---|
| 209940_at | AF083068 | Hs.271742 | poly (ADP-ribose) polymerase family, member 3 | PARP3 | EG2 | 0 | 2 | 4 | 7 | 0,578325958 | 0,896050827 | 9,59E-07 | 0,001562433 |
| 213260_at | AU145890 | Hs.348883 | forkhead box C1 | FOXC1 | EG2 | 2 | 0 | 1 | 10 | 0,578325958 | 0,896050827 | 9,59E-07 | 0,001562433 |
| 213572_s_at | AI554300 | Hs.381167 | serpin peptidase inhibitor, clade B (ovalbumin), mem | SERPINB1 | EG2 | 0 | 2 | 5 | 6 | 0,578325958 | 0,896050827 | 9,59E-07 | 0,001562433 |
| 219210_s_at | NM_016530 | Hs.389733 | RAB8B, member RAS oncogene family | RAB8B | EG2 | 2 | 0 | 9 | 2 | 0,578325958 | 0,896050827 | 9,59E-07 | 0,001562433 |
| 238478_at | H97386 | Hs.435309 | basonuclin 2 | BNC2 | EG2 | 2 | 0 | 5 | 6 | 0,578325958 | 0,896050827 | 9,59E-07 | 0,001562433 |
| 1554097_a_at | BC021861 | Hs.458096 | | LOC554202 | EG2 | 1 | 1 | 6 | 4 | 0,578325958 | 0,896050827 | 9,97E-06 | 0,001562433 |
| 201538_s_at | NM_004090 | Hs.181046 | dual specificity phosphatase 3 (vaccinia virus phospha | DUSP3 | EG2 | 1 | 1 | 9 | 1 | 0,578325958 | 0,896050827 | 9,97E-06 | 0,001562433 |
| 202037_s_at | NM_003012 | Hs.213424 | secreted frizzled-related protein 1 | SFRP1 | EG2 | 1 | 1 | 7 | 3 | 0,578325958 | 0,896050827 | 9,97E-06 | 0,001562433 |
| 202510_s_at | NM_006291 | Hs.525607 | tumor necrosis factor, alpha-induced protein 2 | TNFAIP2 | EG2 | 0 | 2 | 5 | 5 | 0,578325958 | 0,896050827 | 9,97E-06 | 0,001562433 |
| 202627_s_at | AL574210 | Hs.414795 | serpin peptidase inhibitor, clade E (nexin, plasminoge | SERPINE1 | EG2 | 2 | 0 | 7 | 3 | 0,578325958 | 0,896050827 | 9,97E-06 | 0,001562433 |
| 203385_at | NM_001345 | Hs.524488 | diacylglycerol kinase, alpha 80kDa | DGKA | EG2 | 0 | 2 | 3 | 7 | 0,578325958 | 0,896050827 | 9,97E-06 | 0,001562433 |
| 203504_s_at | NM_005502 | Hs.429294 | ATP-binding cassette, sub-family A (ABC1), member 1 | ABCA1 | EG2 | 1 | 1 | 3 | 7 | 0,578325958 | 0,896050827 | 9,97E-06 | 0,001562433 |
| 203579_s_at | AI660619 | Hs.334848 | solute carrier family 7, member 6 opposite strand | SLC7A6OS | EG2 | 1 | 1 | 5 | 5 | 0,578325958 | 0,896050827 | 9,97E-06 | 0,001562433 |
| 203741_s_at | NM_001114 | Hs.513578 | adenylate cyclase 7 | ADCY7 | EG2 | 0 | 2 | 4 | 6 | 0,578325958 | 0,896050827 | 9,97E-06 | 0,001562433 |
| 203951_at | NM_001299 | Hs.465929 | calponin 1, basic, smooth muscle | CNN1 | EG2 | 1 | 1 | 2 | 8 | 0,578325958 | 0,896050827 | 9,97E-06 | 0,001562433 |
| 204925_at | NM_004937 | Hs.187667 | cystinosis, nephropathic | CTNS | EG2 | 0 | 2 | 7 | 3 | 0,578325958 | 0,896050827 | 9,97E-06 | 0,001562433 |
| 206396_at | NM_004170 | Hs.444915 | solute carrier family 1 (neuronal/epithelial high affini | SLC1A1 | EG2 | 1 | 1 | 7 | 3 | 0,578325958 | 0,896050827 | 9,97E-06 | 0,001562433 |
| 218000_s_at | NM_007350 | Hs.484885 | pleckstrin homology-like domain, family A, member 1 | PHLDA1 | EG2 | 2 | 0 | 9 | 1 | 0,578325958 | 0,896050827 | 9,97E-06 | 0,001562433 |
| 221577_x_at | AF003934 | Hs.515258 | growth differentiation factor 15 | GDF15 | EG2 | 2 | 0 | 9 | 1 | 0,578325958 | 0,896050827 | 9,97E-06 | 0,001562433 |
| 222719_s_at | AB033831 | Hs.148162 | platelet derived growth factor C | PDGFC | EG2 | 2 | 0 | 8 | 2 | 0,578325958 | 0,896050827 | 9,97E-06 | 0,001562433 |
| 223734_at | AF329088 | Hs.154140 | | OSAP | EG2 | 1 | 1 | 4 | 6 | 0,578325958 | 0,896050827 | 9,97E-06 | 0,001562433 |
| 223789_s_at | AF116627 | Hs.485449 | GTP binding protein 2 | GTPBP2 | EG2 | 1 | 1 | 5 | 5 | 0,578325958 | 0,896050827 | 9,97E-06 | 0,001562433 |
| 224942_at | BG434272 | Hs.494928 | pregnancy-associated plasma protein A, pappalysin 1 | PAPPA | EG2 | 2 | 0 | 7 | 3 | 0,578325958 | 0,896050827 | 9,97E-06 | 0,001562433 |
| 224964_s_at | AK026424 | Hs.187772 | guanine nucleotide binding protein (G protein), gam | GNG2 | EG2 | 2 | 0 | 5 | 5 | 0,578325958 | 0,896050827 | 9,97E-06 | 0,001562433 |
| 226278_at | AI150224 | Hs.349096 | | DKFZp313A2432 | EG2 | 0 | 2 | 4 | 6 | 0,578325958 | 0,896050827 | 9,97E-06 | 0,001562433 |
| 226810_at | BE500942 | Hs.368337 | chromosome 6 open reading frame 155 | C6orf155 | EG2 | 2 | 0 | 8 | 2 | 0,578325958 | 0,896050827 | 9,97E-06 | 0,001562433 |
| 227396_at | AI631833 | | | | EG2 | 2 | 0 | 7 | 3 | 0,578325958 | 0,896050827 | 9,97E-06 | 0,001562433 |
| 229011_at | AA150501 | | | | EG2 | 2 | 0 | 6 | 4 | 0,578325958 | 0,896050827 | 9,97E-06 | 0,001562433 |
| 231067_s_at | BF114967 | Hs.371240 | A kinase (PRKA) anchor protein (gravin) 12 | AKAP12 | EG2 | 1 | 1 | 4 | 6 | 0,578325958 | 0,896050827 | 9,97E-06 | 0,001562433 |
| 232790_at | AI744580 | | | | EG2 | 1 | 1 | 3 | 7 | 0,578325958 | 0,896050827 | 9,97E-06 | 0,001562433 |
| 235311_at | BG110260 | Hs.390838 | FK506 binding protein 14, 22 kDa | FKBP14 | EG2 | 2 | 0 | 8 | 2 | 0,578325958 | 0,896050827 | 9,97E-06 | 0,001562433 |
| 1557080_s_at | AI753143 | Hs.508597 | integrin, beta-like 1 (with EGF-like repeat domains) | ITGBL1 | EG2 | 1 | 1 | 3 | 6 | 0,578325958 | 0,896050827 | 8,42E-05 | 0,002977773 |
| 201185_at | NM_002775 | Hs.501280 | HtrA serine peptidase 1 | HTRA1 | EG2 | 0 | 2 | 5 | 4 | 0,578325958 | 0,896050827 | 8,42E-05 | 0,002977773 |
| 201615_x_at | AI685060 | Hs.490203 | caldesmon 1 | CALD1 | EG2 | 2 | 0 | 3 | 6 | 0,578325958 | 0,896050827 | 8,42E-05 | 0,002977773 |
| 201668_x_at | AW163148 | Hs.519909 | myristoylated alanine-rich protein kinase C substrate | MARCKS | EG2 | 0 | 2 | 3 | 6 | 0,578325958 | 0,896050827 | 8,42E-05 | 0,002977773 |
| 202948_at | NM_000877 | Hs.516249 | interleukin 1 receptor, type I | IL1R1 | EG2 | 0 | 2 | 2 | 7 | 0,578325958 | 0,896050827 | 8,42E-05 | 0,002977773 |
| 203354_s_at | AW117368 | Hs.434255 | pleckstrin and Sec7 domain containing 3 | PSD3 | EG2 | 2 | 0 | 2 | 7 | 0,578325958 | 0,896050827 | 8,42E-05 | 0,002977773 |
| 203424_s_at | AW157548 | Hs.369982, H | insulin-like growth factor binding protein 5 | IGFBP5 | EG2 | 2 | 0 | 3 | 6 | 0,578325958 | 0,896050827 | 8,42E-05 | 0,002977773 |
| 203827_at | NM_017983 | Hs.463964 | | WIPI49 | EG2 | 0 | 2 | 6 | 3 | 0,578325958 | 0,896050827 | 8,42E-05 | 0,002977773 |
| 203879_at | U86453 | Hs.518451 | phosphoinositide-3-kinase, catalytic, delta polypeptid | PIK3CD | EG2 | 0 | 2 | 3 | 6 | 0,578325958 | 0,896050827 | 8,42E-05 | 0,002977773 |
| 204807_at | BF224146 | Hs.216386 | transmembrane protein 5 | TMEM5 | EG2 | 1 | 1 | 3 | 6 | 0,578325958 | 0,896050827 | 8,42E-05 | 0,002977773 |
| 204979_s_at | NM_007341 | Hs.473847 | SH3 domain binding glutamic acid-rich protein | SH3BGR | EG2 | 1 | 1 | 8 | 1 | 0,578325958 | 0,896050827 | 8,42E-05 | 0,002977773 |
| 205234_at | NM_004696 | Hs.351306 | solute carrier family 16 (monocarboxylic acid transpor | SLC16A4 | EG2 | 0 | 2 | 4 | 5 | 0,578325958 | 0,896050827 | 8,42E-05 | 0,002977773 |
| 205741_s_at | NM_001392 | Hs.58919 | dystrobrevin, alpha | DTNA | EG2 | 2 | 0 | 5 | 4 | 0,578325958 | 0,896050827 | 8,42E-05 | 0,002977773 |
| 205925_s_at | NM_002867 | Hs.123072 | RAB3B, member RAS oncogene family | RAB3B | EG2 | 2 | 0 | 6 | 3 | 0,578325958 | 0,896050827 | 8,42E-05 | 0,002977773 |

| | | | | | | | | | | | | | |
|---|---|---|---|---|---|---|---|---|---|---|---|---|---|
| 207264_at | NM_016657 | Hs.554798 | KDEL (Lys-Asp-Glu-Leu) endoplasmic reticulum protei | KDELR3 | EG2 | 2 | 0 | 5 | 4 | 0,578325958 | 0,896050827 | 8,42E-05 | 0,002977773 |
| 208107_s_at | NM_030941 | Hs.177926 | | LOC81691 | EG2 | 1 | 1 | 5 | 4 | 0,578325958 | 0,896050827 | 8,42E-05 | 0,002977773 |
| 208191_x_at | NM_002780 | Hs.466860 | pregnancy specific beta-1-glycoprotein 4 | PSG4 | EG2 | 0 | 2 | 6 | 3 | 0,578325958 | 0,896050827 | 8,42E-05 | 0,002977773 |
| 209216_at | BC000464 | Hs.156701 | WD repeat domain 45 | WDR45 | EG2 | 0 | 2 | 3 | 6 | 0,578325958 | 0,896050827 | 8,42E-05 | 0,002977773 |
| 211340_s_at | M28882 | Hs.511397 | melanoma cell adhesion molecule | MCAM | EG2 | 0 | 2 | 2 | 7 | 0,578325958 | 0,896050827 | 8,42E-05 | 0,002977773 |
| 211713_x_at | BC005832 | Hs.81892 | KIAA0101 | KIAA0101 | EG2 | 2 | 0 | 3 | 6 | 0,578325958 | 0,896050827 | 8,42E-05 | 0,002977773 |
| 212386_at | BF592782 | Hs.200285 | transcription factor 4 | TCF4 | EG2 | 1 | 1 | 4 | 5 | 0,578325958 | 0,896050827 | 8,42E-05 | 0,002977773 |
| 213206_at | AW149492 | Hs.463278 | golgi SNAP receptor complex member 2 | GOSR2 | EG2 | 0 | 2 | 3 | 6 | 0,578325958 | 0,896050827 | 8,42E-05 | 0,002977773 |
| 213765_at | AW665892 | Hs.512842 | microfibrillar associated protein 5 | MFAP5 | EG2 | 0 | 2 | 3 | 6 | 0,578325958 | 0,896050827 | 8,42E-05 | 0,002977773 |
| 213959_s_at | BF515597 | Hs.298382 | | KIAA1005 | EG2 | 2 | 0 | 5 | 4 | 0,578325958 | 0,896050827 | 8,42E-05 | 0,002977773 |
| 214151_s_at | AU144243 | Hs.126115 | phosphatidylinositol glycan, class B | PIGB | EG2 | 2 | 0 | 5 | 4 | 0,578325958 | 0,896050827 | 8,42E-05 | 0,002977773 |
| 214581_x_at | BE568134 | Hs.443577 | tumor necrosis factor receptor superfamily, member 2 | TNFRSF21 | EG2 | 2 | 0 | 4 | 5 | 0,578325958 | 0,896050827 | 8,42E-05 | 0,002977773 |
| 214724_at | AF070621 | Hs.116796 | DIX domain containing 1 | DIXDC1 | EG2 | 1 | 1 | 3 | 6 | 0,578325958 | 0,896050827 | 8,42E-05 | 0,002977773 |
| 217502_at | BE888744 | Hs.437609 | interferon-induced protein with tetratricopeptide rep | IFIT2 | EG2 | 2 | 0 | 5 | 4 | 0,578325958 | 0,896050827 | 8,42E-05 | 0,002977773 |
| 218284_at | NM_015400 | Hs.36915, Hs | SMAD, mothers against DPP homolog 3 (Drosophila) | SMAD3 | EG2 | 0 | 2 | 4 | 5 | 0,578325958 | 0,896050827 | 8,42E-05 | 0,002977773 |
| 218665_at | NM_012193 | Hs.19545 | frizzled homolog 4 (Drosophila) | FZD4 | EG2 | 0 | 2 | 3 | 6 | 0,578325958 | 0,896050827 | 8,42E-05 | 0,002977773 |
| 218986_s_at | NM_017631 | Hs.481141 | | FLJ20035 | EG2 | 0 | 2 | 2 | 7 | 0,578325958 | 0,896050827 | 8,42E-05 | 0,002977773 |
| 219703_at | NM_018365 | Hs.444483 | meiosis-specific nuclear structural 1 | MNS1 | EG2 | 2 | 0 | 4 | 5 | 0,578325958 | 0,896050827 | 8,42E-05 | 0,002977773 |
| 221012_s_at | NM_030912 | Hs.336810 | tripartite motif-containing 8 | TRIM8 | EG2 | 0 | 2 | 4 | 5 | 0,578325958 | 0,896050827 | 8,42E-05 | 0,002977773 |
| 221139_s_at | NM_015989 | Hs.279815 | cysteine sulfinic acid decarboxylase | CSAD | EG2 | 0 | 2 | 4 | 5 | 0,578325958 | 0,896050827 | 8,42E-05 | 0,002977773 |
| 221489_s_at | W48843 | Hs.323308 | sprouty homolog 4 (Drosophila) | SPRY4 | EG2 | 1 | 1 | 7 | 2 | 0,578325958 | 0,896050827 | 8,42E-05 | 0,002977773 |
| 222020_s_at | AW117456 | Hs.504352 | | HNT | EG2 | 1 | 1 | 6 | 3 | 0,578325958 | 0,896050827 | 8,42E-05 | 0,002977773 |
| 222621_at | BF591419 | Hs.499000 | DnaJ (Hsp40) homolog, subfamily C, member 1 | DNAJC1 | EG2 | 0 | 2 | 9 | 0 | 0,578325958 | 0,896050827 | 8,42E-05 | 0,002977773 |
| 222802_at | J05008 | | | | EG2 | 2 | 0 | 3 | 6 | 0,578325958 | 0,896050827 | 8,42E-05 | 0,002977773 |
| 224839_s_at | BG328998 | Hs.460693 | glutamic pyruvate transaminase (alanine aminotransf | GPT2 | EG2 | 0 | 2 | 6 | 3 | 0,578325958 | 0,896050827 | 8,42E-05 | 0,002977773 |
| 224959_at | AI718385 | Hs.302738 | solute carrier family 26 (sulfate transporter), member | SLC26A2 | EG2 | 2 | 0 | 5 | 4 | 0,578325958 | 0,896050827 | 8,42E-05 | 0,002977773 |
| 225411_at | AI769794 | Hs.469690 | | FLJ14681 | EG2 | 1 | 1 | 3 | 6 | 0,578325958 | 0,896050827 | 8,42E-05 | 0,002977773 |
| 225571_at | AA701657 | Hs.133421 | leukemia inhibitory factor receptor | LIFR | EG2 | 0 | 2 | 2 | 7 | 0,578325958 | 0,896050827 | 8,42E-05 | 0,002977773 |
| 226833_at | AI921877 | Hs.27475 | | FLJ32499 | EG2 | 2 | 0 | 7 | 2 | 0,578325958 | 0,896050827 | 8,42E-05 | 0,002977773 |
| 227037_at | AA029500 | Hs.31652 | | LOC201164 | EG2 | 1 | 1 | 5 | 4 | 0,578325958 | 0,896050827 | 8,42E-05 | 0,002977773 |
| 227070_at | W63754 | Hs.159993 | glycosyltransferase 8 domain containing 2 | GLT8D2 | EG2 | 0 | 2 | 5 | 4 | 0,578325958 | 0,896050827 | 8,42E-05 | 0,002977773 |
| 227347_x_at | NM_021170 | Hs.154029 | hairy and enhancer of split 4 (Drosophila) | HES4 | EG2 | 1 | 1 | 3 | 6 | 0,578325958 | 0,896050827 | 8,42E-05 | 0,002977773 |
| 227657_at | AA722069 | Hs.480825 | ring finger protein 150 | RNF150 | EG2 | 0 | 2 | 1 | 8 | 0,578325958 | 0,896050827 | 8,42E-05 | 0,002977773 |
| 227897_at | N20927 | Hs.98643 | RAP2B, member of RAS oncogene family | RAP2B | EG2 | 1 | 1 | 5 | 4 | 0,578325958 | 0,896050827 | 8,42E-05 | 0,002977773 |
| 228946_at | AW150229 | Hs.391481 | PDZ domain containing 6 | PDZK6 | EG2 | 2 | 0 | 3 | 6 | 0,578325958 | 0,896050827 | 8,42E-05 | 0,002977773 |
| 228998_at | AV693653 | Hs.372082 | trinucleotide repeat containing 6B | TNRC6B | EG2 | 0 | 2 | 2 | 7 | 0,578325958 | 0,896050827 | 8,42E-05 | 0,002977773 |
| 229893_at | BF589413 | Hs.127535 | FERM domain containing 3 | FRMD3 | EG2 | 1 | 1 | 6 | 3 | 0,578325958 | 0,896050827 | 8,42E-05 | 0,002977773 |
| 230425_at | AI674183 | Hs.116092 | EPH receptor B1 | EPHB1 | EG2 | 0 | 2 | 8 | 1 | 0,578325958 | 0,896050827 | 8,42E-05 | 0,002977773 |
| 230559_x_at | AI277617 | Hs.117835 | FYVE, RhoGEF and PH domain containing 4 | FGD4 | EG2 | 0 | 2 | 0 | 9 | 0,578325958 | 0,896050827 | 8,42E-05 | 0,002977773 |
| 230645_at | BF110588 | Hs.127535 | FERM domain containing 3 | FRMD3 | EG2 | 1 | 1 | 6 | 3 | 0,578325958 | 0,896050827 | 8,42E-05 | 0,002977773 |
| 231904_at | AU122448 | Hs.365116 | U2(RNU2) small nuclear RNA auxiliary factor 1 | U2AF1 | EG2 | 1 | 1 | 9 | 0 | 0,578325958 | 0,896050827 | 8,42E-05 | 0,002977773 |
| 235202_x_at | BG498328 | Hs.252543 | | IKIP | EG2 | 2 | 0 | 7 | 2 | 0,578325958 | 0,896050827 | 8,42E-05 | 0,002977773 |
| 235706_at | AW663908 | Hs.334873 | carboxypeptidase M | CPM | EG2 | 1 | 1 | 0 | 9 | 0,578325958 | 0,896050827 | 8,42E-05 | 0,002977773 |
| 238841_at | AA651920 | Hs.437943 | protein tyrosine phosphatase domain containing 1 | PTPDC1 | EG2 | 1 | 1 | 8 | 1 | 0,578325958 | 0,896050827 | 8,42E-05 | 0,002977773 |
| 239761_at | AI088120 | Hs.521568 | glucosaminyl (N-acetyl) transferase 1, core 2 (beta-1,6 | GCNT1 | EG2 | 2 | 0 | 5 | 4 | 0,578325958 | 0,896050827 | 8,42E-05 | 0,002977773 |

| | | | | | | | | | | | | | |
|---|---|---|---|---|---|---|---|---|---|---|---|---|---|
| 242343_x_at | H57111 | Hs.147895 | zinc finger protein 518 | ZNF518 | EG2 | 0 | 2 | 8 | 1 | 0,578325958 | 0,896050827 | 8,42E-05 | 0,002977773 |
| 1555812_a_at | AF498927 | Hs.504877 | Rho GDP dissociation inhibitor (GDI) beta | ARHGDIB | EG2 | 0 | 1 | 3 | 9 | 0,865113269 | 0,896050827 | 7,43E-08 | 0,001562433 |
| 200743_s_at | NM_000391 | Hs.523454 | tripeptidyl peptidase I | TPP1 | EG2 | 0 | 1 | 10 | 2 | 0,865113269 | 0,896050827 | 7,43E-08 | 0,001562433 |
| 227529_s_at | BF511276 | Hs.371240 | A kinase (PRKA) anchor protein (gravin) 12 | AKAP12 | EG2 | 1 | 0 | 5 | 7 | 0,865113269 | 0,896050827 | 7,43E-08 | 0,001562433 |
| 203571_s_at | NM_006829 | Hs.74120 | chromosome 10 open reading frame 116 | C10orf116 | EG2 | 0 | 1 | 5 | 6 | 0,865113269 | 0,896050827 | 9,59E-07 | 0,001562433 |
| 204484_at | NM_002646 | Hs.497487 | phosphoinositide-3-kinase, class 2, beta polypeptide | PIK3C2B | EG2 | 0 | 1 | 4 | 7 | 0,865113269 | 0,896050827 | 9,59E-07 | 0,001562433 |
| 227611_at | AA442856 | Hs.6918 | threonyl-tRNA synthetase-like 2 | TARSL2 | EG2 | 0 | 1 | 5 | 6 | 0,865113269 | 0,896050827 | 9,59E-07 | 0,001562433 |
| 228128_x_at | AI110886 | Hs.494928 | pregnancy-associated plasma protein A, pappalysin 1 | PAPPA | EG2 | 1 | 0 | 8 | 3 | 0,865113269 | 0,896050827 | 9,59E-07 | 0,001562433 |
| 229638_at | AI681917 | Hs.499205 | iroquois homeobox protein 3 | IRX3 | EG2 | 1 | 0 | 4 | 7 | 0,865113269 | 0,896050827 | 9,59E-07 | 0,001562433 |
| 201981_at | AA148534 | Hs.494928 | pregnancy-associated plasma protein A, pappalysin 1 | PAPPA | EG2 | 1 | 0 | 8 | 2 | 0,865113269 | 0,896050827 | 9,97E-06 | 0,001562433 |
| 202036_s_at | AF017987 | Hs.213424 | secreted frizzled-related protein 1 | SFRP1 | EG2 | 0 | 1 | 7 | 3 | 0,865113269 | 0,896050827 | 9,97E-06 | 0,001562433 |
| 206172_at | NM_000640 | Hs.336046 | interleukin 13 receptor, alpha 2 | IL13RA2 | EG2 | 1 | 0 | 7 | 3 | 0,865113269 | 0,896050827 | 9,97E-06 | 0,001562433 |
| 206371_at | NM_000804 | Hs.352 | folate receptor 3 (gamma) | FOLR3 | EG2 | 0 | 1 | 7 | 3 | 0,865113269 | 0,896050827 | 9,97E-06 | 0,001562433 |
| 209460_at | AF237813 | Hs.336768 | 4-aminobutyrate aminotransferase | ABAT | EG2 | 1 | 0 | 6 | 4 | 0,865113269 | 0,896050827 | 9,97E-06 | 0,001562433 |
| 210517_s_at | AB003476 | Hs.371240 | A kinase (PRKA) anchor protein (gravin) 12 | AKAP12 | EG2 | 1 | 0 | 4 | 6 | 0,865113269 | 0,896050827 | 9,97E-06 | 0,001562433 |
| 217564_s_at | W80357 | Hs.149252 | carbamoyl-phosphate synthetase 1, mitochondrial | CPS1 | EG2 | 1 | 0 | 5 | 5 | 0,865113269 | 0,896050827 | 9,97E-06 | 0,001562433 |
| 219441_s_at | NM_024652 | Hs.513207 | leucine-rich repeat kinase 1 | LRRK1 | EG2 | 0 | 1 | 0 | 10 | 0,865113269 | 0,896050827 | 9,97E-06 | 0,001562433 |
| 222150_s_at | AK026747 | Hs.186649, Hs.549342 | | LOC54103 | EG2 | 1 | 0 | 5 | 5 | 0,865113269 | 0,896050827 | 9,97E-06 | 0,001562433 |
| 224940_s_at | BF107618 | Hs.494928 | pregnancy-associated plasma protein A, pappalysin 1 | PAPPA | EG2 | 1 | 0 | 7 | 3 | 0,865113269 | 0,896050827 | 9,97E-06 | 0,001562433 |
| 224941_at | BF107618 | Hs.494928 | pregnancy-associated plasma protein A, pappalysin 1 | PAPPA | EG2 | 1 | 0 | 7 | 3 | 0,865113269 | 0,896050827 | 9,97E-06 | 0,001562433 |
| 227997_at | AW007080 | Hs.150725 | interleukin 17 receptor D | IL17RD | EG2 | 0 | 1 | 5 | 5 | 0,865113269 | 0,896050827 | 9,97E-06 | 0,001562433 |
| 229441_at | AI569872 | Hs.19545 | frizzled homolog 4 (Drosophila) | FZD4 | EG2 | 1 | 0 | 9 | 1 | 0,865113269 | 0,896050827 | 9,97E-06 | 0,001562433 |
| 230831_at | AW294986 | Hs.368399 | FERM domain containing 5 | FRMD5 | EG2 | 1 | 0 | 7 | 3 | 0,865113269 | 0,896050827 | 9,97E-06 | 0,001562433 |
| 235299_at | AI769269 | | | | EG2 | 0 | 1 | 7 | 3 | 0,865113269 | 0,896050827 | 9,97E-06 | 0,001562433 |
| 239629_at | AI634046 | Hs.390736 | CASP8 and FADD-like apoptosis regulator | CFLAR | EG2 | 1 | 0 | 7 | 3 | 0,865113269 | 0,896050827 | 9,97E-06 | 0,001562433 |
| 1552812_a_at | NM_014554 | Hs.371957 | SUMO1/sentrin specific peptidase 1 | SENP1 | EG2 | 1 | 0 | 5 | 4 | 0,865113269 | 0,896050827 | 8,42E-05 | 0,002977773 |
| 1557275_a_at | BF726849 | | | | EG2 | 0 | 1 | 0 | 9 | 0,865113269 | 0,896050827 | 8,42E-05 | 0,002977773 |
| 200706_s_at | NM_004862 | Hs.459940 | lipopolysaccharide-induced TNF factor | LITAF | EG2 | 0 | 1 | 5 | 4 | 0,865113269 | 0,896050827 | 8,42E-05 | 0,002977773 |
| 201645_at | NM_002160 | Hs.143250 | tenascin C (hexabrachion) | TNC | EG2 | 1 | 0 | 8 | 1 | 0,865113269 | 0,896050827 | 8,42E-05 | 0,002977773 |
| 201801_s_at | AF079117 | Hs.25450 | solute carrier family 29 (nucleoside transporters), me | SLC29A1 | EG2 | 0 | 1 | 8 | 1 | 0,865113269 | 0,896050827 | 8,42E-05 | 0,002977773 |
| 202741_at | AA130247 | Hs.487325 | protein kinase, cAMP-dependent, catalytic, beta | PRKACB | EG2 | 1 | 0 | 4 | 5 | 0,865113269 | 0,896050827 | 8,42E-05 | 0,002977773 |
| 205542_at | NM_012449 | Hs.61635 | six transmembrane epithelial antigen of the prostate | STEAP1 | EG2 | 0 | 1 | 7 | 2 | 0,865113269 | 0,896050827 | 8,42E-05 | 0,002977773 |
| 205920_at | NM_003043 | Hs.529488 | solute carrier family 6 (neurotransmitter transporter, t | SLC6A6 | EG2 | 1 | 0 | 7 | 2 | 0,865113269 | 0,896050827 | 8,42E-05 | 0,002977773 |
| 206942_s_at | NM_002674 | Hs.2182 | pro-melanin-concentrating hormone | PMCH | EG2 | 0 | 1 | 2 | 7 | 0,865113269 | 0,896050827 | 8,42E-05 | 0,002977773 |
| 209505_at | AI951185 | Hs.519445 | nuclear receptor subfamily 2, group F, member 1 | NR2F1 | EG2 | 1 | 0 | 3 | 6 | 0,865113269 | 0,896050827 | 8,42E-05 | 0,002977773 |
| 211527_x_at | M27281 | Hs.73793 | vascular endothelial growth factor | VEGF | EG2 | 0 | 1 | 8 | 1 | 0,865113269 | 0,896050827 | 8,42E-05 | 0,002977773 |
| 212382_at | BF433429 | Hs.200285 | transcription factor 4 | TCF4 | EG2 | 0 | 1 | 4 | 5 | 0,865113269 | 0,896050827 | 8,42E-05 | 0,002977773 |
| 212521_s_at | BE568219 | Hs.9333 | phosphodiesterase 8A | PDE8A | EG2 | 0 | 1 | 3 | 6 | 0,865113269 | 0,896050827 | 8,42E-05 | 0,002977773 |
| 213295_at | AA555096 | Hs.432993 | cylindromatosis (turban tumor syndrome) | CYLD | EG2 | 1 | 0 | 4 | 5 | 0,865113269 | 0,896050827 | 8,42E-05 | 0,002977773 |
| 214023_x_at | AL533838 | Hs.300701 | | TUBB-PARALOG | EG2 | 0 | 1 | 1 | 8 | 0,865113269 | 0,896050827 | 8,42E-05 | 0,002977773 |
| 222668_at | W73820 | Hs.221873 | potassium channel tetramerisation domain containing | KCTD15 | EG2 | 0 | 1 | 6 | 3 | 0,865113269 | 0,896050827 | 8,42E-05 | 0,002977773 |
| 222717_at | BF982174 | Hs.26530 | serum deprivation response (phosphatidylserine bind | SDPR | EG2 | 1 | 0 | 5 | 4 | 0,865113269 | 0,896050827 | 8,42E-05 | 0,002977773 |
| 224002_s_at | AF100751 | Hs.410378 | FK506 binding protein 7 | FKBP7 | EG2 | 0 | 1 | 7 | 2 | 0,865113269 | 0,896050827 | 8,42E-05 | 0,002977773 |
| 224719_s_at | BG339653 | Hs.405913 | | GRCC10 | EG2 | 0 | 1 | 5 | 4 | 0,865113269 | 0,896050827 | 8,42E-05 | 0,002977773 |
| 224817_at | W93554 | Hs.549085 | neuralized-like (Drosophila) | NEURL | EG2 | 1 | 0 | 4 | 5 | 0,865113269 | 0,896050827 | 8,42E-05 | 0,002977773 |

| | | | | | | | | | | | | | |
|---|---|---|---|---|---|---|---|---|---|---|---|---|---|
| 224837_at | AW080845 | Hs.431498 | forkhead box P1 | FOXP1 | EG2 | 0 | 1 | 4 | 5 | 0,865113269 | 0,896050827 | 8,42E-05 | 0,002977773 |
| 225653_at | AV755269 | Hs.446350 | transforming growth factor, beta receptor associated | TGFBRAP1 | EG2 | 0 | 1 | 5 | 4 | 0,865113269 | 0,896050827 | 8,42E-05 | 0,002977773 |
| 226719_at | AI868039 | | | | EG2 | 0 | 1 | 9 | 0 | 0,865113269 | 0,896050827 | 8,42E-05 | 0,002977773 |
| 226771_at | AB032963 | Hs.435700 | ATPase, Class I, type 8B, member 2 | ATP8B2 | EG2 | 0 | 1 | 4 | 5 | 0,865113269 | 0,896050827 | 8,42E-05 | 0,002977773 |
| 229830_at | AW205919 | Hs.376032, H | platelet-derived growth factor alpha polypeptide | PDGFA | EG2 | 0 | 1 | 2 | 7 | 0,865113269 | 0,896050827 | 8,42E-05 | 0,002977773 |
| 230748_at | AI873273 | Hs.42645 | solute carrier family 16 (monocarboxylic acid transpor | SLC16A6 | EG2 | 0 | 1 | 7 | 2 | 0,865113269 | 0,896050827 | 8,42E-05 | 0,002977773 |
| 231403_at | N21108 | Hs.130031 | triple functional domain (PTPRF interacting) | TRIO | EG2 | 0 | 1 | 6 | 3 | 0,865113269 | 0,896050827 | 8,42E-05 | 0,002977773 |
| 235508_at | AW291023 | Hs.526464 | promyelocytic leukemia | PML | EG2 | 1 | 0 | 8 | 1 | 0,865113269 | 0,896050827 | 8,42E-05 | 0,002977773 |
| 237169_at | AI266750 | Hs.143250 | tenascin C (hexabrachion) | TNC | EG2 | 1 | 0 | 9 | 0 | 0,865113269 | 0,896050827 | 8,42E-05 | 0,002977773 |
| 242979_at | AI474666 | | | | EG2 | 1 | 0 | 3 | 6 | 0,865113269 | 0,896050827 | 8,42E-05 | 0,002977773 |
| 217853_at | NM_022748 | Hs.520814 | tensin 3 | TNS3 | EG2 | 0 | 0 | 5 | 7 | 1 | 0,896050827 | 7,43E-08 | 0,001562433 |
| 204203_at | NM_001806 | Hs.429666 | CCAAT/enhancer binding protein (C/EBP), gamma | CEBPG | EG2 | 0 | 0 | 8 | 3 | 1 | 0,896050827 | 9,59E-07 | 0,001562433 |
| 209199_s_at | N22468 | Hs.444409 | MADS box transcription enhancer factor 2, polypeptid | MEF2C | EG2 | 0 | 0 | 8 | 3 | 1 | 0,896050827 | 9,59E-07 | 0,001562433 |
| 210041_s_at | BC001258 | Hs.553496 | phosphoglucomutase 3 | PGM3 | EG2 | 0 | 0 | 6 | 4 | 1 | 0,896050827 | 9,97E-06 | 0,001562433 |
| 213664_at | AW235061 | Hs.444915 | solute carrier family 1 (neuronal/epithelial high affini | SLC1A1 | EG2 | 0 | 0 | 7 | 3 | 1 | 0,896050827 | 9,97E-06 | 0,001562433 |
| 213895_at | BF445047 | Hs.436298 | epithelial membrane protein 1 | EMP1 | EG2 | 0 | 0 | 6 | 4 | 1 | 0,896050827 | 9,97E-06 | 0,001562433 |
| 224838_at | AK026898 | Hs.431498 | forkhead box P1 | FOXP1 | EG2 | 0 | 0 | 4 | 6 | 1 | 0,896050827 | 9,97E-06 | 0,001562433 |
| 225362_at | AI341165 | Hs.404706 | | LOC159090 | EG2 | 0 | 0 | 5 | 5 | 1 | 0,896050827 | 9,97E-06 | 0,001562433 |
| 228335_at | AW264204 | Hs.31595 | claudin 11 (oligodendrocyte transmembrane protein) | CLDN11 | EG2 | 0 | 0 | 3 | 7 | 1 | 0,896050827 | 9,97E-06 | 0,001562433 |
| 39582_at | AL050166 | Hs.432993 | cylindromatosis (turban tumor syndrome) | CYLD | EG2 | 0 | 0 | 5 | 5 | 1 | 0,896050827 | 9,97E-06 | 0,001562433 |
| 1554795_a_at | BC019895 | Hs.530101 | filamin binding LIM protein 1 | FBLIM1 | EG2 | 0 | 0 | 1 | 8 | 1 | 0,896050827 | 8,42E-05 | 0,002977773 |
| 207265_s_at | NM_016657 | Hs.554798 | KDEL (Lys-Asp-Glu-Leu) endoplasmic reticulum protei | KDELR3 | EG2 | 0 | 0 | 6 | 3 | 1 | 0,896050827 | 8,42E-05 | 0,002977773 |
| 210026_s_at | AY028896 | Hs.57973 | caspase recruitment domain family, member 10 | CARD10 | EG2 | 0 | 0 | 1 | 8 | 1 | 0,896050827 | 8,42E-05 | 0,002977773 |
| 210138_at | AF074979 | Hs.368733 | regulator of G-protein signalling 20 | RGS20 | EG2 | 0 | 0 | 7 | 2 | 1 | 0,896050827 | 8,42E-05 | 0,002977773 |
| 212816_s_at | BE613178 | Hs.533013 | cystathionine-beta-synthase | CBS | EG2 | 0 | 0 | 4 | 5 | 1 | 0,896050827 | 8,42E-05 | 0,002977773 |
| 213358_at | AB018345 | Hs.464563 | KIAA0802 | KIAA0802 | EG2 | 0 | 0 | 3 | 6 | 1 | 0,896050827 | 8,42E-05 | 0,002977773 |
| 220161_s_at | NM_019114 | Hs.269180 | erythrocyte membrane protein band 4.1 like 4B | EPB41L4B | EG2 | 0 | 0 | 1 | 8 | 1 | 0,896050827 | 8,42E-05 | 0,002977773 |
| 224204_x_at | AF231339 | Hs.434269 | aryl hydrocarbon receptor nuclear translocator-like 2 | ARNTL2 | EG2 | 0 | 0 | 2 | 7 | 1 | 0,896050827 | 8,42E-05 | 0,002977773 |
| 230121_at | BF508679 | | | | EG2 | 0 | 0 | 2 | 7 | 1 | 0,896050827 | 8,42E-05 | 0,002977773 |
| 237466_s_at | AW444502 | Hs.507991 | hedgehog interacting protein | HHIP | EG2 | 0 | 0 | 0 | 9 | 1 | 0,896050827 | 8,42E-05 | 0,002977773 |
| 241387_at | AW276701 | Hs.395482 | PTK2 protein tyrosine kinase 2 | PTK2 | EG2 | 0 | 0 | 3 | 6 | 1 | 0,896050827 | 8,42E-05 | 0,002977773 |
