# Supplementary material for: Identification of evolutionarily conserved genetic regulators of cellular aging
Source: Aging Cell. 2010 Dec;9(6):1084–97. doi: 10.1111/j.1474-9726.2010.00637.x (PMC2997327; doi:10.1111/j.1474-9726.2010.00637.x)
Supplement: Supplementary file 6 [file acel0009-1084-SD6.pdf]

| Symbol | Description | Unigene | GenBank | EG1 up | EG1 down | EG2 up | EG2 down | EG1 + EG2 DE |
|---|---|---|---|---|---|---|---|---|
| KIAA0101 | KIAA0101/PAF | Hs.81892 | NM_014736 | 7 | 7 | 6 | 7 | 27 |
| APOBEC3G | apolipoprotein B mRNA editing enzyme, catalytic polypeptide-like 3G | Hs.660143 | NM_021822 | 0 | 10 | 8 | 8 | 26 |
| MEST | mesoderm specific transcript homolog (mouse) | Hs.270978 | NM_002402 | 4 | 5 | 8 | 8 | 25 |
| RRM2 | ribonucleotide reductase M2 polypeptide | Hs.226390 | BE966236 | 7 | 5 | 4 | 9 | 25 |
| SYDE2 | synapse defective 1, Rho GTPase, homolog 2 (C. elegans) | Hs.718601 | N90719 | 7 | 5 | 5 | 8 | 25 |
| TXNIP | thioredoxin interacting protein | Hs.715525 | AA812232 | 2 | 9 | 7 | 7 | 25 |
| BHLHB2 | basic helix-loop-helix domain containing, class B, 2 | Hs.719093 | NM_003670 | 5 | 6 | 10 | 3 | 24 |
| BICD1 | bicaudal D homolog 1 (Drosophila) | Hs.505202 | BC010091 | 10 | 3 | 7 | 4 | 24 |
| CYP51A1 | cytochrome P450, family 51, subfamily A, polypeptide 1 | Hs.417077 | U40053 | 10 | 1 | 10 | 3 | 24 |
| HMGCS1 | 3-hydroxy-3-methylglutaryl-Coenzyme A synthase 1 (soluble) | Hs.397729 | NM_002130 | 9 | 3 | 9 | 3 | 24 |
| SKP2 | S-phase kinase-associated protein 2 (p45) | Hs.23348 | BC001441 | 5 | 9 | 3 | 7 | 24 |
| SLC1A4 | solute carrier family 1 (glutamate/neutral amino acid transporter), me | Hs.654352 | BF340083 | 5 | 1 | 11 | 7 | 24 |
| TCF8 | transcription factor 8 (represses interleukin 2 expression) | Hs.282113 | NM_030751 | 6 | 6 | 6 | 6 | 24 |
| C20orf129 | chromosome 20 open reading frame 129 | Hs.472716 | BC001068 | 11 | 1 | 7 | 4 | 23 |
| CD302 | CD302 antigen | Hs.130014 | NM_014880 | 7 | 4 | 6 | 6 | 23 |
| IFI44L | interferon-induced protein 44-like | Hs.389724 | NM_006820 | 3 | 7 | 8 | 5 | 23 |
| PDGFD | platelet derived growth factor D | Hs.352298 | NM_025208 | 5 | 4 | 6 | 8 | 23 |
| RUNX1 | runt-related transcription factor 1 (acute myeloid leukemia 1, aml1 onc | Hs.675708 | BU789637 | 2 | 9 | 9 | 3 | 23 |
| TCEA3 | transcription elongation factor A (SII), 3 | Hs.446354 | AI675780 | 2 | 8 | 4 | 9 | 23 |
| TSPAN2 | tetraspanin 2 | Hs.310458 | AI743596 | 4 | 6 | 7 | 6 | 23 |
| ULBP2 | UL16 binding protein 2 | Hs.656778 | AA831769 | 10 | 2 | 7 | 4 | 23 |

| | | | | | | | | |
|---|---|---|---|---|---|---|---|---|
| VIM | vimentin | Hs.628678 | AI520969 | 8 | 3 | 10 | 2 | 23 |
| ARRB1 | arrestin, beta 1 | Hs.503284 | BE207758 | 0 | 5 | 8 | 9 | 22 |
| BCL2L11 | BCL2-like 11 (apoptosis facilitator) | Hs.469658 | AI949179 | 5 | 4 | 7 | 6 | 22 |
| BM039 | | Hs.55028 | AK023669 | 10 | 2 | 3 | 7 | 22 |
| CASP1 | caspase 1, apoptosis-related cysteine peptidase (interleukin 1, beta, co | Hs.2490 | U13699 | 2 | 10 | 7 | 3 | 22 |
| CCNB2 | cyclin B2 | Hs.194698 | NM_004701 | 5 | 7 | 3 | 7 | 22 |
| CDKN1C | cyclin-dependent kinase inhibitor 1C (p57, Kip2) | Hs.106070 | N33167 | 5 | 6 | 5 | 6 | 22 |
| CEBPD | CCAAT/enhancer binding protein (C/EBP), delta | Hs.440829 | NM_005195 | 1 | 9 | 3 | 9 | 22 |
| CLIC3 | chloride intracellular channel 3 | Hs.64746 | NM_004669 | 0 | 9 | 7 | 6 | 22 |
| CTSC | cathepsin C | Hs.128065 | AI246687 | 2 | 5 | 10 | 5 | 22 |
| DLEU2 | deleted in lymphocytic leukemia, 2 | Hs.547964 | H48516 | 7 | 4 | 5 | 6 | 22 |
| FAM54A | family with sequence similarity 54, member A | Hs.121536 | AL138828 | 9 | 2 | 2 | 9 | 22 |
| GLS | glutaminase | Hs.116448 | AI828035 | 10 | 1 | 7 | 4 | 22 |
| IGFBP3 | insulin-like growth factor binding protein 3 | Hs.450230 | BF340228 | 4 | 8 | 7 | 3 | 22 |
| LOC387763 | | Hs.714890 | AW276078 | 7 | 3 | 9 | 3 | 22 |
| MALAT1 | metastasis associated lung adenocarcinoma transcript 1 (non-coding RN | --- | AF001540 | 6 | 4 | 5 | 7 | 22 |
| MGC39900 | | Hs.376474 | H09657 | 6 | 4 | 2 | 10 | 22 |
| PARP9 | poly (ADP-ribose) polymerase family, member 9 | Hs.518200 | AF307338 | 1 | 8 | 4 | 9 | 22 |
| Pfs2 | | Hs.433180 | BC003186 | 4 | 6 | 2 | 10 | 22 |
| PRC1 | protein regulator of cytokinesis 1 | Hs.567385 | NM_003981 | 10 | 1 | 5 | 6 | 22 |
| PTGS2 | prostaglandin-endoperoxide synthase 2 (prostaglandin G/H synthase a | Hs.196384 | NM_000963 | 1 | 7 | 8 | 6 | 22 |
| RAB30 | RAB30, member RAS oncogene family | Hs.40758 | AW294640 | 10 | 0 | 5 | 7 | 22 |
| SLC20A1 | solute carrier family 20 (phosphate transporter), member 1 | Hs.187946 | AI671885 | 7 | 4 | 8 | 3 | 22 |
| SLC7A5 | solute carrier family 7 (cationic amino acid transporter, y+ system), me | Hs.513797 | AB018009 | 7 | 2 | 9 | 4 | 22 |
| TncRNA | | Hs.648467 | AU134977 | 8 | 3 | 6 | 5 | 22 |

| | | | | | | | | |
|---|---|---|---|---|---|---|---|---|
| ZWINT | ZW10 interactor | Hs.591363 | NM_007057 | 5 | 5 | 4 | 8 | 22 |
| ADAM19 | ADAM metallopeptidase domain 19 (meltrin beta) | Hs.483944 | Y13786 | 2 | 7 | 3 | 9 | 21 |
| C3orf6 | chromosome 3 open reading frame 6 | Hs.478682 | AI247881 | 5 | 6 | 9 | 1 | 21 |
| CCNB1 | cyclin B1 | Hs.23960 | BE407516 | 5 | 5 | 4 | 7 | 21 |
| CTH | cystathionase (cystathionine gamma-lyase) | Hs.19904 | NM_001902 | 7 | 2 | 8 | 4 | 21 |
| DHCR7 | 7-dehydrocholesterol reductase | Hs.503134 | AW150953 | 7 | 4 | 7 | 3 | 21 |
| DKFZP586A0522 | | Hs.716437 | NM_014033 | 3 | 3 | 10 | 5 | 21 |
| EGR1 | early growth response 1 | Hs.326035 | NM_001964 | 6 | 6 | 7 | 2 | 21 |
| EIF2S3 | eukaryotic translation initiation factor 2, subunit 3 gamma, 52kDa | Hs.539684 | NM_001415 | 10 | 1 | 3 | 7 | 21 |
| EIF4A2 | eukaryotic translation initiation factor 4A, isoform 2 | Hs.518475 | AI332397 | 10 | 3 | 6 | 2 | 21 |
| ENC1 | ectodermal-neural cortex (with BTB-like domain) | Hs.104925 | AF010314 | 6 | 2 | 9 | 4 | 21 |
| EPSTI1 | epithelial stromal interaction 1 (breast) | Hs.546467 | AA633203 | 2 | 8 | 2 | 9 | 21 |
| FBXO9 | F-box protein 9 | Hs.216653 | AK095307 | 8 | 4 | 8 | 1 | 21 |
| FHL1 | four and a half LIM domains 1 | Hs.435369 | AF063002 | 3 | 5 | 1 | 12 | 21 |
| GLIPR1 | GLI pathogenesis-related 1 (glioma) | Hs.205558 | AV682252 | 5 | 1 | 7 | 8 | 21 |
| HDAC9 | histone deacetylase 9 | Hs.196054 | NM_014707 | 4 | 6 | 7 | 4 | 21 |
| IFIT3 | interferon-induced protein with tetratricopeptide repeats 3 | Hs.714337 | AI075407 | 2 | 5 | 7 | 7 | 21 |
| IRAK1BP1 | interleukin-1 receptor-associated kinase 1 binding protein 1 | Hs.656212 | AI168124 | 3 | 5 | 3 | 10 | 21 |
| KLF4 | Kruppel-like factor 4 (gut) | Hs.376206 | BF514079 | 3 | 6 | 7 | 5 | 21 |
| KRT18 | keratin 18 | Hs.406013 | NM_000224 | 2 | 7 | 4 | 8 | 21 |
| LOC284611 | | Hs.200230 | AI478747 | 9 | 2 | 5 | 5 | 21 |
| MAD2L1 | MAD2 mitotic arrest deficient-like 1 (yeast) | Hs.591697 | NM_002358 | 7 | 4 | 3 | 7 | 21 |
| NUSAP1 | nucleolar and spindle associated protein 1 | Hs.615092 | NM_016359 | 6 | 5 | 3 | 7 | 21 |
| PGAP1 | | Hs.229988 | NM_024989 | 6 | 1 | 8 | 6 | 21 |
| PHGDH | phosphoglycerate dehydrogenase | Hs.487296 | NM_006623 | 2 | 5 | 4 | 10 | 21 |
| PHLDA1 | pleckstrin homology-like domain, family A, member 1 | Hs.602085 | AK026181 | 6 | 1 | 10 | 4 | 21 |

| | | | | | | | | |
|---|---|---|---|---|---|---|---|---|
| PKIA | protein kinase (cAMP-dependent, catalytic) inhibitor alpha | Hs.433700 | NM_006823 | 4 | 4 | 8 | 5 | 21 |
| PLEKHA2 | pleckstrin homology domain containing, family A (phosphoinositide bi | Hs.369123 | BF347859 | 4 | 6 | 5 | 6 | 21 |
| PRR6 | proline rich 6 | Hs.433422 | AA722878 | 7 | 1 | 5 | 8 | 21 |
| PSF1 | | Hs.658464 | NM_021067 | 5 | 7 | 3 | 6 | 21 |
| RAB27B | RAB27B, member RAS oncogene family | Hs.25318 | BF438386 | 3 | 3 | 11 | 4 | 21 |
| RAB8B | RAB8B, member RAS oncogene family | Hs.389733 | AB038995 | 10 | 2 | 6 | 3 | 21 |
| SKIL | SKI-like | Hs.581632 | BF725121 | 9 | 2 | 7 | 3 | 21 |
| SLC40A1 | solute carrier family 40 (iron-regulated transporter), member 1 | Hs.643005 | AL136944 | 2 | 7 | 3 | 9 | 21 |
| SMC4L1 | SMC4 structural maintenance of chromosomes 4-like 1 (yeast) | Hs.58992 | NM_005496 | 10 | 1 | 5 | 5 | 21 |
| STK6 | serine/threonine kinase 6 | Hs.250822 | NM_003158 | 9 | 2 | 3 | 7 | 21 |
| TBL1X | transducin (beta)-like 1X-linked | Hs.495656 | AW968555 | 2 | 6 | 6 | 7 | 21 |
| TOX | | Hs.491805 | AI961231 | 6 | 2 | 4 | 9 | 21 |
| TPD52 | tumor protein D52 | Hs.368433 | BG389015 | 1 | 8 | 9 | 3 | 21 |
| TUFT1 | tuftelin 1 | Hs.489922 | NM_020127 | 6 | 3 | 4 | 8 | 21 |
| ZFHX1B | zinc finger homeobox 1b | Hs.34871 | AL546529 | 8 | 1 | 7 | 5 | 21 |
| ANK3 | ankyrin 3, node of Ranvier (ankyrin G) | Hs.499725 | NM_020987 | 8 | 1 | 3 | 8 | 20 |
| ASB3 | ankyrin repeat and SOCS box-containing 3 | Hs.585944 | AI191897 | 9 | 2 | 7 | 2 | 20 |
| ATAD2 | ATPase family, AAA domain containing 2 | Hs.370834 | NM_014109 | 8 | 1 | 4 | 7 | 20 |
| ATM | ataxia telangiectasia mutated (includes complementation groups A, C a | Hs.367437 | NM_000051 | 7 | 1 | 7 | 5 | 20 |
| BCL3 | B-cell CLL/lymphoma 3 | Hs.31210 | NM_005178 | 7 | 2 | 4 | 7 | 20 |
| BIRC5 | baculoviral IAP repeat-containing 5 (survivin) | Hs.514527 | NM_001168 | 6 | 5 | 3 | 6 | 20 |
| CKAP2 | cytoskeleton associated protein 2 | Hs.444028 | NM_018204 | 11 | 1 | 4 | 4 | 20 |
| CTSK | cathepsin K (pycnodysostosis) | Hs.632466 | NM_000396 | 0 | 10 | 9 | 1 | 20 |
| CTTNBP2NL | CTTNBP2 N-terminal like | Hs.485899 | AB037854 | 5 | 3 | 11 | 1 | 20 |
| DAAM1 | dishevelled associated activator of morphogenesis 1 | Hs.19156 | AA890373 | 8 | 4 | 5 | 3 | 20 |

| | | | | | | | | |
|---|---|---|---|---|---|---|---|---|
| ENO2 | enolase 2 (gamma, neuronal) | Hs.511915 | NM_001975 | 2 | 6 | 5 | 7 | 20 |
| EPHA4 | EPH receptor A4 | Hs.371218 | AI799018 | 4 | 4 | 4 | 8 | 20 |
| FLJ44635 | | Hs.496303 | AI250910 | 4 | 5 | 8 | 3 | 20 |
| FOS | v-fos FBJ murine osteosarcoma viral oncogene homolog | Hs.707896 | BC004490 | 1 | 6 | 6 | 7 | 20 |
| GAS1 | growth arrest-specific 1 | Hs.65029 | NM_002048 | 3 | 5 | 6 | 6 | 20 |
| HK2 | hexokinase 2 | Hs.406266 | AI761561 | 6 | 1 | 7 | 6 | 20 |
| HSUP1 | | Hs.356766 | AI625235 | 8 | 0 | 4 | 8 | 20 |
| ID3 | inhibitor of DNA binding 3, dominant negative helix-loop-helix protein | Hs.76884 | NM_002167 | 6 | 2 | 6 | 6 | 20 |
| ITGA6 | integrin, alpha 6 | Hs.133397 | AV733308 | 6 | 1 | 6 | 7 | 20 |
| KIF2C | kinesin family member 2C | Hs.69360 | U63743 | 5 | 6 | 3 | 6 | 20 |
| KLF11 | Kruppel-like factor 11 | Hs.12229 | AA149594 | 7 | 1 | 7 | 5 | 20 |
| KLF7 | Kruppel-like factor 7 (ubiquitous) | Hs.471221 | AA488672 | 3 | 6 | 3 | 8 | 20 |
| LASS6 | LAG1 longevity assurance homolog 6 (S. cerevisiae) | Hs.718403 | AI658534 | 6 | 3 | 5 | 6 | 20 |
| LBH | | Hs.567598 | NM_030915 | 3 | 4 | 5 | 8 | 20 |
| LRP8 | low density lipoprotein receptor-related protein 8, apolipoprotein e re | Hs.719248 | NM_004631 | 7 | 2 | 5 | 6 | 20 |
| MCC | mutated in colorectal cancers | Hs.593171 | BE967311 | 3 | 3 | 3 | 11 | 20 |
| MELK | maternal embryonic leucine zipper kinase | Hs.184339 | NM_014791 | 5 | 4 | 3 | 8 | 20 |
| MGP | matrix Gla protein | Hs.365706 | NM_000900 | 2 | 6 | 4 | 8 | 20 |
| MX1 | myxovirus (influenza virus) resistance 1, interferon-inducible protein p | Hs.517307 | NM_002462 | 1 | 7 | 2 | 10 | 20 |
| NT5E | 5'-nucleotidase, ecto (CD73) | Hs.153952 | BC015940 | 5 | 5 | 5 | 5 | 20 |
| OGT | O-linked N-acetylglucosamine (GlcNAc) transferase (UDP-N-acetylgluc | Hs.405410 | U77413 | 7 | 2 | 8 | 3 | 20 |
| PRO1073 | | Hs.642877 | AF113016 | 5 | 3 | 7 | 5 | 20 |
| PSAT1 | phosphoserine aminotransferase 1 | Hs.494261 | BC004863 | 7 | 2 | 5 | 6 | 20 |
| PSMB9 | proteasome (prosome, macropain) subunit, beta type, 9 (large multifu | Hs.654585 | NM_002800 | 3 | 5 | 4 | 8 | 20 |

| | | | | | | | | |
|---|---|---|---|---|---|---|---|---|
| RGC32 | | Hs.507866 | NM_014059 | 8 | 3 | 1 | 8 | 20 |
| SETBP1 | SET binding protein 1 | Hs.435458 | BF739885 | 3 | 6 | 6 | 5 | 20 |
| SFRS6 | splicing factor, arginine/serine-rich 6 | Hs.6891 | NM_006275 | 6 | 3 | 1 | 10 | 20 |
| SLC2A1 | solute carrier family 2 (facilitated glucose transporter), member 1 | Hs.706748 | NM_006516 | 6 | 4 | 7 | 3 | 20 |
| TBRG4 | transforming growth factor beta regulator 4 | Hs.231411 | NM_004749 | 2 | 9 | 3 | 6 | 20 |
| TOP2A | topoisomerase (DNA) II alpha 170kDa | Hs.156346 | AU159942 | 6 | 4 | 3 | 7 | 20 |
| TRIB3 | tribbles homolog 3 (Drosophila) | Hs.516826 | NM_021158 | 0 | 5 | 10 | 5 | 20 |
| TRIM59 | tripartite motif-containing 59 | Hs.212957 | AW182459 | 3 | 6 | 4 | 7 | 20 |
| TRPV2 | transient receptor potential cation channel, subfamily V, member 2 | Hs.279746 | NM_015930 | 4 | 10 | 5 | 1 | 20 |
| UBE2T | ubiquitin-conjugating enzyme E2T (putative) | Hs.5199 | AB032931 | 5 | 6 | 2 | 7 | 20 |
| ZNF207 | zinc finger protein 207 | Hs.500775 | AI201248 | 7 | 2 | 8 | 3 | 20 |
| ZNF431 | zinc finger protein 431 | Hs.687547 | AU144815 | 8 | 3 | 5 | 4 | 20 |
| ZNF688 | zinc finger protein 688 | Hs.301463 | AI095896 | 2 | 8 | 6 | 4 | 20 |
| AK5 | adenylate kinase 5 | Hs.559718 | BG169832 | 4 | 2 | 6 | 7 | 19 |
| ARL8 | ADP-ribosylation factor-like 8 | Hs.25362 | BG032269 | 9 | 1 | 4 | 5 | 19 |
| ATP5S | ATP synthase, H+ transporting, mitochondrial F0 complex, subunit s (fac | Hs.656689 | AI308101 | 4 | 6 | 5 | 4 | 19 |
| C1RL | complement component 1, r subcomponent-like | Hs.631730 | NM_016546 | 1 | 8 | 2 | 8 | 19 |
| C5orf13 | chromosome 5 open reading frame 13 | Hs.36053 | NM_004772 | 4 | 7 | 5 | 3 | 19 |
| CD44 | CD44 antigen (homing function and Indian blood group system) | Hs.502328 | BE467023 | 7 | 2 | 5 | 5 | 19 |
| ChGn | | Hs.655166 | NM_018371 | 2 | 9 | 2 | 6 | 19 |
| CTSB | cathepsin B | Hs.520898 | AA130998 | 3 | 7 | 4 | 5 | 19 |
| DAPK1 | death-associated protein kinase 1 | Hs.380277 | NM_004938 | 2 | 5 | 7 | 5 | 19 |
| DKFZP564F0522 | | Hs.468140 | AI743261 | 4 | 5 | 7 | 3 | 19 |
| DNAJC7 | DnaJ (Hsp40) homolog, subfamily C, member 7 | Hs.663572 | AW814026 | 7 | 2 | 6 | 4 | 19 |
| DSCR1L1 | Down syndrome critical region gene 1-like 1 | Hs.440168 | NM_005822 | 5 | 2 | 5 | 7 | 19 |

| | | | | | | | | |
|---|---|---|---|---|---|---|---|---|
| DUSP6 | dual specificity phosphatase 6 | Hs.298654 | BC003143 | 6 | 3 | 8 | 2 | 19 |
| E2F7 | E2F transcription factor 7 | Hs.416375 | AI341146 | 3 | 3 | 11 | 2 | 19 |
| ELOVL6 | ELOVL family member 6, elongation of long chain fatty acids (FEN1/Elo2 | Hs.412939 | NM_024090 | 5 | 1 | 7 | 6 | 19 |
| ENOSF1 | enolase superfamily member 1 | Hs.369762 | AF305057 | 8 | 2 | 6 | 3 | 19 |
| EZH2 | enhancer of zeste homolog 2 (Drosophila) | Hs.444082 | NM_004456 | 5 | 2 | 4 | 8 | 19 |
| FAS | Fas (TNF receptor superfamily, member 6) | Hs.244139 | Z70519 | 4 | 5 | 8 | 2 | 19 |
| FBXO32 | F-box protein 32 | Hs.403933 | BF244402 | 5 | 2 | 4 | 8 | 19 |
| FLJ10292 | | Hs.104650 | NM_018048 | 4 | 5 | 3 | 7 | 19 |
| FLJ43663 | | Hs.150556 | AL569506 | 6 | 1 | 11 | 1 | 19 |
| G1P3 | interferon, alpha-inducible protein (clone IFI-6-16) | Hs.523847 | NM_022873 | 0 | 8 | 3 | 8 | 19 |
| GALE | UDP-galactose-4-epimerase | Hs.632380 | NM_000403 | 2 | 5 | 11 | 1 | 19 |
| H6PD | hexose-6-phosphate dehydrogenase (glucose 1-dehydrogenase) | Hs.463511 | AK024548 | 0 | 9 | 3 | 7 | 19 |
| HIST1H4C | histone 1, H4c | Hs.46423 | NM_003542 | 3 | 7 | 3 | 6 | 19 |
| IGF1R | insulin-like growth factor 1 receptor | Hs.643120 | H05812 | 3 | 5 | 1 | 10 | 19 |
| IGSF4 | immunoglobulin superfamily, member 4 | Hs.370510 | AL519710 | 3 | 5 | 9 | 2 | 19 |
| JMJD1C | jumonji domain containing 1C | Hs.413416 | BF002296 | 7 | 2 | 7 | 3 | 19 |
| KIAA0582 | KIAA0582 | Hs.709257 | AB011154 | 1 | 4 | 3 | 11 | 19 |
| KIAA0907 | KIAA0907 | Hs.660143 | BF508948 | 2 | 6 | 5 | 6 | 19 |
| KIAA1333 | KIAA1333 | Hs.509008 | AA642341 | 11 | 0 | 4 | 4 | 19 |
| KIAA1799 | | Hs.659291 | AA905286 | 6 | 4 | 4 | 5 | 19 |
| KLF5 | Kruppel-like factor 5 (intestinal) | Hs.508234 | AF132818 | 7 | 4 | 2 | 6 | 19 |
| KLF9 | Kruppel-like factor 9 | Hs.150557 | AI690205 | 3 | 2 | 3 | 11 | 19 |
| KRT19 | keratin 19 | Hs.654568 | NM_002276 | 4 | 5 | 5 | 5 | 19 |
| LARP2 | La ribonucleoprotein domain family, member 2 | Hs.657067 | AI767732 | 9 | 1 | 7 | 2 | 19 |
| LAT1-3TM | | Hs.460179 | BG180003 | 6 | 2 | 8 | 3 | 19 |
| LOC283788 | | Hs.696627 | N50864 | 5 | 4 | 6 | 4 | 19 |
| LOC284009 | | Hs.632237 | AL045793 | 5 | 3 | 5 | 6 | 19 |

| | | | | | | | | |
|---|---|---|---|---|---|---|---|---|
| LOC285812 | | Hs.593631 | N52572 | 5 | 2 | 11 | 1 | 19 |
| LOC338620 | | Hs.660499 | AI435839 | 3 | 4 | 7 | 5 | 19 |
| MAFF | v-maf musculoaponeurotic fibrosarcoma oncogene homolog F (avian) | Hs.517617 | NM_012323 | 8 | 3 | 5 | 3 | 19 |
| MBNL2 | muscleblind-like 2 (Drosophila) | Hs.679513 | AF339768 | 3 | 6 | 9 | 1 | 19 |
| MGC45871 | | Hs.719324 | AI961778 | 1 | 7 | 6 | 5 | 19 |
| MRPS6 | mitochondrial ribosomal protein S6 | --- | AI867198 | 6 | 2 | 11 | 0 | 19 |
| MT1F | metallothionein 1F (functional) | Hs.513626 | BF246115 | 2 | 7 | 6 | 4 | 19 |
| MTERFD3 | MTERF domain containing 3 | Hs.5009 | BF697312 | 8 | 1 | 5 | 5 | 19 |
| MXD1 | MAX dimerization protein 1 | Hs.468908 | AW071793 | 7 | 1 | 8 | 3 | 19 |
| NAP1L3 | nucleosome assembly protein 1-like 3 | Hs.21365 | NM_004538 | 5 | 1 | 9 | 4 | 19 |
| NEU1 | sialidase 1 (lysosomal sialidase) | Hs.520037 | U84246 | 4 | 2 | 11 | 2 | 19 |
| NUAK1 | NUAK family, SNF1-like kinase, 1 | Hs.719171 | NM_014840 | 3 | 4 | 8 | 4 | 19 |
| PCK2 | phosphoenolpyruvate carboxykinase 2 (mitochondrial) | Hs.75812 | NM_004563 | 0 | 7 | 4 | 8 | 19 |
| PHF19 | PHD finger protein 19 | Hs.460124 | BE544837 | 6 | 3 | 4 | 6 | 19 |
| PRDM1 | PR domain containing 1, with ZNF domain | Hs.436023 | AI692659 | 0 | 10 | 4 | 5 | 19 |
| PTTG1 | pituitary tumor-transforming 1 | Hs.350966 | NM_004219 | 4 | 5 | 6 | 4 | 19 |
| RALGPS2 | Ral GEF with PH domain and SH3 binding motif 2 | Hs.709811 | AW003297 | 4 | 2 | 3 | 10 | 19 |
| RFC3 | replication factor C (activator 1) 3, 38kDa | Hs.115474 | BC000149 | 10 | 1 | 3 | 5 | 19 |
| RPL37A | ribosomal protein L37a | Hs.433701 | AU155515 | 7 | 1 | 5 | 6 | 19 |
| S100A4 | S100 calcium binding protein A4 (calcium protein, calvasculin, metastas | Hs.654444 | NM_002961 | 1 | 8 | 5 | 5 | 19 |
| SEC22L1 | SEC22 vesicle trafficking protein-like 1 (S. cerevisiae) | Hs.661819 | AU154125 | 3 | 4 | 9 | 3 | 19 |
| SFRS1 | splicing factor, arginine/serine-rich 1 (splicing factor 2, alternate splicin | Hs.68714 | AL521786 | 10 | 0 | 3 | 6 | 19 |
| SIGIRR | | Hs.501624 | NM_021805 | 0 | 9 | 1 | 9 | 19 |
| SLC27A3 | solute carrier family 27 (fatty acid transporter), member 3 | Hs.438723 | BC003654 | 2 | 7 | 4 | 6 | 19 |
| SLC2A3 | solute carrier family 2 (facilitated glucose transporter), member 3 | Hs.419240 | AL110298 | 4 | 2 | 7 | 6 | 19 |

| | | | | | | | | |
|---|---|---|---|---|---|---|---|---|
| SOCS1 | suppressor of cytokine signaling 1 | Hs.50640 | AB005043 | 4 | 3 | 2 | 10 | 19 |
| SPRY2 | sprouty homolog 2 (Drosophila) | Hs.18676 | NM_005842 | 4 | 3 | 10 | 2 | 19 |
| SSFA2 | sperm specific antigen 2 | Hs.591602 | AL556611 | 6 | 1 | 9 | 3 | 19 |
| SULF2 | sulfatase 2 | Hs.162016 | AL133001 | 0 | 8 | 7 | 4 | 19 |
| SYNGR2 | synaptogyrin 2 | Hs.464210 | NM_004710 | 2 | 5 | 6 | 6 | 19 |
| SYTL2 | synaptotagmin-like 2 | Hs.369520 | AB046817 | 4 | 4 | 6 | 5 | 19 |
| TCEA2 | transcription elongation factor A (SII), 2 | Hs.505004 | NM_003195 | 0 | 8 | 2 | 9 | 19 |
| TNFRSF10A | tumor necrosis factor receptor superfamily, member 10a | Hs.591834 | W65310 | 4 | 3 | 6 | 6 | 19 |
| TPX2 | TPX2, microtubule-associated, homolog (Xenopus laevis) | Hs.719145 | AF098158 | 5 | 5 | 3 | 6 | 19 |
| TSGA14 | testis specific, 14 | Hs.368315 | AJ278890 | 5 | 4 | 3 | 7 | 19 |
| TYMS | thymidylate synthetase | Hs.592338 | NM_001071 | 4 | 7 | 2 | 6 | 19 |
| UQCRC2 | ubiquinol-cytochrome c reductase core protein II | Hs.528803 | AI961429 | 5 | 4 | 4 | 6 | 19 |
| USP32 | ubiquitin specific peptidase 32 | Hs.132868 | AW268357 | 3 | 6 | 7 | 3 | 19 |
| ZNF677 | zinc finger protein 677 | Hs.700718 | AI816281 | 8 | 3 | 5 | 3 | 19 |
| ABLIM1 | actin binding LIM protein 1 | Hs.438236 | BC002448 | 2 | 7 | 2 | 7 | 18 |
| ADM | adrenomedullin | Hs.441047 | NM_001124 | 5 | 1 | 5 | 7 | 18 |
| ALDOC | aldolase C, fructose-bisphosphate | Hs.155247 | NM_005165 | 1 | 8 | 5 | 4 | 18 |
| AMSH-LP | | Hs.16229 | AI638611 | 5 | 4 | 5 | 4 | 18 |
| ANAPC7 | anaphase promoting complex subunit 7 | Hs.524741 | AL137586 | 4 | 5 | 5 | 4 | 18 |
| APRIN | androgen-induced proliferation inhibitor | Hs.646051 | BF111072 | 6 | 3 | 7 | 2 | 18 |
| ARMCX2 | armadillo repeat containing, X-linked 2 | Hs.48924 | NM_014782 | 3 | 4 | 5 | 6 | 18 |
| ASAH1 | N-acylsphingosine amidohydrolase (acid ceramidase) 1 | Hs.527412 | BC016828 | 5 | 4 | 7 | 2 | 18 |
| ASK | | Hs.485380 | NM_006716 | 10 | 0 | 3 | 5 | 18 |
| B4GALT5 | UDP-Gal:betaGlcNAc beta 1,4- galactosyltransferase, polypeptide 5 | Hs.370487 | BF691447 | 5 | 3 | 7 | 3 | 18 |
| BRAF | v-raf murine sarcoma viral oncogene homolog B1 | Hs.600998 | AW184034 | 6 | 4 | 4 | 4 | 18 |

| | | | | | | | | |
|---|---|---|---|---|---|---|---|---|
| C1orf24 | chromosome 1 open reading frame 24 | Hs.518662 | AF288391 | 2 | 4 | 6 | 6 | 18 |
| C6orf106 | chromosome 6 open reading frame 106 | Hs.643498 | AL523965 | 7 | 3 | 8 | 0 | 18 |
| C6orf173 | chromosome 6 open reading frame 173 | Hs.486401 | BG492359 | 4 | 5 | 2 | 7 | 18 |
| C9orf81 | chromosome 9 open reading frame 81 | Hs.374421 | AW268594 | 4 | 2 | 10 | 2 | 18 |
| CCNL1 | cyclin L1 | Hs.4859 | AY034790 | 6 | 1 | 4 | 7 | 18 |
| CDCA5 | cell division cycle associated 5 | Hs.434886 | BE614410 | 5 | 4 | 3 | 6 | 18 |
| CDCA7 | cell division cycle associated 7 | Hs.470654 | AY029179 | 5 | 3 | 3 | 7 | 18 |
| CDKL5 | cyclin-dependent kinase-like 5 | Hs.696079 | AI611074 | 5 | 3 | 4 | 6 | 18 |
| CIRBP | cold inducible RNA binding protein | Hs.634522 | AL565767 | 8 | 4 | 3 | 3 | 18 |
| CLK4 | CDC-like kinase 4 | Hs.406557 | AW975057 | 7 | 3 | 5 | 3 | 18 |
| CMKOR1 | chemokine orphan receptor 1 | Hs.471751 | AI817041 | 0 | 10 | 3 | 5 | 18 |
| CNNM3 | cyclin M3 | Hs.711617 | NM_017623 | 0 | 8 | 7 | 3 | 18 |
| COP1 | | Hs.348365 | NM_052889 | 1 | 9 | 7 | 1 | 18 |
| CRIP1 | cysteine-rich protein 1 (intestinal) | Hs.70327 | NM_001311 | 0 | 7 | 4 | 7 | 18 |
| CRLF3 | cytokine receptor-like factor 3 | Hs.658293 | AA843122 | 4 | 3 | 6 | 5 | 18 |
| CYBA | cytochrome b-245, alpha polypeptide | Hs.513803 | NM_000101 | 3 | 9 | 5 | 1 | 18 |
| DAF | decay accelerating factor for complement (CD55, Cromer blood group s | Hs.126517 | NM_000574 | 5 | 3 | 4 | 6 | 18 |
| DCBLD2 | discoidin, CUB and LCCL domain containing 2 | Hs.203691 | AI378788 | 6 | 1 | 10 | 1 | 18 |
| DDIT3 | DNA-damage-inducible transcript 3 | Hs.505777 | BC003637 | 5 | 1 | 10 | 2 | 18 |
| DDIT4 | DNA-damage-inducible transcript 4 | Hs.523012 | NM_019058 | 0 | 6 | 4 | 8 | 18 |
| DKFZp547E087 | | Hs.648439 | BE972419 | 8 | 2 | 5 | 3 | 18 |
| DLG7 | discs, large homolog 7 (Drosophila) | Hs.77695 | NM_014750 | 5 | 3 | 3 | 7 | 18 |
| DPP4 | dipeptidylpeptidase 4 (CD26, adenosine deaminase complexing protei | Hs.368912 | M80536 | 4 | 4 | 3 | 7 | 18 |
| DTX3L | deltex 3-like (Drosophila) | Hs.518201 | AA577672 | 2 | 5 | 4 | 7 | 18 |
| ECT2 | epithelial cell transforming sequence 2 oncogene | Hs.518299 | NM_018098 | 6 | 3 | 4 | 5 | 18 |
| EFNB2 | ephrin-B2 | Hs.149239 | BF001670 | 7 | 2 | 2 | 7 | 18 |
| EIF4EBP1 | eukaryotic translation initiation factor 4E binding | Hs.411641 | AB044548 | 0 | 4 | 8 | 6 | 18 |

| | | | | | | | | |
|---|---|---|---|---|---|---|---|---|
| | protein 1 | | | | | | | |
| EIF5A | eukaryotic translation initiation factor 5A | Hs.534314 | NM_001970 | 11 | 1 | 4 | 2 | 18 |
| ETV1 | ets variant gene 1 | Hs.22634 | BE881590 | 2 | 2 | 9 | 5 | 18 |
| EXOSC9 | exosome component 9 | Hs.58974 | AI346350 | 6 | 2 | 4 | 6 | 18 |
| FBXO6 | F-box protein 6 | Hs.464419 | AF129536 | 3 | 6 | 4 | 5 | 18 |
| FEN1 | flap structure-specific endonuclease 1 | Hs.409065 | NM_004111 | 4 | 5 | 4 | 5 | 18 |
| FKBP11 | FK506 binding protein 11, 19 kDa | Hs.655103 | NM_016594 | 3 | 3 | 8 | 4 | 18 |
| FLJ11029 | | Hs.633096 | BG165011 | 5 | 7 | 2 | 4 | 18 |
| FLJ39370 | | Hs.23439 | AI110850 | 5 | 3 | 6 | 4 | 18 |
| G0S2 | G0/G1switch 2 | Hs.432132 | NM_015714 | 2 | 3 | 9 | 4 | 18 |
| GBP2 | guanylate binding protein 2, interferon-inducible | Hs.386567 | NM_004120 | 0 | 8 | 1 | 9 | 18 |
| GRIP2 | glutamate receptor interacting protein 2 | Hs.529488 | BG150485 | 3 | 3 | 9 | 3 | 18 |
| GRN | granulin | Hs.514220 | AK023348 | 0 | 8 | 8 | 2 | 18 |
| HCFC1R1 | host cell factor C1 regulator 1 (XPO1 dependant) | Hs.719259 | AA436930 | 0 | 8 | 4 | 6 | 18 |
| HIST1H2AC | histone 1, H2ac | Hs.699831 | AL353759 | 5 | 2 | 5 | 6 | 18 |
| HIST1H2BD | histone 1, H2bd | Hs.591797 | BC002842 | 5 | 2 | 6 | 5 | 18 |
| HIST2H2AA | histone 2, H2aa | Hs.530461 | AI313324 | 4 | 5 | 4 | 5 | 18 |
| HMMR | hyaluronan-mediated motility receptor (RHAMM) | Hs.72550 | NM_012485 | 7 | 3 | 3 | 5 | 18 |
| HNRPA1 | heterogeneous nuclear ribonucleoprotein A1 | Hs.656277 | AI144007 | 9 | 1 | 4 | 4 | 18 |
| HPS3 | Hermansky-Pudlak syndrome 3 | --- | AA215451 | 6 | 2 | 8 | 2 | 18 |
| IER3 | immediate early response 3 | Hs.591785 | NM_003897 | 4 | 5 | 8 | 1 | 18 |
| IGFBP6 | insulin-like growth factor binding protein 6 | Hs.274313 | NM_002178 | 1 | 5 | 4 | 8 | 18 |
| IL6R | interleukin 6 receptor | Hs.709210 | AV700030 | 3 | 6 | 3 | 6 | 18 |
| IRS2 | insulin receptor substrate 2 | Hs.442344 | BF700086 | 6 | 2 | 2 | 8 | 18 |
| ITGAV | integrin, alpha V (vitronectin receptor, alpha polypeptide, antigen CD5 | Hs.656287 | AU144005 | 4 | 6 | 8 | 0 | 18 |
| JAK2 | Janus kinase 2 (a protein tyrosine kinase) | Hs.656213 | AF001362 | 6 | 3 | 4 | 5 | 18 |
| KIAA1718 | | Hs.308710 | BE217882 | 5 | 2 | 6 | 5 | 18 |
| KIF23 | kinesin family member 23 | Hs.270845 | NM_004856 | 7 | 3 | 3 | 5 | 18 |

| | | | | | | | | |
|---|---|---|---|---|---|---|---|---|
| KNTC2 | kinetochore associated 2 | Hs.414407 | NM_006101 | 4 | 3 | 4 | 7 | 18 |
| KRT7 | keratin 7 | Hs.411501 | BC002700 | 3 | 4 | 5 | 6 | 18 |
| LOC145474 | | Hs.658241 | AU158212 | 4 | 5 | 7 | 2 | 18 |
| LOC158402 | | Hs.494822 | BE504242 | 3 | 4 | 4 | 7 | 18 |
| LOC284454 | | Hs.436426 | BU617052 | 6 | 1 | 10 | 1 | 18 |
| LOC284801 | | Hs.370699 | AI825833 | 7 | 5 | 1 | 5 | 18 |
| LOC441168 | | Hs.381220 | AV734646 | 5 | 7 | 3 | 3 | 18 |
| LOC90355 | | Hs.482976 | AL565741 | 9 | 0 | 6 | 3 | 18 |
| MAP3K4 | mitogen-activated protein kinase kinase kinase 4 | Hs.594896 | AI633559 | 7 | 1 | 9 | 1 | 18 |
| MASK | | Hs.444247 | AF344882 | 10 | 1 | 4 | 3 | 18 |
| MCM5 | MCM5 minichromosome maintenance deficient 5, cell division cycle 46 | Hs.517582 | AA807529 | 5 | 6 | 2 | 5 | 18 |
| METTL4 | methyltransferase like 4 | Hs.126888 | AA764787 | 7 | 2 | 3 | 6 | 18 |
| MGC12965 | | Hs.82502 | BC019340 | 3 | 7 | 1 | 7 | 18 |
| MGC20481 | | Hs.654661 | AK098781 | 5 | 4 | 6 | 3 | 18 |
| MLF1 | myeloid leukemia factor 1 | Hs.85195 | NM_022443 | 6 | 2 | 8 | 2 | 18 |
| MLSTD1 | male sterility domain containing 1 | Hs.719237 | H16791 | 1 | 6 | 1 | 10 | 18 |
| MUC1 | mucin 1, transmembrane | Hs.89603 | AI610869 | 1 | 8 | 0 | 9 | 18 |
| MUM1 | melanoma associated antigen (mutated) 1 | Hs.713099 | AI860326 | 7 | 3 | 4 | 4 | 18 |
| MXRA7 | matrix-remodelling associated 7 | Hs.250723 | BE966768 | 3 | 3 | 8 | 4 | 18 |
| NOG | noggin | Hs.248201 | AL575177 | 1 | 5 | 6 | 6 | 18 |
| NQO1 | NAD(P)H dehydrogenase, quinone 1 | Hs.406515 | NM_000903 | 9 | 4 | 3 | 2 | 18 |
| OPN3 | opsin 3 (encephalopsin, panopsin) | Hs.654545 | AU155565 | 7 | 2 | 3 | 6 | 18 |
| OSTM1 | osteopetrosis associated transmembrane protein 1 | Hs.719130 | AV713913 | 7 | 1 | 8 | 2 | 18 |
| PDLIM1 | PDZ and LIM domain 1 (elfin) | Hs.368525 | BC000915 | 2 | 5 | 5 | 6 | 18 |
| PKP4 | plakophilin 4 | Hs.664068 | AA913146 | 6 | 4 | 8 | 0 | 18 |
| PLCB1 | phospholipase C, beta 1 (phosphoinositide-specific) | Hs.431173 | AL049593 | 5 | 3 | 3 | 7 | 18 |
| PLEKHA1 | pleckstrin homology domain containing, family A (phosphoinositide bi | Hs.643512 | NM_021622 | 4 | 4 | 8 | 2 | 18 |

| | | | | | | | | |
|---|---|---|---|---|---|---|---|---|
| PPIG | peptidyl-prolyl isomerase G (cyclophilin G) | Hs.593163 | AW194716 | 9 | 1 | 1 | 7 | 18 |
| PRDX6 | peroxiredoxin 6 | Hs.658605 | N55072 | 3 | 3 | 12 | 0 | 18 |
| RACGAP1 | Rac GTPase activating protein 1 | Hs.505469 | AU153848 | 4 | 5 | 3 | 6 | 18 |
| RASA1 | RAS p21 protein activator (GTPase activating protein) 1 | Hs.657767 | AK021812 | 2 | 4 | 5 | 7 | 18 |
| RDH10 | retinol dehydrogenase 10 (all-trans) | Hs.244940 | AV697515 | 2 | 5 | 5 | 6 | 18 |
| RNPC2 | RNA-binding region (RNP1, RRM) containing 2 | Hs.282901 | BE466173 | 1 | 6 | 6 | 5 | 18 |
| SCML1 | sex comb on midleg-like 1 (Drosophila) | Hs.109655 | NM_006746 | 9 | 0 | 3 | 6 | 18 |
| SESN2 | sestrin 2 | Hs.469543 | AL136551 | 5 | 1 | 10 | 2 | 18 |
| SESN3 | sestrin 3 | Hs.120633 | BE883841 | 6 | 2 | 6 | 4 | 18 |
| SGK | serum/glucocorticoid regulated kinase | Hs.510078 | NM_005627 | 4 | 2 | 4 | 8 | 18 |
| SLC2A14 | solute carrier family 2 (facilitated glucose transporter), member 14 | Hs.419240 | AA778684 | 5 | 5 | 4 | 4 | 18 |
| SNRPN | small nuclear ribonucleoprotein polypeptide N | --- | AW770748 | 5 | 1 | 6 | 6 | 18 |
| SPBC25 | spindle pole body component 25 homolog (S. cerevisiae) | Hs.421956 | AF225416 | 4 | 3 | 2 | 9 | 18 |
| STC1 | stanniocalcin 1 | Hs.25590 | U46768 | 3 | 3 | 10 | 2 | 18 |
| STX3A | syntaxin 3A | Hs.180711 | BE966922 | 4 | 4 | 9 | 1 | 18 |
| SYNGR3 | synaptogyrin 3 | Hs.435277 | NM_004209 | 2 | 7 | 6 | 3 | 18 |
| TBC1D8 | TBC1 domain family, member 8 (with GRAM domain) | Hs.469473 | AI348010 | 7 | 0 | 3 | 8 | 18 |
| THNSL1 | threonine synthase-like 1 (bacterial) | Hs.645274 | AI809864 | 3 | 3 | 6 | 6 | 18 |
| TNFRSF10D | tumor necrosis factor receptor superfamily, member 10d, decoy with tr | Hs.213467 | AI738556 | 4 | 2 | 5 | 7 | 18 |
| TPP1 | tripeptidyl peptidase I | Hs.523454 | BG231932 | 0 | 4 | 11 | 3 | 18 |
| TRIB2 | tribbles homolog 2 (Drosophila) | Hs.467751 | BC002637 | 3 | 5 | 5 | 5 | 18 |
| TRPC1 | transient receptor potential cation channel, subfamily C, member 1 | Hs.250687 | NM_003304 | 2 | 4 | 4 | 8 | 18 |
| TSPAN13 | tetraspanin 13 | Hs.364544 | NM_014399 | 6 | 1 | 9 | 2 | 18 |
| VCAM1 | vascular cell adhesion molecule 1 | Hs.109225 | NM_001078 | 6 | 3 | 5 | 4 | 18 |
| VEGF | vascular endothelial growth factor | Hs.73793 | AF022375 | 4 | 4 | 9 | 1 | 18 |

| | | | | | | | | |
|---|---|---|---|---|---|---|---|---|
| ZBTB10 | zinc finger and BTB domain containing 10 | Hs.591868 | AK024296 | 8 | 2 | 3 | 5 | 18 |
| ZC3H11A | zinc finger CCCH-type containing 11A | --- | AA280627 | 3 | 7 | 5 | 3 | 18 |
| ZNF117 | zinc finger protein 117 (HPF9) | Hs.250693 | AW058673 | 3 | 2 | 3 | 10 | 18 |
| ZNF292 | zinc finger protein 292 | Hs.485892 | BF223237 | 8 | 4 | 3 | 3 | 18 |
| ZNF365 | zinc finger protein 365 | Hs.22653 | NM_014951 | 3 | 5 | 5 | 5 | 18 |
