# Supplementary material for: Identification of evolutionarily conserved genetic regulators of cellular aging
Source: Aging Cell. 2010 Dec;9(6):1084–97. doi: 10.1111/j.1474-9726.2010.00637.x (PMC2997327; doi:10.1111/j.1474-9726.2010.00637.x)
Supplement: Supplementary file 7 [file acel0009-1084-SD7.pdf]

Probe Set ID	Gene Symbol	Gene Title	Ensembl	SGD accession number
1779132_at	AIP1	Actin cortical patch component, interacts with the actin depolymerizing fact	YMR092C	S000004698
1776624_at	ALD4	Mitochondrial aldehyde dehydrogenase, required for growth on ethanol and	YOR374W	S000005901
1771943_at	ALD5	Mitochondrial aldehyde dehydrogenase, involved in regulation or biosynthe	YER073W	S000000875
1779402_at	ALG5	UDP-glucose:dolichyl-phosphate glucosyltransferase, involved in asparagine	YPL227C	S000006148
1778563_at	ALT2	Putative alanine transaminase (glutamic pyruvic transaminase)	YDR111C	S000002518
1770280_at	APM1	Mu1-like medium subunit of the clathrin-associated protein complex (AP-1);	YPL259C	S000006180
1776418_at	ARF1	ADP-ribosylation factor, GTPase of the Ras superfamily involved in regulatio	YDL192W	S000002351
1774082_at	ATG18	Phosphoinositide binding protein required for vesicle formation in autopha	YFR021W	S000001917
1779762_at	CAF40	Evolutionarily conserved subunit of the CCR4-NOT complex involved in contr	YNL288W	S000005232
1776167_at	CHD1	Nucleosome remodeling factor that functions in regulation of transcription e	YER164W	S000000966
1777009_at	CHK1	Serine/threonine kinase and DNA damage checkpoint effector, mediates cel	YBR274W	S000000478
1777889_at	CHL1	Conserved nuclear protein required to establish sister-chromatid pairing dur	YPL008W	S000005929
1769973_at	CLB1	B-type cyclin involved in cell cycle progression; activates Cdc28p to promote	YGR108W	S000003340
1776774_at	CLB2	B-type cyclin involved in cell cycle progression; activates Cdc28p to promote	YPR119W	S000006323
1773548_at	CYC1	Cytochrome c, isoform 1; electron carrier of the mitochondrial intermembra	YJR048W	S000003809
1773888_at	CYS3	Cystathionine gamma-lyase, catalyzes one of the two reactions involved in t	YAL012W	S000000010
1775526_at	CYS4	Cystathionine beta-synthase, catalyzes the synthesis of cystathionine from s	YGR155W	S000003387
1769674_at	DBP1	Putative ATP-dependent RNA helicase of the DEAD-box protein family; muta	YPL119C	S000006040
1773176_at	DIE2	Dolichyl-phosphoglucose-dependent alpha-1,2 glucosyltransferase of the ER	YGR227W	S000003459
1772590_at	DNF1	Aminophospholipid translocase (flippase) that localizes primarily to the plas	YER166W	S000000968
1771781_at	DPH5	Methyltransferase required for synthesis of diphthamide, which is a modifie	YLR172C	S000004162
1774346_at	ENO1	Enolase I, a phosphopyruvate hydratase that catalyzes the conversion of 2-p	YGR254W	S000003486
1778104_at	ERG24	C-14 sterol reductase, acts in ergosterol biosynthesis; mutants accumulate th	YNL280C	S000005224
1777611_at	ERP6	Protein with similarity to Emp24p and Erv25p, member of the p24 family invo	YGL002W	S000002970
1779105_at	GSY2	Glycogen synthase, similar to Gsy1p; expression induced by glucose limitatio	YLR258W	S000004248
1778399_at	HAP3	Subunit of the heme-activated, glucose-repressed Hap2p/3p/4p/5p CCAAT-b	YBL021C	S000000117
1775160_at	HAT1	Catalytic subunit of the Hat1p-Hat2p histone acetyltransferase complex that	YPL001W	S000005922

1776159_at	HRT3	Putative SCF-ubiquitin ligase F-box protein, based on both genetic and physi	YLR097C	S000004087
1775235_at	HTA2	Histone H2A, core histone protein required for chromatin assembly and chro	YBL003C	S000000099
1775976_at	HXK1	Hexokinase isoenzyme 1, a cytosolic protein that catalyzes phosphorylation	YFR053C	S000001949
1777461_at	IBA57 /	Mitochondrial matrix protein involved in the incorporation of iron-sulfur clu	YJR122W	S000003883
1779660_at	IDP2	Cytosolic NADP-specific isocitrate dehydrogenase, catalyzes oxidation of iso	YLR174W	S000004164
1777183_at	INP53	Polyphosphatidylinositol phosphatase, dephosphorylates multiple phospha	YOR109W	S000005635
1769462_at	KGD1	Component of the mitochondrial alpha-ketoglutarate dehydrogenase compl	YIL125W	S000001387
1779214_at	LHS1	Molecular chaperone of the endoplasmic reticulum lumen, involved in poly	YKL073W	S000001556
1770534_at	LSB6	Type II phosphatidylinositol 4-kinase that binds Las17p, which is a homolog o	YJL100W	S000003636
1772003_at	LYS9	Saccharopine dehydrogenase (NADP+, L-glutamate-forming); catalyzes the f	YNR050C	S000005333
1770927_at	MAD2	Component of the spindle-assembly checkpoint complex, which delays the	YJL030W	S000003567
1772731_at	MRE11	Subunit of a complex with Rad50p and Xrs2p (MRX complex) that functions in	YMR224C	S000004837
1774349_at	MRT4	Protein involved in mRNA turnover and ribosome assembly, localizes to the	YKL009W	S000001492
1770036_at	MSH2	Protein that forms heterodimers with Msh3p and Msh6p that bind to DNA mi	YOL090W	S000005450
1776554_at	MSH5	Protein of the MutS family, forms a dimer with Msh4p that facilitates crossov	YDL154W	S000002313
1775548_at	MSH6	Protein required for mismatch repair in mitosis and meiosis, forms a comple	YDR097C	S000002504
1780201_at	MSW1	Mitochondrial tryptophanyl-tRNA synthetase	YDR268W	S000002676
1772812_at	NCR1	Vacuolar membrane protein that transits through the biosynthetic vacuolar	YPL006W	S000005927
1772041_at	NPL3	RNA-binding protein that promotes elongation, regulates termination, and c	YDR432W	S000002840
1772463_at	NUP170	Subunit of the nuclear pore complex (NPC), required for NPC localization of	YBL079W	S000000175
1769993_at	PCH2	Nucleolar component of the pachytene checkpoint, which prevents chromos	YBR186W	S000000390
1776029_at	PER1	Protein of the endoplasmic reticulum, required for GPI-phospholipase A2 ac	YCR044C	S000000640
1777099_at	PEX13	Integral peroxisomal membrane required for the translocation of peroxisom	YLR191W	S000004181
1769766_at	PHO89	Na+/Pi cotransporter, active in early growth phase; similar to phosphate tran	YBR296C	S000000500
1776390_at	PIM1	ATP-dependent Lon protease, involved in degradation of misfolded proteins	YBL022C	S000000118
1776097_at	PRS3	5-phospho-ribosyl-1(alpha)-pyrophosphate synthetase, synthesizes PRPP, w	YHL011C	S000001003
1773162_at	PRY1	Protein of unknown function	YJL079C	S000003615
1770697_at	PTC5	Mitochondrial type 2C protein phosphatase involved in regulation of pyruvat	YOR090C	S000005616

1777959_at	RAD27	5' to 3' exonuclease, 5' flap endonuclease, required for Okazaki fragment pro	YKL113C	S000001596
1774709_at	REV3	Catalytic subunit of DNA polymerase zeta, which is involved in DNA repair an	YPL167C	S000006088
1775076_at	RNH201	Ribonuclease H2 catalytic subunit, removes RNA primers during Okazaki frag	YNL072W	S000005016
1780044_at	RNR3	One of two large regulatory subunits of ribonucleotide-diphosphate reducta	YIL066C	S000001328
1770882_at	RPL13B	Protein component of the large (60S) ribosomal subunit, nearly identical to	YMR142C	S000004750
1769929_at	RPL22B	Protein component of the large (60S) ribosomal subunit, has similarity to Rpl	YFL034C-A	S000006436
1779599_at	RPL31A	Protein component of the large (60S) ribosomal subunit, nearly identical to	YDL075W	S000002233
1770284_at	RPL37A	Protein component of the large (60S) ribosomal subunit, has similarity to Rpl	YLR185W	S000004175
1779559_at	RPL43B	Protein component of the large (60S) ribosomal subunit, identical to Rpl43A	YJR094W-A	S000003855
1774801_at	SER1	3-phosphoserine aminotransferase, catalyzes the formation of phosphoseri	YOR184W	S000005710
1773718_at	SGT2	Glutamine-rich cytoplasmic protein of unknown function; contains tetratrico	YOR007C	S000005533
1774806_at	SHM1	Mitochondrial serine hydroxymethyltransferase, converts serine to glycine p	YBR263W	S000000467
1776295_at	SIZ1	SUMO/Smt3 ligase that promotes the attachment of sumo (Smt3p; small ubi	YDR409W	S000002817
1773984_at	SLH1	Putative RNA helicase related to Ski2p, involved in translation inhibition of	YGR271W	S000003503
1773993_at	SOD2	Mitochondrial superoxide dismutase, protects cells against oxygen toxicity;	YHR008C	S000001050
1778906_at	SPE2	S-adenosylmethionine decarboxylase, required for the biosynthesis of sper	YOL052C	S000005412
1779158_at	SSA3	ATPase involved in protein folding and the response to stress; plays a role in	YBL075C	S000000171
1774186_at	SSF1	Constituent of 66S pre-ribosomal particles, required for ribosomal large sub	YHR066W	S000001108
1772868_at	SSN3	Cyclin-dependent protein kinase, component of RNA polymerase II holoenz	YPL042C	S000005963
1776891_at	SSO1	Plasma membrane t-SNARE involved in fusion of secretory vesicles at the pl	YPL232W	S000006153
1774055_at	SYG1	Plasma membrane protein of unknown function; truncation and overexpress	YIL047C /// YIL047C-A	S000001309 /// S000028790
1772447_at	SYM1	Protein required for ethanol metabolism; induced by heat shock and localize	YLR251W	S000004241
1779786_s_at	TIF1 /// TIF2	Translation initiation factor eIF4A, identical to Tif2p; DEA(D/H)-box RNA heli	YJL138C /// YKR059W	S000001767 /// S000003674
1771027_at	TIF3	Translation initiation factor eIF-4B, has RNA annealing activity; contains an R	YPR163C	S000006367
1771925_at	TPK1	cAMP-dependent protein kinase catalytic subunit; promotes vegetative gro	YJL164C	S000003700
1774482_at	TRX1	Cytoplasmic thioredoxin isoenzyme of the thioredoxin system which protect	YLR043C	S000004033
1774387_at	UBC11	Ubiquitin-conjugating enzyme most similar in sequence to Xenopus ubiquiti	YOR339C	S000005866
1771212_at	UBC12	Enzyme that mediates the conjugation of Rub1p, a ubiquitin-like protein, to	YLR306W	S000004297

1774010_at	UGA1	Gamma-aminobutyrate (GABA) transaminase (4-aminobutyrate aminotransf	YGR019W	S000003251
1775990_at	YDC1	Alkaline dihydroceramidase, involved in sphingolipid metabolism; preferen	YPL087W	S000006008
1775390_at	YPK2	Protein kinase with similarity to serine/threonine protein kinase Ypk1p; func	YMR104C	S000004710
1774772_at	YTA7	Protein that localizes to chromatin and has a role in regulation of histone ge	YGR270W	S000003502
